# Evaluation of integrin αvβ_6_ cystine knot PET tracers to detect cancer and idiopathic pulmonary fibrosis

**DOI:** 10.1038/s41467-019-11863-w

**Published:** 2019-10-14

**Authors:** Richard H. Kimura, Ling Wang, Bin Shen, Li Huo, Willemieke Tummers, Fabian V. Filipp, Haiwei Henry Guo, Thomas Haywood, Lotfi Abou-Elkacem, Lucia Baratto, Frezghi Habte, Rammohan Devulapally, Timothy H. Witney, Yan Cheng, Suhas Tikole, Subhendu Chakraborti, Jay Nix, Christopher A. Bonagura, Negin Hatami, Joshua J. Mooney, Tushar Desai, Scott Turner, Richard S. Gaster, Andrea Otte, Brendan C. Visser, George A. Poultsides, Jeffrey Norton, Walter Park, Mark Stolowitz, Kenneth Lau, Eric Yang, Arutselvan Natarajan, Ohad Ilovich, Shyam Srinivas, Ananth Srinivasan, Ramasamy Paulmurugan, Juergen Willmann, Frederick T. Chin, Zhen Cheng, Andrei Iagaru, Fang Li, Sanjiv S. Gambhir

**Affiliations:** 1 Department of Radiology, Stanford University, Stanford, CA 94305 USA; 2 Department of Nuclear Medicine, Peking Union Medical College, Beijing, 100730 China; 3 Cancer Systems Biology, Institute of Computational Biology, Helmholtz Zentrum München, Ingolstädter Landstraße 1, D-85764 München, Germany; 4 School of Life Sciences Weihenstephan, Technical University München, Maximus-von-Imhof-Forum 3, D-85354 Freising, Germany; 5 Department of Chemistry, Mississippi State University, Mississippi State, MS 39762 USA; 6 Advanced Light Source, Lawrence Berkeley National Laboratory, Berkeley, CA 94720 USA; 7 Art Robbins Instruments, Sunnyvale, CA 94089 USA; 8 Department of Medicine, Stanford University, Stanford, CA 94305 USA; 9 Department of Internal Medicine, Stanford University, Stanford, CA 94305 USA; 10 Pliant Therapeutics, Redwood City, CA 94063 USA; 11 Department of Surgery, Stanford University, Stanford, CA 94305 USA; 12 Department of Pathology, Stanford University, Stanford, CA 95305 USA

**Keywords:** Protein design, Cancer imaging, Diagnostic markers, Solution-state NMR, X-ray crystallography

## Abstract

Advances in precision molecular imaging promise to transform our ability to detect, diagnose and treat disease. Here, we describe the engineering and validation of a new cystine knot peptide (knottin) that selectively recognizes human integrin αvβ_6_ with single-digit nanomolar affinity. We solve its 3D structure by NMR and x-ray crystallography and validate leads with 3 different radiolabels in pre-clinical models of cancer. We evaluate the lead tracer’s safety, biodistribution and pharmacokinetics in healthy human volunteers, and show its ability to detect multiple cancers (pancreatic, cervical and lung) in patients at two study locations. Additionally, we demonstrate that the knottin PET tracers can also detect fibrotic lung disease in idiopathic pulmonary fibrosis patients. Our results indicate that these cystine knot PET tracers may have potential utility in multiple disease states that are associated with upregulation of integrin α_v_β_6_.

## Abstract (web summary)

Knottin is a cystine knot peptide. Here, the authors develop a knottin-based tracer for positron emission tomography and demonstrate its ability to detect cancer and idiopathic pulmonary fibrosis through selective binding to integrin αvβ_6_.

## Introduction

Through the use of high affinity ligands, positron emission tomography (PET) imaging is an effective way to distinguish between diseased and healthy tissues. Accumulation of PET tracers in tissues provides a way to image the molecular signature of disease, such as cancer by targeting overexpressed cell surface proteins. Aberrant protein expression also occurs in other diseases such as idiopathic pulmonary fibrosis (IPF), a chronic, progressive fibrotic interstitial lung disease (ILD) of unknown cause^1^. Analogous to many cancers, prognosis in IPF is poor with a mean survival of ~3–5 years^2^.

Integrin receptors are the focus of extensive efforts aimed at the development of PET tracers^3–6^. Integrins are a family of proteins that mediate cellular adhesion to extracellular matrix (ECM) proteins. In normal cells, integrins transduce signals from the cell surface to support gene expression of multiple proteins that regulate differentiation, migration, proliferation, and apoptosis^7^. In certain diseases such as cancer, the expression of some integrins become dysregulated^8^. In a well-studied example, integrin α_v_β_3_ (the angiogenesis integrin) is highly overexpressed on both tumor-neovasculature, and the surface of some human cancer cells^8^. Other integrins, such as α_v_β_1_ and α_v_β_5_ can be biomarkers of specific types of cancer^8^. Similarly, fibrosing diseases are known to express unique subsets of integrins^2,9–11^. Expression expression profiles of different integrins have shown prognostic value, so detecting them in vivo may be useful for diagnosing, managing, or treating disease^8,12,13^. A number of PET tracer and therapeutics, such as the RGD derivatives, that target integrin receptors are currently undergoing clinical trials^5,12^.

A different member of the integrin family, α_v_β_6_, is overexpressed on the surface of many types of cancer cells^14^. Integrin α_v_β_6_ overexpression has been confirmed in oral squamous cell carcinoma^15^, pancreatic ductal adenocarcinomas (PDAC)^16^, intestinal gastric carcinomas^16^, ovarian cancer^17^, and stage III basal cell carcinoma^18^. Integrin α_v_β_6_ overexpression is a prognostic indicator of reduced survival in colon carcinoma^19^, non-small cell lung cancer^20^, cervical squamous carcinoma^21^, and gastric carcinoma^22^. Furthermore, α_v_β_6_ expression is associated with increased liver metastasis of colon cancer^23^. In contrast, its expression is undetectable in many normal tissues including ovary^17^, kidney, lung, skin^15^, and pancreas^16^. In recent studies, immunohistochemical analyses (IHC) revealed robust expression of α_v_β_6_ in PDAC compared to cancers of other systems^14,16^. Expression of α_v_β_6_ was reported to be significantly higher in PDAC compared to chronic pancreatitis, and its expression was observed in tumor-positive lymph nodes^24^. In well differentiated pancreatic tumors, elevated levels of α_v_β_6_ were reported in 100% of the samples (*n* = 34) by IHC^14,16^. Currently, different research groups, including ours, are developing peptide-based PET tracers to image α_v_β_6_ for cancer detection^25–27^.

Integrin α_v_β_6_ has also been the subject of numerous studies as a potential biomarker of fibrotic ILD, which include IPF, nonspecific interstitial pneumonitis (NSIP), chronic hypersensitivity pneumonitis (HP)^2,28^. α_v_β_6_ is a potent activator of transforming growth factor (TGF)β1, which enhances matrix deposition by fibroblasts in wound healing and fibrosis^29,30^. In a recent study, where a number of potential biomarker candidates were screened by tissue IHC, only α_v_β_6_ was found to be statistically associated with a poor prognosis in fibrotic ILDs. Levels of α_v_β_6_ correlate directly and significantly to the risk of death from IPF. A 25-month median survival was observed in patients with the highest levels of α_v_β_6_ expression^2^.

Targeted PET ligands created by directed evolution strategies often demonstrate exquisite selectivity and high binding affinity their targets^31,32^. A given PET tracer’s pharmacokinetic profile is a function of its biochemical and biophysical characteristics, such as the overall electrostatic charge and molecular weight. Cystine knot peptides (knottins) are small (~4 kDa) peptides characterized by three threaded disulfide bonds arranged in a topological knot; this stabilizing core motif is known as a cystine knot^31,33^. Solvent exposed loops, that can be bioactive, extend from this knotted core. In our studies, we have engineered both high-affinity antigen recognition (*K*_D_~1 nM) and pharmacokinetic stabilizers into these loops^26^. Since peptides are generally small, modification of a single amino acid in the framework could have a dramatic effect on overall tracer pharmacokinetics. One advantage of the knottin scaffold is that their backbone residues are highly variable such that they can be fine-tuned to greatly increase tumor uptake or decrease renal uptake in mouse models^26^. In this study, we developed several pharmacokinetically stabilized α_v_β_6_ knottins and evaluated their ability to image disease in living systems as [^64^Cu]1,4,7,10-Tetraazacyclododecane-1,4,7,10-tetraacetic acid (DOTA), [^68^Ga]2,2′-(7-(1-carboxy-4-((2,5-dioxopyrrolidin-1-yl)oxy)-4-oxobutyl)-1,4,7-triazonane-1,4-diyl)diacetic acid (NODAGA) and [^18^F]fluoropropyl (FP) PET tracers.

Here, we describe the development and validation of knottin PET tracers that selectively recognizes human α_v_β_6_ with single-digit nanomolar affinity. We solve its 3D structure by NMR and x-ray crystallography and validate leads with 3 different radiolabels in pre-clinical models of cancer. We evaluate the lead tracer’s safety and performance in healthy human volunteers and show its ability to detect multiple cancers (pancreatic, cervical, and lung). Additionally, we demonstrate the knottin PET tracer’s ability to detect fibrotic lung disease in IPF patients. Our studies show the cystine knot PET tracers’ potential in multiple disease states associated with elevated α_v_β_6_.

## Results

### Development of the lead candidate R_0_1-MG

**Fig. 1:**
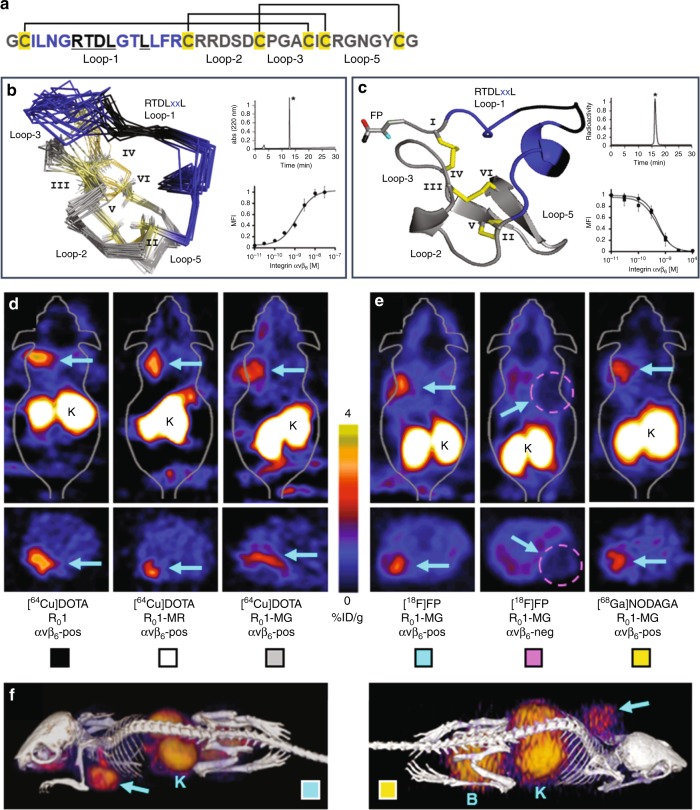
Summary of the knottin PET ligand. **a** Primary structure of lead candidate R_0_1-MG. The engineered active loop-1 is shown in blue. The integrin a_v_β_6_ core binding motif, RTDLxxL, is shown in black. The framework residues are shown in gray. Cysteine residues are shown in yellow and the pattern of disulfide bonds are indicated by the connecting lines. **b** Ensemble of 10 lowest energy three-dimensional ^1^H NMR of R_0_1-MG structures. **c** Crystal structure of non-radioactive reference standard, [^19^F]FP-R_0_1-MG-F2, at <1 Å. The carbonyl oxygen of the ester is shown in cyan. The fluorine atom is shown in red. The methyl carbon is shown in black. The insert on top shows the RP-HPLC trace of R_0_1-MG, and the radio-RP-HPLC trace of the purified clinical-grade PET tracer [^18^F]FP-R_0_1-MG-F2, respectively. The product is indicated by the asterisk. The insert on the bottom shows **b** the equilibrium binding curve of between R_0_1-MG expressed on yeast surface and soluble human integrin αvβ_6_ (*K*_D_ = 1.24 ± 0.21 nM S.D.), and **c** the competition binding between unlabeled R_0_1-MG (IC_50_ = 0.61 ± 0.31 nM S.D., circles) or the N-terminus labeled version [^19^F]FP-R_0_1-MG-F2 (IC_50_ = 0.56 ± 0.46 nM S.D., squares) vs. yeast surface expressed R_0_1-MG, respectively. **d** Comparison at 1 h post injection of the top 3 [^64^Cu]DOTA-labeled PET tracer candidates, R_0_1 (black), R_0_1-MR (white) and R_0_1-MG (gray). **e** Comparison at 1 h post injection of the [^18^F]FP labeled lead candidate R_0_1-MG in integrin αvβ_6_ positive (cyan) vs. αvβ_6_ negative tumor models (magenta, dashed circle in the figure). In vivo validation of the [^68^Ga]NODAGA-R_0_1-MG at 1 h post injection (yellow). **f** Volume rendered PET/CT images of [^18^F]FP-R_0_1-MG (cyan) and [^68^Ga]NODAGA-R_0_1-MG (yellow) in integrin avβ_6_ positive models at 1 h post injection. **d**–**f** The cyan arrows point to the tumor. The letters K and B represent the kidneys and bladder, respectively. (**d**–**f** and Table 3, below) Box colors are colored legends that cross-relate the images to the quantification table

Three cystine knot peptides (R_0_1, R_0_1-MG, R_0_1-MR) were engineered for stability, and high-affinity molecular recognition of human α_v_β_6_. Equilibrium binding assays indicated that all variants bind α_v_β_6_ with low single-digit nanomolar binding affinity (*K*_D_~1 nM, Supplementary Fig. 1). As a result of comparative imaging studies in vivo, R_0_1-MG emerged as the lead translational candidate (Fig. 1a). It was produced by cGMP solid phase peptide synthesis, and its mass was verified by MALDI-TOF-MS (Supplementary Table 1). In addition, the three-dimensional (3D) ^1^H-NMR solution structure of R_0_1-MG provided evidence that the cystine knot structural element, which defines knottin peptides, was conserved throughout the directed evolution process (Fig. 1b, Table 1). Furthermore, x-ray crystallography studies confirmed a disulfide bond pattern consistent with the cystine knot motif (Fig. 1c, Supplementary Fig. 2, Table 2). Competition binding assays demonstrated that the N-terminus imaging label, located ~20 Å from the RTDL integrin-recognition motif in active loop-1, did not adversely affect the tracer’s ability to bind α_v_β_6_ with high-affinity. The half-maximal inhibitory concentration (IC_50_) values for the unlabeled precursor and FP-labeled standard were 0.61 ± 0.31 nM and 0.56 ± 0.46 nM S.D., respectively (Fig. 1c insert, Supplementary Fig. 3).

**Table 1:** NMR refinement statistics

NMR distance and dihedral constraints	R_0_1-MG
Distance constraints	
Total NOE	225
Intra-residue	2
Inter-residue	84
Sequential (|*i - j*| = 1)	86
Medium -range (|*i - j*| < 4)	42
Long-range (|*i - j*| > 5)	97
Total dihedral angle restraints	
ϕ	35
ψ	37
Structure statistics	
Average pairwise r.m.s. deviation based on an ensemble of 20 structures (Å)	0.322

Structural Refinement Statistics. The table corresponds to the NMR structure shown in Fig. 1b

**Table 2:** X-ray crystallography data collection and refinement statistics

Data Collection	[^19^F]FP-R_0_1-MG-F2
Space group	*p*2_1_2_1_2_1_
Cell dimensions (Å)	16.90 × 44.40 × 66.71
Resolution (Å)	1–36.8200
*R*_merge_	0.036
*I/σI*	32.3
Completeness (%)	94.1
Redundancy	29
Refinement	
Resolution (Å)	1–36.8200
No. reflections.	24257
*R*_work_/*R*_free_	0.3590/0.3640
*B*-factors	10.1520
R.M.S. deviations	0.032

Structural Refinement Statistics. The table corresponds to the crystal structure shown in Fig. 1c

In order to select the most promising ligand for clinical translation, [^64^Cu]DOTA-R_0_1, [^64^Cu]DOTA-R_0_1-MR, and [^64^Cu]DOTA-R_0_1-MG were first evaluated in mouse models with α_v_β_6_ expressing BxPC3 pancreatic cancer xenografts (Fig. 1d, Supplementary Fig. 4, and Table 3). At 1 h post injection (p.i.), accumulation of tracer in the tumors were comparable at ~3% injected dose per gram (%ID g^−1^) across the variants (Table 3). However, [^64^Cu]DOTA-R_0_1-MR was eliminated from further consideration because its kidney uptake (~90 %ID g^−1^) was approximately 3-fold higher than the other two candidates (~30 %ID g^−1^, Table 3 and Supplementary Table 2). Based on these initial studies, R_0_1-MG was selected as the lead translational candidate and evaluated as a radiofluorinated PET tracer.

**Table 3:** PET quantification table

Legend	PET Tracer	αvβ_6_	Tumor	Liver	Kidney	Muscle	T:M Ratio
				(average % injected dose gram^−1^ ± SD)			
	[^64^Cu]DOTA-R_0_1	(+)	3.07 ± 0.17	2.26 ± 0.11	30.30 ± 2.33	0.433 ± 0.008	7.09 ± 0.27
	[^64^Cu]DOTA-R_0_1-MR	(+)	3.45 ± 0.34	4.63 ± 0.09	89.95 ± 3.64	0.536 ± 0.004	6.43 ± 0.58
	[^64^Cu]DOTA-R_0_1-MG	(+)	2.74 ± 0.35	2.14 ± 0.38	28.35 ± 2.61	0.472 ± 0.003	5.80 ± 0.72
	[^18^F]FP-R_0_1-MG	(+)	2.72 ± 0.60	0.88 ± 0.10	15.10 ± 1.28	0.490 ± 0.116	5.59 ± 0.55
	[^18^F]FP-R_0_1-MG	(−)	0.47 ± 0.12	0.87 ± 0.08	22.36 ± 1.50	0.455 ± 0.057	1.02 ± 0.19
	[^68^Ga]NODAGA-R_0_1-MG	(+)	2.78 ± 0.30	1.20 ± 0.35	30.05 ± 5.51	0.589 ± 0.124	4.86 ± 1.04

PET image quantification of tissues including the tumor, liver, muscle, and kidneys. Tumor-to-muscle ratio is indicated by T:M Ratio. The table lists the average value ± the standard deviation (SD, *n* = 3). Source Data are provided as a Source Data File

[^18^F]FP-R_0_1-MG-F2 was evaluated in pre-clinical models with either human α_v_β_6_-positive (~3.5 %ID g^−1^) or α_v_β_6_-negative (~0.5 %ID g^−1^) tumors (Fig. 1e, Supplementary Figs. 5 and 6). Selective tracer uptake was demonstrated in the α_v_β_6_ overexpressing tumors (Table 3 and Supplementary Table 3). Additionally, due to the widely adopted use and ubiquitous availability of ^68^Ga for PET imaging, R_0_1-MG was also evaluated as [^68^Ga]NODAGA-R_0_1-MG (Fig. 1e, f). [^68^Ga]NODAGA-R_0_1-MG demonstrated comparable tumor and organ uptake with respect to the radiofluorinated version. At 1 h p.i., [^68^Ga]NODAGA-R_0_1-MG’s tumor uptake was 2.8 ± 0.3 %ID g^−1^. Liver, muscle and kidney uptake was 1.2 ± 0.4 %ID g^−1^, 0.6 ± 0.1 %ID g^−1^ and 30.4 ± 5.5 %ID g^−1^, respectively (Table 3).

### [^18^F]FP-R_0_1-MG-F2 in healthy volunteers (Stanford)

[^18^F]FP-R_0_1-MG-F2 (Supplementary Fig. 7) was administered intravenously to five healthy male and female adult volunteers (Supplementary Table 4). No adverse effects were reported. Oxygen levels, vital signs, and electrocardiography (ECG) readings were normal (Supplementary Tables 5 and 6).

**Fig. 2:**
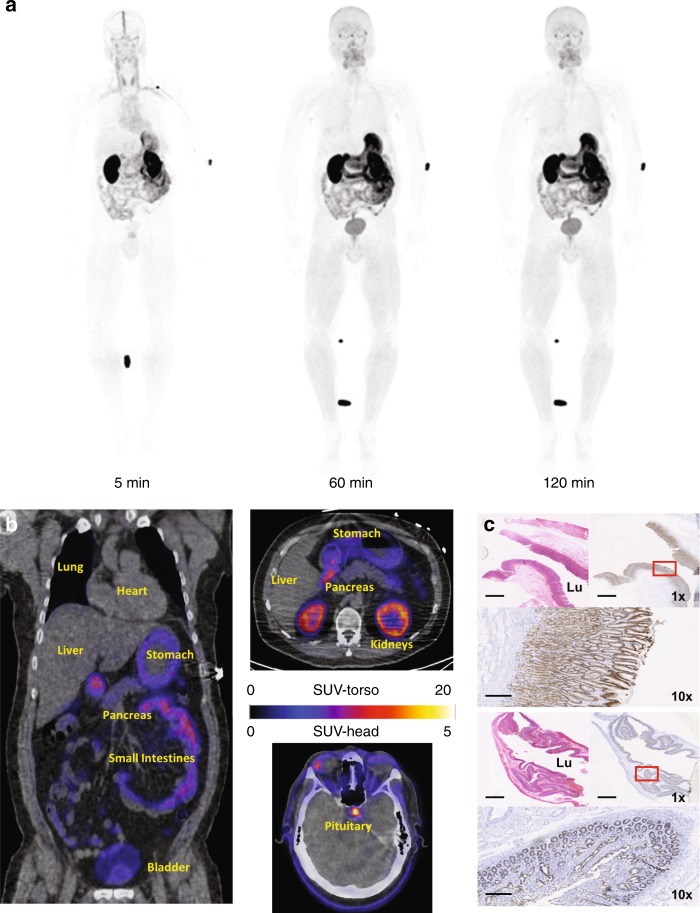
[^18^F]FP-R_0_1-MG-F2 PET imaging of five healthy human volunteers. **a** Representative whole-body [^18^F]FP-R_0_1-MG-F2 maximum intensity projection (MIP) PET images of a healthy volunteer (50-year-old male) show the biodistribution of PET tracer at ~ 5, 60, and 120 min post injection (p.i.). Focal uptake near the elbow and between the legs are the site of intravenous injection, and a tube containing the reference calibration standard, respectively. **b** Axial and coronal PET/CT images of the same healthy volunteer at 1 h p.i. Close-ups of the chest and abdominal regions show the heart (SUV_mean_ ~ 0.9), liver (SUV_mean_ ~ 0.8), lung (SUV_mean_ ~ 1.3), pancreas (SUV_mean_ ~ 1.9), stomach (SUV_mean_ ~ 12.6), small intestines (SUV_mean_ ~ 7.8), kidneys (SUV_mean_ ~ 14.3), and bladder (5.9). Accumulation of the tracer is evident in the pituitary gland (SUV_mean_ ~ 5). **c** H&E staining (top left) and integrin α_v_β_6_ (top right and bottom) immunohistochemical analysis of healthy stomach and small bowel tissue, where uptake was relatively high (Supplementary Table 7, below), shows expression of integrin α_v_β_6_ on the luminal (Lu) side of these organs. Scale bars on the 1x and 10x images represent 2.5 cm and 250 mm, respectively

Maximum intensity projection (MIP) PET shows the biodistribution of [^18^F]FP-R_0_1-MG-F2 in a healthy volunteer (HV-1, Fig. 2a and Supplementary Movies 1–3). Transaxial PET/CT images show tracer distribution in the abdomen and brain (Fig. 2b). [^18^F]FP-R_0_1-MG-F2 was renally cleared (SUV_mean_ ~ 15) with uptake noted in the stomach (SUV_mean_ ~ 10) and small intestines (SUV_mean_ ~ 9, Supplementary Table 7). IHC confirmed expression of α_v_β_6_ in normal stomach and small intestine tissues (Fig. 2c). [^18^F]FP-R_0_1-MG-F2 reached peak levels in blood within ~1–3 min after administration (Supplementary Table 8). Uptake in most normal organs including the lung and liver were relatively low (SUV_mean_ < 1, Fig. 2c). Muscle SUV_mean_ ~ 1.5 and pancreatic uptake was slightly higher at 1 h p.i. (SUV_mean_ ~ 2). Tracer accumulation was elevated in the pituitary gland (SUV_mean_ ~ 4, 1 h p.i.) and the large intestines (SUV_mean_ ~ 3, 1 h p.i.). The kidneys were the dose-limiting organ (SUV_mean_ ~ 12, 1 h p.i., Supplementary Tables 7 and 9).

### [^18^F]FP-R_0_1-MG-F2 vs. [^18^F]FDG in pancreatic CA (Stanford)

**Fig. 3:**
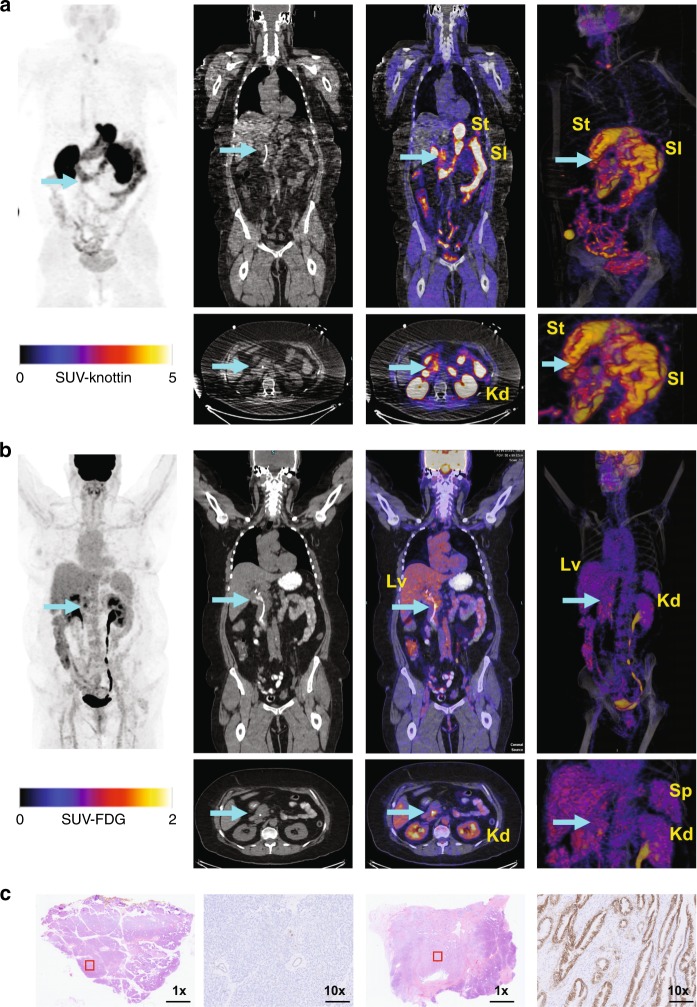
[^18^F]FP-R_0_1-MG-F2 PET imaging of pancreatic cancer. **a** [^18^F]FP- R_0_1-MG-F2 and (**b**) [^18^F]FDG images, from left to right, show MIP, CT, PET/CT (axial and coronal) and volume rendered PET/CT (full and magnified) images of a 71-year-old female pancreatic cancer patient at ~60 min post injection. The liver, kidneys, small intestines, stomach and spleen are denoted by Lv, Kd, SI, St, and Sp, respectively. The cyan arrow points to the tumor. **a** Accumulation of [^18^F]FP- R_0_1-MG-F2 at the head of the tumor is shown by the cyan arrow (SUV_mean_ = 6.3). PET/CT images demonstrate several regions of relatively high accumulation including the kidneys, the main clearance route, and the stomach (St), small intestines (SI) where integrin α_v_β_6_ is expressed. **b** An area of focal [^18^F]FDG uptake (SUV_mean_ = 2.3) which is located within the tumor coincides with a biliary stent (white tube) that is apparent in the CT image. The tracer is seen draining out of the kidney (Kd) through the ureter and collecting in the bladder in the MIP, and volume rendered PET/CT images. **c** From left to right, H&E staining and IHC analysis of the resected pancreatic mass, which included some healthy pancreatic tissue. A section of normal pancreas (left) and a section of malignant tissue (right) are shown. Scale bars on the 1x and 10x images represent 2.5 cm and 250 mm, respectively

[^18^F]FDG and [^18^F]FP-R_0_1-MG-F2 were separately administered on different days to a 61-year-old (y.o.) woman with pancreatic cancer (Supplementary Table 10). MIP PET, PET/CT and volume rendered PET/CT images show the biodistribution of [^18^F]FP-R_0_1-MG-F2 (Fig. 3a) and [^18^F]FDG (Fig. 3b) ~1 h p.i. Dynamic PET of the abdominal region are quantified in Supplementary Fig. 8. The biodistribution of [^18^F]FDG and [^18^F]FP-R_0_1-MG-F2 at 1 h p.i. are compared in Supplementary Fig. 9.

[^18^F]FP-R_0_1-MG-F2 was distributed broadly throughout the tumor (ROI ~ 8000 mm^3^) and resulted in a SUV_mean_ of 6.2 at 1 h p.i. (Fig. 3a, Supplementary Movie 4). Comparatively, a region of focal [^18^F]FDG uptake (ROI ~ 3000 mm^3^, SUV_mean_ = 4.1) was present adjacent to a biliary stent (Fig. 3b, Supplementary Movie 5). Tumor uptake is also shown in the volume rendered PET/CT images (Fig. 3a, b, right panels), where the cyan arrows point to the tumor mass. Uptake of [^18^F]FP-R_0_1-MG-F2 and [^18^F]FDG in the liver exhibited SUV_mean_s of 0.9 and 2.9, respectively, at 1 h p.i. SUV_mean_s of [^18^F]FP-R_0_1-MG-F2 in the stomach and small intestines were 22.9 and 10.7, respectively, at 1 h p.i. In comparison, SUV_mean_s of [^18^F]FDG in the stomach and small intestine at 1 h p.i. were 0.16 and 2.5, respectively. IHC analysis of resected tumor tissue confirmed high levels of α_v_β_6_ (Fig. 3c).

### [^68^Ga]NODAGA-R_0_1-MG in cancer patients (PUMC)

**Fig. 4:**
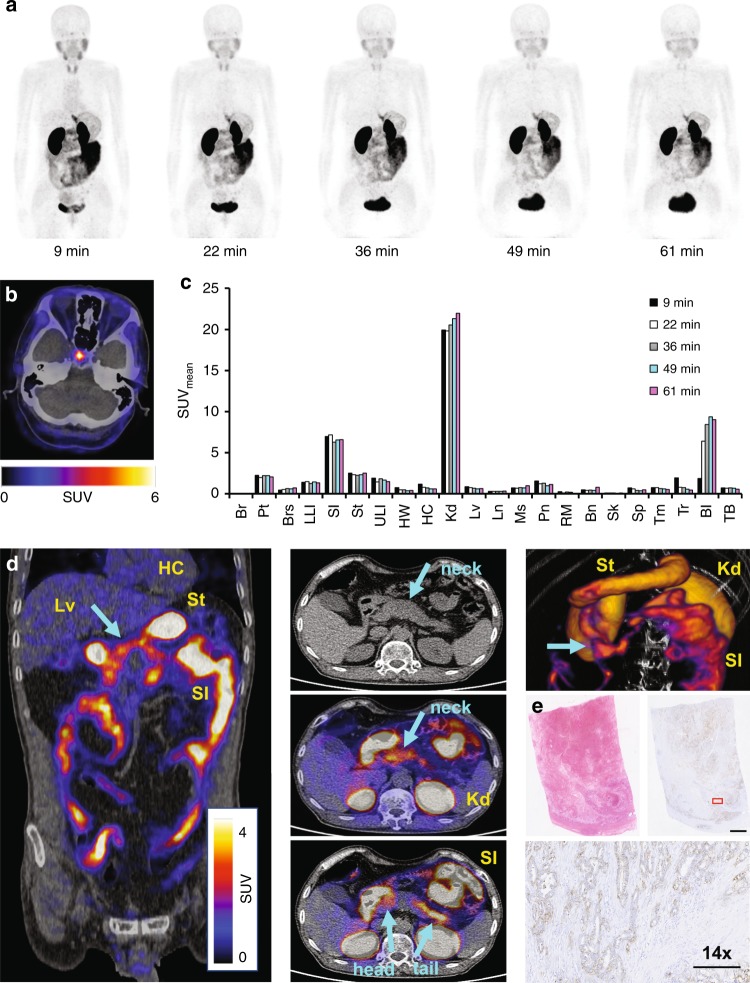
[^68^Ga]NODAGA-R_0_1-MG biodistribution and PET imaging of pancreatic cancer. **a** Sequential MIP PET images of a 69-year-old female study subject show the biodistribution of the tracer for up to ~1 h after intravenous administration. **b** PET/CT image of the brain shows focal uptake of the tracer in the pituitary gland. **c** ROI analysis of the representative patient shown above. The following organs are shown: brain (Br), pituitary gland (Pt), breast (Brs), lower large intestine (LLI), small intestine (SI), stomach (St), upper large intestine (ULI), heart wall (HW), heart contents (HC), kidney (Kd), liver (Lv), lung (Ln), muscle (Ms), red marrow (RM), osteogenic cells (Bn), skin (Sk), spleen (Sp), thymus (Tm), thyroid (Tr), bladder (Bl), and total body (TB). **d** Coronal and axial PET/CT images (left and center) and volume rendered PET/CT image (top right) acquired from a patient diagnosed with pancreatic cancer. The arrows indicate the location of the tumor. High uptake is observed throughout most of the pancreas including the head (SUV_mean_ ~ 3.1), uncinate process, neck and tail (SUV_mean_ ~ 4.4). Comparatively, the SUV_mean_ = 1.8 ± 0.5 S.D. for normal pancreas (*n* = 5). **e** IHC confirms integrin α_v_β_6_ expression in the viable part of the tumor. Red box represents area shown at 14x magnification in image below. Pathology report indicated a large amount of necrosis in the tumor. Scale bars on the unmagnified and 14x images represent 2.5cm and 250 mm, respectively. Source data for panel **c** are provided in a Source Data file

**Fig. 5:**
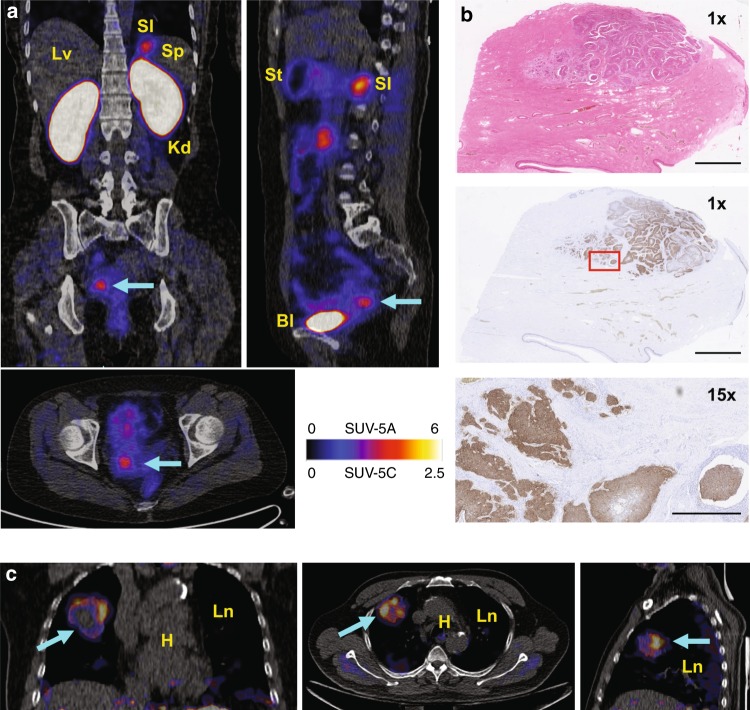
[^68^Ga]NODAGA-R_0_1-MG PET imaging of cervical cancer and lung cancer. **a** Coronal (top left), sagittal (top right) and axial (bottom left) planes of PET/CT images of a 47-year-old cervical cancer patient shows tracer accumulation in the tumor (cyan arrow). Bl, Kd, Lv, SI, Sp, and St refer to the bladder, kidneys, liver, small intestine, spleen and stomach, respectively. **b** H&E staining and IHC analysis confirm expression of integrin α_v_β_6_ by the larger diffuse cancer cells. Integrin α_v_β_6_ was not expressed by the normal cells of the cervix surrounding the tumor. Red box represents area shown at 15x magnification in image below. Scale bars on the 1x and 15x images represent 2.5 cm and 250 mm, respectively. **c** Coronal (left), axial (middle) and sagittal (right) planes of PET/CT Images of a 70-year-old patient diagnosed with poorly differentiated squamous cell carcinoma of the lung (SUV_mean_ = 2.2). The cyan arrow points to the tumor. The healthy lung and heart are indicated by Ln and H, respectively

[^68^Ga]NODAGA-R_0_1-MG was administered to two adult male patients (lung and pancreatic cancer) and four adult female patients (three cervical and one pancreatic cancer) who were diagnosed with cancer, as confirmed by tissue biopsy (Figs. 4 and 5, Supplementary Table 11). Sequential MIP PET images of a representative study subject acquired from 9 to 61 min after intravenous tracer administration (Fig. 4a) show comparable pharmacokinetics and biodistribution between [^68^Ga]-R_0_1-MG and the radiofluorinated version, [^18^F]FP-R_0_1-MG-F2. Similarly, the ^68^Ga-labeled knottin PET tracer also accumulated in the pituitary gland (Fig. 4b). ROI analysis of PET/CT images (Figs. 4c, d, Fig. 5a, Supplementary Figs. 10 and 11) show that [^68^Ga]-R_0_1-MG rapidly cleared via the kidneys, which was the dose-limiting organ (Supplementary Table 12). The average kidney SUV_mean_ was 19.92 ± 2.23 at ~1 h p.i. Notable uptake and retention of the tracer occurred in the stomach and small intestines where the average SUV_mean_s were 8.87 ± 6.52 and 7.06 ± 2.74, respectively. The SUV_mean_ of [^68^Ga]-R_0_1-MG in normal portions of pancreas was 1.79 ± 0.47 (*n* = 5). The average uptake in pancreatic cancer was SUV_mean_ = 4.2 ± 0.1 (Fig. 4 and Supplementary Fig. 12, Supplementary Movies 6 and 7). In one pancreatic cancer patient, a continuous band of tracer uptake occurred in a portion of the pancreas that spanned the head, uncinate process, neck, and tail (Fig. 4d). Here, the majority of the tumor mass occurred in the neck of the pancreas, but tracer uptake was seen in only a portion of that enlarged mass. The pathology report indicated moderately to poorly differentiated pancreatic adenocarcinoma, as well as a significant amount of necrosis in that tissue. H&E staining and IHC confirmed the expression of α_v_β_6_ in the viable parts of the tumor (Fig. 4e).

In cervical cancer patients, relatively low tracer uptake in the lower large intestines (SUV_mean_ = 1.54 ± 0.28) allowed for straightforward detection of cervical tumor where the average SUV_mean_ = 4.79 ± 0.37 (Fig. 5, Supplementary Fig. 13, Supplementary Movies 8 and 9). Comparatively, [^18^F]FDG accumulated to similar levels (SUV_mean_ = 4.1) in the tumor of the second cervical cancer patient (Supplementary Fig. 13). H&E staining and IHC confirmed the expression of α_v_β_6_ in the larger diffuse cancer cells shown in the tissue slice (Fig. 5b). Additionally, in one patient with lung cancer, very low uptake in the normal lung, SUV_mean_ = 0.41 ± 0.11, led to high contrast of a lung tumor (Fig. 5c, SUV_mean_ = 2.20) with a ~5:1 ratio of lung tumor to normal lung.

### [^18^F]FP-R_0_1-MG-F2 in IPF and healthy lungs (Stanford)

**Fig. 6:**
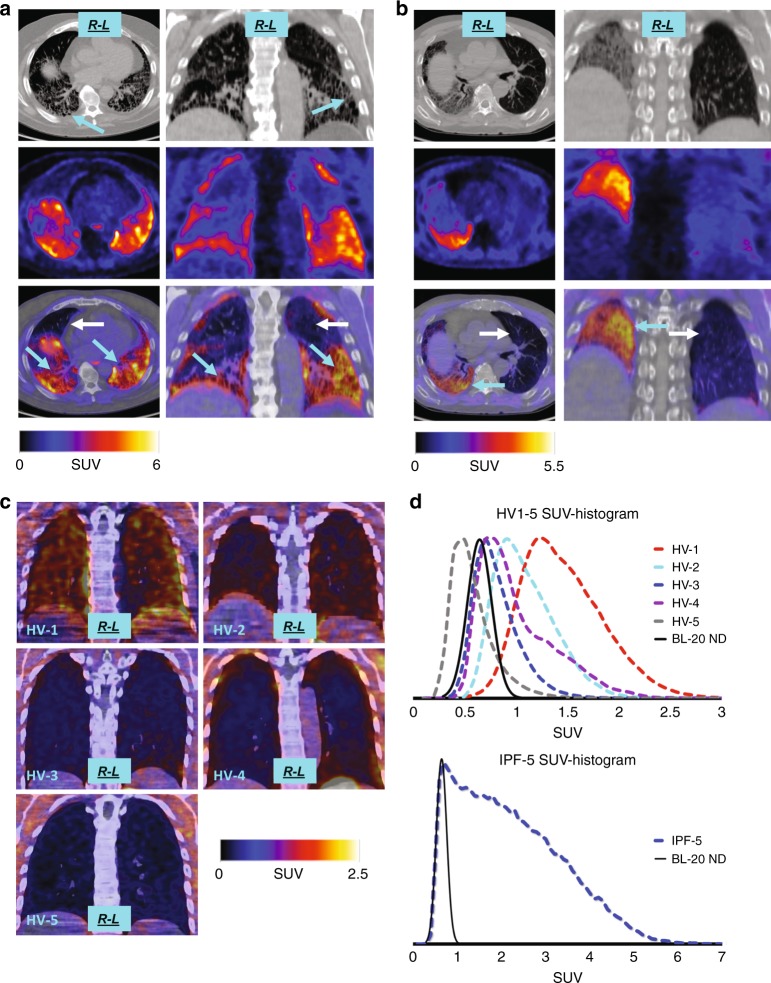
[^18^F]FP-R_0_1-MG-F2 PET imaging of lungs: IPF patients and healthy volunteers. **a** PET/CT images of IPF-5, a 72-year-old male IPF patient with a definite-UIP pattern, as indicated by peripheral, basilar predominant reticulation and honeycombing (CT, top, cyan arrows). Correspondingly, the PET tracer is concentrated in regions of greatest fibrosis in the areas of high reticulation and honeycombing in the lung bases (PET, middle and PET/CT, bottom, cyan arrows). In contrast, relatively healthy regions of the upper and anterior lungs show low tracer accumulation (PET/CT, bottom, white arrows). **b** PET/CT images of a 72-year-old male IPF patient with a single transplanted (2016) left lung (white arrows), which is relatively devoid of the PET tracer. The fibrotic right lung (IPF) shows elevated PET tracer levels in areas that also correspond to the highly fibrosed regions demonstrated on CT (PET/CT, bottom, cyan arrows). **c** PET/CT images of the lungs of five healthy volunteers, varying in age from 20- to 48-year old. The group demonstrated a range of lower [^18^F]FP- R_0_1-MG-F2 accumulation in their lungs. **d** The dashed lines represent histograms of SUVs found within ROI contours of total lung. The upper histogram represents the healthy volunteers (HV-1 to HV-5). The lower histogram (dashed blue line) represents patient IPF-5 shown in panel A. For comparative purposes, the solid black curves in both (top and bottom graphs) represent baseline-20 SUV_mean_ and normal distribution (BL-20 ND) derived from the two youngest healthy volunteers in their 20 s (HV-3 and HV-5)

One female and five male study subjects, 72.5 ± 1 y.o., who were diagnosed with IPF by chest CT demonstrating a definite-UIP pattern or lung tissue biopsy with a histopathologic UIP pattern, were administered 5–10 mCi of [^18^F]FP-R_0_1-MG-F2. Combined PET/CT-based whole-lung contours were used to quantify PET-tracer distribution in each lung. The left and right halves were treated independently in the following analysis. Immediately upon injection, a 15-min dynamic PET scans showed that the tracer rapidly entered and cleared from the healthy portions of the lungs (dark areas on CT), but accumulated and persisted in the diseased portions of those same lungs (white or gray on CT, Supplementary Movies 10–13). [^18^F]FP-R_0_1-MG-F2 accumulation was greatest in the most severely fibrosed portions of the lung or in those regions of the lung that clearly demonstrated the UIP pattern by CT (Fig. 6a, b, magenta arrows, reticulation, honeycombing, and basal/subpleural predominance).

**Fig. 7:**
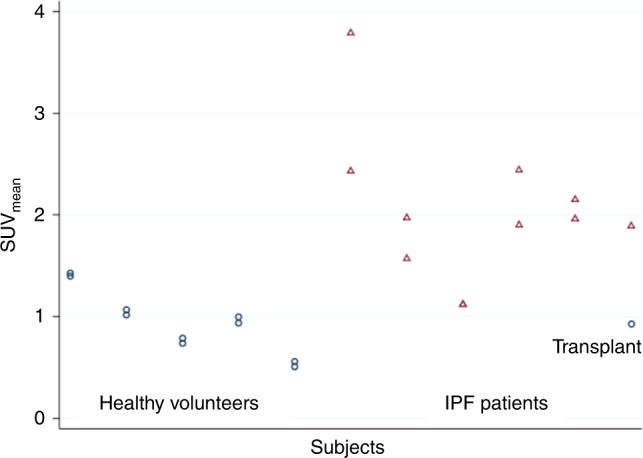
Mean SUVs of healthy volunteers and IPF patients. Mean SUVs from healthy volunteers and IPF patients are shown as circles and triangles, respectively. The single transplanted lung in patient IPF-4 is shown as a circle (far right). The right/left mean SUVs for patient IPF-3 were identical and appear as a single triangle (Supplemental Table 13). The right/left correlation is 0.90 and 0.70 for HVs and IPF patients, respectively. The average SUV_mean_s ± S.D.s for the right lungs of HVs (*n* = 5) and the IPF group (*n* = 6) is 0.92 ± 0.33 and 2.04 ± 0.91, respectively (*p* = 0.0087). Source data are referred to in the Source Data File

IPF-5 is a 72y.o. male IPF patient who was diagnosed by CT with a definite-UIP pattern in 2015 (Fig. 6a). The highest levels of [^18^F]FP-R_0_1-MG-F2 were found in the basal and subpleural regions of the lungs, consistent with the CT-UIP diagnosis as shown by the high degree of reticulation and honeycombing (magenta and cyan arrows). In contrast, the healthy portions of IPF-5’s lungs were relatively devoid of the PET tracer (white arrows). The SUV_mean_s for the entire left and right lungs were 2.44 ± 1.16 and 1.90 ± 1.11, respectively (Fig. 7, Supplementary Table 13). Due to the heterogenous distribution of fibrosis in ILDs, the extent of disease may be quantified by the SUV_range_ (SUV_max_ − SUV_min_, 6.82 ± 0.14 S.D.) as well as the fractional amount of damaged lung tissue (Supplementary Fig. 14B). While the SUV_max_s for the left and right lungs were 6.93 and 7.07, the lung regions most severely affected by the disease are comprehensively described by the distribution of SUVs (SUV histogram) in the upper part of the SUV_range_ (Fig. 6d). For the left lung, ~18% of the SUVs were between 3 and 3.9, while ~10% of the SUVs were >4 as shown in the PET tracers SUV histogram (Supplementary Fig. 14B) and a summary of SUV metrics in the knottin PET tracer’s fibrosis spectrum (Supplementary Table 13). For the right lung, ~13% of the SUVs were between 3 and 3.9, while ~5% were >4.

IPF-4 is a 72 y.o. male IPF patient who received a left lung transplant at Stanford Hospital in 2016. (Fig. 6b). Chest CT shows extensive fibrosis in the native right lung where [^18^F]FP-R_0_1-MG-F2 accumulation was high and sustained (Fig. 6b). Accumulation of the PET tracer correlated with the CT-based UIP pattern of fibrosis in the right lung, where the SUV_mean_ and SUV_range_ were 1.89 ± 0.74 and 5.10, respectively. In contrast, the SUV_mean_ and SUV_range_ of the transplanted left lung were 0.93 ± 0.38 and 4.34, respectively (Fig. 7, Supplementary Fig. 14A, and Supplementary Table 13). IPF-4’s transplanted left lung is relatively devoid of the PET tracer, and the CT images are also consistent with a typical healthy lung (Fig. 6b).

In order to calibrate the IPF lungs to a reference standard, the lungs of the five healthy volunteers were similarly quantified. The within-subject intraclass correlation between lungs in healthy volunteers was 0.99 compared to 0.76 in IPF patients^34^. Due to the high degree of correlation between right and left lungs of healthy volunteers and IPF patients, their right lungs were compared to avoid the left lung transplant of IPF-4. A statistically significant difference (*p* = 0.0087) in PET-tracer level was found between the set of healthy right lungs (average SUV_mean_ = 0.92 ± 0.33, *n* = 5) versus the diseased right lungs of IPF patients (average SUV_mean_ = 2.04 ± 0.91, Fig. 7, *n* = 6). The lowest [^18^F]FP-R_0_1-MG-F2 levels occurred in the two youngest volunteers, 20 (HV-3) and 26-year old (HV-5), where their total-lung SUV_mean_s = 0.76 ± 0.22 and 0.53 ± 0.20, respectively. For comparative purposes, the relatively pristine young lungs in these two individuals served as an age-based SUV referential-baseline (baseline-20 SUV_mean_ = 0.67 ± 0.12, and normal distribution) for comparison of all lung data reported in this study (Fig. 6c–d, Supplementary Table 13). The SUV-histograms of young lungs exhibited a Gaussian distribution (Fig. 6d) as modeled by baseline-20’s normal distribution (Fig. 6d). In contrast, the highest [^18^F]FP-R_0_1-MG-F2 levels (SUV_mean_ = 1.41 ± 0.38) were found in HV-1, a 48-year-old currently asymptomatic male, who is a long-term bird-owner. Exposure to birds is a well-studied risk-factors for fibrotic ILDs^35,36^. The two remaining volunteers, both females in their 40s, showed mildly higher levels of [^18^F]FP-R_0_1-MG-F2 relative to baseline-20 (Fig. 6d, top, Supplementary Table 13).

## Discussion

Our manuscript describes the complete process of developing and translating a cystine knot PET tracer for detection of multiple α_v_β_6_-positive indications, including cancer and IPF. In both pre-clinical small animal models and human clinical trial participants, accumulation of the knottin PET tracers with α_v_β_6_-positive tumors and the fibrotic portions of lungs was rapid, high, and sustained, compared to healthy tissues. Human cancer was detected in multiple regions of the body including the thorax (lung cancer), upper abdomen (pancreatic cancer), and lower abdomen (cervical cancer). Compared to very low background levels of this tracer in healthy young lungs, elevated tracer levels and a robust dynamic range of uptake were observed in the lungs of patients diagnosed with IPF. Accumulation of our PET tracers in most normal human tissues was generally low with notable exceptions in the upper gut and pituitary gland. Our initial findings suggest that the α_v_β_6_ knottin PET tracers may find broad clinical application in the detection of diseases marked by overexpression of α_v_β_6_.

α_v_β_6_ belongs to a subfamily of integrins called the RGD integrins^3,37^. Proteins or peptides that contain RGD motif are able to discriminate between different integrins by structural differences in the amino acids that flank the core RGD motif. The foot and mouth disease virus (FMDV) evolved an RGD flanking sequence in a viral coat protein that selectively recognizes α_v_β_6_ with high-affinity, while molecularly ignoring the other integrins such as α_v_β_3_. Sutcliffe et al. have extensive experience developing and translating different FMDV-based PET tracers^38^. Kraft et al. and Nothelfer et al. utilized phage display to develop affinity ligands that recognize α_v_β_6_^39,40^. Recently, a simple disulfide-stabilized peptide demonstrated modest uptake in head and neck cancer^27^.

Cystine knot peptides engineered for selective, high-affinity molecular recognition of cancer markers have recently shown promise in pre-clinical models, not only as PET tracers, but in near infrared fluorescence imaging, molecularly targeted ultrasound imaging, and photoacoustic imaging^4,41,42^. Studies of knottin PET tracers have revealed that their pharmacokinetics are highly tunable through simple amino acid substitutions in their loops^26^. This pharmacokinetic versatility is one characteristic that distinguishes cystine knot PET tracers from other peptide-tracers such as the linear FMDV derivatives, simple cyclic peptides (cyclo-RGD) or peptides that depend on their framework amino acids (non-binding) for structural stability (triple helix bundle). Comparatively, these simple linear or cyclic peptides have shown improved pharmacokinetic profiles through chemical modification strategies such as dimerization and/or PEGylation^38,43^.

Three different imaging labels ([^64^Cu]DOTA-, [^68^Ga]NODAGA-, and [^18^F]FP-) were used to evaluate the preliminary set of high-affinity knottins. Due to the relatively long half-life of ^64^Cu (12.7 h), [^64^Cu]DOTA- is well suited for initial screening of ligands beyond 2 h so that we may survey tracer uptake in tumor and normal tissues for up to 24 h after injection in pre-clinical models. In this case, once we determined that there was no benefit to imaging beyond a certain timepoint, shorter-lived radioisotopes with higher positron yields and lower overall radiation exposure were used. The equipment (e.g. cyclotron) and expertize at Stanford radiochemistry facilitates the development of [^18^F]FP-R_0_1-MG-F2 and over a dozen clinical-grade ^18^F labeled PET tracers for routine clinical use. However, the [^68^Ga]NODAGA-R_0_1-MG version evaluated in this study by PUMC hospital may potentially be better suited for deployment due to the ubiquitous availability of ^68^Ga (not requiring a dedicated cyclotron), and the ease of radiosynthesis through a pre-prepared kit. For these reasons, both the ^18^F and ^68^Ga versions were studied.

R_0_1-MG PET tracers were evaluated in several different α_v_β_6_-positive cancers. In pancreatic cancer, accumulation of the PET tracer, [^18^F]FP-R_0_1-MG-F2 was rapid and remained high throughout the study (SUV_mean_ ~ 6 at 1 h p.i.). Here, we were fortunate to have [^18^F]FDG data from a clinical PET/CT for comparison. Accumulation of [^18^F]FDG by the tumor was concentrated (~3000 mm^3^, SUV_mean_ ~ 4 at 1 h p.i.) near a biliary stent which was implanted to mitigate tumor-compression of the common bile duct. Comparatively in this same patient, the uptake of [^18^F]FP-R_0_1-MG was more uniformly distributed over a greater tumor volume (~8000 mm^3^). The difference in uptake profiles between the two classes of PET tracers probably reflects the different activities that are targeted (glucose metabolism vs. expression of ECM protein). In another pancreatic cancer patient, we observed a continuous band of intense uptake of [^68^Ga]NODAGA-R_0_1-MG from the head of the pancreas (SUV_mean_ ~ 3.8) to the tail (SUV_mean_ ~ 7), while the main tumor mass was located at the neck of the pancreas. Interestingly, the uptake did not occur throughout this entire tumor mass; tracer accumulation occurred only within the craniad of the enlarged mass as part of that continuous band of head-to-tail uptake. The pathology report indicated significant necrosis within the resected mass, and α_v_β_6-_positive IHC staining occurred only in the viable part of the tumor. Pancreatic tumors in three patients were easily discernable from their PET/CT images despite high tracer accumulation in neighboring stomach and small intestine.

Accumulation of the knottin PET tracer was observed in the primary tumors of cervical cancer patients (SUV_mean_ ~ 5) where uptake by neighboring lower bowel and uterus were relatively low. Due to the spatial resolution of the clinical PET scans, cervical cancers were clearly delineated from adjacent bladder containing urine-excreted knottin PET tracer. However, one caveat comes from an early report that found a 10-fold higher expression of β6 integrin subunit’s mRNA in the stratum functionalis of endometrial epithelium^44^. Much lower mRNA levels were reported for the analogous specimen during the proliferative phase endometrium, suggesting that α_v_β_6_ levels may be in flux during the menstrual cycle. Finally, very low α_v_β_6_ knottin PET tracers accumulation in the upper thorax of normal subjects allowed the detection of lung cancer in one patient (Fig. 5c).

This pilot clinical study with the knottin PET tracers for cancer detection is limited mainly by the small number of cancer patients. Therefore, recruitment of additional cancer patients is ongoing as we aim to comprehensively evaluate these knottin PET tracers for the cancers described here along with serous ovarian cancer, and head and neck cancer, both of which demonstrate elevated α_v_β_6_ levels.

α_v_β_6_’s role in TGF-β1 activation/signaling and in the pathogenesis of fibrotic diseases originating in lung and liver has been well documented^45–48^. Inhibition of fibrosis through molecular recognition of α_v_β_6_ has been achieved in the bleomycin mouse model as well as in human clinical trials by a small molecule inhibitor and several monoclonal antibodies^28,49,50^.

[^18^F]FP-R_0_1-MG-F2 enables whole lung molecular assessment of lung fibrosis through expression of α_v_β_6_, an ILD histological marker that correlates with prognosis^2^. The knottin PET tracer’s fibrosis spectrum (Supplementary Table 13 provides information about the extent of disease, with IPF patients exhibiting right-ward shift of the SUV_mean_, extension of the SUV_range_, and high-SUV voxels populating the upper bins of the SUV_histogram_. The ability to non-invasively characterize active fibrosis in ILDs including IPF, based on molecular expression may be useful for treatment planning and monitoring.

A lower range of [^18^F]FP-R_0_1-MG-F2-accumulation was observed in the lungs of healthy volunteers, which suggests that this PET-tracer may be able to discern healthy individuals from IPF patients^51,52^. Data for the low (healthy) end of the knottin PET tracer’s fibrosis spectrum were obtained from the two youngest healthy volunteers (HV-3 and HV-5) in their twenties, who provided a hypothetical reference SUV baseline-20 mean and normal distribution for young lungs. Statistical departure (z-score) away from baseline-20 appears to correlate with the gradual transition from healthy lungs to fibrotic lungs in the case of IPF, as shown in the fibrosis spectrum (Supplementary Fig. 12). The mildly increased levels of PET-tracer accumulation in the lungs of the two healthy mid-40s female volunteers (HV-2 and HV-4, ~ 3*σ* from baseline-20 SUV_mean_) suggests that α_v_β_6_ levels increase with age. Interestingly, the high end of [^18^F]FP-R_0_1-MG-F2’s fibrosis spectrum from the healthy volunteers group (7*σ* from baseline-20 SUV_mean_) was observed in a 48-year-old asymptomatic male (HV-1), who is a long-term bird-owner with a history of chronic second-hand-smoke and wood-dust exposure^35,51^. Although his low-dose chest CT showed relatively normal lungs, [^18^F]FP-R_0_1-MG-F2 levels in HV-1 (SUV_mean_ ~ 1.4) were higher and overlapped with IPF-3’s SUV metrics in the lower range of the fibrosis spectrum (SUV_mean_ ~ 1.1, Supplementary Table 13).

The possibility that [^18^F]FP-R_0_1-MG-F2 can monitor dynamic changes in fibrosis was also suggested by patient IPF-4’s successfully transplanted left lung, where tracer uptake levels were similar to levels recorded in lungs from healthy volunteers (Fig. 7). α_v_β_6_ plays a key role in wound healing, and accumulation of the PET tracer is expected if the administration of [^18^F]FP-R_0_1-MG-F2 had occurred during active tissue remodeling^53^. Interestingly, [^18^F]FP-R_0_1-MG-F2 did not accumulate at the bronchial anastomosis site, suggesting the wound-healing process was complete 2-year post-transplant when the PET study was conducted.

The current pilot study for IPF was limited by the lack of histological data for patient-matched tissues. Although the expression of α_v_β_6_ in IPF has been immunohistologically confirmed in multiple previous studies, patient-matched IHC studies may provide additional insight into correlation between PET tracer uptake and severity of disease^2,28^. Finally, our study does not contain enough clinical data to determine statistical correlation to other metrics used in IPF such as pulmonary function tests.

The accumulation of the knottin PET tracers occurred in the pituitary gland of all study subjects (where the PET scan included the brain, *n* = 11); this was an unexpected finding in our study. Although accumulation of our tracers did not occur in the other major regions of the normal human brain, we observed rapid and sustained uptake of our α_v_β_6_ probes in the pituitary region throughout the duration of the study (SUV_mean_ ~ 4 at 1 h p.i.). A review of the literature indicated that several integrins are expressed in the normal pituitary gland, and that the expression pattern of integrins expressed in the pituitary changes with the occurrence of adenomas, which represent about 10–15% of all intracranial neoplasms^54–56^. Most of the studies that surveyed integrin expression in the pituitary were conducted many years earlier with a limited repertoire of antibodies for IHC, and before most investigators ever looked for the expression of the β6 integrin subunit in the pituitary gland. Therefore, there are several reports that provide a list of integrin subunits that were investigated at that time except for the β6 subunit. We were not able to find any current studies which confirmed, either at the message or protein level, the expression the β6 subunit in the pituitary. Moreover, there are several conflicting reports about the expression of the α_v_ subunit in the healthy and diseased pituitary^54,57^. A parallel line of potentially related research has focused on the expression of TGF-β1 expression in the pituitary gland. Local TGF-β1 expression has been confirmed in both normal and cancerous pituitary tissues^55,58,59^. The reason that this is important for our studies is that expression of TGF-β1 is often associated with the expression of its activator, α_v_β_6_^29,60^. Accumulation of our probe may have provided evidence for the expression of α_v_β_6_ in the human pituitary. Although the anterior portion of pituitary gland remains outside of the blood brain barrier, our studies show that it is possible to target a 4 kDa peptide probe to the pituitary gland, which may allow the detection of adenomas or prolactinomas by developing probes that target cell surface proteins, such as other integrins that are specific for those diseases. Moreover, because of the sustained uptake of the knottin PET tracers in the pituitary, our studies suggest that it may be possible to exploit knottins, such as R_0_1-MG, for delivery of therapeutic activities to that region of the brain.

In conclusion, we have addressed several unmet clinical needs by developing cystine knot PET tracers that effectively detect multiple cancers in different regions of the body, as well as fibrotic changes in the lungs in IPF patients. In pancreatic, cervical and lung cancer, and in IPF, the knottin PET tracers demonstrated rapid and sustained accumulation in diseased tissues with relatively low background uptake in healthy organs or regions of the body prone to different cancers or fibrosis (lung and liver). These results suggest that the R_0_1-MG based PET tracers will have broad clinical application in detecting/diagnosing multiple indications, monitoring the efficacy of multiple therapeutics, as well as in staging both cancer and pulmonary fibrosis. The results from these pilot clinical studies encourage comprehensive evaluation of these α_v_β_6_ cystine knot PET tracer across a broad range of disease states and applications in different patient populations.

## Methods

### Materials, cell lines, and reagents

BxPC-3 human pancreatic cancer cells were obtained from American Type Culture Collection (ATCC) and grown in RPMI 1640 media (ATCC). A431 epidermoid cancer cells, human embryonic kidney 293T cells (293T) cells were obtained from frozen lab stocks and grown in DMEM supplemented with 10% FBS and penicillin/streptomycin (Invitrogen). Recombinant human integrins αvβ_6_ was purchased from R&D Systems. Yeast growth materials (SD-CAA, SG-CAA, SD-CAA agar plates, YPD) were purchased from Teknova. All other chemicals were obtained from Fisher Scientific unless otherwise specified. Integrin binding buffer (IBB) is composed of 25 mM Tris pH 7.4, 150 mM NaCl, 2 mM CaCl_2_, 1 mM MgCl_2_, 1 mM MnCl_2_, and 0.1% bovine serum albumin (BSA).

### Site directed evolution

The open reading frames encoding cystine knot peptides were generated by overlap-extension PCR using yeast-optimized codons defined by Lasergene (DNASTAR.com). The position that was randomized, as denoted by the letter x in Supplementary Fig. 1, was constructed with the NNB degenerate codon sequence. PCR products were amplified using primers with overlap to the pCT yeast display plasmid, which were upstream or downstream of the NheI and BamHI restriction sites, respectively. For each library, ~40 µg of DNA insert and 4 µg of linearized pCT vector were electroporated into the *S. cerevisiae* strain EBY100 by homologous recombination. Electroporation was performed using cuvettes with a 2 mm gap. The electroporator was set to exponential decay mode, 540mV and 25µf.

The library was incubated and screened at room temperature for 2 h in 1 nM of recombinant integrin α_v_β_6_ in IBB^31^. Next, a 1:250 dilution of chicken anti-cMyc IgY antibody (AB_2535826, Cat # A-21281, ThermoFisher) was added for 1 h at 4 °C. The cells were washed with ice-cold IBB and incubated with a 1:25 dilution of fluorescein-conjugated anti-human α_v_ integrin antibody (Clone NK1-M9, Cat # 327908, Biolegend) and a 1:100 dilution of Alexa 555-conjugated goat anti-chicken IgG secondary antibody (AB_2535858, Cat # A-21427, ThermoFisher) for 0.5 h at 4 °C. Cells were washed in IBB and α_v_β_6_ integrin binders were isolated using a Becton Dickinson FACS Aria III instrument. A diagonal sort gate was used to isolate yeast cells with enhanced integrin binding (FITC fluorescence) for a given protein expression level (Alexa 555 fluorescence). Plasmid DNA was recovered by Zymoprep (Zymo Research), amplified in Max Efficiency DH5α *E. coli* cells (Invitrogen) and sequenced.

### Determination of equilibrium dissociation constants

Various concentrations (100 nM to 300 pM) of recombinant integrin α_v_β_6_ were incubated with 10^5^ yeast cells expressing R_0_1, R_0_1-MG, R_0_1-MR, or R_0_1-MW in the presence of 10^6^ un-induced yeast cells. Prior to flow cytometry and analysis, yeast cells were processed, stained, and washed as described above in the section called Library Synthesis and Screening. Using two color flow cytometry, the binding of knottin (FITC) to integrin α_v_β_6_ was normalized to expression level (Alexa 555) prior to determination of equilibrium dissociation constants (*K*_D_). The normalized binding was plotted against the log of the concentration of recombinant integrin α_v_β_6_. The *K*_D_ was determined by nonlinear regression analysis using Kaleidagraph (Synergy Software).

### Determination of IC_50_s of knottin derivatives

Various concentrations of synthetic knottin peptide R_0_1-MG and its derivatives were incubated with 10 nM integrin α_v_β_6_ at room temperature overnight. In order to determine the half-maximal inhibitory concentration, the ligands, R_0_1-MG, DOTA-R_0_1-MG, NODOGA-R_0_1-MG, [^19^F]FP-R_0_1-MG-F1 and [^19^F]FP-R_0_1-MG-F2 were allowed to compete with 10^5^ induced yeast cells (in the presence of 10^6^ un-induced carrier yeast cells) surface-displaying R_0_1-MG for binding to recombinant integrin α_v_β_6_. A ten-fold molar excess of test ligand, relative to the moles of yeast surface displayed R_0_1-MG, was provided to the system (~50,000 R_0_1-MG/yeast cell).

### Peptide synthesis and folding

Precursor peptide R_0_1-MG was synthesized on a CS Bio CS336 instrument via 9-fluorenylmethoxycarbonyl (Fmoc)-based solid phase peptide synthesis methods, and a Rink amide resin (CS Bio). Fmoc-protected amino acids were purchased from CS Bio. Fmoc groups were removed with 20% piperidine in DMF. Amino-acid coupling was performed using HOBt/diisopropylcarbodiimide (DIC) chemistry in DMF. Side-chain deprotection and resin cleavage was achieved by addition of a 94:2.5:2.5:1 (vv^−1^) mixture of trifluoroacetic acid (TFA)/trimethylsilane/ethanedithiol/water for 2 h at room temperature. The crude peptides were precipitated with cold, anhydrous diethyl ether and purified to >95% purity by semi-preparative reversed-phase HPLC using a Dionex Ultimate 3000 HPLC system and a Vydac 218TP1010 C18 column. Linear gradients of 90% acetonitrile in water containing 0.1% (v v^−1^) TFA were used for all peptide purifications, which were monitored at an absorbance of 220 nm. Peptide purity was analyzed by analytical reversed-phase HPLC using either a 214TP C4 5μ or Phenomenex Aeris C_18_ column. Molecular masses were confirmed by matrix-assisted laser desorption/ionization-mass spectrometry (MALDI-MS; ABI 5800, Supplementary Table 1).

Precursor peptide folding reactions were performed by gently rocking peptides for 12–20 h in a 0.1 M ammonium bicarbonate, pH9 solution containing 2.5 mM reduced glutathione, 20% dimethlysulfoxide (v v^−1^), 20% isopropanol (vv^−1^), and 20% 0.8 M guanidinium hydrochloride. The final oxidized (folded) precursor product was purified by semi-preparative reversed-phase HPLC as described above. Following purification, folded peptides were lyophilized and stored at room temperature until used. Purified peptides were dissolved in water, and concentrations were determined by amino acid analysis (Jay Gambee AAA Service Laboratory, Damascus, OR). Peptide purity and molecular mass were confirmed by analytical reversed-phase HPLC and MADLI-MS. The CGMP precursor peptide R_0_1-MG was made, using a similar protocol, by CS Bio (Menlo Park CA).

### DOTA or NODAGA conjugation

Briefly, 1–2 mg of peptides were conjugated to ~2 mg DOTA-NHS (Macrocyclics, Plano TX) or NODAGA-NHS (Chematech, Dijon France) in 500 μL DMF containing 2 μL DIPEA at room temperature for up to 1 h. Chelator-peptide conjugates were acidified in solvent A purified by semi-preparative reverse phase HPLC as described above.

### ^64^Cu radiolabeling

Approximately 10 μg of peptide were combined with ~2 mCi ^64^CuCl_2_ in ~500 μL 100 mM sodium acetate buffer pH5.5 at 37 °C with gentle shaking at 250 rpm for at least 1 h prior to purification by PD-10 column. Twelve 500 μL fractions were collected from the column. The active fraction (typically 6 and 7) was subsequently used for in vivo studies.

### ^68^Ga radiolabeling

^68^GaCl_3_ was eluted from a ^68^Ge/^68^Ga generator with 0.1 N HCl. [^68^Ga]NODAGA-R_0_1-MG was prepared by adding ~2 mCi (~75 MBq) ^68^GaCl_3_ to ~10–20 μg NODAGA-R_0_1-MG in 500 μl 0.1 N NH_4_OAc (pH 4.5). The mixture was incubated in a shaker at 250 rpm for 15 min at 40 °C. The mixture was purified by PD-10 column as described above in ^64^Cu radiolabeling.

### Radiosynthesis and purification of [^18^F]FP-R_0_1-MG-F2

The radiosynthesis of [^18^F]-4-nitrohenyl-2-fluoropropanoate ester ([^18^F]NPE) was accomplished via nucleophilic ^18^F fluorination of methyl 2-bromopropionate, hydrolysis, and esterification in an automated radiosynthesizer (TRACERlab FX N PRO; GE Healthcare). Subsequently, [^18^F]NPE was purified on a reverse phase HPLC column (Luna C18 250 × 10 mm, 5 μm, Phenomenex) with gradient conditions (A: H_2_O + 0.1% TFA, B: CH_3_CN + 0.1% TFA; 0-2 min 5% B, 2-32 min 5–65% B, 5.0 mL min^−1^). The conjugation between pure [^18^F]NPE and the cystine knot peptide, R_0_1-MG was performed in a semi-automated customized radiosynthesis module to yield [^18^F]FP-R_0_1-MG. The crude product was purified on a reverse phase HPLC column (Gemini C18 250 × 10 mm, 5 μm, Phenomenex) with gradient conditions (A: H_2_O + 0.1% TFA, B: CH_3_CN + 0.1% TFA; 0–2 min 10% B, 2–10 min 10–20% B, 10–20 min 20–23% B, 20–65 min 23–30% B, 5.0 mL min^−1^). As described above, both fractions of [^18^F]FP-R_0_1-MG, F1 and F2, were present. However, it was hard to achieve pure [^18^F]FP-R_0_1-MG-F1 since it was coeluted with a radioactive impurity. Therefore, it was excluded from further development. At the same time, [^18^F]FP-R_0_1-MG-F2 was consistently obtained >90% purity.

### Tumor models

We have complied with all relevant ethical regulations for animal testing and research. Animal procedures were performed with approval from the Stanford University Administrative Panels on Laboratory Animal Care (APLAC, protocol #9536). Female athymic nude mice, 4–6 weeks old (Charles River), were subcutaneously shoulder-injected with 10^7^ cells suspended in 100 µL PBS. Mice were used for imaging and biodistribution studies when xenografted tumors grew to a diameter of ~1 cm.

### Static small animal PET imaging

Tumor-bearing *Nu/Nu* female mice (n = 3 for each probe) were anesthetized using 2% isoflurane in oxygen and injected with ~100 µCi (~0.15 nmol) of the tracers described above via the tail vein. Five-minute static PET scans were acquired on an Inveon PET-CT or Inveon D-PET scanner (Siemens Healthcare, Malvern PA). Images were reconstructed by a two-dimensional ordered expectation maximum subset algorithm and calibrated as described below. ROIs were drawn over the tumor on decay-corrected whole-body images using Inveon Research Workplace (IRW) software (Siemens) or ASIPro VM software (Siemens). ROIs were converted to counts g^−1^ min^−1^, and %ID g^−1^ values were determined assuming a tissue density of 1 g ml^−1^.

### Calibration of small animal PET

Scanner activity calibration was performed to map between PET image units and units of radioactivity concentration. A preweighed 50-mL centrifuge tube was filled with distilled water and ^64^CuCl_2_ (∼9.3 MBq [∼250 μCi] as determined by the dose calibrator) was used to simulate the whole body of the mouse. This tube was weighed, centered in the scanner aperture, and imaged for a 30-min static image, single bed position. From the sample weight and assuming a density of 1 g ml^−1^, the activity concentration in the bottle was calculated in units of μCi mL^−1^. Eight planes were acquired in the coronal section. A rectangular region of interest (ROI) (counts/pixel/s) was drawn on the middle of eight coronal planes. Using these data, a calibration factor (C) was obtained by dividing the known radioactivity in the cylinder (μCi mL^−1^) by the image ROI. This calibration factor was determined periodically and did not vary significantly with time.

### Dynamic small animal PET and PET/CT imaging

Dynamic scans were acquired over ~2 h p.i. Image acquisition was initiated 15 seconds prior to tracer injection and proceeded for 115.1 min p.i. The acquired data were then sorted into 0.5-mm sinogram bins and 26 time frames for image reconstruction (4 × 15 s, 1 × 37.5 s, 4 × 60 s, 11 × 300 s and 5 × 600 s), which was done by iterative reconstruction using the following parameters: 3D ordered-subsets expectation maximization (3D-OSEM) followed by fast maximum a posteriori (fastMAP); MAP OSEM iterations: 2; MAP subsets: 16; MAP iterations: 18. ROI analysis (IRW) was performed on the tumor and the major organs (heart, liver, kidneys, bladder, and muscle) seen on the dynamic PET scan images. The count densities were averaged for all volumes of interest at each timepoint to obtain a time versus radioactivity curve (TAC). Tumor TACs were normalized to injected dose, measured by a CRC-15 PET dose calibrator (Capintec, Inc.), and expressed as percentage injected dose per gram of tissue (%ID g^−1^), assuming 1 g ml^−1^.

### Pre-clinical radiation dosimetry

Non-decay-corrected uptake (%ID g^−1^) values from the dynamic PET study (above) were converted to %ID organ^−1^ and then subjected to an animal-to-human biokinetic extrapolation using the percent kg g^−1^ method where (%ID organ)human = [(%ID g^−1^)_animal_ × (kg_TBweight_)_animal_ × g_organ_ (kg_TBweight_)_human_^−1^]. The animal whole body weight was 34 g and the weights of the human organs were derived from a 73 kg male and 58 kg female in Organ level Internal Dose Assessment (OLINDA) software; source organ residence times were calculated using a bi-exponential model OLINDA^61^. The projected human doses were then computed for human phantoms using the source organ residence times.

### In vitro and in vivo stability

Aliquots of [^18^F]FP-R_0_1-MG-F2 were incubated in an equal volume of AB Human Serum (Invitrogen, 34005100) for 2 h at 37 °C. Samples were acidified with TFA and centrifuged at 18,000 g for 3 min to remove precipitants. For in vivo stability, mouse urine was collected with a syringe immediately after euthanasia. Typically, ~100 μL of urine was released by the bladder as it relaxed when the mice died; urine pooled at the genitals and was stabilized by surface tension. All samples were analyzed by radio-HPLC on a Dionex C_4_ column.

### Biodistribution analysis

Anesthetized nude mice bearing xenograft tumors were injected with ~100 µCi (~0.15 nmol) of the radio peptides described above via the tail vein, and euthanized after 1 h, 2 h, 4 h and or 24 h for the studies involving the [^64^Cu]DOTA-labeled peptides, and at 0.5 h and 2 h for studies involving [^18^F]FP- and [^68^Ga]-NODAGA labeled peptides. Tissues were removed, weighed and measured by scintillation counting^62^. Radiotracer uptake in tissues was reported as %ID g^−1^ and represents the mean ± standard deviation of experiments performed on three or more mice.

### Synthesis and characterization of [^19^F]FP-R01-MG-F2

In preparation for clinical development, the cold fluorine-19 lead compound, [^19^F]FP-R_0_1-MG, was chemically synthesized, and characterized by RP-HPLC, MALDI-MS, x-ray crystallography, and NMR spectroscopy.

The following describes part 1 of the [^19^F]FP-R_0_1-MG-F2 Synthesis, the synthesis of the [^19^F] Nitrophenyl Ester (NPE). 2-Fluoropropionic Acid (FPA) was mixed with 1 M NaOH in a 1:1 ratio and allowed to react at 95 °C for 10 min. The reaction mixture was transferred to a round bottom flask and two volumes of acetonitrile was added to the mixture for azeotropic distillation at 30 °C on a Rotary Evaporator. The sodium 2-fluoropropionate (FP^-^Na^+^) product appeared to be a white flaky powder with a molecular weight of 130.15 g n^−1^. Its mass and structure were verified by ESI-MS and NMR, respectively.

Next, sodium 2-fluoropropionate (solid) was mixed with 328 mM bis(4-nitrophenyl) carbonate (NPC) in acetonitrile in a 1:2 to 1:7 molar ratio. The round bottom flask was placed in 90–110 °C oil bath for 20–30 min. The resulting product, 2-fluoro nitrophenyl propanoate referred to as the nitrophenyl ester ([^19^F]NPE) compound was purified by semi-preparative RP-HPLC using a Phenomenex Gemini C18 column. A 90% acetonitrile, 0.1% trifluoroacetic acid (TFA, solvent B) gradient was used. The solution containing purified [^19^F]NPE was next diluted in a 1:1 (vv^−1^) ratio with solvent A (99.9% H2O, 0.1% TFA) and loaded onto a Waters Sepak C18 column, which was rinsed three times with solvent A. The purified product, [^19^F]NPE was eluted from the Sepak into a 20 mL glass storage bottle using 2 mL diethyl ether. The ether was evaporated on a hot plate (80–90%) in the presence of an air stream, which resulted in dry [^19^F]NPE as a white crystal film.

The following describes part 2 of the [^19^F]FP-R_0_1-MG-F2 Synthesis, coupling of Precursor R_0_1-MG to NPE. Both the precursor R_0_1-MG and NPE were resuspended in anhydrous DMF and mixed in a 1:1 molar ratio in the presence of 0.5% (v v^−1^) DIEA. Coupling of the fluoropropyl group to the precursor occurs on the sole amine functionality present on the N-terminus of R_0_1-MG and nowhere else on the peptide. The reaction was allowed to proceed for up to 1 h. The reaction was terminated by addition of 9 volumes of HPLC buffer A (99.9% H_2_0 and 0.1% TFA). RP-HPLC indicated two products, fraction 1 ([^19^F]FP-R_0_1-MG-F1) and fraction 2 ([^19^F]FP-R_0_1-MG-F2), separated by ~1 minute. Each product was separately purified to homogeneity by RP-HPLC. MALDI-MS and NMR revealed identical spectra and masses of 3906 Da for both [^19^F]FP-R_0_1-MG-F1 and [^19^F]FP-R_0_1-MG-F2 (Supplementary Table 1).

### NMR spectroscopy

2D ^1^H-^1^H NOESY and ^1^H-^1^H TOCSY NMR spectroscopy data were acquired on 1.0 mM R_0_1-MG and ^18^F-fluoropropyl-R_0_1-MG ([^18^F]FP-R_0_1-MG) cystine knot peptides in 20 mM potassium phosphate, pH 6.0 at 300 K^63,64,65^. After HPLC fractionization, peptide preparations were lyophilized. Aqueous peptide solutions with 5% D_2_O including 0.05 wt% 3-(trimethylsilyl)propionic-2,2,3,3-d_4_ acid (Sigma-Aldrich 450510) of R_0_1-MG and [^18^F]FP-R_0_1-MG were prepared, neutralized, and pH adjusted to pH 6.0 with 3 μl 1 N NaOH (1:10 diluted Amresco E584) to a final volume of 200 μl into 3 mm NMR tubes (C-S-3-HT-7, Norrell). 2D ^1^H-^1^H NMR spectroscopy data were recorded using phase-sensitive homonuclear 2D ^1^H-^1^H NOESY with presaturation and homonuclear 2D ^1^H-^1^H TOCSY with Hartman-Hahn transfer using MLEV17 spinlock sequence with presaturation at 128 scans, 512 indirect complex data point, d1 = 2.5 s, 1 H pulse 7.6μs, power level of shaped pulse 25.55db, mixing time d8 = 80 ms (in the case of NOESY also 120, 250, 300 ms to assess NOE spin diffusion)^66,67^ and an experimental time of about 24 h at 300 K at an Avance II 600-MHz spectrometer fitted with a cryogenic probe operating with TOPSPIN 3.0 (Bruker BioSpin GmbH). All spectra were Fourier transformed, phased, baseline corrected, and referenced to TSP (δ 0.00 ppm) using NMRPipe version X^68^. Solutions of R_0_1-MG before and after ^18^F-fluoropropyl derivatization showed similar dispersion of the amide finger print region between 8 and 10 ppm ^1^H chemical shift indicative of an intact cystine knot fold. 2D NMR spectra were overlaid, analyzed, assigned, and integrated using NMRView version 5.2.2^69–72^. 2D ^1^H-^1^H NOESY spectral peak lists were calibrated at a reference distance of 5.2 Å and a tolerance spectral resolution limit for ^1^H-^1^H NOE resonance set at 0.03 ppm. 2D ^1^H-^1^H NOESY spectral peak lists were converted into 233 upper limit distance constraints for automated structure calculation using CYANA version 3.97^73^. In addition, disulfide bond restraints were generated for the three disulfide bonds. 2, 90, 86, 45, and 97 intra-residue, sequential, short-range, medium-range, and long-range upper limit distance constraints, respectively, were observed. A structural ensemble of 100 different structural conformers based on upper limit distance constraints using simulated annealing and torsion angle dynamics protocol were calculated and the top 20 lowest energy structures were archived. NMR structural ensembles of R_0_1-MG wildtype and [^18^F]FP-R_0_1-MG FP-labeled cystine knot recognizing the integrin α_v_β_6_ cancer recognition site were deposited in the protein data bank with coordinates (2N8B.pdb) and (2N8C.pdb).

### Crystallization of [^19^F]FP-R_0_1-MG-F2

Initial crystallization conditions were screened with four commercial screening kits: (1) Crystal Screen HT (CSHT), 2) SaltRx HT, 3) PEG/Ion HT, and 4) Index HT by Hampton Research (Aliso Viejo, CA). Screens were performed with 96-well Intelliplates on a Crystal Gryphon Flex Instrument (Art Robbins Instruments, ARI, Sunnyvale, CA). Briefly, 0.3 μl or 0.6 μl of [^19^F]FP-R_0_1-F2 (15 mg ml^−1^) were added to 0.3 μl of screening solution. Images were obtained using a CrysCam Digital Microscope System (ARI). At ~24 h, crystals were observed in SCHT wells B7, C3 and D4, which contained 30%, 20% and 20% v v^−1^ 2-propanol, respectively. (Supplementary Fig. 2).

*Summary of Buffer Compositions*: B7: 0.2 M Ammonium acetate, 0.1 M TRIS hydrochloride pH 8.5, 30% vv^−1^ 2-propanol

C3: 0.2 M Sodium citrate tribasic dehydrate, 0.1 M HEPES sodium pH 7.5, 20% v v^−1^ 2-propanol

D4: 0.1 M Sodium citrate tribasic dehydrate pH 5.6, 20% v v^−1^ 2-propanol, 20% w v^−1^ PEG 4000

Noting the three isopropanol-containing CSHT solutions, a 24-way optimization grid was set-up with a Scorpion robotic instrument (ARI). The CSHT D4-based wells contained 0.1 M Sodium citrate pH 5.6. The variables included 16–26% PEG 4 K (*x*-axis) and 15–30% 2-propanol (*y*-axis). Crystals were observed throughout the grid (Fig. 6b). Next, the concentration of [^19^F]FP-R_0_1-F2 was decreased to 10 mg ml^−1^ to produce discrete high-quality crystals (50–200 μm), which grew slowly after several days in a well that contained 0.1 M sodium citrate tribasic dehydrate pH 5.6, 25% v v^−1^ 2-propanol and 18% w v^−1^ PEG 4000. Crystals were preserved in a universal cryo-protectant, mounted on magnetic ALS CrystalCap/Cryoloop pins (Hampton Research) and stored in liquid nitrogen.

### X-ray data collection of [^19^FN]FP-R_0_1-MG-F2

X-ray diffraction experiments were performed at the MBC Beamline 4.2.2 of the Advanced Light Source using the RDI-8 m CMOS detector^74^. Crystals were tested for diffraction using a Superbend magnet source coupled to a Rosenbaum-Rock Si(111) sagitally focused monochromator with an energy range of 5500–16,000 eV. An ACTOR robot (Rigaku) was used to load frozen crystals into position on the beamline. A number of CSHT-optimized D4 crystals (50–200 μm) were screened and produced high-quality diffraction data beyond 1.0 Å resolution. For each crystal, 180 degrees of data were collected in shutterless mode with 0.1 degree frames at an energy of 1.00 Å and a temperature of 100 K; if necessary, a second dataset was collected on the same crystal at 50% attenuation to record overloaded reflections from the first pass. Datasets from individual crystals were indexed, integrated, scaled, and merged in the XDS/XSCALE package^75^. The scaled reflections were converted to mtz format with Free R flags in the CCP4 package^76,77^.

### X-ray structure determination of [^19^F]FP-R_0_1-MG-F2

In order to solve the structure, we used a high-quality dataset obtained from a crystal that diffracted to 1.05 Å (Table 2). The phases for [^19^F]FP-R_0_1-MG-F2 were solved using a molecular replacement search model made from the NMR structure of the parent peptide scaffold *Momordica Cochinchinensis* Trypsin Inhibitor II (MCoTI-II, 2IT8.pdb and 2N8B.pdb) subsequently engineered by our group to develop the precursor peptide R_0_1-MG. MCoTI-II and R_0_1-MG share ~60% sequence identity; they are different in their active Loop-1 (Supplementary Fig. 2).

The initial search model was generated by deletion of the active 2000-1 and conversion of all other non-identical (non-loop) residues to serine. Molecular replacement program PHASER, which is a part of the CCP4 crystallography program suite, was used to search two molecules in the asymmetric unit (ASU)^76,78^. An initial solution with the search model was obtained in *P*2_1_2_1_2_1_ space group.

The model-building program ARP/wARP was used to automatically construct more than 90% of the model in the ASU^79^. A complete model was obtained by further cycles of manual refinement and loop building in Refmac5 (CCP4 suite) and COOT^80^. Once all the amino-acid residues were fitted to the electron density map, the N-terminus fluropropyl group was manually determined by using the ligand generating tools in the CCP4 suite and COOT. Further refinement cycles led to a final molecule, 6CDX.pdb, with an R-Factor (*R*_work_) of 0.18 and a resolution of 1.05 Å.

### Toxicology study on the reference standard [^19^F]FP-R_0_1-MG-F2

A toxicity study of [^19^F]FP-R_0_1-MG-F2 peptide in Sprague Dawley rats was conducted by Sobran Inc, Bioscience Division, Sobran Rangos Animal Facility (Baltimore, MD). A complete report called “14-Day Single Intravenous Dose Toxicity Study of [^19^F]FP-R_0_1-MG-F2 in Sprague Dawley Rats” was dated February 24, 2015 (Supplementary Information).

Briefly, twenty male and female rats (10 sex^−1^ group^−1^, 5 sex^−1^ sacrifice timepoint^−1^) were assigned to a dose group and a vehicle control group and administered [^19^F]FP-R_0_1-MG-F2 peptide tracer intravenously via the tail vein at 0 (vehicle only) and 1.10 mg kg^−1^ (0.22 mg mL^−1^) as a single dose on Study Day 1. Groups of 5 animals sex^−1^ were sacrificed on Study Days 3 (48 h) and 15 for evaluation of clinical pathology and organ toxicity. The dose of [^19^F]FP-R_0_1-MG-F2 used for these toxicity studies was more than 250× the maximum estimated dose of the [^18^F]FP-R_0_1-MG-F2 tracer that may be injected into a human subject. The animals were monitored prior to the administration of the test article, [^19^F]FP-R_0_1-MG-F2, and up to 14 days following the administration of the test article. Parameters evaluated for test article effect included survival, clinical observations, body weight, body weight gain, clinical pathology, gross pathology, organ weights, and microscopic pathology.

### Clinical inclusion and exclusion criteria (Stanford)

The following studies were approved by Stanford’s Institutional Review Board and Scientific Review Committee. The clinical studies were conducted in the Division of Nuclear Medicine and Molecular Imaging at Stanford University Nuclear Medicine Clinic under US-FDA eIND 126379 (S.S. Gambhir), and ClinicalTrials.gov Identifier NCT02683824.

### Healthy volunteer inclusion criteria

Volunteers met all of the following inclusion criteria and were considered eligible for participation in this study.

- 18-year old or older at the time of radiotracer administration
- No known medical problems and completed a full medical exam within 6 months of the study
- Understood and voluntarily signed an Informed Consent after its contents were fully explained

### Pancreatic cancer patient inclusion criteria

Cancer patients met all of the following inclusion criteria and were considered eligible for participation in this study.

- 18-year old or older at the time of radiotracer administration
- Provided written Informed Consent
- Diagnosed with pancreatic cancer and scheduled to undergo surgery
- Able to remain still for duration of each imaging procedure (about 1 h)

### IPF patient inclusion criteria

- 18-year old or older at the time of radiotracer administration
- Patient provided written Informed Consent
- Patient diagnosed with IPF by a pulmonologist according to ATS guidelines
- Patient has high-resolution CT with usual interstitial pneumonia (UIP) pattern
- Patient has PFT’s within the last 12 months with

- FVC < 85% predicted
- DLCO < 65% predicted
- FEV1/FCV ratio > 0.7

- Patient is able to comply with study procedure

- Scanning Option A (60 min dynamic PET scan and two 20 min static PET scan) OR
- Scanning Option B (two 10 min static PET scans)

### Healthy volunteer exclusion criteria

If volunteers met any of the following exclusion criteria, they were considered ineligible for participation in this study.

- Less than 18-year old at the time of radiotracer administration
- Pregnant or nursing

### Pancreatic cancer patient exclusion criteria

If cancer patients met any of the following exclusion criteria, they were considered ineligible for participation in this study.

- Less than 18-year old at the time of radiotracer administration
- Pregnant or nursing
- Contraindications for PET/CT
- Unable complete a PET/CT scan
- Unable to comply with the study procedures
- Serious uncontrolled concurrent medical illness that would limit compliance with study requirements
- Eastern Cooperative Oncology Group Performance Status (ECOG PS) > 2

### IPF patient exclusion criteria

- Patients with serious uncontrolled concurrent medical illness that would limit compliance with study requirements.
- Patient has history of any clinically significant lung disease other than IPF as determined by pulmonologist.
- Patient has had a lung infection of any kind in the last 3 months.

### Healthy volunteer, Panc CA* and IPF patient** (Stanford)

1. Participant was asked to drink 1-2 glasses oher arrival at the clini/her arrival at the clinic.
2. Participants was consented by the responsible physician.
3. Female participants had an early pregnancy test (EPT) to rule out pregnancy.
4. Participants was asked to urinate prior to start of study and instructed to collect a urine sample.
5. Participant was weighed and measured in height.
6. Participant had an IV line placed in an arm vein for tracer administration.
7. Participant had a second IV line placed in an arm vein for blood sampling.
8. A blood sample was taken 5 min prior to the injection of the tracer and used for baseline chemistry and hematology laboratory testing.
9. [^18^F]FP-R_0_1-MG-F2 was formulated in a sterile and pyrogen-free isotonic solution and was administered in a single slow IV injection. The line was flushed with at least 10 ml normal saline after injection.
10. Blood samples for blood time activity measurements were taken at 1, 3, 5, and 10 min after tracer administration and then at 30-minute intervals for up to 3 h after tracer injection. The counts in whole blood and plasma were measured by gamma counter (Perkin Elmer Wizard 1470). All blood counts were decay corrected to the time of tracer injection.
11. A small low activity calibration source of known activity of ^18^F was placed in the field of view in each image collected.
*For pancreatic cancer, dynamic PET scans were acquired for approximately 45 min after intravenous administration of the radiopharmaceutical [^18^F]FP-R_0_1-MG-F2. The scan was performed over the abdominal region to include the pancreas. Attenuation correction was employed via CT (120 kV, 10 mA). For the pancreatic cancer patient, an [^18^F]-FDG PET scan of the abdominal region was performed on a different day prior to the [^18^F]FP-R_0_1-MG-F2 study.
**For the IPF cohort (option A), dynamic PET scans were acquired for approximately 60 min after intravenous administration of the radiopharmaceutical [^18^F]FP-R_0_1-MG-F2. The scan was performed over the chest region. Attenuation correction was employed via CT (120 kV, 10 mA).
14. Static total-body (vertex-to-toe) PET/CT scans were acquired at 60 min and 120 min post injection. CT was performed at 120 kV; it was dose-modulated based on body habitus with current ranging from 10-105 mA for the 60-minute scan. Attenuation correction was also used with CT for the 120-minute scan (120 kV, 10 mA).
15. Blood pressure, temperature, heart rate and pulse oximetry measurements were taken before injection (baseline) and at 5, 10, 15, 60, and 120 min after [^18^F]FP-R_0_1-MG-F2 injection.
16. EKG monitoring was performed before [^18^F]FP-R_0_1-MG-F2 injection and then at 15 min intervals for up to 3 h after [^18^F]FP-R_0_1-MG-F2 injection. EKG data was analyzed to assess changes in overall rhythm of the heart.
17. On the day of the study, participants were asked to void frequently to reduce radiation exposure. Urine samples were collected at prior to tracer administration (baseline), prior to the static PE scans, at the end of the study day 1, and at the follow up visits 1 day and 7 days after tracer administration. Urine was analyzed by gamma counting.
18. Participants were given a copy of the informed consent form that they signed and were dismissed.
19. Adverse drug events were recorded for 3 h after [^18^F]FP-R_0_1-MG-F2 injection and at 24 h and 1 week after [^18^F]FP-R_0_1-MG-F2 injection.
20. Participants were asked to return to the clinic the next day and at 7 days post injection of tracer, for blood sample collection (for blood chemistry and hematology) and also for monitoring of vital signs.

### Administration of [^18^F]FP-R_0_1-MG-F2 in healthy volunteers

A bolus of [^18^F]FP-R_0_1-MG-F2 was administered intravenously to five healthy male and female adult volunteers; the mean ± SD injected dose of [^18^F]FP-R_0_1-MG-F2 was 7.5 ± 1.3 mCi and the mean injected mass was 12.5 ± 4.0 μg or 0.18 ± 0.06 μg kg^−1^ (Supplementary Table 4). No adverse effects were reported by volunteers or noticed by physicians and staff for up to 7 days after injection of [^18^F]FP-R_0_1-MG-F2. Oxygen levels, vital signs and electrocardiography (ECG) readings stayed within normal range from pre-injection to 24 h and 7 days after administration of the PET tracer (Supplementary Tables 5 and 6).

### [^18^F]FP-R_0_1-MG-F2 vs. [^18^F]FDG in pancreatic CA

Dosages of 10.2 mCi [^18^F]FDG and 5.1 mCi [^18^F]FP-R_0_1-MG-F2 were separately administered on different days to a 61-year-old woman with pancreatic cancer scheduled for surgical resection (Supplementary Table 10).

### [^18^F]FP-R_0_1-MG-F2 in IPF lungs

One female and five male study subjects, 72.5 ± 1 y.o., who were diagnosed with IPF according to international consensus diagnostic criteria of either a chest CT demonstrating a definite UIP pattern or lung tissue biopsy with a histopathologic UIP pattern, were administered 5–10 mCi of [^18^F]FP-R_0_1-MG-F2^1^. One male study subject experienced dyspnea, elevated heart rate, coughing and shivering during the study. The female study subject reported shortness of breath and nausea after 48 h. Upon review by our nuclear medicine physicians and pulmonary physicians, it was determined that these potentially adverse reactions were not likely study-related, but rather a result of the underlying disease.

### Analysis of radiotracer in study subject’s blood

Blood samples for time-activity measurements were obtained at 1, 3, 5, and 10 min after tracer injection and then at 30-min intervals for up to 3 h after tracer injection. For each timepoint, 500 μL of blood was transferred to a 13 × 75 mm plastic tube containing 75 USP sodium heparin (Benton Dickinson Vacutainer 367871). A separate fraction of 500 μL aliquot of blood was spun in a clinical centrifuge for 5 min at room temperature. Two hundred microliter of the plasma fraction from this second aliquot was transferred to a new Vacutainer tube. In order to match the volume of the whole blood sample, water was added to the plasma fraction and to the cellular fraction to a final volume of 500 μL. The radioactive counts in whole blood (first fraction), and the plasma fractions were measured with a gamma counter (Perkin Elmer Wizard 1470). All blood counts were decay corrected to the time of tracer injection.

### Dosimetry (OLINDA)

Organ doses values were calculated using organ-level internal dose assessment (OLINDA) software (Vanderbilt University, 2003)^61^. The organs with highest uptake were included in the assessment. These include the kidneys, stomach, small intestine and bladder. A threshold-based segmentation on the last frame of dynamic PET images were used to determine ROIs for the kidney and bladder due to their high tracer uptake relative to background. PET/CT overlay images were used to manually trace ROIs of the stomach and small intestine. Organs with lower uptake, were also included in the dosimetry assessment including the pancreas, liver, lung, and heart. ROIs for these organs were also determined manually on PET/CT overlay images. Muscle uptake was also included in this study due to its large mass representation. In addition, a whole-body ROI was determined by threshold-based segmentation with the baseline threshold value set to exclude image noise occurring outside the body. ROIs were drawn in consensus (by R.H.K, L.B, and F.H.). Normalized cumulative activity was determined for each organ from the area under the curve (AUC) which was calculated using the trapezoidal rule and assuming a physical decay only after the last measurement divided by the total injected activity. These data were used to estimate the radiation doses absorbed by all of the organs.

### Immunohistochemistry

Formalin-fixed paraffin-embedded tumor tissues were sectioned at 5 µm thickness. All histologic sections were stained with standard hematoxylin-eosin immunohistochemical staining (H&E). To confirm the presence of α_v_β6, additional sections were stained with immunohistochemistry (IHC) for anti-human α_v_β6 (0.625 μg ml^−1^, 6.2A1, Biogen Idec, Cambridge, MA). For preparation of IHC staining, the slides were deparaffinized with xylene and rehydrated in serially diluted ethanol solutions (100–50%), followed by demineralized water according to standard protocols. Endogenous peroxidase activity was blocked by incubation in 0.3% hydrogen peroxide in phosphate buffered saline (PBS) for 20 min. Antigen retrieval for α_v_β_6_ was performed with 0.4% pepsin incubation (Dako) at 37 °C for 10 min. Following antigen retrieval, the tissue sections were incubated overnight with the primary antibodies in 100 µl 1% BSA in PBS at room temperature. The slides were washed with PBS, followed by incubation with secondary antibody at room temperature according to the Vectastain ABC HRP kit (Peroxidase, Mouse IgG, Vector Laboratories Inc., Burlingame, CA). After additional washing, the staining was visualized with 3,3-diaminobenzidine tetrahydrochloride solution (DAKO, Glustrup, Denmark) at room temperature for 5 min and counterstained with hematoxylin for 20 s. Finally, the tissue sections were dehydrated and mounted in Pertex (Histolab, Rockville, MD, USA).

### [^68^Ga]NODAGA-R_0_1-MG PET/CT imaging (PUMC Hospital)

The following study was conducted at Peking Union Medical College Hospital under local Institutional Review Board oversight. This study is compliant with the guidance of the Ministry of Science and Technology (MOST) for the review and approval of human genetic resources. A dosage of ~2 mCi [^68^Ga]NODAGA-R_0_1-MG was administered to two adult male patients (lung and pancreatic cancer) and four adult female patients (3 cervical and one pancreatic cancer) who were diagnosed with cancer, as confirmed by tissue biopsy (Figs. 4 and 5, Supplementary Table 11). One patient reported nausea and vertigo ~1 h p.i.; the complaints lasted about an hour and disappeared without treatment. The remaining six study subjects did not report any adverse reactions, and none were observed by the attending physicians and clinical researchers.

Study participants did not prepare in any way (i.e. fasting) for the imaging study. Each patient, except for one (described below), underwent a single whole-body static PET/CT scan at 29–60 min after intravenous injection of [^68^Ga]NODAGA-R_0_1-MG. The administered dose was 55.5–96.2MBq (1.5–2.6 mCi). The scan (6 bed positions, 2 min bed^−1^) covered an area that included the top of the skull to the middle of the femur. OSEM was applied for reconstruction. One patient with cervical cancer underwent multiple sequential whole-body static PET scans. For this cervical cancer patient, a single whole-body low-dose CT scan was performed prior to tracer administration, which was used for reconstruction and as an anatomical reference for the sequential PET images. The CT scan (140 kV, 35 mA, pitch of 1:1, layer of 5 mm thickness; layer spacing of 3 mm, 512*512 matrix, field of view of 70 cm) also covered the top of the skull to the middle of the femur. After the CT scan, the first whole-body PET scan (6 bed positions, 2 min bed^−1^) was acquired concurrent with the intraveneous administration of 77.7MBq (2.1 mCi) [^68^Ga]NODAGA-R_0_1-MG. Immediately upon completion of the first scan, the second whole-body static PET data was acquired. Sequentially, multiple whole-body static PET scans were acquired, one by one, from 0 to 75 min. A total of 6 whole-body static PET scans (6 beds of each timepoint) were performed in this patient.

### [^18^F]FDG PET/CT (PUMC Hospital)

All patients were instructed to avoid strenuous work or exercise for at least 24 h prior to the scheduled study date. Study participants were also instructed to fast for at least 4 h before intravenous administration of the PET tracer. Patients were administered [^18^F]FDG at a dosage of 5.55 MBq (0.15 mCi) per kilogram body weight. Immediately after tracer administration, patients were allowed to rest and relax in a warm, darkened room for approximately 45–60 min. Next, each patient emptied their bladder. The PET scan that followed spanned a region from the mid-thigh area to the base of the skull (six bed positions, 2 min bed^−1^).

### Region of interest

Upon co-registration of the PET and CT images, the CT images were used to determine regions of interest for the organs or tissues reported in the manuscript. These ROIs were applied to the PET images of the brain, pituitary gland, breast, lower and upper large intestine, small intestine, stomach, pancreas, heart wall, liver, lung, muscle, red marrow, bone, skin, and thyroid gland. For high uptake organs such as the kidneys, bladder and for the whole body, threshold-based segmentation of PET images were used to determine a close-fitting ROI around the entire organ or whole body. In order to determine accuracy, these PET-derived ROIs were compared to the CT overlay for a close match. For the pancreatic lesions, several ROIs for the knottin vs. FDG tracers were compared. These data are reported as mean SUVs.

### Statistical analysis

All data are presented as the average value ± the SD of at least three independent measurements. Statistical analysis for animal studies and binding studies were performed by two factor ANOVA without replication analysis using Microsoft Excel. Significance was assigned for *p* values of <0.05. Difference in SUV between healthy volunteers and IPF patients was tested by an exact Wilcoxon ranksum test using Stata Release 15.1 (StataCorp LP, College Station, TX). The correlation between left and right lungs was estimated by an average absolute-agreement intraclass correlation from a one-way random-effects model^34^. Due to high degree of correlation between the two halves of the lungs, only the right ones were used to avoid the transplanted left lung of IPF-4.

### Reporting summary

Further information on research design is available in the Nature Research Reporting Summary linked to this article.

## Supplementary information

Supplementary Information

Supplementary Tox Report

Description of Additional Supplementary Files

Supplementary Movie 1

Supplementary Movie 2

Supplementary Movie 3

Supplementary Movie 4

Supplementary Movie 5

Supplementary Movie 6

Supplementary Movie 7

Supplementary Movie 8

Supplementary Movie 9

Supplementary Movie 10

Supplementary Movie 11

Supplementary Movie 12

Supplementary Movie 13

Reporting Summary

Source Data

## Data Availability

Source data for the following Figures, Supplementary Figs. and Supplementary Tables are provided in a Source Data file, which include Figs. 1B, C, 4C, 6D, 7, Table 3 and Supplementary Figs. 1B, 3C, 4B, 5B, 6, 8, 9, 10B, 11, 14 and Supplementary Tables 2, 3, 7, 8, 13. The NMR and crystal structures can be accessed at the protein data bank under accession codes, 2N8B.pdb (10.2210/pdb2N8B/pdb), 2N8C.pdb (10.2210/pdb2N8C/pdb), and 6CDX.pdb (10.2210/pdb6CDX/pdb), respectively.
