# Supplementary material for: Evaluation of integrin αvβ6 cystine knot PET tracers to detect cancer and idiopathic pulmonary fibrosis
Source: Nat Commun. 2019 Oct 14;10:4673. doi: 10.1038/s41467-019-11863-w (PMC6791878; doi:10.1038/s41467-019-11863-w)
Supplement: Supplementary file 1 — Supplementary Information [file 41467_2019_11863_MOESM1_ESM.pdf]

Supplementary Information

Evaluation of Integrin αvβ6 Cystine Knot PET Tracers to Detect Cancer and Idiopathic Pulmonary Fibrosis

Kimura *et. al.*

Development of the Lead Candidate $R_0$1-MG.

Recently, a cystine knot peptide, $R_0$1, engineered to selectively bind human integrin $\alpha_v\beta_6$ (K_D ~3nM), was validated in mouse models by our lab as [^{64}Cu]DOTA-$R_0$1 and [^{18}F]FB-$R_0$1 PET tracers (Kimura et al., Clin. Can. Res., 2012, and Hackel et al., J. Nucl. Med, 2013). However, during storage and routine handling of the knottin precursor, $R_0$1, oxidation would occur at a methionine residue, forming methionine sulfoxide, in the active loop-1 (CILN<u>M</u>RTDLGTLLFRC, Figure 1A, Supplementary Figure 1); a gain in mass of ~16Da as observed by matrix-assisted laser desorption/ionization time-of-flight mass spectrometry (MALDI-TOF-MS). Tandem MS/MS studies indicated the presence of an extra oxygen atom (data not shown). We therefore employed a site-directed evolution strategy that allowed any of the 20 amino acids to occur at this methionine's position in the loop. These studies yielded three highly-represented clones containing either an arginine ($R_0$1-MR), a glycine ($R_0$1-MG, Figure 1A) or a tryptophan residue ($R_0$1-MW) at that position (Supplementary Figure 1). We hypothesized that non-methionine residues may be better able to resist spontaneous chemical modification resulting from insults such as physical handling, extended storage, multiple freeze thaw cycles in an oxidative environment, or exposure to various chemical environments during coupling reactions.

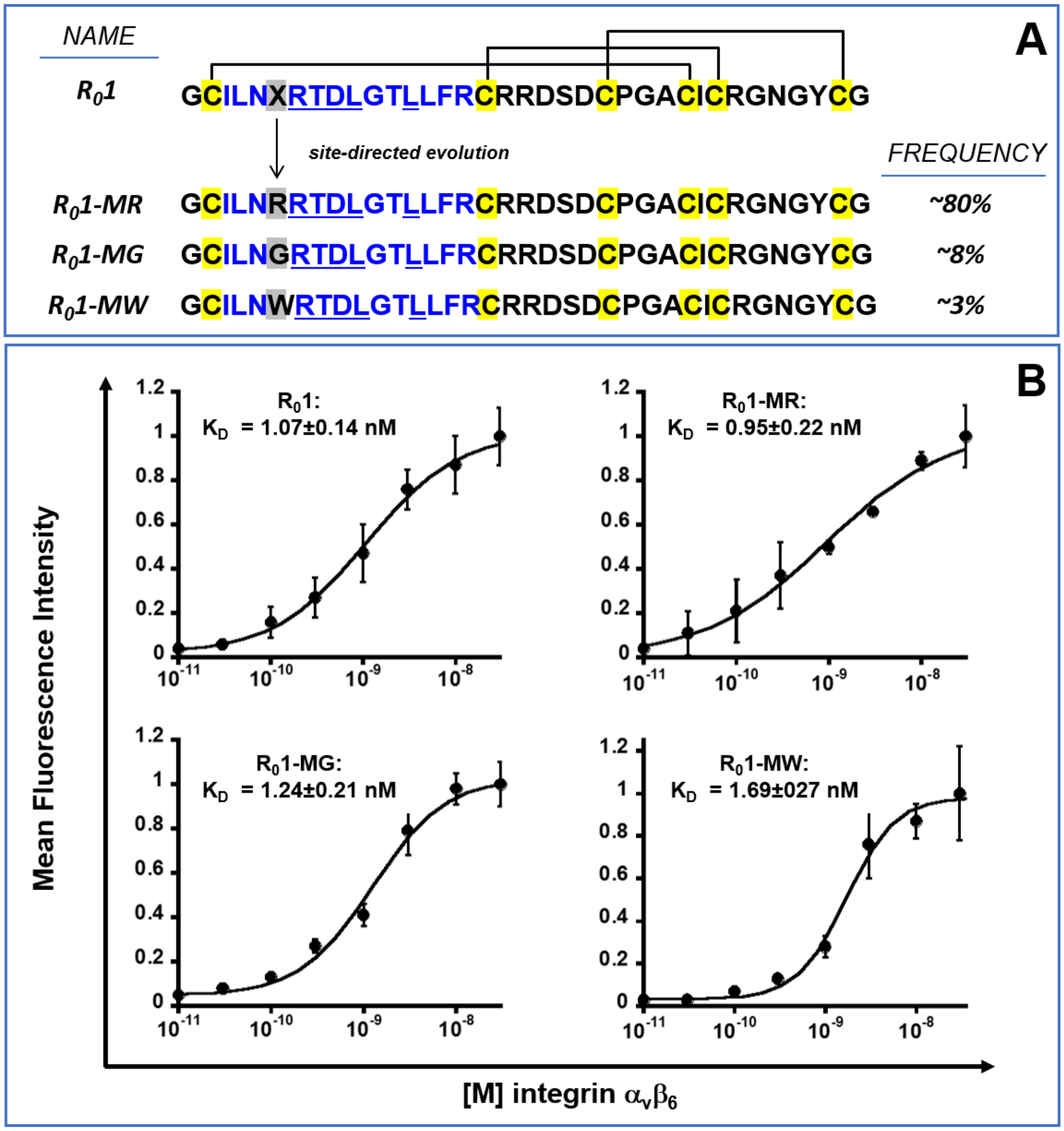

Supplementary Figure 1. Site-Directed Evolution and Determination of Binding Affinity. (A) Site-Directed evolution of $R_0$1. The position of methionine in loop-1 was randomized to any amino acid. The set of 20 mutants competed for molecular recognition of integrin $\alpha_v\beta_6$. The R01-MR variant was present in ~80% of the selected clones. $R_0$1-MG and $R_0$1-MW represented ~8, and ~3% of pool, respectively. The original methionine-containing variant was not present. (B) Equilibrium dissociation constants of the selected variants, $R_0$1, $R_0$1-MR, $R_0$1-MG and $R_0$1-MW are shown in the respective charts. Three replicates were obtained on three separate days to generate an average. Source data are provided in a Source Data File.

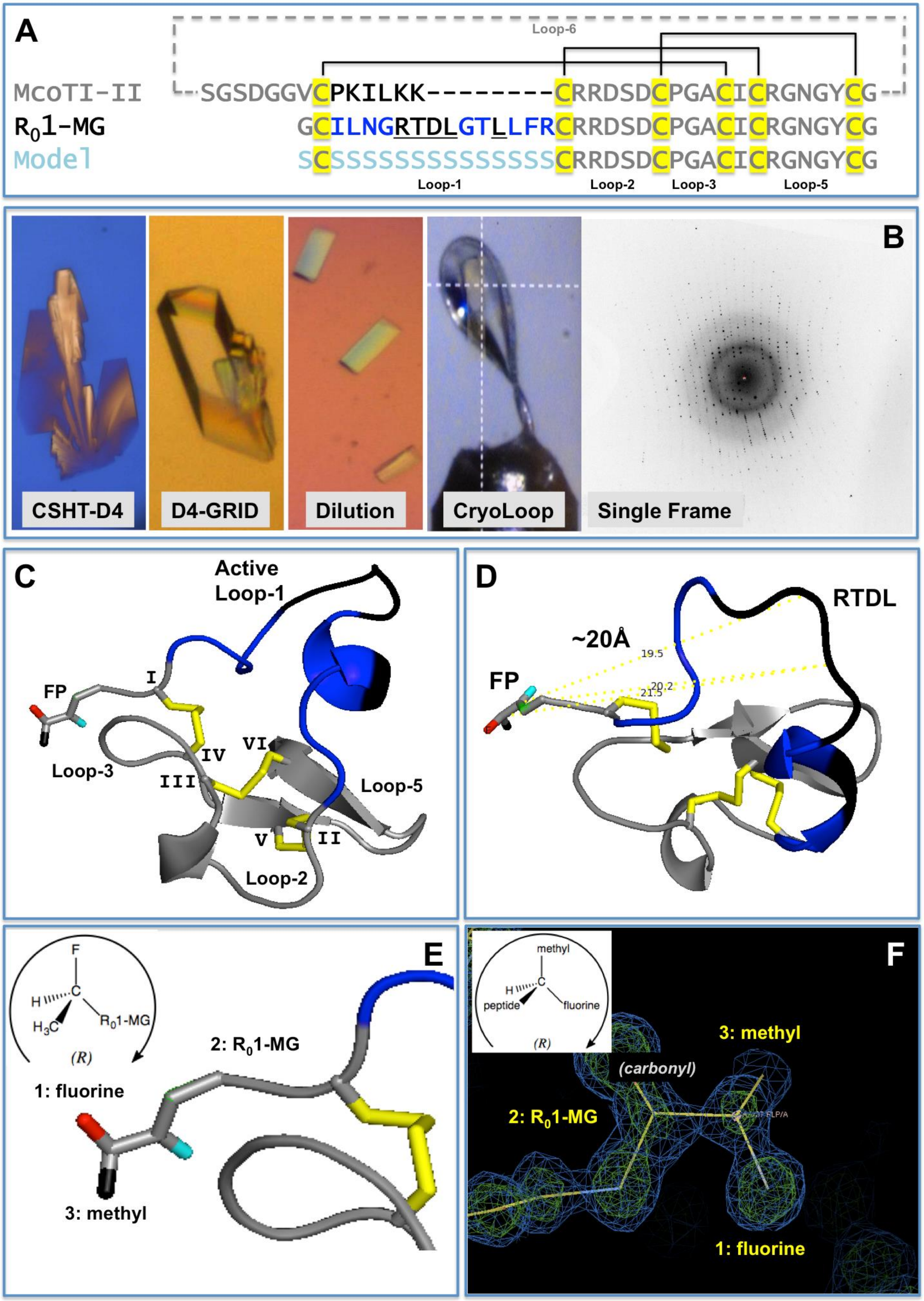

A
Loop-6
McoTI-II
SGSDGGVCPKILKK--------CRRDSDCPGACICRGNGYCG
R0 1-MG
GCILNGRTDLGTLLFRCRRDSDCPGACICRGNGYCG
Model
SCSSSSSSSSSSSSSSCRRDSDCPGACICRGNGYCG
Loop-1
Loop-2
Loop-3
Loop-5
B
CSHT-D4
D4-GRID
Dilution
CryoLoop
Single Frame
C
Active
Loop-1
FP
I
IV
VI
Loop-3
III
Loop-5
V
II
Loop-2
D
RTDL
~20Å
19.5
FP
20.2
21.5
E
F
H
C
H3C
R0 1-MG
(R)
2: R0 1-MG
1: fluorine
3: methyl
F
methyl
peptide
fluorine
(R)
3: methyl
(carbonyl)
2: R0 1-MG
1: fluorine

G

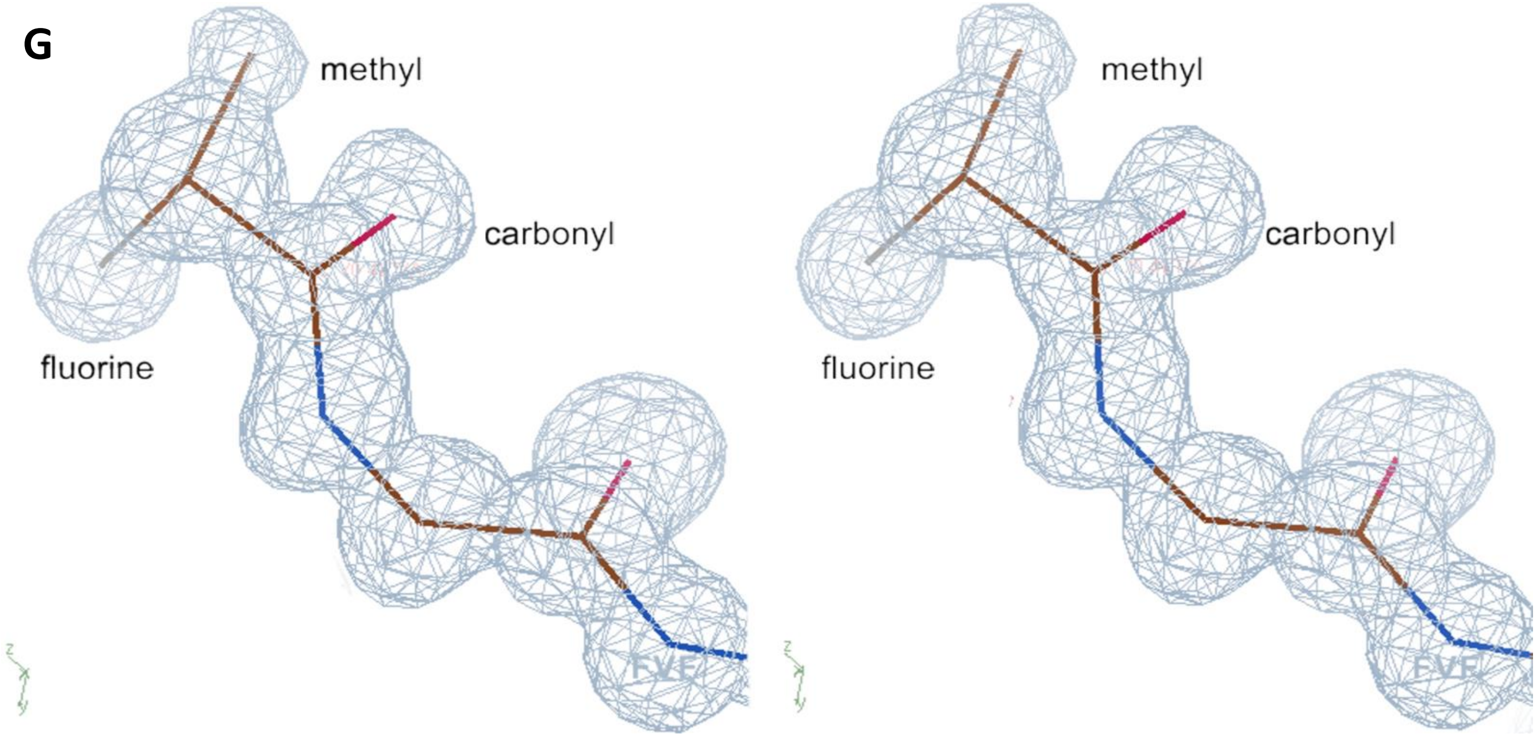

Supplementary Figure 2. Structural Determination of Reference Standard [^{19}F]FP-R$_0$1-MG-F2. (A) The primary structure of *Momordica Cochinchinensis* Trypsin Inhibitor-II (MCoTI-II, grey), the parent cyclic cysteine knot (cyclotide) scaffold derived from the southeast asian fruit "gac", and the linear drug compound precursor peptide R$_0$1-MG (black). "Model" (cyan) represents the initial structure used in molecular replacement studies. The dotted line represents backbone cyclization by an additional loop-6 in MCoTI-II (B) Crystal growth and x-ray data collection. Crystals of [^{19}F]FP-R$_0$1-MG-F2 grown in CSHT-D4 and D4-GRID (Hampton Research (HR), Aliso Viejo California). "Dilution" shows three discrete high quality crystals grown slowly in D4-GRID using a lower initial concentration of [^{19}F]FP-R$_0$1-MG-F2. A ~50um crystal loaded on a CryoLoop MicroTube pin (HR). Single frame shows Beamline 4.2.2 (Advanced Lightsource, Lawrence Berkeley National Laboratory, Berkeley, CA) image of [^{19}F]FP-R$_0$1-MG-F2 taken in 0.1 sec, with 0.1^o angular rotation of crystal. (C-E) Three dimensional structure of the reference standard [^{19}F]FP-R$_0$1-MG-F2. Roman numerals I-VI represent the positions of six cysteine residues and three disulfide bonds (yellow). The active loop-1 is shown in blue, highlighting the RTDLXXL integrin $\alpha_v\beta_6$ recognition motif in black. The red portion of the fluoropropyl (FP) group represents the fluorine atom, the black represents the methyl group, and the cyan represents the carbonyl oxygen of the amide bond connecting the peptide. (D) Distance measurement between the FP group and the RTDLXXL motif indicates approximately 20 Å separation. (E-F) Stereochemical analysis of the chiral center indicates the R enantiomer. (E) Electron density maps. The blue shell and the green shell represent the F_0-F_c, and $2F_0$-F_c maps, respectively. (G) Stereo electron density map of the N-terminus fluoropropyl group.

Synthesis of the FP-labeled PET Tracer and Standard

During the chemical development of [^{18}F]FP-$R_0$1-MG, two closely related forms of the tracer (and the reference standard [^{19}F]FP-$R_0$1-MG) emerged in equal amounts. Interestingly, the isomers had the identical mass and activity relative to the other, even though the two fractions (F1 and F2) were separable by ~ 1min by RP-HPLC (Supplementary Figure 1 and Supplementary Figure 3A). Competition binding assays among the two fractions and the unlabeled peptide indicated that the imaging label did not adversely affect binding affinity for integrin $\alpha_v\beta_6$ (Supplementary Figure 3C). Further evaluation in pre-clinical models demonstrated indistinguishable biological behavior between the two fractions (data not shown). Unfortunately, the first fraction coeluted with a radiochemical impurity, so we were not able to simply translate the mixture. Instead, fraction 2 ([^{18}F]FP-$R_0$1-MG-F2) was purified to >95% and advanced as the lead candidate for further evaluation.

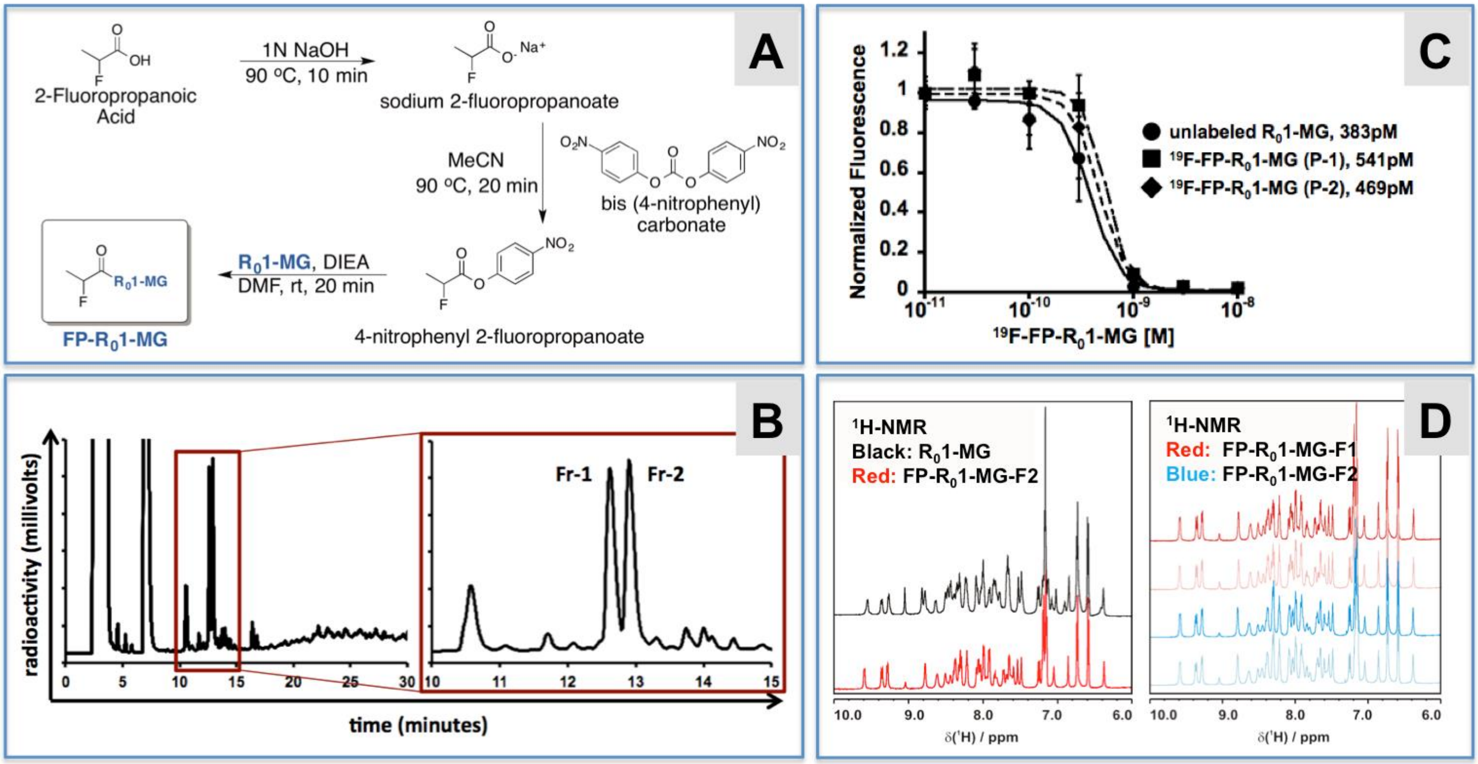

Supplementary Figure 3. Chemical Synthesis and Characterization. (A) Synthesis scheme for the non-radioactive reference standard [^{19}F]FP-$R_0$1-MG. Nonradoactive fluoropropanoaote salt (Sigma-Aldrich Corp., St. Louis, MO), was reacted with bis (4-nitrophenyl) carbonate (Sigma-Aldrich) to yield the active nitrophenyl propanoate ester [NPE], which was subsequently reacted with $R_0$1-MG to produce [^{19}F]FP-$R_0$1-MG. (B) RP-HPLC. Fractions 1 and 2 are separated by ~ 1 minute. (C) Competition Binding (IC_{50}). Unlabeled $R_0$1-MG and both fractions [^{19}F]FP-$R_0$1-MG-F1 and [^{19}F]FP-$R_0$1-MG-F2 recognize integrin a_vb_6 with subnanomolar binding affinity as they effectively compete with $R_0$1-MG displayed on the surface of yeast. (D) 1H-NMR studies of the precursor peptide $R_0$1-MG compared to [^{19}F]FP-$R_0$1-MG-F2 (left panel). ^{1}H-NMR of the two fractions, [^{19}F]FP-$R_0$1-MG-F1 and [^{19}F]FP-$R_0$1-MG-F2 show identical chemical shifts (right panel). Source data are provided in a Source Data File.

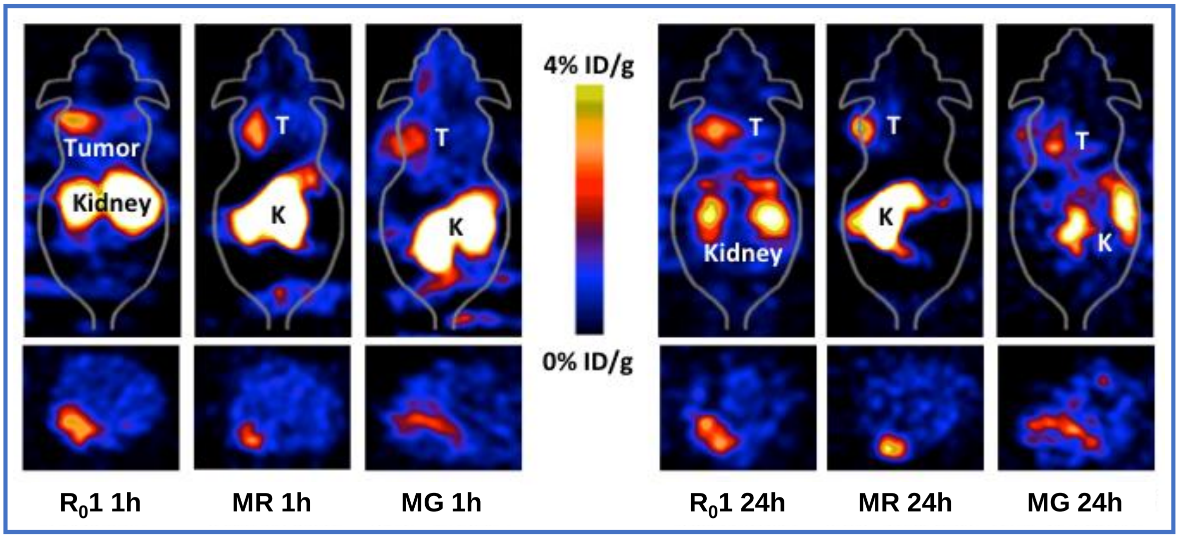

Supplementary Figure 4. **PET Imaging**. (A) [^{64}Cu]DOTA-R$_0$1, [^{64}Cu]DOTA-R$_0$1-MR, [^{64}Cu]DOTA-R$_0$1-MG were evaluated in BxPC3 xenograft mouse models. PET images were acquired 1 hour and 24 hour post injection. Tumor (T), and kidney (K) are identified in the image shown.

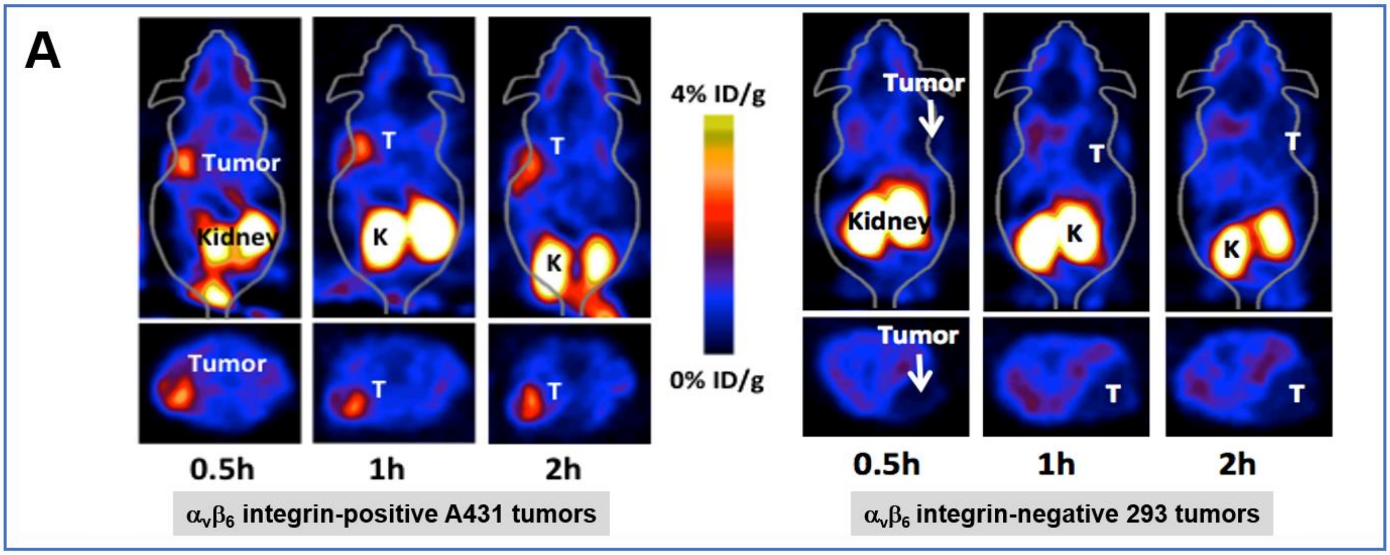

Supplementary Figure 5. [^{18}F]FP-R$_0$1-MG-F2 Static PET Imaging. (A) Representative PET images of *nu/nu* mice bearing in integrin $\alpha_v\beta_6$-positive A431 xenografts (left) and integrin $\alpha_v\beta_6$-negative 293 xenograft (right) models following injection of ~100μCi [^{18}F]FP-R$_0$1-MG-F2. Location of tumor and kidneys are indicated by the letters T and K.

[^{18}F]FP-R$_0$1-MG-F2 Dynamic PET Imaging

Dynamic PET/CT imaging indicated that uptake of [^{18}F]FP-R$_0$1-MG-F2 in integrin $\alpha_v\beta_6$ expressing tumors was rapid and high. At 5 minutes p.i., average tumor uptake reached 5.4 ± 2.7 %ID/g, and plateaued at 5.7 ± 2.8 %ID/g at 60 minutes p.i.. Immediately upon tail vein injection, the PET tracer quickly entered and cleared both the heart and the liver (6C). A region-of-interest (ROI) drawn around the heart indicated that the maximum flow-through of the PET tracer through the heart occurred approximately 0.5 minutes p.i, and quickly decreased by 50% within 1.5 minutes p.i. At 60 minutes p.i., the heart and liver values continued to decrease to ~1.4 %ID/g, and ~1.2 %ID/g, respectively, indicating rapid clearance of the PET tracer from the blood compartment and from normal tissues as shown in 6C. [^{18}F]FP-R$_0$1-MG-F2 cleared primarily via the kidneys where maximum uptake values of ~54 %ID/g were recorded at 4.5 minutes p.i.. Kidney uptake steadily decreased for the duration of the dynamic scan to ~16% ID/g at 60 min p.i., while the radiotracer accumulated in the bladder to ~110 %ID/g. Tumor-to-muscle ratio was approximately 8:1 and 5:1 at 30 and 60 minutes p.i, respectively. The tumor-to-heart ratio was similarly ~7:1 at 30 and 60 minutes p.i. [^{18}F]FP-R$_0$1-MG-F2 did not accumulate to a great extent in liver, which gave rise to contrast ratios of ~7:1 to ~10:1 at time points beyond 30 minutes.

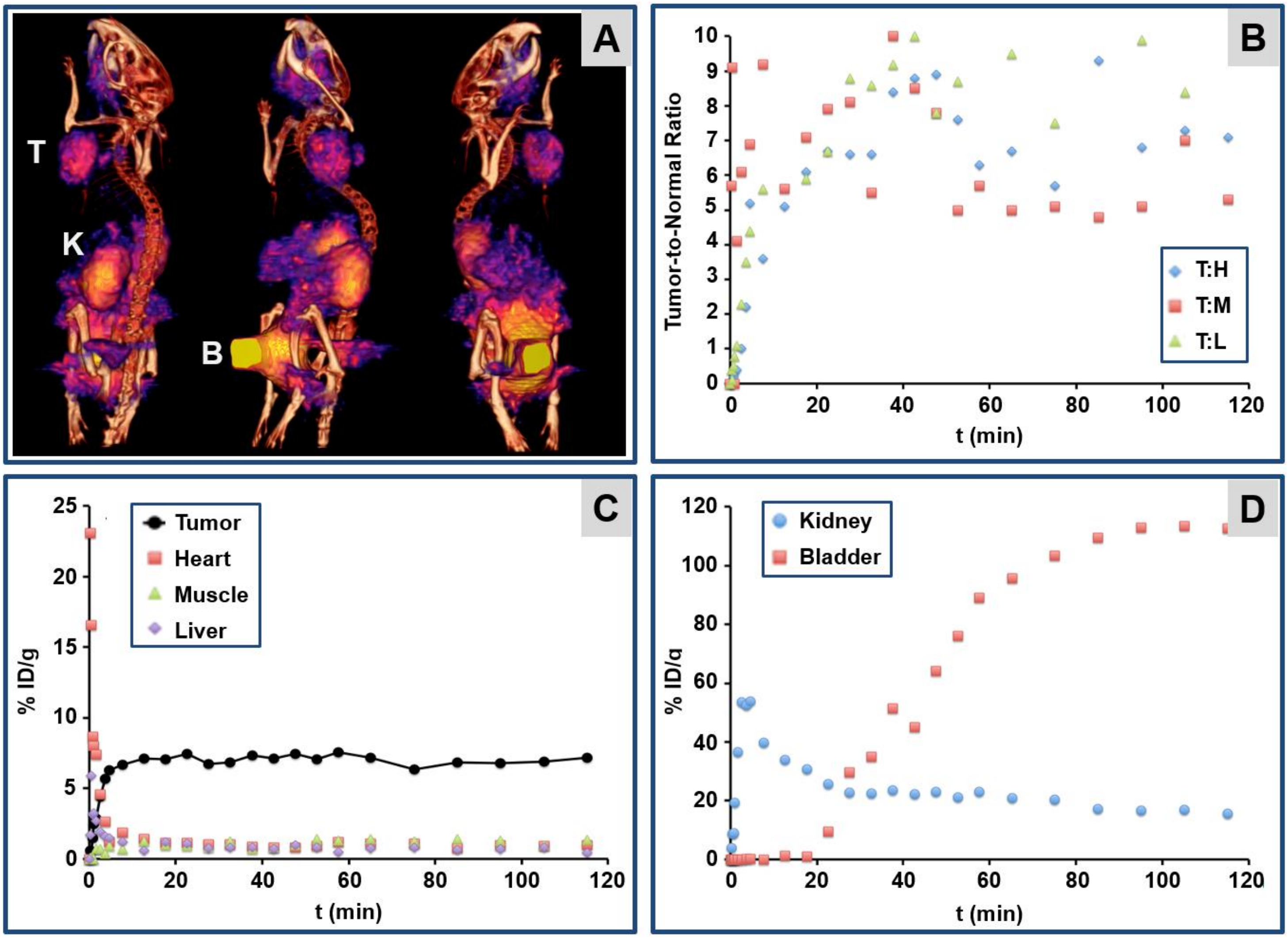

Supplementary Figure 6. Dynamic PET and PET/CT Imaging. Nu/Nu female (*n=3*) mice were anesthetized using 2% isoflurane in oxygen and approximately 100 µCi of the [^{18}F]FP-R$_0$1-MG-F2 was administered via tail-vein. (A) Volume rendered PET images of a representative animal model viewed from 3 different angles. The tumor, kidney and bladder are denoted by T, K and B. Dynamic PET scans were acquired for up to 2 hours post-injection in a small animal PET/CT scanner (1.4-mm resolution, Inveon; Siemens, Malvern, Pa). resulting in the (B) Tumor-to-normal tissue ratios for heart (H), muscle (M) and liver (L). (C) Time activity curves of tumor and normal heart, muscle and liver tissues over a 2 hour dynamic PET scan indicate rapid tumor uptake and (D) renal excretion routes. Source data are provided in a Source Data File.

cGMP Radiosynthesis of PET Tracer and Toxicology Studies

In order to produce the reference standard and the PET tracer, the knottin precursor's sole amine, located at its N-terminus, was used to couple either a [^{19}F]FP group or the radioactive analog, [^{18}F]FP, through the electrophilic carbonyl carbon of the active nitrophenyl ester (Supplementary Figure 7A and 7B).Three cGMP-compliant technical validations runs were performed on three separate days to establish and validate the radiosynthesis protocol for production of clinical-grade [^{18}F]FP-R$_0$1-MG-F2 (Supplementary Figure 7C). Radiochemical purity of [^{18}F]FP-R$_0$1-MG-F2 ranged from 96.1% to 98.3%, and the chemical purify of the probe, for each synthesis, was greater than 99%. In addition, the probe remained stable in injection buffer for two radio half-lives after completion of the radiosynthesis (Supplementary Figure 7D). As shown by radio-HPLC, minor defluorination of the PET Tracer was observed from 2 hr incubation in human serum, and in mouse urine samples collected 2 hr p.i. (Supplementary Figure 7D).

The specific activity of [^{18}F]FP-R$_0$1-MG-F2 was used to calculate the mass of tracer that would be administered to human from a PET study involving a dose of 15mCi (Supplementary Figure 7). For toxicological evaluation, the reference standard [^{19}F]FP-R$_0$1-MG-F2 was administered to 20 male and 20 female 7-week-old Sprague-Dawley rats at 250 times the anticipated clinical dose. Toxicity was evaluated on days three and fifteen following intravenous injection of the reference standard. All rats survived to the scheduled termination date and remained bright, alert and responsive during the course of the study. No treatment-related differences were found in mean body weights, organ weights, clinical pathology, gross pathology, microscopic pathology or in the biochemical composition of blood (Sobran Inc, Baltimore MD). Under the conditions of this study, there were no treatment related findings in Sprague Dawley rats three or fifteen days after a single 1.1mg/kg intravenous dose of [^{19}F]FP-R$_0$1-MG-F2.

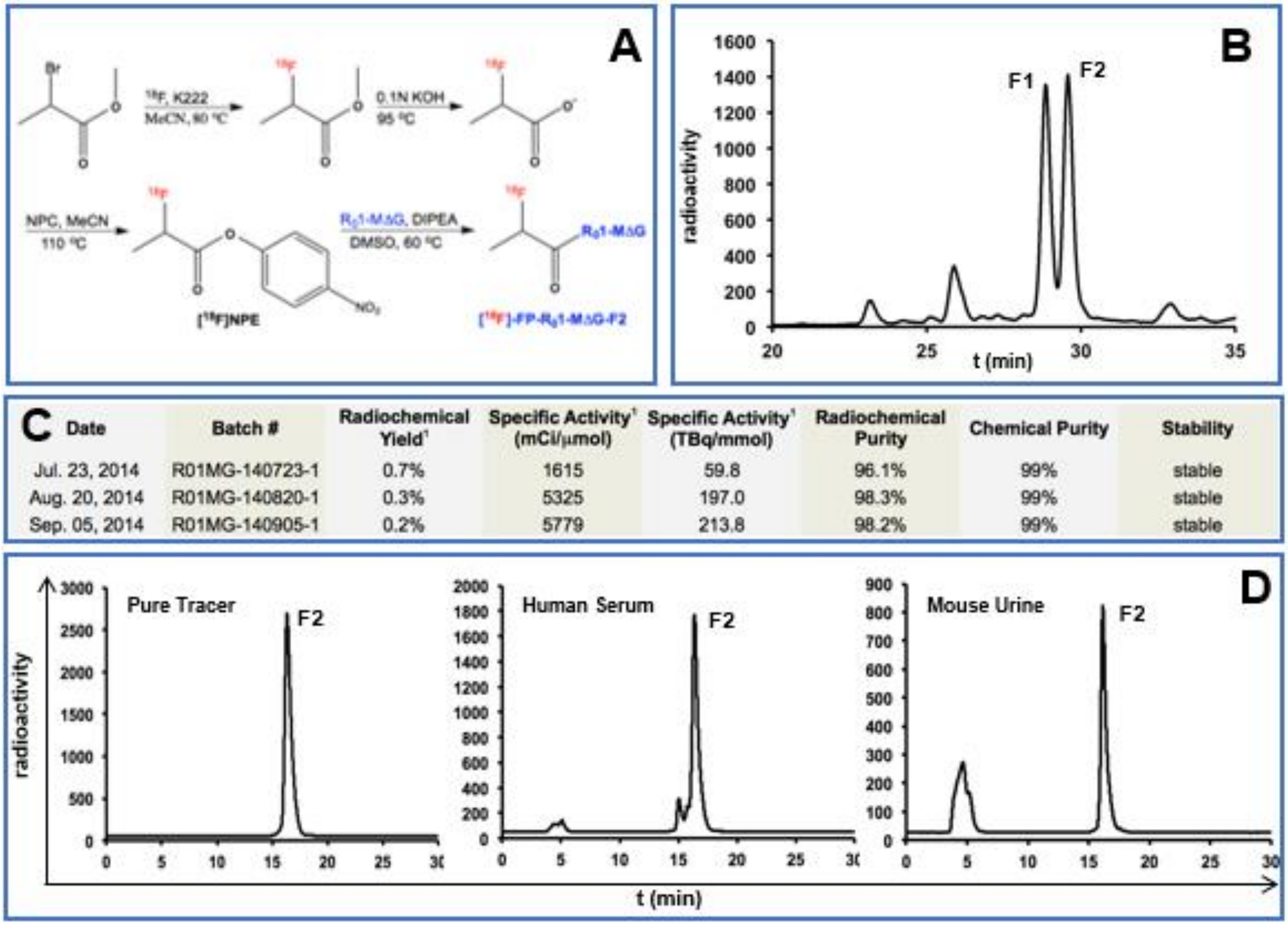

Date	Batch #	Radiochemical Yield[1]	Specific Activity[1] (mCi/µmol)	Specific Activity[1] (TBq/mmol)	Radiochemical Purity	Chemical Purity	Stability
Jul. 23, 2014	R01MG-140723-1	0.7%	1615	59.8	96.1%	99%	stable
Aug. 20, 2014	R01MG-140820-1	0.3%	5325	197.0	98.3%	99%	stable
Sep. 05, 2014	R01MG-140905-1	0.2%	5779	213.8	98.2%	99%	stable

Supplementary Figure 7. Radiosynthesis and Validation of [^{18}F]FP-R$_0$1-MG-F2. (A) Radiosynthetic reaction scheme. The [^{18}F]-fluorpropanoate radiosynthon, was used to produce the reactive [^{18}F] Nitrophenylester (NFE) which was coupled to knottin R$_0$1-MG to produce (B) Two products emerged [^{18}F]FP-R$_0$1-MG-F1 and F2. [^{18}F]FP-R$_0$1-MG-F2 was purified by RP-HPLC to >95% purity. (C) Summary of three technical validation runs. (D) RP-radioHPLC of the purified PET tracer [^{18}F]FP-R$_0$1-MG-F2, and stability assay of tracer after incubation in human serum (37°C, 2h) and mouse urine at 2 hours post injection.

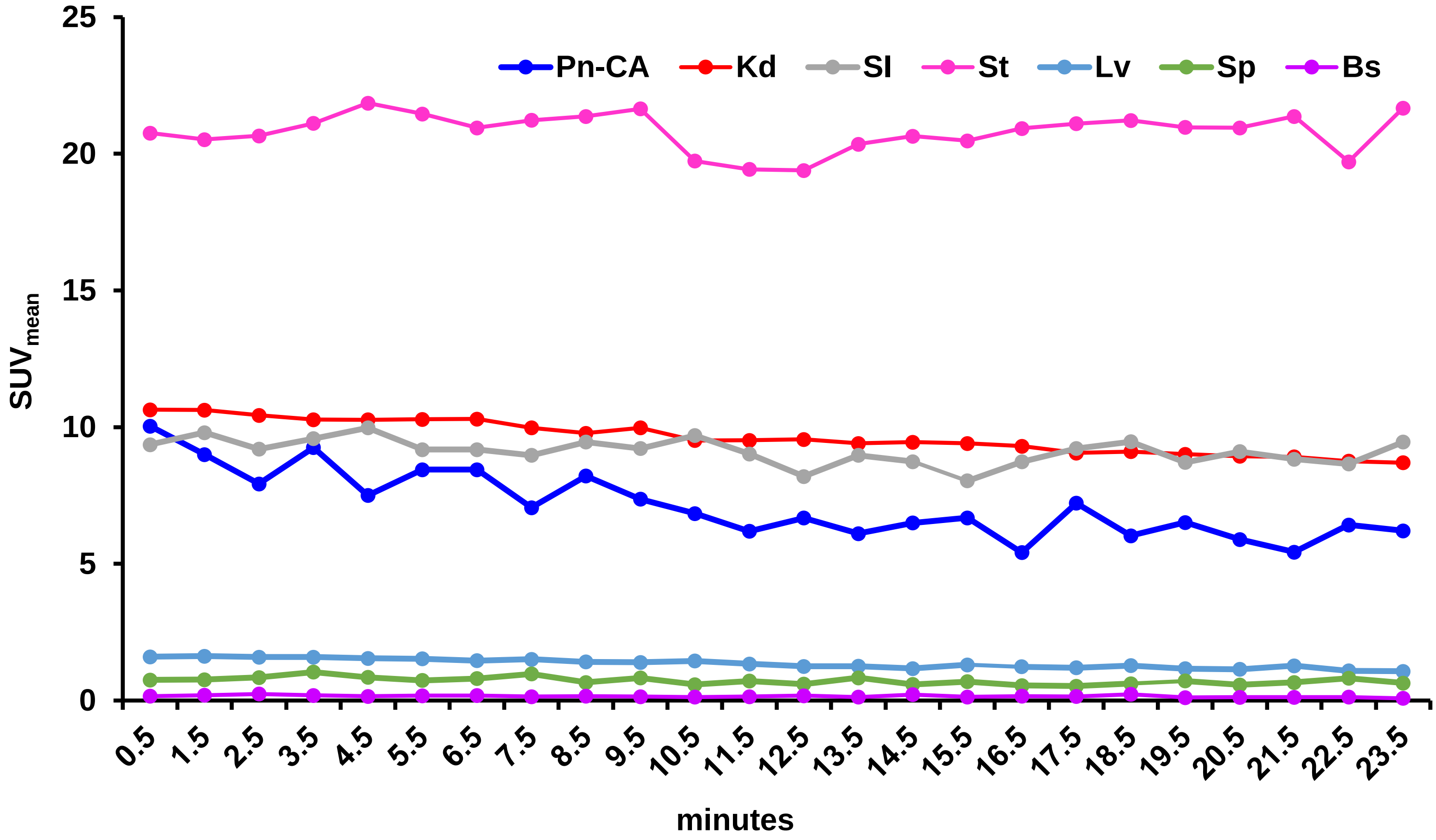

Supplementary Figure 8. **Dynamic PET Study of a Pancreatic Cancer Patient (Stanford).** ROI analysis of the organs that were scanned in a dynamic PET study of the abdomen are given as mean SUV values for 0.5 to 23.5 minutes after injection of [^{18}F]FP-R$_0$1-MG-F2. Pn-CA refers to the tumor in the pancreas. Kidney, small intestine, stomach, liver, spleen and breast tissue are indicated as Kd, SI, St, Lv, Sp and Bs, respectively. Source data are provided in a Source Data File.

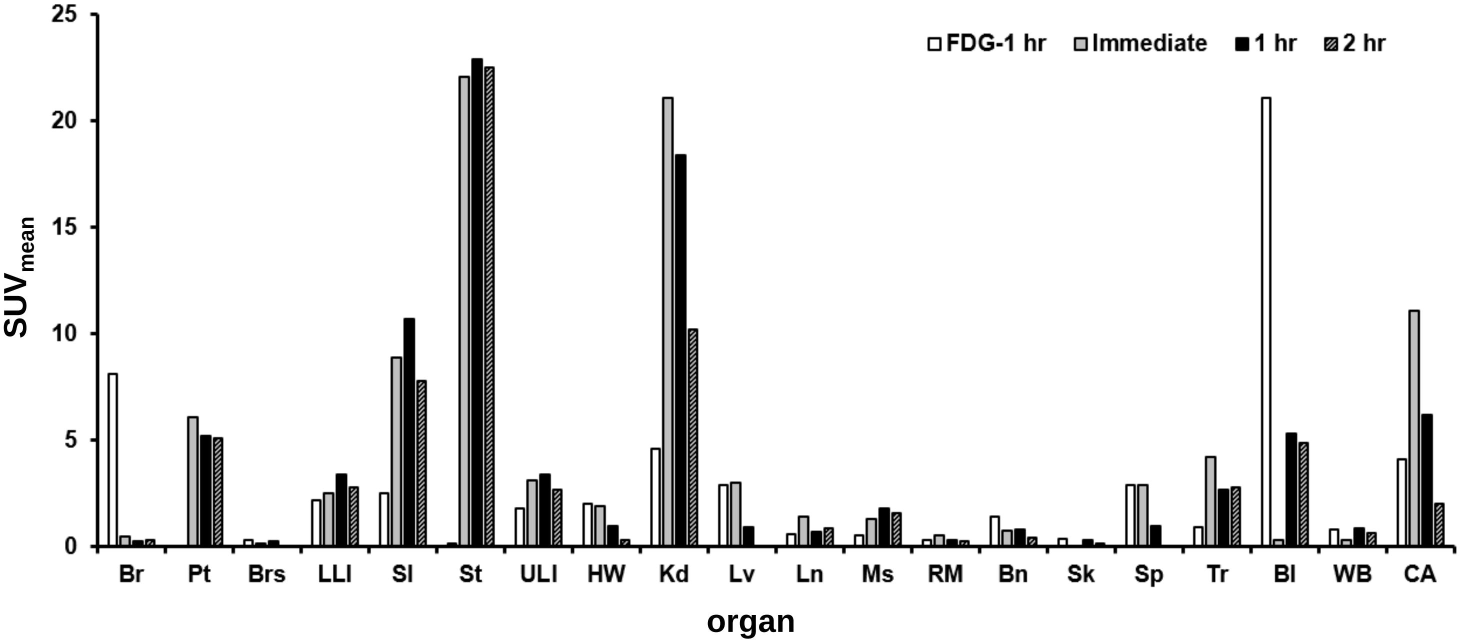

Supplementary Figure 9. Comparative Imaging of a Pancreatic Cancer Patient (Stanford). Comparison of [^{18}F]FDG PET (white bar) and [^{18}F]FP-R$_0$1-MG-F2 (black bar) uptake in normal and pancreatic cancer tissue at 1 hour after injection of tracer. The light and gray bars show uptake of [^{18}F]FP-R$_0$1-MG-F2 immediately after injection and at 2 hours after injection. The following organs are shown: brain (Br), pituitary gland (Pt), breast (Brs), lower large intestine (LLI), small intestine (SI), stomach (St), upper large intestine (ULI), heart wall (HW), kidney (Kd), liver (Lv), lung (Ln), muscle (Ms), red marrow (RM), osteogenic cells (Bn), skin (Sk), spleen (Sp), thyroid (Tr), bladder (Bl), whole body (WB) and pancreatic cancer (CA). Bladder was voided before each [^{18}F]FP-R$_0$1-MG-F2 PET scan. Bladder was not voided prior to the [^{18}F]FDG PET scan. Source data are provided in a Source Data File.

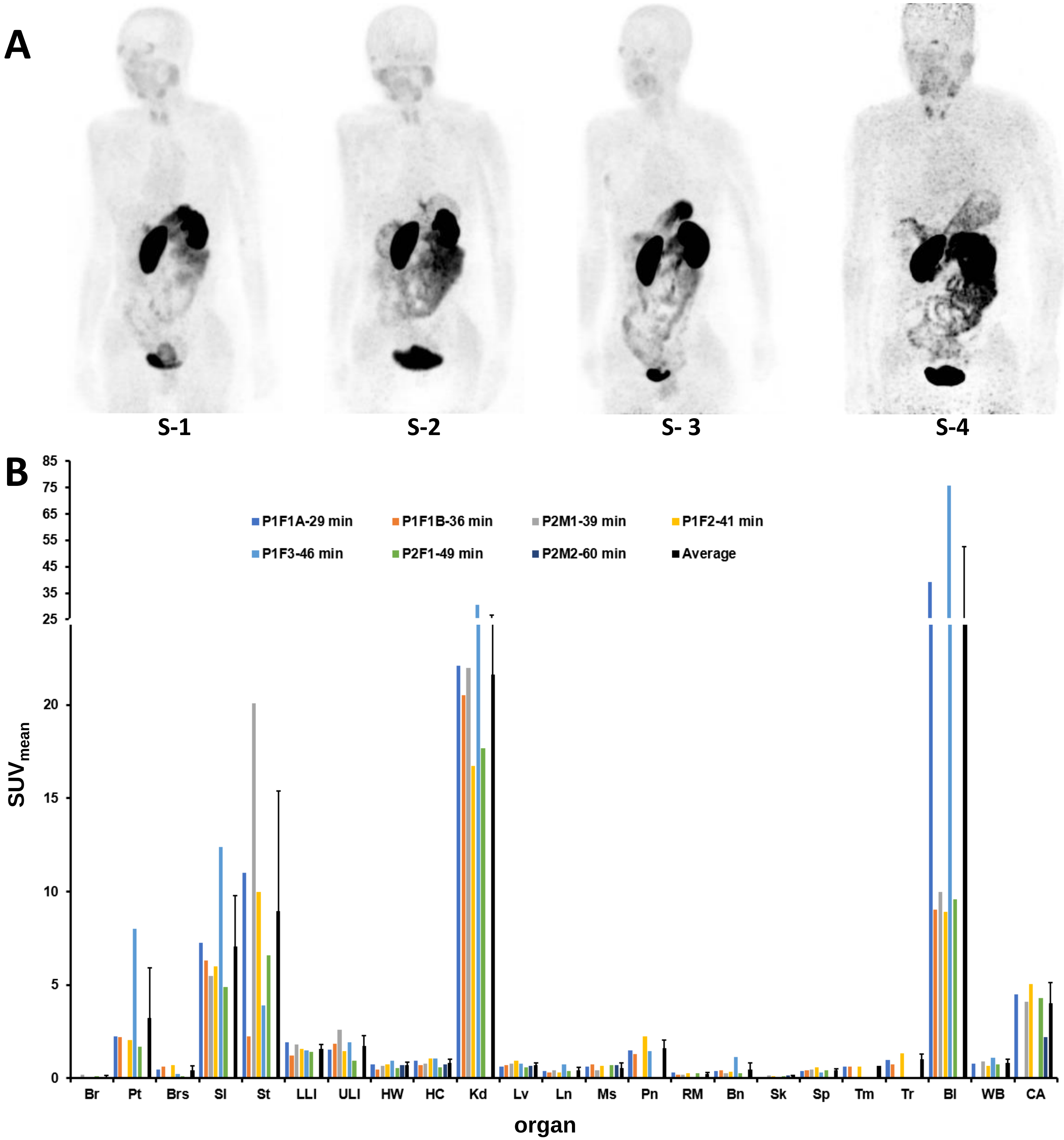

Supplementary Figure 10. [68Ga]NODA-GA-R$_0$1-MG PET Study of Patients Imaged at Peking Union Medical College Hospital, China. (A) [68Ga]NODAGA-R$_0$1-MG MIP PET images of female study subjects (47 to 69 years-old) acquired ~ 30 to 60 minutes post injection. (B) Static PET scans of 6 cancer patients imaged between 29-60 minutes after injection of tracer. The following organs are shown: brain (Br), pituitary gland (Pt), breast (Brs), lower large intestine (LLI), small intestine (SI), stomach (St), upper large intestine (ULI), heart wall (HW), heart contents (HC), kidney (Kd), liver (Lv), lung (Ln), muscle (Ms), red marrow (RM), osteogenic cells (Bn), skin (Sk), spleen (Sp), thymus (Tm), thyroid (Tr), bladder (Bl), whole body (WB) and cancer (CA). Source data are provided in a Source Data File.

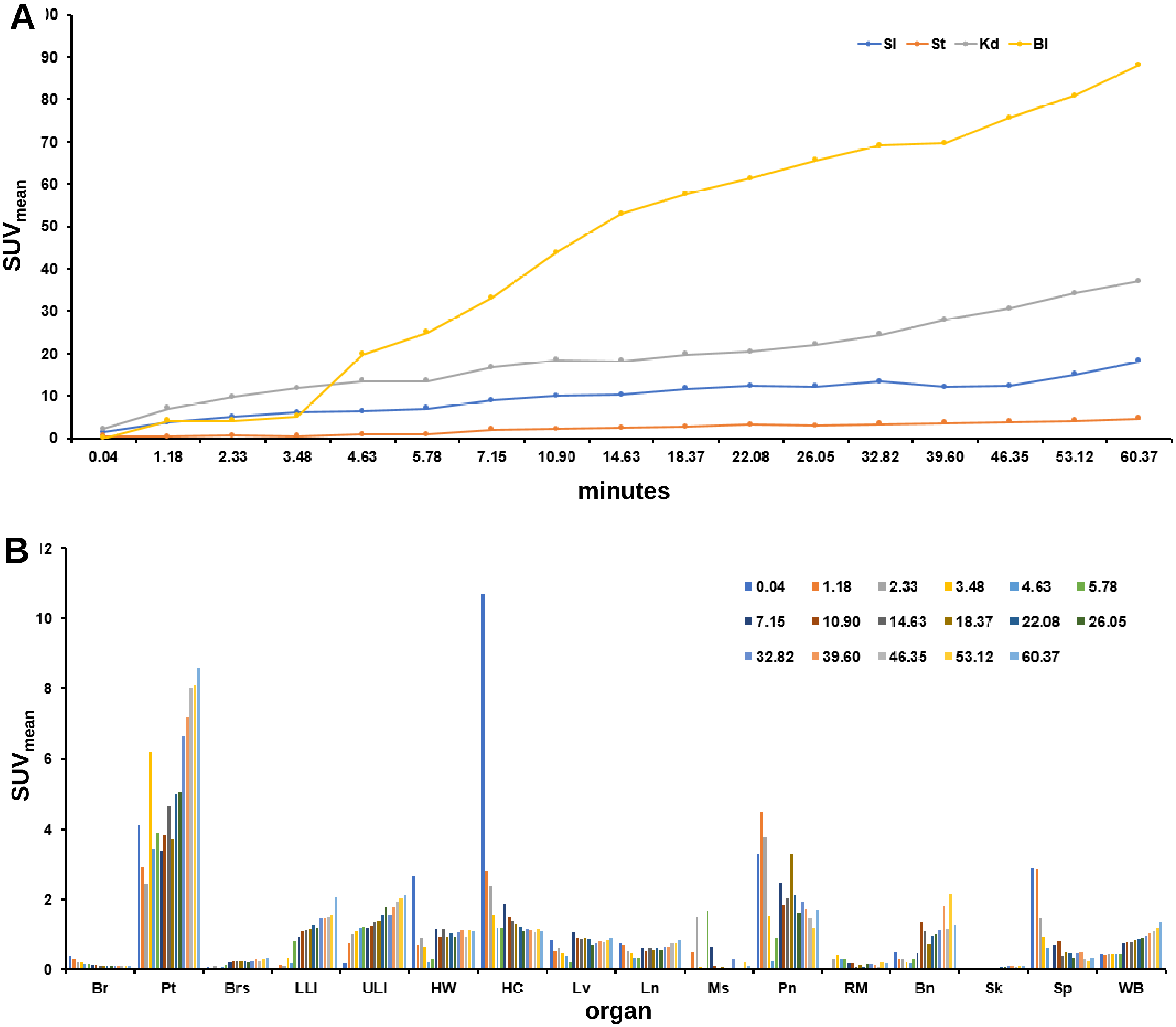

Supplementary Figure 11. [^{68}Ga]NODA-GA-R$_0$1-MG Dynamic PET Study of Tracer Pharmacokinetics of Patients Imaged at PUMC Hospital, China. (A) High uptake organs including the small intestines (SI), stomach (St), kidneys (Kd) and bladder (Bl) are quantified by ROI analysis from 0.4 to 60.37 minutes after injection. (B) Relatively lower uptake organs are quantified by ROI analysis at the same timepoints. Organs include the: brain (Br), pituitary gland (Pt), breast (Brs), lower large intestine (LLI), upper large intestine (ULI), heart wall (HW), heart contents (HC), liver (Lv), lung (Ln), muscle (Ms), pancreas (Pn), red marrow (RM), osteogenic cells (Bn), skin (Sk), spleen (Sp), and, whole body (WB). Source Data are provided in a Source Data File.

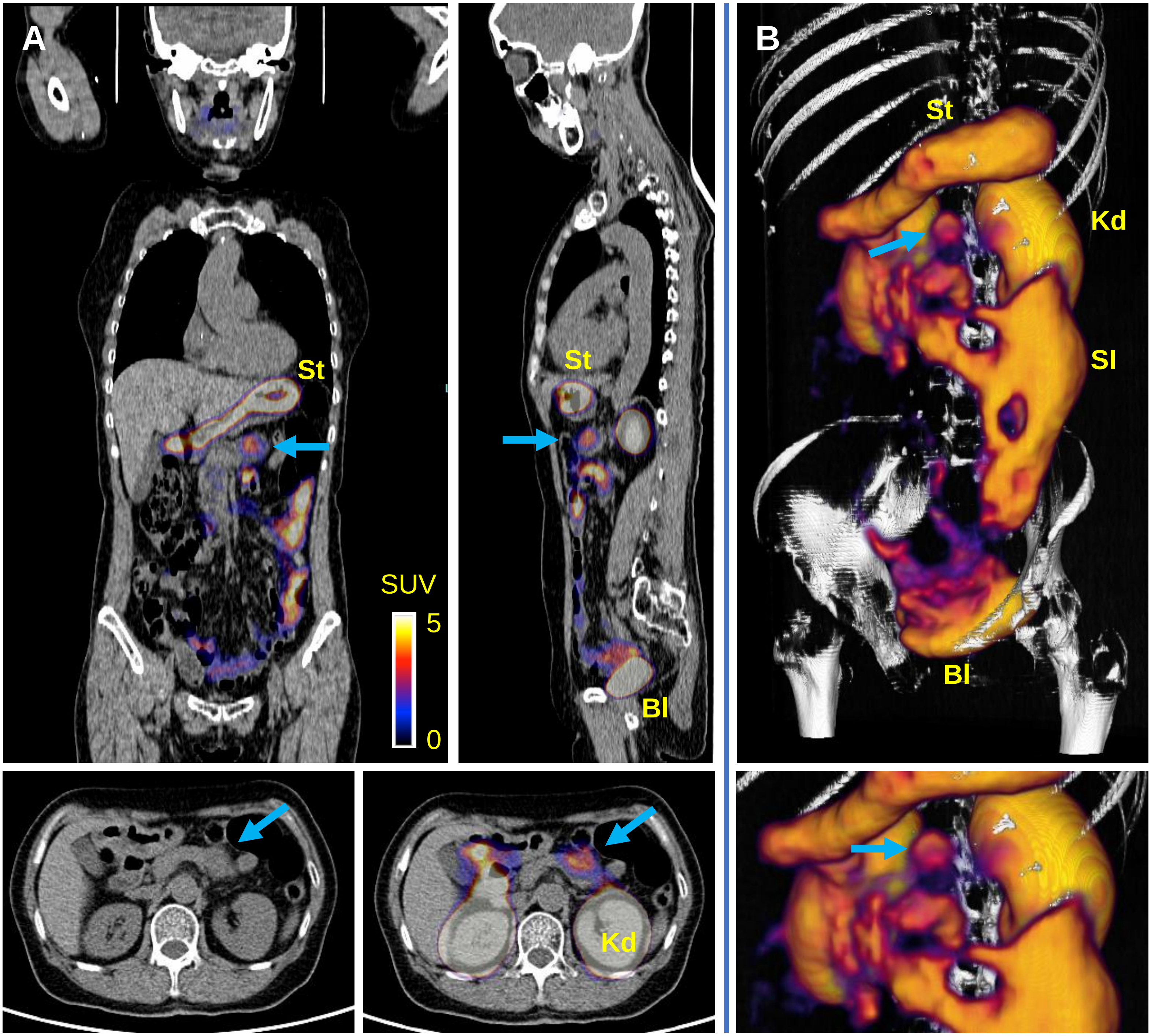

Supplementary Figure 12. [^{68}Ga]NODA-GA-R$_0$1-MG PET/CT Scan, Pancreatic Cancer Patient Imaged at Peking Union Medical College Hospital, China. (A) PET/CT Images of a 59 year old patient diagnosed with moderately differentiated pancreatic ductal adenocarcinoma. Uptake is observed in the tail of the pancreas (SUV$_{mean}$ = 4.3) as indicated by the blue arrow. CT image shows enlargement of the tail of the pancreas. (B) Volume rendered PET/CT images show the homogeneous uptake of the tracer in the tumor. Images were acquired ~50 minutes after intravenous injection of 2.2 mCi of [^{68}Ga]NODA-GA-R$_0$1-MG. Off-target uptake of the tracer is seen in the kidneys and upper gut. IHC analysis for integrin $\alpha_v\beta_6$ was inconclusive for this patient and additional studies are underway.

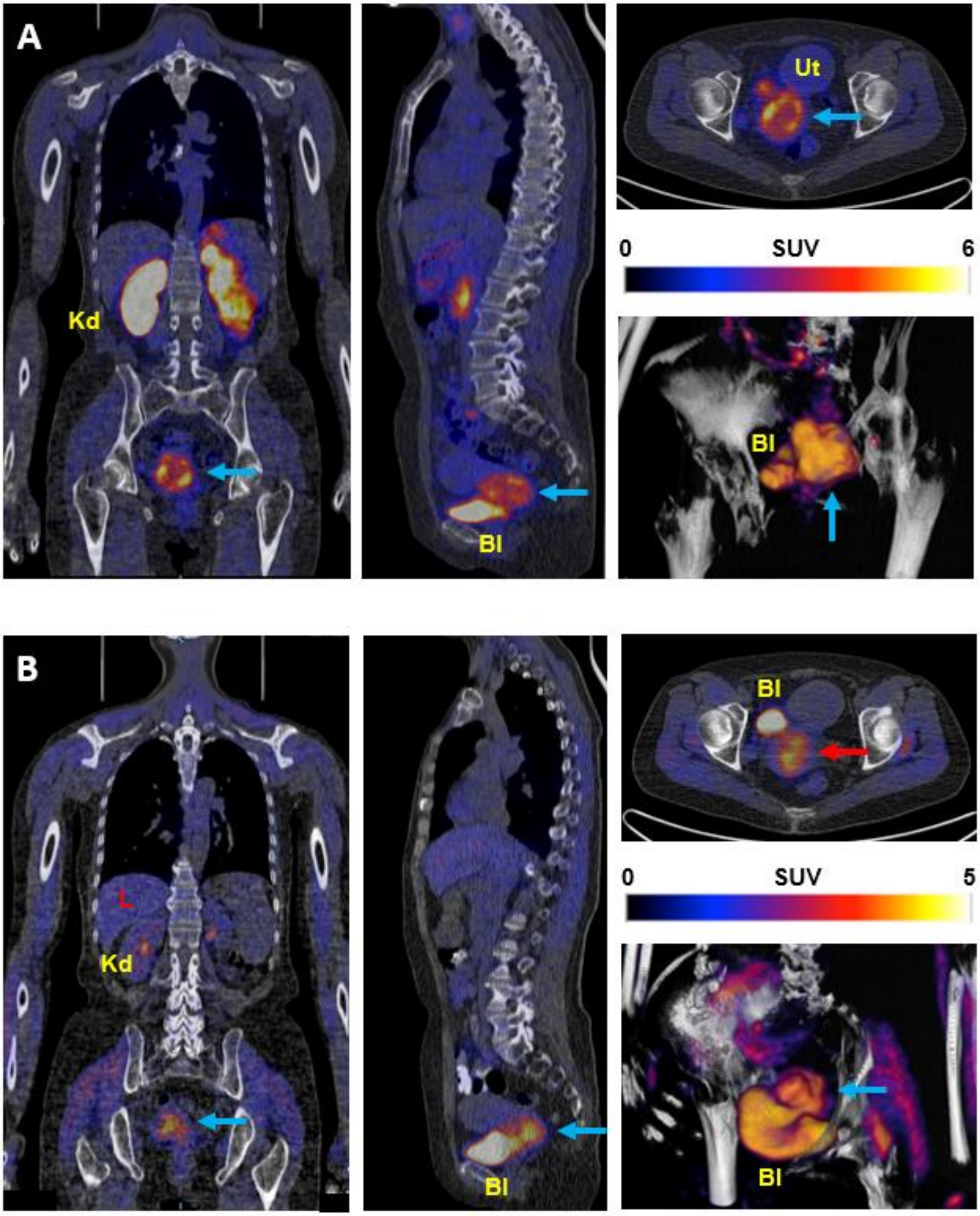

Supplementary Figure 13. [^{68}Ga]NODA-GA-R$_0$1-MG PET/CT Scan and [^{18}F]FDG PET/CT Scan, Cervical Cancer Patient Imaged at Peking Union Medical College Hospital, China. (A) PET/CT Images of a 54 year old patient diagnosed with well differentiated squamous cell carcinoma of the cervix. The bottom-right panel shows volume rendered PET/CT images that demonstrate good tracer uptake in the tumor as indicated by the cyan arrow (SUV$_{mean}$ = 4.5). Images were acquired ~30 minutes after intravenous injection of 2.1mCi of [^{68}Ga]NODA-GA-R$_0$1-MG. Off-target uptake of the tracer is seen in the kidneys and upper gut. (B) For comparison, [^{18}F]FDG PET/CT imaging shows tumor uptake of SUV$_{mean}$ = 4.1 in the same patient.

A

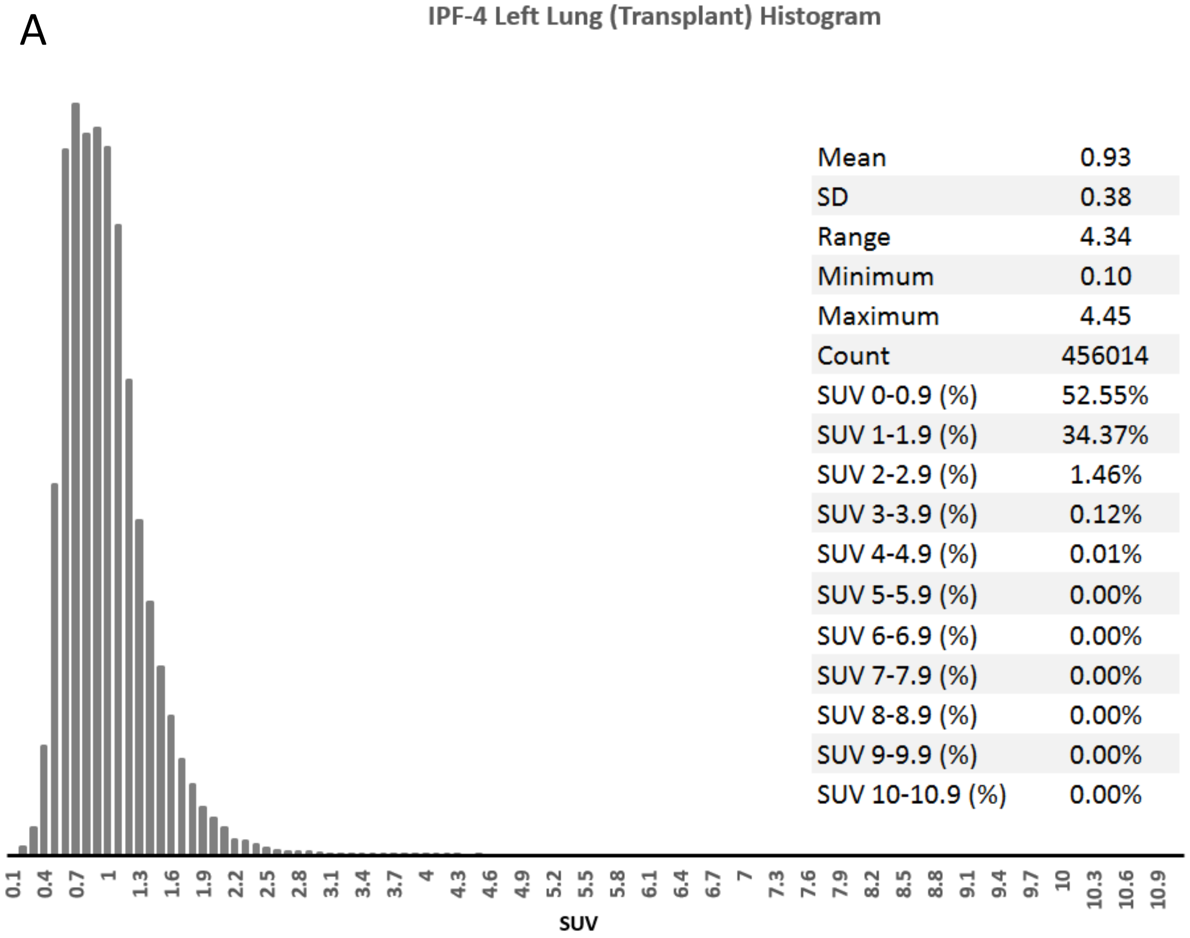
IPF-4 Left Lung (Transplant) Histogram
Mean 0.93
SD 0.38
Range 4.34
Minimum 0.10
Maximum 4.45
Count 456014
SUV 0-0.9 (%) 52.55%
SUV 1-1.9 (%) 34.37%
SUV 2-2.9 (%) 1.46%
SUV 3-3.9 (%) 0.12%
SUV 4-4.9 (%) 0.01%
SUV 5-5.9 (%) 0.00%
SUV 6-6.9 (%) 0.00%
SUV 7-7.9 (%) 0.00%
SUV 8-8.9 (%) 0.00%
SUV 9-9.9 (%) 0.00%
SUV 10-10.9 (%) 0.00%
0.1 0.4 0.7 1 1.3 1.6 1.9 2.2 2.5 2.8 3.1 3.4 3.7 4 4.3 4.6 4.9 5.2 5.5 5.8 6.1 6.4 6.7 7 7.3 7.6 7.9 8.2 8.5 8.8 9.1 9.4 9.7 10 10.3 10.6 10.9
SUV

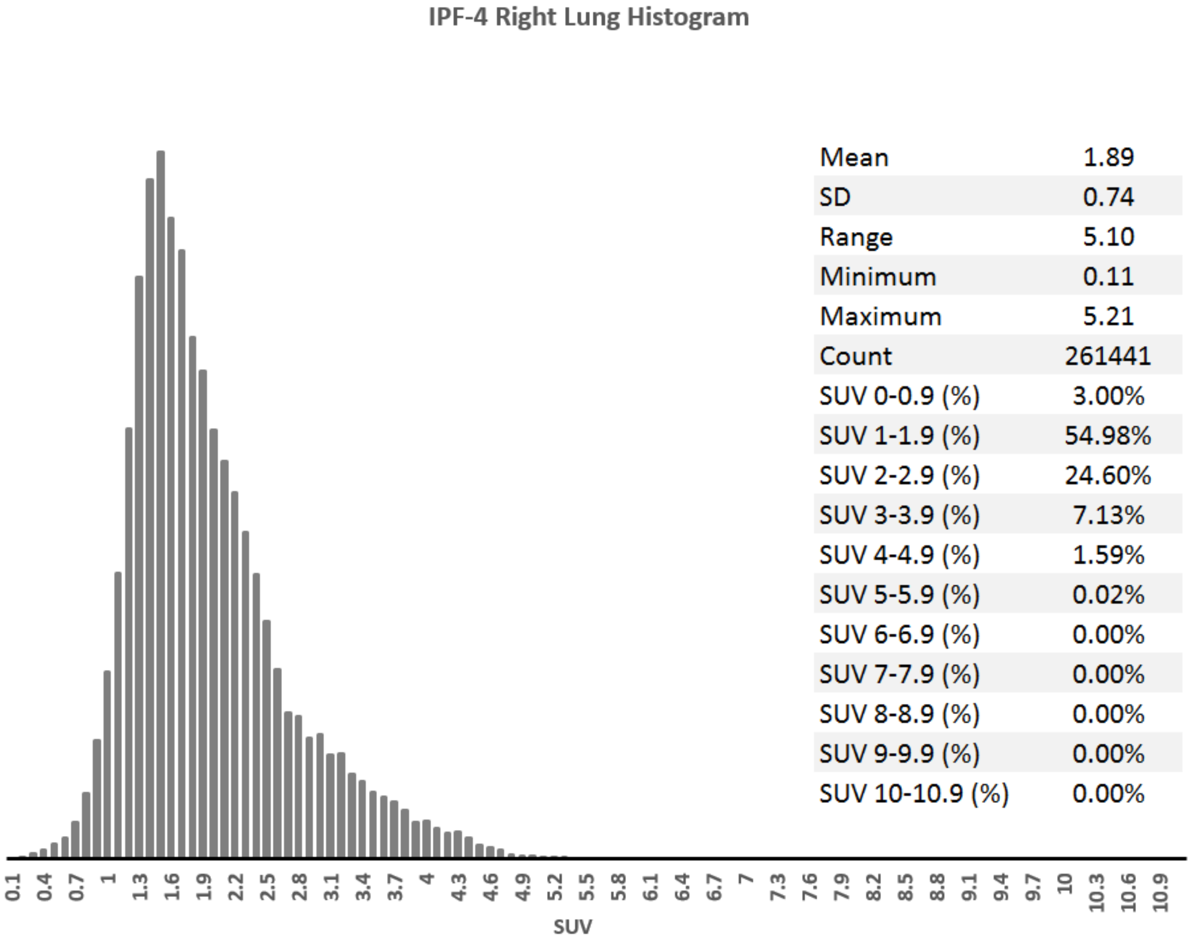
IPF-4 Right Lung Histogram
Mean 1.89
SD 0.74
Range 5.10
Minimum 0.11
Maximum 5.21
Count 261441
SUV 0-0.9 (%) 3.00%
SUV 1-1.9 (%) 54.98%
SUV 2-2.9 (%) 24.60%
SUV 3-3.9 (%) 7.13%
SUV 4-4.9 (%) 1.59%
SUV 5-5.9 (%) 0.02%
SUV 6-6.9 (%) 0.00%
SUV 7-7.9 (%) 0.00%
SUV 8-8.9 (%) 0.00%
SUV 9-9.9 (%) 0.00%
SUV 10-10.9 (%) 0.00%
0.1 0.4 0.7 1 1.3 1.6 1.9 2.2 2.5 2.8 3.1 3.4 3.7 4 4.3 4.6 4.9 5.2 5.5 5.8 6.1 6.4 6.7 7 7.3 7.6 7.9 8.2 8.5 8.8 9.1 9.4 9.7 10 10.3 10.6 10.9
SUV

B

IPF-5 Left Lung Histogram

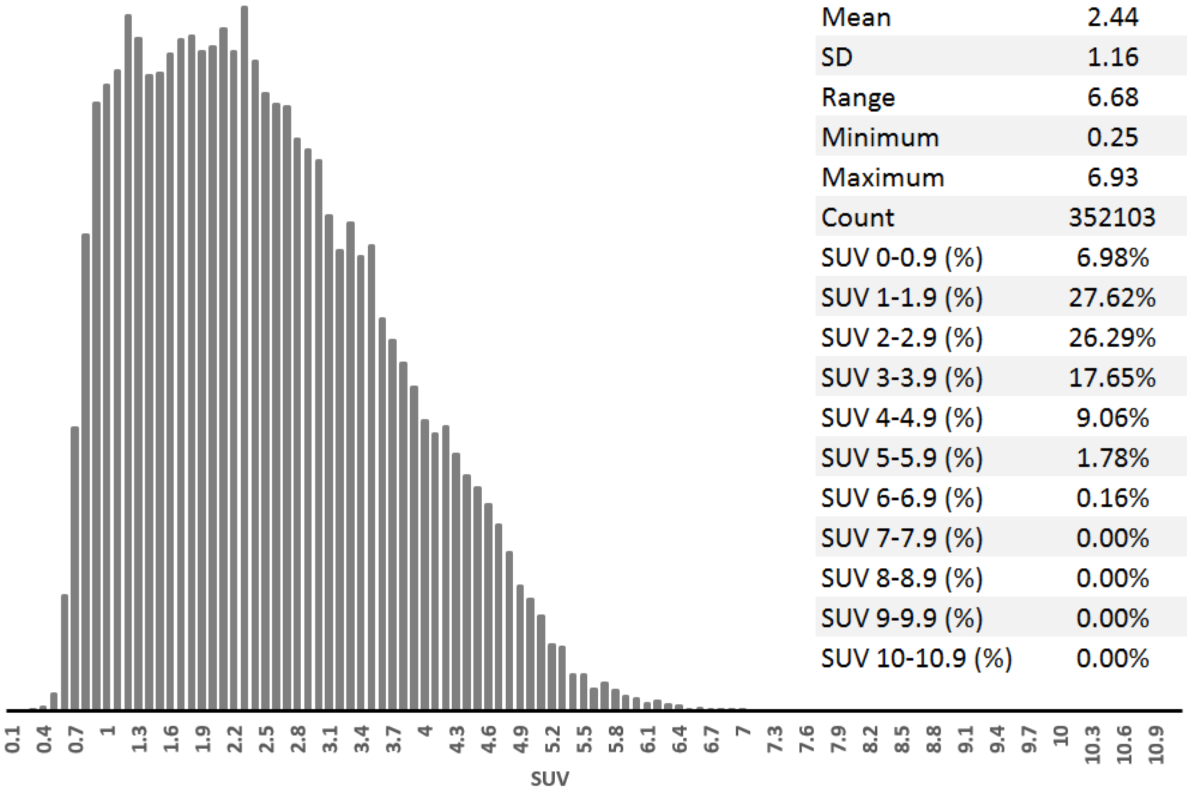

Mean	2.44
SD	1.16
Range	6.68
Minimum	0.25
Maximum	6.93
Count	352103
SUV 0-0.9 (%)	6.98%
SUV 1-1.9 (%)	27.62%
SUV 2-2.9 (%)	26.29%
SUV 3-3.9 (%)	17.65%
SUV 4-4.9 (%)	9.06%
SUV 5-5.9 (%)	1.78%
SUV 6-6.9 (%)	0.16%
SUV 7-7.9 (%)	0.00%
SUV 8-8.9 (%)	0.00%
SUV 9-9.9 (%)	0.00%
SUV 10-10.9 (%)	0.00%

IPF-5 Right Lung Histogram

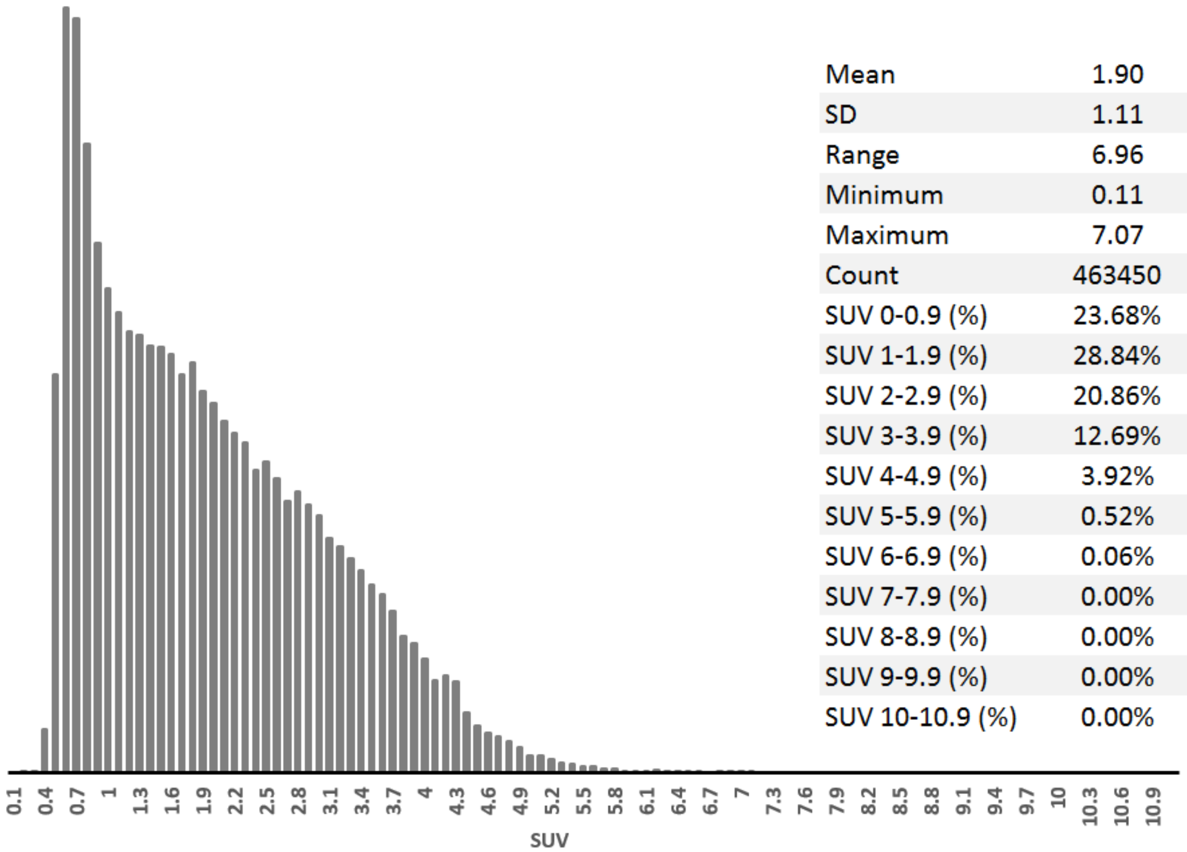

Mean	1.90
SD	1.11
Range	6.96
Minimum	0.11
Maximum	7.07
Count	463450
SUV 0-0.9 (%)	23.68%
SUV 1-1.9 (%)	28.84%
SUV 2-2.9 (%)	20.86%
SUV 3-3.9 (%)	12.69%
SUV 4-4.9 (%)	3.92%
SUV 5-5.9 (%)	0.52%
SUV 6-6.9 (%)	0.06%
SUV 7-7.9 (%)	0.00%
SUV 8-8.9 (%)	0.00%
SUV 9-9.9 (%)	0.00%
SUV 10-10.9 (%)	0.00%

Supplementary Figure 14. Representative SUV-Histograms of the Lungs of patients IPF-4 (A) and IPF-5 (B). The charts show the distribution of SUVs at 1 hr after administration of [^{18}F]FP-$R_0$1-MG-F2 with the bin size set to increments of 0.1 SUV unit. The summary table includes SUV metrics for the given number of voxels used in the analysis. The initial histogram bins were consolidated into larger bins of ~1 SUV unit and expressed as a percent of the total number of counts recorded in the lung. Source data are provided in a Source Data File.

Name	Expected Mass (Da)	Observed Mass (Da)
$R_0$1	3902.8	3903
DOTA-$R_0$1	4289.9	4289
$R_0$1-MR	3929.6	3929
DOTA-$R_0$1-MR	4317.0	4318
$R_0$1-MG	3831.5	3830
DOTA-$R_0$1-MG	4218.9	4219
NODA-GA-$R_0$1-MG	4189.8	4189
[^{19}F]FP-$R_0$1-MG-F1	3905.6	3906
[^{19}F]FP-$R_0$1-MG-F2	3905.6	3906

Supplementary Table 1. MALDI-MS of Cystine Knot Peptides and their Derivatives. The expected and observed average [M+H] for the oxidized/folded $R_0$1 peptide variants used in this study.

PET Tracer	Timepoint	Tumor	Liver	Kidney	Muscle	T:M Ratio
			(Average % Injected Dose / gram ± S.D.)			
$[^{64}Cu]$DOTA-$R_0$1	1 hr	3.07 ± 0.17	2.26 ± 0.11	30.30 ± 2.33	0.43 ± 0.01	7.09 ± 0.27
	2 hr	2.91 ± 0.05	2.23 ± 0.44	29.69 ± 4.31	0.41 ± 0.01	7.09 ± 0.02
	4 hr	2.43 ± 0.45	2.63 ± 0.74	27.70 ± 3.64	0.41 ± 0.01	6.00 ± 1.03
	24 hr	2.06 ± 0.40	3.30 ± 0.37	12.95 ± 3.62	0.38 ± 0.01	5.48 ± 0.95
$[^{64}Cu]$DOTA-$R_0$1-MR	1 hr	3.45 ± 0.34	4.63 ± 0.09	89.95 ± 3.64	0.54 ± 0.00	6.43 ± 0.58
	2 hr	3.44 ± 0.49	3.95 ± 0.28	91.62 ± 2.91	0.52 ± 0.00	6.64 ± 0.90
	4 hr	3.26 ± 0.65	3.99 ± 0.48	89.84 ± 12.58	0.47 ± 0.00	6.93 ± 1.38
	24 hr	3.44 ± 0.20	7.27 ± 0.36	40.34 ± 4.13	0.52 ± 0.00	6.67 ± 0.36
$[^{64}Cu]$DOTA-$R_0$1-MG	1 hr	2.74 ± 0.35	2.14 ± 0.38	28.35 ± 2.61	0.47 ± 0.00	5.80 ± 0.72
	2 hr	2.17 ± 0.35	2.27 ± 0.12	29.34 ± 2.42	0.40 ± 0.00	5.43 ± 0.86
	4 hr	2.11 ± 0.33	2.33 ± 0.10	27.12 ± 3.87	0.35 ± 0.03	6.06 ± 0.95
	24 hr	2.49 ± 0.18	3.89 ± 0.50	11.83 ± 1.21	0.40 ± 0.00	6.29 ± 0.43

Supplementary Table 2. Comparative Biodistribution. One hour to 24 hour biodistribution data of the tumor, kidney, liver and tumor-to-muscle ratios corresponding to the PET images of the 3 PET tracers shown in Figure 4 (above, first column). Source data are provided in a Source Data File.

Body Component	Integrin αvβ6 (+)		Integrin αvβ6 (-)	
	0.5 hr	2 hr	0.5 hr	2 hr
	(average % Injected Dose/ gram ± S.D.)			
Tumor	3.56 ± 0.33	3.07 ± 0.70	0.48 ± 0.02	0.30 ± 0.11
Blood	1.16 ± 0.15	0.53 ± 0.20	0.89 ± 0.09	0.33 ± 0.06
Heart	0.94 ± 0.07	0.67 ± 0.13	0.80 ± 0.24	0.63 ± 0.11
Lungs	3.47 ± 0.36	3.28 ± 0.42	3.85 ± 0.29	3.49 ± 0.17
Stomach	4.42 ± 0.81	5.23 ± 1.62	4.31 ± 0.26	5.41 ± 0.85
Intestine	3.28 ± 0.45	3.95 ± 1.12	3.08 ± 0.07	3.33 ± 0.71
Liver	0.60 ± 0.02	0.37 ± 0.08	0.55 ± 0.10	0.37 ± 0.06
Pancreas	0.66 ± 0.05	0.44 ± 0.13	0.62 ± 0.00	0.44 ± 0.04
Spleen	0.63 ± 0.13	0.34 ± 0.06	0.64 ± 0.05	0.25 ± 0.07
Kidney	29.19 ± 4.69	13.12 ± 2.47	43.00 ± 3.33	15.23 ± 2.72
Brain	0.24 ± 0.11	0.21 ± 0.03	0.18 ± 0.04	0.20 ± 0.09
Muscle	0.90 ± 0.06	0.42 ± 0.19	0.73 ± 0.13	0.51 ± 0.08
Skin	1.69 ± 0.35	1.57 ± 0.06	1.78 ± 0.71	1.31 ± 0.12
Bone	0.73 ± 0.20	0.66 ± 0.52	0.46 ± 0.02	0.45 ± 0.29
Tail	4.14 ± 2.06	2.78 ± 2.05	4.45 ± 1.55	1.93 ± 1.10

Supplementary Table 3. [^{18}F]FP-R$_0$1-MG-F2 Biodistribution. Biodistribution of [^{18}F]FP-R$_0$1-MG-F2 corresponding to mouse models shown in Supplementary Figure 5 (above). Source data are provided in a Source Data File.

Volunteer No/Sex/Age(y)	Height (m)	Weight (kg)	Inj. Activity (mCi)	Inj. Mass (μg)	Normalized Inj. Mass (μg/kg)
1/M/50	1.79	93.0	8.12	16.3	0.18
2/F/46	1.65	68.0	7.34	15.7	0.23
3/M/21	1.87	68.9	8.12	11.1	0.16
4/F/51	1.57	53.1	5.25	6.5	0.09
5/M/28	1.80	72.1	8.58	12.8	0.24
Mean	1.74	71.0	7.48	12.5	0.18
Standard Deviation	0.12	14.3	1.32	4.0	0.06

Healthy Volunteers had a mean age of 39.2 ± 12.3 years old with a range of 21 to 50 years old.

Supplementary Table 4. Demographics and Injected [^{18}F]FP-$R_0$1-MG-F2 Dose Data of the Healthy Volunteers Imaged at Stanford University Nuclear Medicine Clinic, USA. Parameters include sex, age, height of the 5 healthy volunteers. Injected activity was the difference between the activity measured prior to injection and the residual left in the syringe after injection of the tracer. Injected masses were calculated based on the specific activity of the tracer per batch and decay corrected for the time at the end of synthesis.

5 Healthy Volunteers<br>Serum Laboratory Value		1 Day After Injection	2 Days After Injection	7 Days After Injection
Hematology				
	White Blood Cell Count (x10^3 cells/µL)	6.84 ± 1.43	6.38 ± 0.99	6.36 ± 1.24
	Red Blood Cell Count (x10^3 cells/µL)	4.87 ± 0.53	4.89 ± 0.58	5.05 ± 0.50
	Hemoglobin Concentration (g/dL)	14.10 ± 2.23	14.14 ± 2.35	14.64 ± 2.25
	Hematocrit Concentration (g/dL)	42.66 ± 6.11	43.00 ± 6.46	44.28 ± 5.92
	Corpuscular Volume (fL)	87.42 ± 6.61	87.66 ± 6.69	87.42 ± 6.64
	Corpuscular Hemoglobin Count (pg)	28.82 ± 2.73	28.82 ± 2.70	28.86 ± 2.76
	Corpuscular Hemoglobin Concentration (g/dL)	32.94 ± 0.95	32.88 ± 0.76	32.94 ± 1.25
	Red Cell Distribution Width (%)	13.90 ± 2.12	13.74 ± 2.05	13.88 ± 1.77
	Platelet Count (x10^3 cells/µL)	236.20 ± 40.47	220.40 ± 36.12	224.40 ± 33.44
Chemistry				
	Sodium Concentration (mmol/L)	138.60 ± 2.97	138.20 ± 2.28	138.6 ± 1.52
	Potassium Concentration (mmol/L)	4.02 ± 0.32	3.98 ± 0.25	4.10 ± 0.23
	Calcium Concentration (mmol/L)	9.18 ± 0.44	9.06 ± 0.25	9.24 ± 0.47
	Chloride Concentration (mmol/L)	102.40 ± 2.19	103.20 ± 1.48	101.60 ± 2.30
	Anion Gap (mEq/L)	6.40 ± 0.89	6.60 ± 1.95	8.40 ± 1.67
	CO_2 Level (mmol/L)	29.80 ± 1.10	28.40 ± 2.19	28.60 ± 2.07
	Urea Nitrogen Level (mg/dL)^2	15.00 ± 5.70	15.60 ± 5.13	14.00 ± 3.08
	Creatinine Concentration (mg/dL)	0.93 ± 0.10	0.89 ± 0.08	0.96 ± 0.16
	Glucose (mg/dL)	103.80 ± 14.34	102.80 ± 30.83	107.40 ± 29.67

Data are mean value ± standard deviations.

Supplementary Table 5. Clinical Laboratory Testing Results of the 5 healthy volunteers Imaged at Stanford University Nuclear Medicine Clinic, USA. Results include the complete blood counts (CBC) and basic metabolite panel (BMP) from blood samples obtained 1, 2 and 7 days after injection with [^{18}F]FP-$R_0$1-MG-F2.

5 Healthy Volunteers<br>Vital Signs	Baseline	5 minutes	10 minutes	15 minutes	60 minutes	120 minutes	Day 2	Day 7
Heart Rate (bpm)	77.60 ± 8.47	73.40 ± 6.88	73.20 ± 5.36	73.00 ± 7.42	72.40 ± 5.55	70.40 ± 5.94	69.40 ± 7.70	72.20 ± 8.58
Oximetry (SO_2)	99.00 ± 1.41	99.20 ± 1.79	99.40 ± 1.34	99.40 ± 1.34	99.60 ± 0.89	100.00 ± 0.00	99.80 ± 0.45	98.40 ± 3.58
Temperature (^{0} F)	97.74 ± 1.43	97.86 ± 1.63	98.24± 0.49	98.40 ± 0.57	97.96 ± 0.47	98.20 ± 0.22	98.24 ± 0.32	98.30 ± 0.49
Systolic Blood Pressure (mmHg)	118.80 ± 12.62	118.40 ± 9.07	118.40 ± 6.84	116.40 ± 6.66	118.60 ± 12.10	119.20 ± 10.64	122.60 ± 11.26	116.60 ± 12.10
Diastolic Blood Pressure (mmHg)	73.00 ± 9.33	73.00 ± 9.80	70.20 ± 10.59	69.00 ± 10.42	70.80 ± 13.68	73.40 ± 13.45	75.20 ± 10.35	71.80 ± 11.56

Data are mean value ± standard deviations.

Supplementary Table 6. Vital Signs of the 5 Healthy Volunteers Imaged at Stanford University Nuclear Medicine Clinic, USA. Vital signs recorded before injection of PET tracer, and 5, 10, 15, 60, 120 minutes, 2 days and 7 days after injection with [^{18}F]FP-$R_0$1-MG-F2.

Body Component	5 min	60 min	120 min
		(average SUV_{mean} ± S.D.)	
Brain	0.38 ± 0.21	0.17 ± 0.02	0.21 ± 0.03
Pituitary Gland	* 2.08 ± n.a.	3.83 ± 0.94	3.55 ± 1.45
Heart Wall	1.73 ± 0.80	0.71 ± 0.19	0.71 ± 0.28
Heart Contents	3.51 ± 1.89	0.77 ± 0.19	** 0.71 ± 0.15
Lungs	0.96 ± 0.41	0.76 ± 0.33	0.79 ± 0.36
Stomach	6.33 ± 2.39	9.81 ± 1.97	8.13 ± 3.78
Small Intestine	7.15 ± 2.81	8.51 ± 1.62	7.34 ± 1.14
Upper Large Intestine	3.18 ± 1.67	3.67 ± 1.65	2.24 ± 0.64
Lower Large Intestine	2.45 ± 0.42	2.82 ± 0.93	2.13 ± 0.59
Pancreas	2.45 ± 1.58	1.89 ± 0.78	1.01 ± 0.24
Liver	2.27 ± 0.21	0.62 ± 0.17	0.42 ± 0.08
Spleen	1.90 ± 1.06	0.96 ± 0.42	0.88 ± 0.53
Right Kidney	16.35 ± 5.91	12.44 ± 4.42	8.00 ± 3.32
Urinary Bladder Contents	1.07 ± 0.63	4.79 ± 2.18	3.66 ± 1.72
Muscle-Leg	0.83 ± 0.12	1.52 ± 0.35	1.50 ± 0.25
Red Marrow	0.46 ± 0.29	0.34 ± 0.12	0.17 ± 0.13
Osteogenic Cells	0.81 ± 0.44	0.44 ± 0.18	0.41 ± 0.07
Skin	0.19 ± 0.21	0.18 ± 0.04	0.24 ± 0.05
Whole Body to Knees	0.74 ± 0.34	0.90 ± 0.46	0.81 ± 0.10

Supplementary Table 7. **[^{18}F]FP-R$_0$1-MG-F2 Biodistribution.** The average (5 healthy volunteers) SUV_{mean} ± standard deviation (SD, n=5, except for * where n=1, and ** where n=4) of major body organs or tissues. Source data are provided in a Source Data file.

Timepoint	Whole Blood			Serum		
	(average normalized CPM ± S.D.)					
Pre-inj	0.05%	±	0.02%	0.04%	±	0.03%
1 min	40.67%	±	23.80%	22.69%	±	12.63%
3 min	100.00%		(norm)	60.92%		7.65%
* 5 min	69.65%	±	18.67%	44.77%	±	11.20%
* 10 min	42.55%	±	12.46%	28.41%	±	8.79%
30 min	12.90%	±	2.88%	8.74%	±	1.82%
60 min	8.62%	±	2.72%	5.45%	±	2.05%
90 min	7.22%	±	2.08%	4.67%	±	1.38%
120 min	6.02%	±	1.57%	3.57%	±	1.06%
150 min	5.79%	±	2.15%	3.51%	±	1.36%
180 min	5.37%	±	1.98%	2.92%	±	1.25%

Supplementary Table 8. Kinetics of [^{18}F]FP-R$_0$1-MG-F2 in Blood. Radioactivity in blood was measured as function of time. Plasma was separated from whole blood; both were measured by gamma counting. Decay-corrected counts per minute were normalized to the highest whole blood measurement at the 3-minute time point. Data represent the mean ± the SD (n=5**,** except for * where n=4). Source data are provided in a Source Data file.

Target Organ	mSv/MBq		rem/mCi	
	Adult Male	Adult Female	Adult Male	Adult Female
Adrenals	0.0069	0.0088	0.0257	0.0325
Brain	0.0022	0.0025	0.0080	0.0091
Breasts	0.0029	0.0036	0.0108	0.0134
Gallbladder Wall	0.0080	0.0095	0.0294	0.0351
LLI Wall*	0.0071	0.0084	0.0262	0.0309
Small Intestine	0.0298	0.0358	0.1100	0.1320
Stomach Wall	0.0176	0.0207	0.0650	0.0765
ULI Wall*	0.0095	0.0114	0.0351	0.0421
Heart Wall	0.0076	0.0092	0.0281	0.0342
Kidneys	0.0446	0.0490	0.1650	0.1810
Liver	0.0104	0.0136	0.0387	0.0501
Lungs	0.0076	0.0098	0.0281	0.0363
Muscle	0.0090	0.0130	0.0335	0.0481
Ovaries	0.0083	0.0101	0.0307	0.0375
Pancreas	0.0161	0.0187	0.0597	0.0693
Red Marrow	0.0050	0.0060	0.0184	0.0223
Osteogenic Cells	0.0052	0.0066	0.0194	0.0243
Skin	0.0031	0.0038	0.0115	0.0139
Spleen	0.0065	0.0081	0.0241	0.0299
Testes	0.0042	n.a.	0.0157	n.a.
Thymus	0.0047	0.0058	0.0173	0.0215
Thyroid	0.0044	0.0047	0.0163	0.0176
Urinary Bladder Wall	0.0120	0.0151	0.0446	0.0559
Uterus	0.0081	0.0099	0.0300	0.0367
Total Body	0.0071	0.0086	0.0264	0.0317
Effective Dose Equivalent	0.0115	0.0137	0.0426	0.0506
Effective Dose	0.0090	0.0109	0.0335	0.0402

* LLI and ULI = Lower and Upper Large Intestines

Supplementary Table 9. Dosimetry in 5 Healthy Human Volunteers Imaged at Stanford University Nuclear Medicine Clinic, USA. Dosimetry values were based on the average percent injected activity (%IA) in the kidney, stomach, small intestine, bladder, pancreas, liver, lung, heart, muscle and whole body remainder. The average area under the curve (AUC) of the %IA was determined by ROI analysis of static [^{18}F]FP-R$_0$1-MG-F2 PET scans acquired immediately upon tracer injection, 1 hour and 2 hours after injection. These values were entered into OLINDA (Stabin et al., J. Nucl. Med, 2005) to determine the radiation dosimetry to each of the organs listed in the table. The kidneys were determined to be the dose limiting organ at 0.0490 mSv/MBq or 0.1650 rem/mCi in an adult male. Next, the small intestines of an adult male would receive 0.0298 mSv/MBq or 0.1100 rem/mCi. Using the value for the kidneys of an adult male, the maximum allowable exposure to [^{18}F]FP-R$_0$1-MG-F2 may be 30.30 mCi/yr or 1121.21 MBq/yr based on the 5 rem limit stated in the federal guidelines.

Patient No/Sex/Age(y)	Height (m)	Weight (kg)	Injected Activity (mCi) Knottin/FDG	Injected Mass (μg) Knottin/FDG	Normalized Inj. Mass (μg/kg) Knottin/FDG
1/F/61	1.70	102.0	5.05/10.2	13.1/<1.3	0.13/<12

Supplementary Table 10. Demographics and [^{18}F]FP-R$_0$1-MG-F2 Dose Data of the Pancreatic Cancer Patient Imaged at Stanford University Nuclear Medicine Clinic, USA. Parameters include sex, age, height of the study subject. Injected activity is the difference between the activity measured prior to injection and the residual left in the syringe after injection of the tracer. Injected masses are calculated based on the specific activity of the tracer per batch and decay corrected for the time elapsed from the end of synthesis to intravenous injection.

Patient No/Sex/Age(y)	Cancer	Weight (kg)	Inj. Activity (mCi)
1/F/54	Cervical	60	2.1
2/F/47	Cervical	63	2.1
3/F/69	Cervical	60	3.1
4/M/66	Pancreatic	65	1.5
5/F/59	Pancreatic	50	2.2
6/M/70	Lung	60	2.6
Mean		59.7	2.3
Standard Deviation		5.2	0.5

Study participants had a mean age of 60.8 ± 8.4 years old with a range of 47 to 70 years old.

Supplementary Table 11. Demographics and [^{68}Ga]NODA-GA-R$_0$1-MG Dosage Information of Cancer Patients Imaged at Peking Union Medical College Hospital, China. Parameters include sex, age, height of 6 cancer patients. Injected activity was the total amount of radioactivity injected into the study subject.

Target Organ	mSv/MBq			
	Bladder Voiding Interval			
	n.a.	0.5 hr	1 hr	2 hr
Adrenals	0.0031	0.0031	0.0031	0.0031
Brain	0.0003	0.0003	0.0003	0.0003
Breasts	0.0006	0.0006	0.0006	0.0006
Gallbladder Wall	0.0034	0.0034	0.0034	0.0034
LLI Wall	0.0032	0.0027	0.0029	0.0032
Small Intestine	0.0680	0.0678	0.0679	0.0680
Stomach Wall	0.0088	0.0087	0.0087	0.0088
ULI Wall	0.0056	0.0054	0.0055	0.0056
Heart Wall	0.0010	0.0010	0.0010	0.0010
Kidneys	0.1810	0.1810	0.1810	0.1810
Liver	0.0068	0.0068	0.0068	0.0068
Lungs	0.0047	0.0047	0.0047	0.0047
Muscle	0.0020	0.0019	0.0020	0.0020
Ovaries	0.0041	0.0036	0.0038	0.0041
Pancreas	0.0028	0.0028	0.0028	0.0028
Red Marrow	0.0017	0.0016	0.0016	0.0017
Osteogenic Cells	0.0011	0.0011	0.0011	0.0011
Skin	0.0007	0.0007	0.0007	0.0007
Spleen	0.0029	0.0029	0.0029	0.0029
Testes	0.0011	0.0007	0.0008	0.0011
Thymus	0.0006	0.0006	0.0006	0.0006
Thyroid	0.0005	0.0005	0.0005	0.0005
Urinary Bladder Wall	0.0657	0.0191	0.0362	0.0654
Uterus	0.0047	0.0035	0.0039	0.0047
Total Body	0.0032	0.0031	0.0032	0.0032
Effective Dose Equivalent	0.0217	0.0188	0.0199	0.0217
Effective Dose	0.0115	0.0090	0.0099	0.0115

* LLI and ULI = Lower and Upper Large Intestines

Target Organ	rem/mCi			
	Bladder Voiding Interval			
	n.a.	0.5 hr	1 hr	2 hr
Adrenals	0.0116	0.0115	0.0115	0.0116
Brain	0.0012	0.0012	0.0012	0.0012
Breasts	0.0021	0.0021	0.0021	0.0021
Gallbladder Wall	0.0126	0.0125	0.0125	0.0126
LLI Wall	0.0118	0.0099	0.0106	0.0118
Small Intestine	0.2520	0.2510	0.2510	0.2520
Stomach Wall	0.0324	0.0323	0.0323	0.0324
ULI Wall	0.0206	0.0200	0.0202	0.0206
Heart Wall	0.0037	0.0037	0.0037	0.0037
Kidneys	0.6690	0.6690	0.6690	0.6690
Liver	0.0252	0.0251	0.0252	0.0252
Lungs	0.0175	0.0175	0.0175	0.0175
Muscle	0.0075	0.0070	0.0072	0.0075
Ovaries	0.0152	0.0134	0.0141	0.0152
Pancreas	0.0104	0.0104	0.0104	0.0104
Red Marrow	0.0062	0.0059	0.0060	0.0062
Osteogenic Cells	0.0042	0.0041	0.0041	0.0042
Skin	0.0026	0.0024	0.0025	0.0026
Spleen	0.0108	0.0107	0.0107	0.0108
Testes	0.0039	0.0026	0.0031	0.0039
Thymus	0.0024	0.0024	0.0024	0.0024
Thyroid	0.0019	0.0018	0.0019	0.0019
Urinary Bladder Wall	0.2430	0.0706	0.1340	0.2420
Uterus	0.0172	0.0130	0.0145	0.0172
Total Body	0.0120	0.0115	0.0117	0.0120
Effective Dose Equivalent	0.0804	0.0695	0.0735	0.0803
Effective Dose	0.0424	0.0331	0.0365	0.0424

* LLI and ULI = Lower and Upper Large Intestines

Supplementary Table 12. Dosimetry based on a dynamic PET scan of a Cervical Cancer Patient. The average area under the curve of the percent injected activity was determined by ROI analysis of a [^{68}Ga]NODA-GA-R$_0$1-MG dynamic PET scan (Supplementary Figure 10). AUCs included the kidney, stomach, small intestine, bladder, pancreas, liver, lung, heart, muscle and whole body remainder. These values were entered into OLINDA to determine the radiation dosimetry to each of the organs listed in the table. Several bladder voiding intervals were explored. The dose limiting organ would be the kidney subject to exposure levels of 0.1810 mSv/MBq or 0.6690 rem/mCi and 0.181 rem/mCi. Next, the small intestines would receive 0.0680 mSv/MBq or 0.2520 rem/mCi. Using these values, the maximum allowable exposure to [^{68}Ga]NODA-GA-R$_0$1-MG is calculated to be 7.5 mCi/yr or 276.5 MBq/yr based on the federal guideline of 5 rem per year maximum allowable exposure.

	Baseline-20				transplant					bird-exposure												
Stubject ID	HV-5 (R)	HV-5 (L)	HV-3 (R)	HV-3 (L)	*IPF-4 (L)**	HV-4 (R)	HV-4 (L)	HV-2 (R)	HV-2 (L)	HV-1 (L)	HV-1 (R)	IPF-3 (R)	IPF-3 (L)	IPF-2 (R)	IPF-2 (L)	IPF-4 (R)	IPF-5 (L)	IPF-5 (R)	IPF-6 (L)	IPF-6 (R)	IPF-1 (L)	IPF-1 (R)
Age	26	26	20	20	*72*	49	49	44	44	48	48	75	75	73	73	72	72	72	72	72	71	71
Gender	M	M	M	M	*M*	F	F	F	F	M	M	M	M	F	F	M	M	M	M	M	M	M
Mean	0.51	0.56	0.74	0.79	*0.93*	0.94	1.00	1.02	1.07	1.43	1.40	1.12	1.12	1.57	1.97	1.89	2.44	1.90	2.15	1.96	2.43	3.79
SD	0.20	0.21	0.20	0.23	*0.38*	0.37	0.36	0.28	0.29	0.40	0.39	0.60	0.53	0.54	0.61	0.74	1.16	1.11	0.97	0.99	0.96	1.27
Range	2.15	1.64	1.78	1.95	*4.34*	2.52	2.07	2.59	2.90	3.13	3.64	4.87	4.75	3.77	4.22	5.10	6.68	6.96	7.00	8.40	8.66	10.16
Minimum	0.15	0.14	0.20	0.20	*0.10*	0.30	0.34	0.39	0.34	0.28	0.37	0.02	0.06	0.30	0.33	0.11	0.25	0.11	0.17	0.10	0.06	0.11
Maximum	2.29	1.78	1.99	2.16	*4.45*	2.82	2.41	2.97	3.24	3.41	4.01	4.89	4.82	4.07	4.55	5.21	6.93	7.07	7.17	8.50	8.71	10.26
Count	442383	360217	457031	387105	*456014*	290900	251342	266208	232940	312419	351610	621897	461261	498242	247798	261441	352103	463450	560374	503008	725282	506797
SUV 0-0.9 (%)	94.86%	92.67%	80.87%	73.62%	*52.55%*	58.90%	48.97%	39.69%	32.18%	7.14%	5.89%	42.19%	41.86%	6.90%	1.81%	3.00%	6.98%	23.68%	4.54%	11.80%	2.10%	0.96%
SUV 1-1.9 (%)	3.13%	3.99%	10.80%	16.50%	*34.37%*	29.63%	40.44%	44.68%	54.02%	74.16%	75.72%	39.39%	41.49%	62.17%	46.08%	54.98%	27.62%	28.84%	42.89%	39.14%	28.58%	6.10%
SUV 2-2.9 (%)	0.00%	0.00%	0.00%	0.02%	*1.46%*	1.65%	0.66%	0.31%	0.43%	8.85%	7.70%	8.19%	6.81%	18.25%	36.24%	24.60%	26.29%	20.86%	26.10%	26.06%	36.61%	16.24%
SUV 3-3.9 (%)	0.00%	0.00%	0.00%	0.00%	*0.12%*	0.00%	0.00%	0.00%	0.00%	0.04%	0.10%	0.66%	0.30%	1.45%	5.80%	7.13%	17.65%	12.69%	12.26%	10.00%	15.49%	25.10%
SUV 4-4.9 (%)	0.00%	0.00%	0.00%	0.00%	*0.01%*	0.00%	0.00%	0.00%	0.00%	0.00%	0.00%	0.05%	0.02%	0.01%	0.12%	1.59%	9.06%	3.92%	3.57%	3.07%	5.75%	25.77%
SUV 5-5.9 (%)	0.00%	0.00%	0.00%	0.00%	*0.00%*	0.00%	0.00%	0.00%	0.00%	0.00%	0.00%	0.00%	0.00%	0.00%	0.00%	0.02%	1.78%	0.52%	1.09%	0.60%	1.36%	12.39%
SUV 6-6.9 (%)	0.00%	0.00%	0.00%	0.00%	*0.00%*	0.00%	0.00%	0.00%	0.00%	0.00%	0.00%	0.00%	0.00%	0.00%	0.00%	0.00%	0.16%	0.06%	0.18%	0.04%	0.11%	2.93%
SUV 7-7.9 (%)	0.00%	0.00%	0.00%	0.00%	*0.00%*	0.00%	0.00%	0.00%	0.00%	0.00%	0.00%	0.00%	0.00%	0.00%	0.00%	0.00%	0.00%	0.00%	0.00%	0.01%	0.01%	0.49%
SUV 8-8.9 (%)	0.00%	0.00%	0.00%	0.00%	*0.00%*	0.00%	0.00%	0.00%	0.00%	0.00%	0.00%	0.00%	0.00%	0.00%	0.00%	0.00%	0.00%	0.00%	0.00%	0.00%	0.00%	0.06%
SUV 9-9.9 (%)	0.00%	0.00%	0.00%	0.00%	*0.00%*	0.00%	0.00%	0.00%	0.00%	0.00%	0.00%	0.00%	0.00%	0.00%	0.00%	0.00%	0.00%	0.00%	0.00%	0.00%	0.00%	0.06%
SUV 10-10.9 (%)	0.00%	0.00%	0.00%	0.00%	*0.00%*	0.00%	0.00%	0.00%	0.00%	0.00%	0.00%	0.00%	0.00%	0.00%	0.00%	0.00%	0.00%	0.00%	0.00%	0.00%	0.00%	0.00%

Supplementary Table 13. Integrin $\alpha_v\beta_6$ Fibrosis Spectrum for [^{18}F]FP-$R_0$1-MG-F2 at 1 hr After Injection. The distribution of lung radioactivity per voxel or SUVs were plotted as histograms and bundled into 11 equal bins. The percentages of SUVs in each of the bins, with respect to total lung counts, were arranged in ascending order in the heatmap. Each half-lung was treated independently of the contralateral half. The vertical dashed blue line separates healthy lungs in HV-1 to HV-5 and the transplanted left lung of IPF-4 (n=11) from the diseased lungs of IPF-1 to IPF-6 (n=11). This same line might occur at a hypothetical high-risk transition zone between subclinical (asymptomatic) and clinically-apparent disease. The colors green, yellow, orange and red on this heat map represent the lowest, low-mid, high-mid and the higher values, respectively. Source Data are provided as a Source Data File.
