# Supplementary material for: Evaluation of integrin αvβ6 cystine knot PET tracers to detect cancer and idiopathic pulmonary fibrosis
Source: Nat Commun. 2019 Oct 14;10:4673. doi: 10.1038/s41467-019-11863-w (PMC6791878; doi:10.1038/s41467-019-11863-w)
Supplement: Supplementary file 2 — Supplementary Tox Report [file 41467_2019_11863_MOESM2_ESM.pdf]

SoBran INC.

Motivated By Discovery

Final Study Report

14-Day Single Intravenous Dose Toxicity Study of [^{19}F]FP-R$_0$1-MG-F2 in Sprague Dawley Rats

Test Article

[^{19}F]FP-R$_0$1-MG-F2

Authors

Adrienne Edgell, B.S., CMAR, LATG
Grace McMonagle, B.S.

Study Completion Date

February 24, 2015

Performing Laboratory

SoBran Rangos Animal Facility
855 N. Wolfe Street, Suite 622
Baltimore, MD 21205

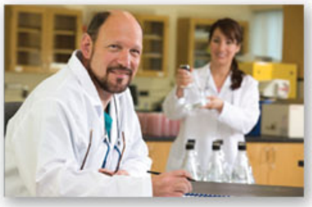
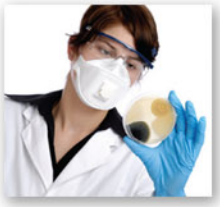
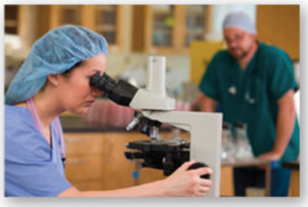
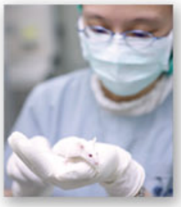

SoBran INC.

Motivated By Discovery

SoBran Study Number

SB-SU-003

Client

Stanford University
1201 Welch Road, Rm PS049
Stanford, CA 94305-5484

Sponsor

Frederick T. Chin, Ph.D.

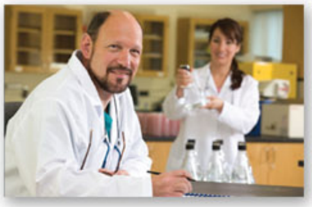

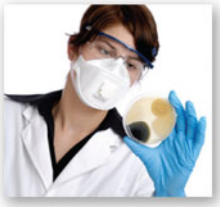

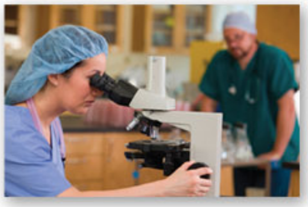

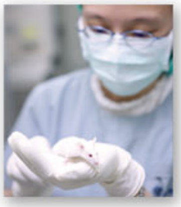

COMPLIANCE STATEMENT

14-Day Single Intravenous Dose Toxicity Study of [^{19}F]FP-$R_0$1-MG-F2 in Sprague Dawley Rats

The study was conducted in compliance with Food and Drug Administration Good Laboratory Practices as found in title 21 Code of Federal Regulations part 58 with the following exceptions:

> Dose formulation analysis for concentration and stability was not conducted for the test article formulation. Manufacturing of the vehicle components, ethanol and sterile saline (0.9% sodium chloride), were conducted in accordance with cGMP regulations and characterization conducted in accordance with USP regulations.
>
> All statistical analyses were performed with non-validated software (statistical package R version 3.1.2 (for ANOVA using the aov function) and the multcomp package for Dunnett's t-tests). The statistical analysis report was audited against the study data by SoBran's QAU.

There were no deviations from the aforementioned regulations that affected the quality or integrity of the study or the interpretations of the results in this report.

Signature: [signature] 2/24/15

Adrienne Edgell, BS, CMAR, LATG
Study Director

Date

QUALITY ASSURANCE STATEMENT

14-Day Single Intravenous Dose Toxicity Study of [^{19}F]FP-$R_0$1-MG-F2 in Sprague Dawley Rats

The SoBran Rangos Animal Facility is reviewed by SoBran's Quality Assurance Unit (QAU) on a monthly basis for quality assurance/quality control compliance. Individual studies are monitored according to the designated Good Laboratory Practices (GLP) status as found in title 21 Code of Federal Regulations part 58, the protocol, and SoBran's Standard Operating Procedures. All findings were reported to the Study Director and Study Management as indicated below.

Study Phase Audited	Date(s) Audited	Date Reported to Study Management	Date Reported to Study Director
Day 1 Test Article Formulation; Dose Administration	10/7/2014	10/15/2014	10/15/2014
Day 15 Blood Collection, Necropsy and Organ Weights	10/21/2014	10/23/2014	10/23/2014
Draft report Audit and Data Review	10/8/2014	10/9/2014	10/9/2014
Statistics Report Audit	10/8/2014	10/9/2014	10/9/2014
Final Report Post Audit	2/20/2015	2/20/2015	2/20/2015

All audit findings were addressed appropriately. This final report accurately describes the study methods and procedures used during the conduct of the study and the results were reported accurately in the raw data.

Signature: [signature] 2/24/15

Patricia Asah
SoBran Quality Assurance

Date

SIGNATURE PAGE

14-Day Single Intravenous Dose Toxicity Study of [^{19}F]FP-$R_0$1-MG-F2 in Sprague Dawley Rats

Signature: ______________________ 2/24/15
Adrienne Edgell, B.S., CMAR, LATG
Study Director
Date

Signature: ______________________ 2/24/15
Gregory Kelly, Ph.D.
Study Management
Date

Signature: ______________________ 2/24/15
Grace McMonagle, B.S.
Technical Writer
Date

Abbreviations

**>**	greater than
**<**	less than
**≥**	greater than or equal to
**≤**	less than or equal to
↑	increase
↓	decrease
♀	female
♂	male
**°**	degree
**%**	percent
**BW or BWT**	bodyweight
**BWG**	Bodyweight gain
**C**	Celsius
**F**	Fahrenheit
**F or f**	female
**g**	gram
**hr or HR**	hour
**kg**	kilogram
**L**	liter
**M or m**	Male
**mg**	milligram
**min**	minute
**mL**	milliliter
**N**	Number or sample size
**NA or N/A**	not applicable
**No. or #**	number
**rcf**	relative centrifugal field
**rpm**	revolutions per minute
**S.D.**	standard deviation
**SD**	study day/ standard deviation
**sec**	second
**ug or µg**	microgram
**U**	units
**wk**	week
**FDA**	Food and Drug Administration
**GLP**	Good Laboratory Practices
**QA**	Quality Assurance
**QAU**	Quality Assurance Unit
**SOP**	Standard Operating Procedures

STUDY INFORMATION PAGE

Test Article:	[^{19}F]FP-$R_0$1-MG-F2
Study Initiation Date:	September 26, 2014
Initiation of Dosing:	October 7, 2014
In-life Completion Date:	October 21, 2014
Laboratory Completion Date:	February 24, 2015
Client:	Stanford University<br>1201 Welch Road, Rm PS049<br>Stanford, CA 94305-5484
Sponsor:	Frederick T. Chin, Ph.D.
Testing Facility:	SoBran Rangos Animal Facility<br>855 N. Wolfe Street, Suite 622<br>Baltimore, MD 21205
SoBran Management:	Greg Kelly, Ph.D.
Study Director:	Adrienne Edgell, BS, CMAR, LATG

TABLE OF CONTENTS

COMPLIANCE STATEMENT **3**

QUALITY ASSURANCE STATEMENT **4**

SIGNATURE PAGE **5**

ABBREVIATIONS **6**

STUDY INFORMATION PAGE **7**

I. SUMMARY 10
II. PURPOSE 11
III. MATERIALS AND METHODS 11
A. Test Article 11
B. Vehicle 11
C. Dose Preparation 11
D. Test System 12
1. Animals 12
2. Housing 12
3. Husbandry 12
E. Test Procedure 13
1. Randomization 13
2. Treatment 13
3. Body Weights 13
4. Clinical Observations 13
5. Blood Collection 14
6. Necropsy 14
F. Statistics 15
1. Body Weight 15
2. Organ Weights 15
3. Clinical Pathology 15
G. Deviations from the Protocol 16
IV. REFERENCES 17
V. ARCHIVES 18
VI. RESULTS 18
1. Body Weights 18
2. Survival and Clinical Observations 18
3. Clinical Pathology 19
4. Pathology/Histology 19
VII. CONCLUSION 20
VIII. APPENDICES 28
APPENDIX A – PROTOCOL 29
APPENDIX B – CLINICAL PATHOLOGY REPORT 47
APPENDIX C – PATHOLOGY REPORT 110
APPENDIX D – CERTIFICATE OF ANALYSIS FOR VEHICLE COMPONENTS AND TEST ARTICLE 176
APPENDIX E – STATISTICAL REPORT 185

Tables

TABLE 1: EXPERIMENTAL DESIGN 13
TABLE 2: TISSUES COLLECTED FOR HISTOLOGY 14
TABLE 3: ORGANS WEIGHED DURING NECROPSY 15
TABLE 4: DAY 1 BODYWEIGHTS FOR MALE AND FEMALE RATS 21
TABLE 5: DAY 2 (PRE-FASTED) BODYWEIGHTS FOR MALE AND FEMALE RATS 22
TABLE 6: DAY 3 (FASTED) BODYWEIGHTS FOR MALE AND FEMALE RATS 22
TABLE 7: DAY 8 BODYWEIGHTS FOR MALE AND FEMALE RATS 23
TABLE 8: DAY 14 (PRE-FASTED) BODYWEIGHTS FOR MALE AND FEMALE RATS 23
TABLE 9: DAY 15 (FASTED) BODYWEIGHTS FOR MALE AND FEMALE RATS 24
TABLE 10: BODYWEIGHT GAINS (BWG) FOR MALE AND FEMALE RATS 25
TABLE 11: ORGAN WEIGHTS FOR MALE AND FEMALE RATS- DAY 3 26
TABLE 12: ORGAN WEIGHTS FOR MALE AND FEMALE RATS- DAY 15 27

I. SUMMARY

The purpose of this study was to evaluate the toxicity of [^{19}F]FP-$R_0$1-MG-F2 following a single intravenous dose in rats. This study consisted of one test article treatment group of ten male and ten female Sprague-Dawley rats dosed with [^{19}F]FP-$R_0$1-MG-F2 (note - small molecule only, [^{19}F] is in place of [^{18}F] radioactive component which will be used clinically) at 1.10 mg/kg, equivalent to 250x the anticipated clinical dose. An additional group of ten males and ten females received the vehicle, 10% Ethanol in Sterile Saline, and served as the control. All rats received a dose volume of 5 mL/kg. The rats were dosed intravenously once on Study Day 1. Five male and five female rats from each group were bled on Study Day 3 and the remaining rats were bled on Study Day 15. All animals were euthanized and necropsied following blood collection. Parameters evaluated for test article effect included survival, clinical observations, body weight, body weight gain, clinical pathology, gross pathology, organ weights, and microscopic pathology.

All rats survived to the scheduled termination and remained bright, alert and responsive during the study. No abnormal findings were indicated during mortality checks (cagesides) or hands-on observations. There was significant increase in bodyweights on Day 3 for treated females, but this does not appear to be treatment related.

Treated male rats showed a statistically significant elevation in white blood cell parameters, select red blood cell factors, and select chemistry parameters but the changes do not seem to be related to treatment related. Treated females showed a statistically significant decrease in certain coagulation factors, but these changes do not appear to be treatment related either.

No treatment-related findings were noted in organ weights or microscopic pathology findings.

Under the conditions of this study, there were no treatment related findings in Sprague Dawley rats three or fifteen days after a single intravenous dose of [^{19}F]FP-$R_0$1-MG-F2 at 1.10 mg/kg.

II. PURPOSE

The purpose of this study was to evaluate the toxicity of [^{19}F]FP-$R_0$1-MG-F2 following a single intravenous dose in rats.

III. MATERIALS AND METHODS

A. Test Article

The test article (TA), [^{19}F]FP-$R_0$1-MG-F2, was received from the Sponsor on August 25, 2014, described as a white powder. The test article was stored frozen in a glass vial.
Copies of the Test Article Data Sheets are included in Appendix D.

Note: Test article name as per the Quality Control Record (Appendix D) is listed as FP-GLY-36-Gly-NH_2, which represents the chemical structure. The test article is referred to by the commercial name provided by the client ([^{19}F]FP-$R_0$1-MG-F2) throughout this report and in the raw study data.

Name	Lot No.	Supplier	Purity
[^{19}F]FP-$R_0$1-MG-F2	N149	C S Bio Co.; Menlo Park, CA	95.48%

B. Vehicle

The vehicle used in this study was 10% Ethanol in Sterile Saline (0.9% sodium chloride, USP). The vehicle components were purchased commercially by SoBran and stored at room temperature in accordance with the manufacturer instructions. Copies of the Certificate of Analyses for the lots of 10% Ethanol and Sterile Saline used are included in Appendix D.

Name	Lot No.	Supplier	Concentration
Ethanol (200 proof), USP	SHBB0212V	Sigma Aldrich; Saint Louis, MO.	99.87%
0.9% Sodium Chloride, USP	C886374	Baxter; Cleveland, MS	0.888%

C. Dose Preparation

The 0.22 mg/mL test article solution was prepared by adding 40 mL of 10% ethanol solution (vehicle control) to 8.8 mg of the test article, mixed gently, and then filtered using a 2µ sterile syringe filter. The pH of the 10% ethanol solution was adjusted using HCl and NaOH to achieve a pH of 7.4. The vehicle was passed through a 2µ sterile syringe filter after pH adjustment. The test article solution pH remained 7.4 and, therefore, did not need further pH adjustment.

D. Test System

1. Animals

On October 2, 2014, 21 male and 21 female Sprague-Dawley rats were obtained from Harlan, Frederick, MD. The supplier verified that the animals were free of specific active infectious diseases prior to arrival at the SoBran facility. At initiation of dosing, the male and female rats were 7 weeks of age, (Date of Birth: 8/15/14). At randomization the body weights of the rats used on study ranged from 199.05 to 218.61 grams (males) and 140.99 to 159.28 grams (females).

Rats were chosen for use in this study since they are considered an acceptable animal model for the rodent species for acute toxicity tests prior to clinical use.

2. Housing

The animals were housed following the specifications recommended in The Guide for the Care and Use of Laboratory Animals, Eighth Edition (National Academy Press, Washington, D.C., 2011). Animals were housed in an environmentally controlled room which maintained the temperatures of 68 to 79°F. The facility maintained a relative humidity of 30 to 70% with a 12-hour light/12-hour dark cycle. Animals were group housed (up to 3 per cage) based on group/sex designation in individually ventilated microisolator cages. Contact sani-chips bedding (Harlan TEKLAD, Indianapolis, IN) was used to absorb liquids. Bedding analysis records are on file in the facility and did not reveal any contaminants that would have an effect on the results of the study.

Access to the Animal Facility is dictated by Standard Operating Procedures (SOPs) and was restricted to authorized personnel only.

3. Husbandry

The animals were provided *ad libitum* access to drinking water (Baltimore City Water System, Baltimore, MD) via water bottles. The animals were provided *ad libitum* access to Harlan TEKLAD Certified Global Rodent Diet 2016C (Harlan TEKLAD, Indianapolis, IN) except when fasted overnight prior to blood collection. Rats were also offered enrichment devices of polycarbonate red tubes. Periodic water analyses and individual feed lot analyses (on file at SoBran) did not reveal any contaminants that would have an effect on the results of the study. The animals were acclimated for five days prior to dosing. No disease-related signs were noted during acclimation period. Prior to being placed on test, the Clinical Veterinarian approved all the animals for study use. All animals assigned to the study appeared normal on Day 1 prior to dosing.

E. Test Procedure

1. Randomization

The animals were randomized on October 6, 2014 by body weight into two groups of 10 male and 10 female rats using a computer-generated randomization program, Excel. The animals were group housed (up to 3 per cage). Each animal was assigned a unique identification number that was displayed using stainless steel ear tags and also on a card on the front of each cage. Raw data and records were also identified with the animal number.

2. Treatment

Dosing was initiated on October 7, 2014. Each rat was dosed once intravenously via the lateral tail vein with 5 mL/kg of the test article formulation or vehicle control. See Table 1 for a summary of the experimental design:

Table 1: Experimental Design

Group	Dose Concentration	Total Number of Animals			
		Day 3 Cohort		Day 15 Cohort	
		Male	Female	Male	Female
1. Control (10% Ethanol in 0.9% Sterile Saline)	0 mg/mL	5	5	5	5
2. [^{19}F]FP-$R_0$1-MG-F2 (1.10 mg/kg)	0.22 mg/mL	5	5	5	5

3. Body Weights

Body weights of individual animals were taken for randomization (pretest) and on Study Day 1 (for calculating individual dose volumes). Day 3 cohort animals were also weighed individually on Study Days 2 (pre-fasting) and 3 (post-fasting). Day 15 cohort animals were also weighed on Study Days 8, 14 (pre-fasting) and 15 (post-fasting).

4. Clinical Observations

During the course of this study, all animals were observed twice daily for moribundity, mortality, signs of toxicity and overall appearance (cageside observations). Clinical hands-on observations were collected on Study Days 1, 2 and 3 (for animals terminated on Day 3), or on Study Days 1, 8, 14 and 15 (for animals terminated on Day 15).

5. Blood Collection

Five male and five female rats from each group were bled on Study Day 3 and the remaining five male and five female rats from each group were bled on Study Day 15. Immediately prior to bleeding the animals were anesthetized with Isoflurane and then bled from the retro orbital sinus. Each animal had a hematology sample collected into a tube containing K_2 EDTA, a clinical chemistry sample collected into a serum separator tube and a coagulation factor sample collected in a sodium citrate tube. Each tube was individually labeled (date, group, sex, and animal number). The hematology samples were sent to Bioanalytical Systems, Inc. (BASI) on cold packs the same day as collection for first overnight delivery. The clinical chemistry and coagulations samples were centrifuged, serum/plasma drawn off and placed into individually labeled vials, and stored frozen until shipped on dry ice to BASI following study termination. The clinical pathology report is provided in Appendix B.

Note- multiple coagulation samples (including all fibrinogen analysis) could not be processed due to inadequate volume required for testing/resampling procedures. The non-processed samples are addressed in the clinical pathology report (Appendix B) and documented as a deviation (as applicable) in Section G.

6. Necropsy

Following blood collection, animals were euthanized using CO_2 overdose, followed by a thoracotomy to ensure death. A comprehensive necropsy was then performed for each animal. Once the lungs were examined and weighed they were inflated with formalin to ensure proper fixation. All tissues were placed in an individually labeled container containing 10% neutral-buffered formalin, with the exception of testis (males) and eyes with optic nerves, which were preserved in modified Davidson's fixative. Testis and eyes with optic nerves were transferred from the modified Davidson's solution to 70% Ethanol following collection. All containers were labeled with study number, date, group number, animal number, and Cohort (Day 3 or 15). The pathology report is provided in Appendix C. See Table 2 for the specific tissues that were collected and Table 3 for the organs that were weighed during necropsy:

Table 2: Tissues Collected for Histology

Brain	Lungs
Cecum	Lymph node (mesenteric)
Colon	Ovaries (2) (females)
Eyes with optic nerves (2)	Salivary glands (mandibular) (2)
Heart	Spleen
Ileum	Testes (2) (males)
Injection site	Thyroid with parathyroid (2)
Kidneys (2)	Trachea
Lesions (if present)	Urinary bladder
Liver	

Table 3: Organs Weighed During Necropsy

Brain	Lungs
Eyes with optic nerves	Ovaries
Heart	Spleen
Kidneys	Testes
Liver	Thyroid with parathyroid

F. Statistics

1. Body Weight

Comprehensive statistical analysis (mean, standard deviations, N) was conducted for group mean body weight data comparing treated groups to the control group of each sex using one-way Analysis of Variance (ANOVA) followed by Dunnett's t-test if there is significance. The probability value of less than 0.05 (two-tailed) was used as the critical level of significance for all tests.

2. Organ Weights

Comprehensive statistical analysis (mean, standard deviations, N) was conducted for organ weight data comparing treated groups to the control group of each sex using one-way Analysis of Variance (ANOVA) followed by Dunnett's t-test if there is significance. The probability value of less than 0.05 (two-tailed) was used as the critical level of significance for all tests.

3. Clinical Pathology

Comprehensive statistical analysis (mean, standard deviations, N) was conducted for clinical pathology data comparing treated groups to the control group of each sex using one-way Analysis of Variance (ANOVA), followed by Dunnett's t-test if there is significance. The probability value of less than 0.05 (two-tailed) was used as the critical level of significance for all tests.

G. Deviations from the Protocol

1. NCR-0109: Protocol section 10.9 states that organ weights will be collected for select tissues. The following animals/tissues were missing weights due to placement in formalin prior to weight collection: #823, Group 1, female - missing weights for both ovaries and thyroid with parathyroid; #833, Group 2, female - missing weight for one ovary.

   Resolution: Missing organ weights do not impact study outcome. Adequate weights were collected on additional animals within each group to provide statistical comparison. Organs are in formalin so they can still be processed and observed for microscopic changes.

2. NCR-0110: Protocol section 10.9 states that tissues preserved in modified Davidson's fixative will be transferred to ethanol 1-2 days following the necropsy. Cohort Day 3 tissues (testis and eyes with optic nerve) were transferred to 70% ethanol 11 days following the 10/9/14 necropsy.

   Resolution: Information was forwarded to Pathologist to take into account when examining tissues. He reported that there were no issues reading the slides for the eyes and testes due to being left in the Davidson's fixative for an extended period of time. This deviation has no impact on the study.

3. NCR-0111: On 10/21/14 (Day 15) The following animals/tissues had weights taken before the scale was pre-calibrated #828, Group 1 – Female: all organs (brain, eyes with optic nerves, heart, kidneys, liver, lungs, ovaries, spleen and thyroid with parathyroid); #806, Group 1 – Male: testes. SOP-FSO-420, Section 5.4 states to calibrate the analytical scale by collecting 3 weights from a set of analytical weights prior to weighing anything.

   Resolution: The late scale calibration has no impact on the study integrity. Scale was properly calibrated during organ weight collection of the second animal necropsied that day and was found to be within normal limits.

4. NCR-0121: As per protocol section 10.7, Fibrinogen (a coagulation factor) should be analyzed for all animals on study. The clinical pathology lab stated the plasma volumes for the coagulation samples collected for both Day 3 and Day 15 animals were insufficient to run Fibrinogen analysis. This parameter was therefore excluded from the study data.

   Resolution: The clinical pathology lab contacted the Study Director on 10/23/14 to inform SoBran that there would not be adequate plasma volume to run all three coagulation tests as stated in the protocol. The Study Director decided to exclude Fibrinogen since that test requires the largest volume and the other tests (PTT and APTT) should provide adequate data to show if the test article had effects on the coagulation factors. The lack of Fibrinogen data has no study impact.

5. NCR-0122: Protocol Section 10.5 states that animals need to be checked once daily in AM during acclimation. The following dates had checks done in the PM: 10/4/14, 10/5/14 and 10/6/14.

   Resolution: There is no study impact since animals were checked at least once daily, which satisfies welfare compliance.

6. NCR-0123: Testes and eyes with optic nerves were transfer to 70% Ethanol by SoBran staff prior to transfer to the histology lab. The protocol states the transfer will be documented in the histology records (Section 10.9), but this was not possible since the tissues were transferred prior to being shipped to the histology lab. The transfer was documented via memos that will be retained in the study files.

   Resolution: A memo was written to address the location of the documentation for the tissue transfer. It was decided that a protocol amendment for this single item was not necessary since the location of the actual tissue transfer and documentation has no impact on study integrity as long as it was documented (which was done via memos included in the raw data files).

IV. REFERENCES

Historical clinical pathology data reference - Harlan Hsd:Sprague Dawley complete blood count and serum chemistry data which is available on the Harlan Research Laboratories website

SBR-SOP-GFO-203 Facility Access and Entry
SBR-SOP-GFO-206 Ordering Receipt and Storage of Feed and Bedding
SBR-SOP-GFO-208 Storage and Shipment of Blood and Tissue Samples
SBR-SOP-FEC-501 Monitoring and Control of Temperature and Humidity
SBR-SOP-FEC-502 Monitoring and Control of Facility Lighting
SBR-SOP-FEC-504 Responding to Facility Alarms for Environmental Controls
SBR-SOP-FEC-505 Use and Maintenance of Dickson Chart Recorder
SBR-SOP-FSO-415 Equipment Calibration and Certification
SBR-SOP-FSO-420 Operation of the Sartorius Practum 224-1S Analytical Balance
SBR-SOP-FSO-421 Operation of the Ohaus EP4102C Balance
SBR-SOP-FSO-423 Operation and Maintenance of the Accuspin Micro 17/17R Centrifuge
SBR-SOP-HUP-601 Husbandry and Health Monitoring Procedures
SBR-SOP-HUP-605 Environmental Enrichment for Rodents
SBR-SOP-HUP-612 Innovive Cage Change Procedures - Rats
SBR-SOP-AOR-701 Ordering, Receiving and Housing Animals
SBR-SOP-TDU-806 Blood Collection Procedures for Rodents
SBR-SOP-TDU-807 Injection Procedures and Guidelines for Rodents
SBR-SOP-TDU-809 Animal Identification-Ear tags
SBR-SOP-TDU-818 Comprehensive Necropsy Procedures in Rodents
SBR-SOP-TDU-819 Collecting Organ Weights
SBR-SOP-TDU-824 Randomization/Allocation Using Microsoft Excel
SBR-SOP-TDU-826 Isoflurane Machine Maintenance and Use
SBR-SOP-TDU-827 Clinical Observations in Rodents

V. ARCHIVES

Raw data generated by the testing facility, any communication regarding the conduct of the study, and a copy of the final report will be stored in SoBran's archive room at the SoBran BioScience office in Burtonsville, Maryland for 5 years following the issuance of the final report. Retention samples of the dose formulations will be retained for this study and stored at the Rangos animal facility in Baltimore, Maryland for 2 years following the issuance of this report.

Archiving of raw data and reports provided by the subcontracted clinical pathology lab is noted in the final clinical pathology report (refer to Appendix B). A hard copy of the final clinical pathology report will be archived by SoBran in Burtonsville, Maryland with other study raw data.

Pathology raw data, a copy of the pathology report final report, tissues, blocks, and slides will be archived at HSRL archive facilities in Mount Jackson, Virginia following issuance of the final pathology report. All raw pathology data and a copy for the final pathology report will be retained for a period of 5 years following issuance of the final study report. All tissues, blocks, and slides will be retained for a period of 2 years prior to proper disposal or transfer to the client. A hard copy of the final clinical pathology report will be archived by SoBran in Burtonsville, Maryland with other study raw data.

VI. RESULTS

1. Body Weights

Mean and individual body weights are presented in Table 4 (Day 1), Table 5 (Day 2), Table 6 (Day 3), Table 7 (Day 8), Table 8 (Day 14) and Table 9 (Day 15).

Mean and individual body weight gains (BWG) are presented Table 10. In the Day 3 cohort, one vehicle control male, two vehicle control females and two treated female rats lost weight between Days 1-2. All Day 3 cohort rats lost weight between Days 1-3 after being fasted, except for one male vehicle control and two male treated rats. In the Day 15 cohort, one female vehicle control and one female treated rat lost weight between Days 8-15 but, all other rats gained weight during that time. All Day 15 cohort rats gained weight between Days 1-8, 8-14, 1-14 and 1-15 and lost weight after being fasted on Days 14-15.

There was a significant increase in bodyweights for treated (Group 2) Females on Day 3 when compared to the control value, $p < 0.05$ for the Female controls (Group 1). Although this difference is significant, it is a single incident and does not appear to be treatment related.

2. Survival and Clinical Observations

All rats survived to the scheduled termination date and remained bright, alert and responsive during the course of this study. No abnormal findings were indicated during daily mortally (cageside) or hands-on observations.

3. Clinical Pathology

The final clinical pathology report is provided in Appendix B. There were no treatment-related findings from clinical chemistry, hematology, and coagulation samples collected on Study Days 3 and 15.

For hematology samples, statistical analysis of the data for study Day 3 revealed white blood cell counts (WBC), absolute lymphocyte counts (ALY), absolute monocyte counts (AMO), and absolute large unstained cell counts (ALUC) were increased as compared to the control value, $p < 0.05$ for Group 2 males. On Day 15, Group 2 males showed a statistically significant increase in mean corpuscular hemoglobin (MCH) as compared to the control value, $p < 0.05$. All levels were found to be within the standard historical range for Sprague Dawley rats, the changes are not believed to be biologically meaningful because of the small magnitude of the difference from the control values. Refer to Section IV for historical reference.

For coagulation samples, statistical analysis of the data for study Day 15 showed a statistically significant decrease in activated partial thromboplastin time as compared to the control value, $p < 0.05$ for Group 2 females. All levels were found to be within the standard historical range for Sprague Dawley rats, and therefore do not appear to be treatment related. Refer to Section IV for historical reference.

For clinical chemistry samples, statistical analysis of the data for study Day 3 revealed that alkaline phosphatase (ALP) showed a significant increase as compared to the control value, $p < 0.05$ for Group 2 males. On study Days 3 and 15 alanine aminotransferase (ALT) was elevated as compared to the control value, $p < 0.05$ for Group 2 males. Although these values do appear higher than the historical reference range and show shows statistical significance, the changes are not believed to be biologically meaningful because of the small magnitude of the difference from the control values. Refer to Section IV for historical reference.

4. Pathology/Histology

Protocol specified organ weights are shown in Tables 10 and 11. The final pathology report is provided in Appendix C.

No treatment-related findings and statistical significance were noted in the organ weights or microscopic pathology findings. The test article, [^{19}F]FP-$R_0$1-MG-F2 at 1.10 mg/kg had no adverse effects in any of the tissues examined.

VII. CONCLUSION

The goal of this study was to evaluate the toxicity of [^{19}F]FP-$R_0$1-MG-F2 three and fifteen days following a single intravenous dose.

Animals remained bright, alert, and responsive at all times and did not exhibit signs of toxicity during the conduct of the study. No treatment-related differences were noted in mean body weights and body weight changes, clinical chemistry, hematology, or coagulation parameters. In addition, no treatment-related effects were observed organ weights or in gross and microscopic pathology.

Under the conditions of this study, there were no treatment related findings in Sprague Dawley rats three or fifteen days after a single intravenous dose of [^{19}F]FP-$R_0$1-MG-F2 at 1.10 mg/kg.

Table 4: Day 1 Bodyweights for Male and Female Rats

Animal Number	Group/ Sex	Bodyweight (g)	Animal Number	Group/ Sex	Bodyweight (g)
801	1/M	209.18	823	1/F	151.91
802	1/M	206.54	824	1/F	149.54
803	1/M	205.90	825	1/F	146.70
804	1/M	219.91	826	1/F	147.89
805	1/M	205.25	827	1/F	154.81
806	1/M	211.33	828	1/F	152.89
807	1/M	213.74	829	1/F	158.21
808	1/M	213.20	830	1/F	152.50
809	1/M	207.12	831	1/F	151.91
810	1/M	204.58	832	1/F	148.87
Mean		209.68	Mean		151.52
SD		4.86	SD		3.43
811	2/M	199.43	833	2/F	153.87
812	2/M	209.47	834	2/F	151.37
813	2/M	209.00	835	2/F	157.87
814	2/M	210.57	836	2/F	152.01
815	2/M	213.35	837	2/F	154.70
816	2/M	211.76	838	2/F	152.16
817	2/M	205.89	839	2/F	150.51
818	2/M	204.12	840	2/F	153.83
819	2/M	212.16	841	2/F	142.77
820	2/M	204.08	842	2/F	149.61
Mean		207.98	Mean		151.87
SD		4.45	SD		3.97

Table 5: Day 2 (pre-fasted) Bodyweights for Male and Female Rats

Animal Number	Group/ Sex	Bodyweight (g)	Animal Number	Group/ Sex	Bodyweight (g)
801	1/M	208.37	823	1/F	150.11
802	1/M	207.51	824	1/F	148.07
803	1/M	214.84	825	1/F	147.53
804	1/M	222.12	826	1/F	154.39
805	1/M	209.76	827	1/F	158.85
Mean		212.52	Mean		151.79
SD		6.07	SD		4.78
811	2/M	203.22	833	2/F	154.58
812	2/M	209.88	834	2/F	150.58
813	2/M	210.79	835	2/F	153.65
814	2/M	214.90	836	2/F	156.79
815	2/M	223.10	837	2/F	155.51
Mean		212.38	Mean		154.22
SD		7.31	SD		2.34

Table 6: Day 3 (fasted) Bodyweights for Male and Female Rats

Animal Number	Group/ Sex	Bodyweight (g)	Animal Number	Group/ Sex	Bodyweight (g)
801	1/M	202.31	823	1/F	144.22
802	1/M	203.44	824	1/F	143.83
803	1/M	207.12	825	1/F	142.89
804	1/M	215.23	826	1/F	146.84
805	1/M	201.67	827	1/F	149.69
Mean		205.95	Mean		145.49
SD		5.60	SD		2.77
811	2/M	198.58	833	2/F	147.96
812	2/M	204.07	834	2/F	151.35
813	2/M	206.66	835	2/F	149.17
814	2/M	215.98	836	2/F	148.96
815	2/M	224.38	837	2/F	147.55
Mean		209.93	Mean		149.00
SD		10.24	SD		1.48

Table 7: Day 8 Bodyweights for Male and Female Rats

Animal Number	Group/ Sex	Bodyweight (g)	Animal Number	Group/ Sex	Bodyweight (g)
806	1/M	248.95	828	1/F	174.81
807	1/M	251.46	829	1/F	179.32
808	1/M	254.46	830	1/F	173.39
809	1/M	250.08	831	1/F	178.61
810	1/M	245.14	832	1/F	162.30
Mean		250.02	Mean		173.69
SD		3.42	SD		6.84
816	2/M	251.42	838	2/F	173.63
817	2/M	243.66	839	2/F	173.53
818	2/M	247.57	840	2/F	178.64
819	2/M	243.31	841	2/F	166.33
820	2/M	245.03	842	2/F	185.14
Mean		246.20	Mean		175.45
SD		3.37	SD		6.97

Table 8: Day 14 (pre-fasted) Bodyweights for Male and Female Rats

Animal Number	Group/ Sex	Bodyweight (g)	Animal Number	Group/ Sex	Bodyweight (g)
806	1/M	286.52	828	1/F	195.95
807	1/M	279.91	829	1/F	192.71
808	1/M	281.42	830	1/F	178.81
809	1/M	277.51	831	1/F	190.75
810	1/M	270.34	832	1/F	176.10
Mean		279.14	Mean		186.86
SD		5.92	SD		8.84
816	2/M	283.76	838	2/F	183.01
817	2/M	270.73	839	2/F	186.02
818	2/M	279.26	840	2/F	187.58
819	2/M	273.84	841	2/F	178.99
820	2/M	273.47	842	2/F	203.68
Mean		276.21	Mean		187.86
SD		5.23	SD		9.43

Table 9: Day 15 (fasted) Bodyweights for Male and Female Rats

Animal Number	Group/ Sex	Bodyweight (g)	Animal Number	Group/ Sex	Bodyweight (g)
806	1/M	279.44	828	1/F	186.02
807	1/M	267.92	829	1/F	182.90
808	1/M	269.08	830	1/F	173.17
809	1/M	269.54	831	1/F	180.75
810	1/M	266.15	832	1/F	166.92
Mean		270.43	Mean		177.95
SD		5.21	SD		7.78
816	2/M	275.69	838	2/F	177.50
817	2/M	262.76	839	2/F	177.43
818	2/M	267.11	840	2/F	178.43
819	2/M	261.76	841	2/F	173.63
820	2/M	265.08	842	2/F	192.24
Mean		266.48	Mean		179.85
SD		5.55	SD		7.17

Table 10: Bodyweight Gains (BWG) for Male and Female Rats

Animal Number	Group/ Sex	BWG(g) Days 1-2	BWG(g) Days 1-3	BWG(g) Days 1-8	BWG(g) Days 8-14	BWG(g) Days 8-15	BWG(g) Days 14-15	BWG(g) Days 1-14	BWG(g) Days 1-15
801	1/M	-0.81	-6.87						
802	1/M	0.97	-3.10						
803	1/M	8.94	1.22						
804	1/M	2.21	-4.68						
805	1/M	4.51	-3.58						
806	1/M			37.62	37.57	30.49	-7.08	75.19	68.11
807	1/M			37.72	28.45	16.46	-11.99	66.17	54.18
808	1/M			41.26	26.96	14.62	-12.34	68.22	55.88
809	1/M			42.96	27.43	19.46	-7.97	70.39	62.42
810	1/M			40.56	25.20	21.01	-4.19	65.76	61.57
823	1/F	-1.80	-7.69						
824	1/F	-1.47	-5.71						
825	1/F	0.83	-3.81						
826	1/F	6.50	-1.05						
827	1/F	4.04	-5.12						
828	1/F			21.92	21.14	11.21	-9.93	43.06	33.13
829	1/F			21.11	13.39	3.58	-9.81	34.50	24.69
830	1/F			20.89	5.42	-0.22	-5.64	26.31	20.67
831	1/F			26.70	12.14	2.14	-10.00	38.84	28.84
832	1/F			13.43	13.80	4.62	-9.18	27.23	18.05
811	2/M	3.79	-0.85						
812	2/M	0.41	-5.40						
813	2/M	1.79	-2.34						
814	2/M	4.33	5.41						
815	2/M	9.75	11.03						
816	2/M			39.66	32.34	24.27	-8.07	72.00	63.93
817	2/M			37.77	27.07	19.10	-7.97	64.84	56.87
818	2/M			43.45	31.69	19.54	-12.15	75.14	62.99
819	2/M			31.15	30.53	18.45	-12.08	61.68	49.60
820	2/M			40.95	28.44	20.05	-8.39	69.39	61.00
833	2/F	0.71	-5.91						
834	2/F	-0.79	-0.02						
835	2/F	-4.22	-8.70						
836	2/F	4.78	-3.05						
837	2/F	0.81	-7.15						
838	2/F			21.47	9.38	3.87	-5.51	30.85	25.34
839	2/F			23.02	12.49	3.90	-8.59	35.51	26.92
840	2/F			24.81	8.94	-0.21	-9.15	33.75	24.60
841	2/F			23.56	12.66	7.30	-5.36	36.22	30.86
842	2/F			35.53	18.54	7.10	-11.44	54.07	42.63

Table 11: Organ Weights for Male and Female Rats- Day 3

Animal Number	Group / Sex	Brain	Eyes with optic nerve	Heart	Kidneys	Liver	Lungs	Ovaries (females)	Spleen	Testes (males)	Thyroid (both lobes with parathyroid)
801	1 / M	1.6187	0.2553	0.8236	1.5969	8.2068	1.3300	N/A	0.4647	2.9974	0.0105
802	1 / M	1.5895	0.2774	0.9431	1.7355	7.3524	1.3785	N/A	0.4082	2.8174	0.0282
803	1 / M	1.7611	0.3125	0.9733	1.5963	8.0136	2.0736	N/A	0.5765	2.9797	0.0293
804	1 / M	1.7167	0.2868	1.6078	1.6141	8.6535	1.6981	N/A	0.5732	2.5926	0.0221
805	1 / M	1.6767	0.2473	0.9815	1.7941	7.8703	1.8987	N/A	0.6079	2.9330	0.0375
	Mean	1.6725	0.2759	1.0659	1.6674	8.0193	1.6758	N/A	0.5261	2.8640	0.0255
	SD	0.0700	0.0260	0.3095	0.0916	0.4755	0.3226	N/A	0.0853	0.1672	0.0100
823	1 / F	1.4885	0.2791	0.6037	1.2982	5.9815	1.1521	N/A*	0.4520	N/A	N/A*
824	1 / F	1.5898	0.2921	0.6100	1.2365	6.8848	1.3262	0.1635	0.4549	N/A	0.0240
825	1 / F	1.5144	0.2189	0.5775	1.2986	6.4101	1.5210	0.1782	0.5683	N/A	0.0446
826	1 / F	1.5544	0.2655	0.6572	1.3486	6.1701	1.4283	0.2033	0.5411	N/A	0.0346
827	1 / F	1.5940	0.2478	0.6720	1.3949	5.6276	1.3971	0.1348	0.4703	N/A	0.0559
	Mean	1.5482	0.2607	0.6241	1.3154	6.2148	1.3649	0.1700	0.4973	N/A	0.0398
	SD	0.0463	0.0286	0.0393	0.0596	0.4712	0.1380	0.0286	0.0537	N/A	0.0136
811	2 / M	1.7281	0.3290	0.8529	1.6206	8.4231	1.6850	N/A	0.6353	3.0781	0.0290
812	2 / M	1.7266	0.3450	0.9952	1.6078	7.6844	1.7598	N/A	0.6231	3.0267	0.0335
813	2 / M	1.8007	0.4062	0.8993	1.6440	8.1038	1.4014	N/A	0.7603	3.0173	0.0301
814	2 / M	1.6529	0.2498	1.0125	1.8016	8.5365	1.5284	N/A	0.6047	2.8828	0.0295
815	2 / M	1.6321	0.2780	0.9593	1.9042	9.7460	1.8096	N/A	0.6770	2.9709	0.0276
	Mean	1.7081	0.3216	0.9438	1.7156	8.4988	1.6368	N/A	0.6601	2.9952	0.0299
	SD	0.0673	0.0609	0.0668	0.1311	0.7717	0.1692	N/A	0.0620	0.0734	0.0022
833	2 / F	1.5601	0.3082	0.6630	1.3477	5.9531	1.1286	N/A*	0.4127	N/A	0.0466
834	2 / F	1.4948	0.2457	0.5905	1.1751	5.9220	1.6180	0.1678	0.5219	N/A	0.0408
835	2 / F	1.5149	0.2349	0.6881	1.3088	5.6584	1.7632	0.1519	0.4723	N/A	0.0351
836	2 / F	1.5319	0.2678	0.7113	1.3993	6.3018	1.7209	0.1453	0.6027	N/A	0.0538
837	2 / F	1.7811	0.2640	0.6170	1.2061	5.9254	1.4211	0.1729	0.4677	N/A	0.0631
	Mean	1.5766	0.2641	0.6540	1.2874	5.9521	1.5304	0.1595	0.4955	N/A	0.0479
	SD	0.1168	0.0281	0.0498	0.0947	0.2292	0.2606	0.0130	0.0713	N/A	0.0110

* Tissues inadvertently not weighed, therefore, not included in calculations.

Table 12: Organ Weights for Male and Female Rats- Day 15

Animal Number	Group / Sex	Brain	Eyes with optic nerve	Heart	Kidneys	Liver	Lungs	Ovaries (females)	Spleen	Testes (males)	Thyroid (both lobes with parathyroid)
806	1 / M	1.8215	0.3210	1.1287	2.0317	11.6219	2.2523	N/A	0.6086	3.7334	0.0347
807	1 / M	1.4194	0.3285	1.1874	2.0879	12.0388	1.8053	N/A	0.7695	3.7895	0.0908
808	1 / M	1.2565	0.2822	0.9670	2.0368	10.7669	2.0478	N/A	0.7287	3.1002	0.0210
809	1 / M	1.4941	0.3395	1.2401	2.0416	10.1976	1.9069	N/A	0.7420	3.5114	0.0307
810	1 / M	1.8118	0.3176	1.1677	2.0815	10.3406	2.1579	N/A	0.6748	3.3889	0.0333
	Mean	1.5607	0.3178	1.1382	2.0559	10.9932	2.0340	N/A	0.7047	3.5047	0.0421
	SD	0.2490	0.0216	0.1038	0.0266	0.8060	0.1814	N/A	0.0638	0.2786	0.0277
828	1 / F	1.6569	0.3078	0.7368	1.3741	6.2577	1.4714	0.1797	0.6371	N/A	0.0312
829	1 / F	1.7590	0.3056	0.7411	1.5149	6.4332	1.5283	0.1207	0.5455	N/A	0.0369
830	1 / F	1.4810	0.2915	0.6506	1.3012	6.2672	1.3336	0.1556	0.5196	N/A	0.0331
831	1 / F	1.6596	0.3079	0.7004	1.4582	6.6302	1.8339	0.1632	0.5827	N/A	0.0260
832	1 / F	1.7192	0.3064	0.6669	1.3826	6.0050	1.2391	0.1621	0.5025	N/A	0.0199
	Mean	1.6551	0.3038	0.6992	1.4062	6.3187	1.4813	0.1563	0.5575	N/A	0.0294
	SD	0.1063	0.0070	0.0405	0.0824	0.2318	0.2275	0.0218	0.0538	N/A	0.0066
816	2 / M	1.5072	0.3435	1.1279	2.2353	11.7634	2.2251	N/A	0.6466	3.7242	0.0214
817	2 / M	1.7842	0.3364	1.0344	1.9431	11.0516	1.8475	N/A	0.6990	3.5247	0.0362
818	2 / M	1.5765	0.3205	1.0491	2.2130	10.5215	1.8609	N/A	0.7520	3.6445	0.0190
819	2 / M	1.6366	0.3185	1.0460	2.0494	11.0145	2.3093	N/A	0.6968	3.3802	0.0178
820	2 / M	1.4663	0.2833	1.0391	2.1277	11.7669	1.8611	N/A	0.7009	3.6255	0.0246
	Mean	1.5942	0.3204	1.0593	2.1137	11.2236	2.0208	N/A	0.6991	3.5798	0.0238
	SD	0.1246	0.0233	0.0388	0.1205	0.5368	0.2270	N/A	0.0373	0.1323	0.0074
838	2 / F	1.5021	0.3203	0.7094	1.3112	7.2205	1.3458	0.1359	0.6333	N/A	0.0375
839	2 / F	1.5809	0.3132	0.7618	1.2866	7.3925	1.4940	0.1485	0.5277	N/A	0.0509
840	2 / F	1.7012	0.2856	0.7220	1.3434	6.3157	1.6097	0.1810	0.4710	N/A	0.0101
841	2 / F	1.5624	0.2713	0.6657	1.5467	7.9629	1.5117	0.1857	0.6217	N/A	0.0280
842	2 / F	1.6111	0.2920	0.6753	1.4593	7.8306	1.5704	0.1335	0.6054	N/A	0.0510
	Mean	1.5915	0.2965	0.7068	1.3894	7.3444	1.5063	0.1569	0.5718	N/A	0.0355
	SD	0.0731	0.0201	0.0385	0.1100	0.6509	0.1009	0.0248	0.0698	N/A	0.0172

VIII. APPENDICES

APPENDIX A – PROTOCOL

Motivated By Discovery

Final- Study Protocol

14-Day Single Intravenous Dose Toxicity Study of [18F]FP-R01-MG-F2 in Sprague Dawley Rats

SoBran Study Number: SB-SU-003

Prepared By
SoBran, Inc.
4000 Blackburn Lane
Suite 100
Burtonsville, MD 20866
Phone: 703-352-9511

September 25, 2014

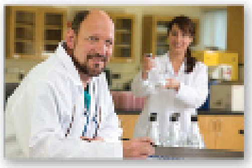
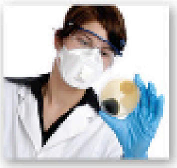
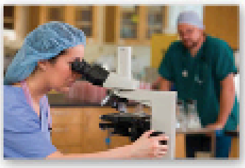
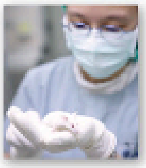

Motivated By Discovery

1. **Study Title:**

   14-Day Single Intravenous Dose Toxicity Study of [18F]FP-R01-MG-F2 in Sprague Dawley Rats

2. **Study Objectives:**

   The goal of this study is to assess the toxicity of [18F]FP-R01-MG-F2 a small molecule (peptide) that is administered with a specific radioactive probe during Positron Emission Tomography (PET) scans to visualize tumors or targeted cells. For this study the toxicity of [18F]FP-R01-MG-F2 (note - small molecule only, no radioactive component) will be assessed following a single intravenous (IV) dose of 1.10 mg/kg (250x the clinical dose) of the test article. The vehicle (10% ethanol in normal saline) will also be tested to establish baseline toxicity.

3. **Regulatory Compliance:**

   This study will be performed under GLP conditions, in compliance with the U.S. FDA's GLP regulations for nonclinical laboratory studies, 21CFR part 58.

4. **IACUC Approval:**

   The Institutional Animal Care and Use Committee (IACUC) has approved the proposed animal study.

   IACUC protocol number: SOB-015-2012
   Approval Date: 08/28/2014 (amendment)
   Expiration Date: 07/05/2015

5. **Test Facility:**

   SoBran Rangos Animal Facility
   855 N. Wolfe Street, Suite 622
   Baltimore, Maryland 21205

Test Sites:

5.1. Clinical Pathology:

Bioanalytical Systems, Inc. (BASi)
10424 Middle Mount Vernon Road
Mount Vernon, IN 47620
Tel: 812-985-5900

5.2. Histology/Pathology:

Histo-Scientific Research Laboratories (HSRL)
5930 Main Street
Mount Jackson, VA 22842
Tel: 540-477-4440

5.3. Formulation Analysis and Preparation:

SoBran
855 N. Wolfe Street, Suite 622
Baltimore, Maryland 21205

Note: Formulation analysis will not be performed as per the client. Only formulation preparation (as described in section 8.4 below) will be performed in the SoBran facility.

6. Study Management:

6.1. Client/Sponsor:

Frederick T. Chin, Ph.D.
Assistant Professor
Head, Cyclotron Radiochemistry
Stanford University School of Medicine
1201 Welch Road, Rm PS049
Stanford, CA 94305-5484 USA
Tel: (650) 725-4182
Email:chinf@stanford.edu

6.2. SoBran Study Director:

Adrienne Edgell, BS, CMAR, LATG
Tel: 703-652-9511 x236
Email: aedgell@sobran-inc.com

6.3. SoBran Study Management:

Greg Kelly, Ph.D.
Tel: 703-352-9511 ext. 238
Email: gkelly@sobran-inc.com

SoBran INC. Motivated By Discovery

7. Key Study Dates:

Study Phase	Study Day	Additional Notes/Proposed Dates
Study Initiation	N/A	Date study director signs protocol
Animal Receipt	N/A	Anticipated arrival:10/2/2014
Dosing	Day 1	IV dosed one time; 10/7/2014
Blood collection	Days 3 and 15	Prior to scheduled necropsy; Day 3 (10/9/2014). Day 15 (10/21/2014)
Euthanasia/Necropsy	Days 3 and 15	Following blood collection, according to scheduled necropsy
Audited draft report to client	Post-study	Within 8 weeks of study end
Final report signed	Post-study	Within 2 weeks of receiving client comments- Marks study completion

8. Test Compounds and Formulation:

8.1. Test Article:

Name: [18F]FP-R01-MG-F2
Supplier: Sponsor
Lot Number(s): Noted on study forms and in final report
Special Handling: Stored frozen (-20°C ±10°C) prior to formulation (as per sponsor); shipped and received from sponsor at ambient temperature

Stability & Storage: Test article will be provided by the sponsor. A Quality Control Record was provided for the lot/batch of test article to be used for this study and specific purity information is known. Test article is considered stable when stored at ambient temperature, but sponsor requested storage under freezing (-20°C ±10°C) conditions following receipt at the testing facility. Following formulation, the prepared test article solution can be stored under room temperature (18-26°C) conditions.

8.2. Vehicle:

Name: 10% Ethanol (USP) in Sterile Saline (0.9% sodium chloride, USP)
Supplier: Commercial vendor/supplier
Lot Number(s): Noted on study forms and in final report
Special Handling: None

Stability & Storage: Commercially available ethanol (200 proof, USP grade) will be mixed with sterile saline (0.9% sodium chloride solution, USP) to formulate the 10% ethanol in saline vehicle solution. Certificates of Analysis to be provided in final report to indicate purity and stability of the vehicle formulation components. Vehicle is considered stable when stored under room temperature (18-26°C) conditions.

Motivated By Discovery

8.3. Vehicle Formulation: Formulations will be prepared on Day 1 of the study, prior to dosing.

Preparation of Vehicle Solution:

1. Prepare 150 ml of 10% ethanol in normal saline solution by adding 15 ml of 200 proof ethanol, USP to 135 ml of sterile saline (0.9% sodium chloride solution, USP). Mix gently.
2. Filter solution using a sterile syringe filter.
3. Test pH of the solution with pH paper (pH should be between 7.2 and 7.9).
4. Use small amounts of NaOH or HCl to adjust pH if necessary. Document all chemical details including volume used on the preparation form.
5. Filter solution a second time if pH adjustment was required to ensure sterility.
6. Label flask with appropriate group designation (Group 1 vehicle).

8.4. Test Article Formulation: Formulations will be prepared on Day 1 of the study, prior to dosing.

Preparation of 0.22 mg/ml solution:

1. Weigh 8.8 mg of test article and transfer to an empty sterile flask.
2. Add 40 ml of 10% ethanol solution (vehicle solution) to the vial containing the test article. Mix gently and verify test article has fully dissolved into solution.
3. Filter solution using a sterile syringe filter.
4. Test pH of the solution with pH paper (pH should be between 7.2 and 7.9).
5. Use small amounts of NaOH or HCl to adjust pH if necessary. Document all chemical details including volume used on the preparation form.
6. Filter solution a second time if pH adjustment was required to ensure sterility.
7. Label with the appropriate group designation (Group 2 high dose).

8.5. **Dose Formulation Sampling and Analysis:**

8.5.1. Homogeneity: Dose formulations are solutions at the intended concentrations; homogeneity determination is not required.

8.5.2. Concentration Analysis: Will not be performed for this study as per the sponsor's request. Appropriate documentation of deviation from requirements will be addressed in the final report.

Motivated By Discovery

8.5.3. Stability: Will not be performed on individual formulations for this study as per the sponsor's request. Appropriate documentation of deviation from requirements will be addressed in the final report. Stability data is available for the bulk test article and will be included in the final report.

8.6. **Retention/Disposition:** A 1 ml aliquot (or remaining test article if less than 1 ml) of each prepared formulation and the vehicle will be retained and securely stored according to the storage conditions in 8.1 and 8.2 above. Following the in-life study phase, SoBran will contact the sponsor regarding the disposition of the remaining neat test article (if applicable). The remaining neat test article will either be returned to the sponsor, or properly discarded following issuance of the final report if sponsor fails to respond to the disposition request.

9. Study Details:

9.1. Animal Species: Rat

9.2. Strain: Sprague Dawley

9.3. Sex: Male and Female

9.4. Number of animals on study: 40 (20 ♂, 20♀)

9.5. Age requirements at study start: 6-8 weeks old, approximately 150-250 grams at study start

9.6. Supplier: Harlan

9.7. Study Summary: The goal of this study is to assess the toxicity of [18F]FP-R01-MG-F2. For this study the toxicity of [18F]FP-R01-MG-F2 (note - small molecule only, no radioactive component) will be assessed following a single intravenous (IV) dose of 1.10 mg/kg (250x the clinical dose) of the test article. The vehicle (10% ethanol in normal saline) will also be tested to establish baseline toxicity. Half of the animals (20) will be euthanized 3 days following dosing, while the remaining will be euthanized at the end of the study (Day 15). Blood will be collected from all animals on the day of euthanasia, followed by a comprehensive necropsy. Blood and tissues will be evaluated for abnormalities.

Motivated By Discovery

9.8. Husbandry Information:

Housing: Rats will be housed up to 3 per cage (based on sex/group/scheduled necropsy day) in individually ventilated microisolator cages. Cages are bedded with Harlan Teklad Sani-chips (7090C) bedding and will be changed according to facility standard operating procedures (SOPs). The room environment will be monitored and maintained within a temperature range of 68-79°F and 30-70% humidity. Fluorescent lighting is on an automatic schedule to provide 12 hours of light per day. General procedures for animal care and housing are conducted in accordance with facility SOPs, the *Guide for the Care and Use of Laboratory Animals*, (National Research Council; National Academies Press, Washington, DC, 2011), and the U.S. Department of Agriculture through the Animal Welfare Act (Public Law 99-198).

Food: Rats will be offered a commercially available rodent diet (Harlan Teklad Global Rodent Diet 2016C) *ad libitum* (except when fasted overnight prior to blood collection). Analysis of the feed, provided by the manufacturer, will be reviewed by the attending veterinarian or designee, to assure that no known contaminants are present that could interfere with or affect the outcome of the study. Analysis reports are kept on file in the SoBran facility.

Water: Rats will have *ad libitum* access to fresh drinking water via the public water system (i.e. tap water). Water will be provided in clean water bottles. Water bottles will be replaced according to facility SOPs. Samples of water from the animal facility are periodically analyzed, and reviewed by the attending veterinarian or designee, to assure that no known contaminants are present that could interfere with or affect the outcome of studies. Analysis reports are kept on file in the SoBran facility.

Enrichment: Rats will be provided polycarbonate red tubes as enrichment devices. Enrichment will be changed according to facility SOPs.

9.9. Animal Identification: Animals will be individually identified using stainless steel ear tags with unique test numbers (i.e. 1, 2, 3). Ear tags will be applied at the time of randomization. The number will correspond with the animal's cage card and raw data generated during conduct of the study.

10. Experimental Design:

10.1. Acclimation: Animals will be allowed to acclimate at least 72 hours prior to use and veterinary release.

10.2. Randomization: Following the acclimation period but prior to dosing, all animals will have individual bodyweights collected and documented. General animal health and condition will also be visualized at the time of bodyweight collection. Any abnormalities or signs of poor health will be noted and those animals will be excluded from randomization. Bodyweights will be used to randomize animals (using Excel random number generation) into designated study groups. Animals assigned to each study group (per sex) will be within 20% from the mean group value. Animals outside of this range may be replaced if suitable animals are available or as instructed by the study director. Animal numbers will be assigned to groups in sequential order following randomization.

10.3. Group Assignments: Randomized animals will be placed in the following groups:

Group Number	Dose Level	Dose Concentration	Dose Volume	Dosing Route	Total # of Animals	Day 3 Cohort	Day 15 Cohort
1	0.00 mg/kg (Vehicle)	0.00 mg/ml	5 ml/kg	IV	10 ♂<br>10 ♀	5 ♂<br>5 ♀	5 ♂<br>5 ♀
2	1.10 mg/kg	0.22 mg/ml	5 ml/kg	IV	10 ♂<br>10 ♀	5 ♂<br>5 ♀	5 ♂<br>5 ♂

10.4. Dosing Procedures: On Day 1 of the study, animals will be dosed once via IV injection with 5 ml/kg of the designated dose. Bodyweights collected prior to dosing (Day 1 bodyweights) will be used to calculate the individual dose volumes. Animals will be dosed using an appropriate sized needle attached to an appropriate sized syringe. All dosing procedures will be performed as per facility SOPs and documented accordingly.

10.5. Observations:
Mortality/Cageside: Animals will be observed once daily (AM) during the acclimation period prior to the study start. Beginning on study Day 1 and over the course of the study, animals will be observed twice daily (AM/PM) for morbidity, mortality, signs of toxicity and overall appearance. Observations and health assessments will be performed according to facility SOPs and will be documented on facility husbandry logs.

Detailed (hands-on): Physical hands-on clinical observations will be collected on Study Days 1 (all study animals), Day 2 (Day 3 cohort only; pre-fasting), Day 3 (Day 3 cohort only; post-fasting), Day 8, Day 14 (pre-fasting) and Day 15 (post-fasting). Animals will be palpated and observed for abnormal signs according to facility SOPs. Physical observations will be documented on the appropriate study form.

10.6. Bodyweights: Animals will have bodyweights collected on Study Days 1 (all study animals), Day 2 (Day 3 cohort only; pre-fasting), Day 3 (Day 3 cohort only; post-fasting), Day 8, Day 14 (pre-fasting) and Day 15 (post-fasting). Individual animal weights and balance calibration information will be documented on the appropriate study form. The Day 1 bodyweights will be used for calculating Day 1 dose volumes.

10.7. Blood Collection: Blood will be collected from each animal via an approved SOP method for clinical chemistry, hematology, and coagulation factors prior to euthanasia on the scheduled day of necropsy (Day 3 or Day 15). Animals will be food-fasted overnight prior to blood collection (up to 24 hours). Blood will be placed into individually labeled K2 EDTA tubes for hematology, serum separator tubes for clinical chemistry, and sodium citrate tubes for coagulation factors. The date, time, method of collection and type of anesthesia used (if applicable) will be documented on the collection form. Following blood collection, animals will be necropsied (refer to section 10.9 below). All blood samples will be refrigerated between 2-8°C following collection. Hematology (K2 EDTA) samples will be packaged on cold packs/wet ice and sent via overnight courier the same day as collection to the clinical pathology lab for processing. Serum separator and sodium citrate tubes will be centrifuged on the day of collection according to tube manufacturer specifications. Serum tubes will be allowed to clot prior to centrifuging. Serum/plasma will be drawn off and placed into individually labeled vials. Vials will be stored frozen at -20°C ±10°C until shipped on dry ice to the clinical pathology lab for processing. The clinical pathology lab used for this study is:

Bioanalytical Systems, Inc. (BASi)
Attn: Thea Riggs
10424 Middle Mount Vernon Road
Mount Vernon, IN 47620
Tel: 812-985-3400

Motivated By Discovery

The clinical pathology lab will perform the following tests on the blood samples:

Hematology

-Complete blood Count (CBC)

- red blood cell (erythrocyte) count
- hemoglobin
- hematocrit
- mean corpuscular volume
- mean corpuscular hemoglobin
- mean corpuscular hemoglobin concentration
- platelet count
- white blood cell (leukocyte) count
- differential blood cell count
- blood smear
- reticulocyte count

Clinical Chemistry

-glucose
-urea nitrogen
-creatinine
-total protein
-albumin
-globulin
-albumin/globulin ratio
-total cholesterol
-total bilirubin
-triglycerides
-alanine aminotransferase
-alkaline phosphatase
-gamma glutamyltransferase
-aspartate aminotransferase
-calcium
-inorganic phosphorus
-sodium
-potassium
-chloride

Coagulation Factors

- Prothrombin Time (PT)
- Activated Partial Thromboplastin Time (aPTT)
- Fibrinogen

The clinical pathology lab will provide a report indicating materials, methods and raw data for inclusion in the final study report.

10.8. Euthanasia:

Following blood collection on the scheduled day of necropsy, animals will be euthanized using CO2 overdose, followed by a secondary method (thoracotomy) to ensure death.

Animals exhibiting morbidity/morbundity prior to the schedule day of necropsy may be euthanized with study director approval. The animal will receive a full necropsy with all tissues saved for possible histological evaluation.

If an animal is found dead prior to the scheduled day of necropsy, a full necropsy will be conducted and tissues saved for possible histological evaluation.

10.9. Necropsy:

Following euthanasia, each animal will receive a comprehensive necropsy including organ weights for select tissues. Although only select tissues will be processed for microscopic evaluation, all tissues, including the remaining carcass and ear tag, will be placed in individually labeled containers containing 10% neutral-buffered formalin; with the exception of testis (males) and eyes with optic nerves which will be preserved in modified Davidson's fixative. Testis and eyes with optic nerves will be transferred from modified Davidson's to a suitable fixative (i.e. ethanol or 10% formalin) 1-2 days following the necropsy. The transfer will be documented in the histology records. Containers will be labeled with study number, date, group number, animal number, and Cohort (Day 3 or 15). Refer to the table below for specific tissues evaluated and weighed. Note- all paired organs will be weighed together.

Preserved tissues will be transferred to the processing lab where all tissues listed under "Tissues for Histology" will be embedded in paraffin, prepared to slide, hematoxylin and eosin (H&E) stained, and examined by a Pathologist for test-article related findings. Findings will be reported to the study director who will determine the course of action. All findings will be documented and provided in the pathology report. Tissues from early or unscheduled deaths and tissues not included on the list below will not be processed and examined unless requested by the study director or client.

SoBran INC. Motivated By Discovery

Tissues for Histology

- brain
- cecum
- colon
- eyes with optic nerves (2)
- heart
- ileum
- injection site
- kidneys (2)
- lesions (if present)
- liver
- lungs
- lymph node (mesenteric)
- ovaries (2) (females)
- salivary glands [mandibular (2)]
- spleen
- testes (2) (males)
- thyroid with parathyroid (2)
- trachea
- urinary bladder

Organ Weights Collected- at time of necropsy

- brain
- eyes with optic nerves
- heart
- kidneys
- liver
- lung
- ovaries (females)
- spleen
- testes (males)
- thyroid with parathyroid

Necropsy and organ weighing procedures will follow applicable facility SOPs. Note: lungs will be inflated with formalin following organ weight collection. The containers with fixatives and tissues will be stored at room temperature until shipped to following processing lab:

HSRL
5930 Main Street
Mount Jackson, VA 22842
Tel: 540-477-4440

The histology lab will provide a comprehensive report consisting of tabulated microscopic data and a discussion of noteworthy changes for inclusion in the final study report.

10.10. Animal Disposition:

Tissues and carcasses of animals are preserved as described above for histological evaluation following necropsy. Animal carcasses not saved (as directed by the study director or client) will be bagged and stored frozen until sent for incineration as medical pathological waste (MPW).

Motivated By Discovery

11. Data Analysis:

11.1. Body Weights and Organ Weights: Comprehensive statistical analysis (mean, standard deviations, N) will be conducted for individual group mean body weight and organ weight data in the final report using one-way Analysis of Variance (ANOVA) and Dunnett's t-test. The probability value of less than 0.05 (two-tailed) will be used as the critical level of significance for all tests.

Details regarding statistical software and additional methods used for bodyweight and organ weight data analysis will be further described in the final report.

11.2. Clinical Pathology Data: Statistical analysis of Clinical Pathology data will be conducted according to the following analysis:

A one-way analysis of variance (ANOVA) will be used to analyze the clinical pathology data; if the ANOVA is significant ($p \leq 0.05$), Dunnett's t-test (1955, 1964) will be used for control versus treated group comparisons.

Details regarding statistical software and additional methods used for clinical pathology data analysis will be further described in the final report.

12. Reports:

A comprehensive in-life study report will be prepared by SoBran following completion of the study. An audited draft report will be issued to the Sponsor within 8 weeks after completion of the in-life phase. The final report will be issued within 2 weeks after receipt of the Sponsor's review comments on the draft report. At finalization, one bound copy and one electronic copy of the final report will be sent to the Sponsor. SoBran retains the right to finalize the report if comments have not been received from the sponsor within 60 days of issuing the audited draft report.

13. Raw Data/Archives:

All raw data generated by the testing facility, any communication regarding the conduct of the study, and a copy of the final report will be stored in SoBran's archive room at our Biomedical Services office in Burtonsville, Maryland for 5 years following the issuance of the final report. Archiving of samples, tissues, blocks, slides, and subcontractor raw data and reports will be noted in the final study report. Residual samples of the test article formulations will be retained by SoBran as described in section 8.6 unless otherwise indicated.

Following the 5-year retention period, the sponsor will be contacted regarding the disposition of the archived documents. If the sponsor fails to respond within 60 days, SoBran reserves the right to destroy all data pertaining to this study.

14. Quality Assurance Oversight:

The Quality Assurance Unit (QAU) will periodically audit the study activities conducted within the test facility in accordance with the study protocol, facility SOPs and applicable GLP regulations. Study activities conducted outside of the test facility (i.e. subcontractors) will be audited accordingly by their QAUs with applicable reports submitted to the SoBran study director. A Quality Assurance Unit (QAU) review will be provided for the draft and final reports according to facility SOPs and GLPs.

15. Protocol Amendments and Deviations:

15.1. Amendments: All revisions to the approved protocol will be documented as protocol amendments. All amendments will be signed and dated by the study director, study, management and client/sponsor. Amendments will be maintained with the protocol. Written approval (i.e. an email directive from the study director approving the amendment) may be used to initiate the protocol change, but will be followed by an official signed amendment.

15.2. Deviations: All activities pertaining to this study, unless specifically defined in this protocol, will be performed according to SoBran SOPs. All deviations from the signed protocol, amendments, or SOPs will be documented in the study raw data and final report.

SoBran INC. Motivated By Discovery

16. Protocol Approval:

This protocol has been reviewed and approved.

Sponsor: [signature] Date: 09/25/2014
Frederick Chin, Ph.D.

SoBran Study Director: [signature] Date: 9/26/14
Adrienne Edgell, BS, CMAR, LATG

SoBran Study Management: [signature] Date: 9/[illegible]/14
Greg Kelly, Ph.D.

Motivated By Discovery

Protocol Amendment
Amendment: 1

14-Day Single Intravenous Dose Toxicity Study of [^{19}F]FP-R$_0$1-MG-F2 in Sprague Dawley Rats

1) **Section Amended:** Title Page and Study Title; Section 1, Pages 1-2

   Change as follows:
   Title was replaced with the following:

   14-Day Single Intravenous Dose Toxicity Study of [^{19}F]FP-R$_0$1-MG-F2 in Sprague Dawley Rats

   Justification: The actual test material contained [^{19}F] in place of the [^{18}F] radioactive component. Also the "zero" should be subscript to reflect the appropriate compound name.

2) **Section Amended:** Study Objectives; Section 2, Page 2

   Change as follows:
   The goal of this study is to assess the toxicity of [^{19}F]FP-R$_0$1-MG-F2 a small molecule (peptide) that is administered with a specific radioactive probe during Positron Emission Tomography (PET) scans to visualize tumors or targeted cells. For this study the toxicity of [^{19}F]FP-R$_0$1-MG-F2 (note - small molecule only, [^{19}F] is in place of the [^{18}F] radioactive component which will be used clinically) will be assessed following a single intravenous (IV) dose of 1.10 mg/kg (250x the clinical dose) of the test article. The vehicle (10% ethanol in normal saline) will also be tested to establish baseline toxicity.

   Justification: The actual test material contained [^{19}F] in place of the [^{18}F] radioactive component. Also the "zero" should be subscript to reflect the appropriate compound name.

3) **Section Amended:** Test Article; Section 8.1, Page 4

   Change as follows:
   Name: *[^{19}F]FP-R$_0$1-MG-F2*
   Supplier: Sponsor
   Lot Number(s): Noted on study forms and in final report
   Special Handling: Stored frozen (-20°C ±10°C) prior to formulation (as per sponsor); shipped and received from sponsor at ambient temperature

   Justification: The actual test material contained [^{19}F] in place of the [^{18}F] radioactive component. Also the "zero" should be subscript to reflect the appropriate compound name.

4) **Section Amended:** Study Summary; Section 9.7, Page 6

SoBran INC. **Motivated By Discovery**

Change as follows:

The goal of this study is to assess the toxicity of [^{19}F]FP-R$_0$1-MG-F2. For this study the toxicity of [^{19}F]FP-R^{0}1-MG-F2 (note - small molecule only, [^{19}F] is in place of [^{18}F] radioactive component) will be assessed following a single intravenous (IV) dose of 1.10 mg/kg (250x the clinical dose) of the test article. The vehicle (10% ethanol in normal saline) will also be tested to establish baseline toxicity. Half of the animals (20) will be euthanized 3 days following dosing, while the remaining will be euthanized at the end of the study (Day 15). Blood will be collected from all animals on the day of euthanasia, followed by a comprehensive necropsy. Blood and tissues will be evaluated for abnormalities.

Justification: The actual test material contained [^{19}F] in place of the [^{18}F] radioactive component. Also the "zero" should be subscript to reflect the appropriate compound name.

Amendment Approval

Sponsor: ____________ Date: 01/07/2015
Frederick Chin, Ph.D.

SoBran Study Director: ____________ Date: 2/2/15
Adrienne Edgell, BS, CMAR, LATG

SoBran Study Management: Gregory Kelly Ph.D. Date:
Digitally signed by Gregory Kelly Ph.D.
DN: cn=Gregory Kelly Ph.D., o=SoBran Inc., ou, email=gkelly@sobran-inc.com, c=US
Date: 2015.02.02 14:36:05 -05'00'
Greg Kelly, Ph.D.

Study Number: SB-SU-003 | Page 2 of 2 Amendment 1 | January 7, 2014

APPENDIX B – CLINICAL PATHOLOGY REPORT

AMENDED FINAL REPORT

CLINICAL PATHOLOGY EVALUATION FOR 14-DAY SINGLE INTRAVENOUS DOSE TOXICITY STUDY OF [^{19}F]FP-$R_0$1-MG-F2 IN SPRAGUE DAWLEY RATS

TEST SITE

BASi
10424 Middle Mt. Vernon Road
Mt. Vernon, IN 47620

BASI PROJECT NUMBER

1327-14292

SOBRAN STUDY NUMBER

SB-SU-003

SPONSOR

Stanford University School of Medicine
1201 Welch Road, Room PS049
Stanford, CA 94305-5484

PRINCIPAL INVESTIGATOR

T.A. Riggs AS, MLT (ASCP)

Report Completion: 24 November 2014
Report Amendment #1 Completion: 04 February 2015

BASi Project Number: 1327-14292 (SB-SU-003)

SIGNATURES

T.A. Riggs AS, MLT (ASCP)<br>Clinical Pathology Coordinator<br>BASi	24 Nov 14<br>Date
P. A. Downing, BA<br>Sr. Director, Preclinical Services<br>BASi	24Nov14<br>Date

i

BASi Project Number: 1327-14292 (SB-SU-003) BASi

STATEMENT OF REGULATORY COMPLIANCE

The nonclinical study described in this report was conducted in accordance with The United States Food and Drug Administration (US FDA) Good Laboratory Practice Regulations for Nonclinical Laboratory Studies (GLPs), 21 Code of Federal Regulations (CFR), Part 58.

This report accurately reflects the experimental data. No events occurred that were considered to have influenced the quality or integrity of the study.

Thea Riggs   24 Nov 14

T.A. Riggs AS, MLT (ASCP)   Date
Clinical Pathology Coordinator
BASi

ii

BASi Project Number: 1327-14292 (SB-SU-003)

QUALITY ASSURANCE STATEMENT

Study Title	Clinical Pathology Evaluation for 14-Day Single Intravenous Dose Toxicity Study of [^{19}F]FP-$R_0$1-MG-F2 in Sprague Dawley Rats
BASi Project No.	1327-14292

In accordance with BASi policy and quality assurance procedures for Good Laboratory Practice (GLP), this project has been audited and the conduct of this study has been inspected as follows:

Date of Inspection	Inspection	Date Reported to Study Director & Management
10 October 2014	Sample Receipt; Hematology Evaluation; Wedge Smear Preparation	10 October 2014
18 November 2014	Data; Draft under Circulation for Review and Comment	19 November 2014
24 November 2014	Final Report	24 November 2014

Quality Assurance Unit
Sandra J. Fox, BS, RQAP-GLP
Quality Assurance Manager
BASi

Signature Date

iii

TABLE OF CONTENTS

Signatures i
Statement of Regulatory Compliance ii
Quality Assurance Statement iii
Table of Contents iv
1. Test Site 1
2. Principal Investigator 1
3. Personnel 1
4. Study Design 1
5. Blood Sample Collection and Storage 2
 5.1. Hematology 2
 5.2. Coagulation 3
 5.3. Clinical Chemistry 3
6. Data Acquisition and Statistical Analysis 3
 6.1. Data Acquisition 3
 6.2. Statistical Analysis 3
7. Data and Specimen Retention 3
8. References 4

Tables:

1 - Summary of Hematology and Coagulation
2 - Summary of Clinical Chemistry

Appendices:

1 - Individual Hematology and Coagulation
2 - Individual Clinical Chemistry
3 - Report Amendment

BASi Project Number: 1327-14292 (SB-SU-003)

Clinical Pathology Evaluation for 14-Day Single Intravenous Dose Toxicity Study of [^{19}F]FP-R$_0$1-MG-F2 in Sprague Dawley Rats

1. TEST SITE

BASi
10424 Middle Mt. Vernon Road
Mt. Vernon, IN 47620 USA

2. PRINCIPAL INVESTIGATOR

T.A. Riggs AS, MLT (ASCP)
BASi
Phone: 812.985.5900 ext. 1122
Fax: 812.985.3403
Email: tariggs@basinc.com

3. PERSONNEL

Sr. Director, Preclinical Services
P. A. Downing, BA

Director of Toxicology
L.D. Hopper, DVM, PhD, DABT, RQAP-GLP

Clinical Pathology Coordinator
T.A. Riggs AS, MLT (ASCP)

Quality Assurance Manager
S. J. Fox, BS, RQAP-GLP

4. STUDY DESIGN

The goal of this study was to assess the toxicity of [^{19}F]FP-R$_0$1-MG-F2 a small molecule (peptide) that is administered with a specific radioactive probe during Positron Emission Tomography (PET) scans to visualize tumors or targeted cells. For this study the toxicity of [^{19}F]FP-R$_0$1-MG-F2 (note - small molecule only, no radioactive component) was assessed following a single intravenous (IV) dose of 1.10 mg/kg (250x the clinical dose) of the test article. The vehicle (10% ethanol in normal saline) was also tested to establish baseline toxicity.

Randomized animals were placed in the following groups:

Group Number	Dose Level	Dose Concentration	Dose Volume	Dosing Route	Total # of Animals	Day 3 Cohort	Day 15 Cohort
1	0.00 mg/kg (Vehicle)	0.00 mg/ml	5 ml/kg	IV	10M 10F	5M 5F	5M 5F
2	1.10 mg/kg	0.22 mg/ml	5 ml/kg	IV	10M 10F	5M 5F	5M 5F

5. BLOOD SAMPLE COLLECTION AND STORAGE

Blood samples were collected at SoBran.

Hematology samples were shipped on frozen ice packs or wet ice on the days of collection (Day 3: 09 October 2014 and Day 15: 21 October 2014) via overnight delivery to BASi. Upon arrival at BASi on 10 October 2014 and 22 October 2014, samples were inventoried and analyzed. Slides were prepared for all animals on the days of sample receipt. Specimens that were flagged by the analyzer for RBCs or platelets were reviewed for RBC and platelet morphology on 10 October 2014 (Day 3) and 23 October 2014 (Day 15). After analysis, samples were refrigerated until disposal.

Coagulation samples were shipped on dry ice on the final day of collection (Day 15: 21 October 2014, Day 3: 09 October 2014 included) via overnight delivery to BASi. Upon arrival at BASi, samples were inventoried, frozen at ≤-70°C, and analyzed on 23 October 2014 (Day 3) and 24 October 2014 (Day 15). After analysis, samples were discarded.

Clinical chemistry samples were shipped on dry ice on the final day of collection (Day 15: 21 October 2014, Day 3: 09 October 2014 included) via overnight delivery to BASi. Upon arrival at BASi, samples were inventoried, frozen at ≤-20°C, and analyzed on 30 October 2014. After analysis, samples were frozen at ≤-20°C until disposal.

All hematology samples collected exceeded the BASi established 24-hour stability for analysis. Review of manufacturer recommendation confirmed that samples are stable for longer than 24 hours with the exception of MCV, which may be effected by a longer storage time.

Coagulation sample quantities were not sufficient for fibrinogen analysis.

5.1. Hematology

Whole blood (approximately 0.50 mL) was collected in EDTA anticoagulant tubes from all surviving animals at scheduled euthanasia (Day 3 and Day 15) for determination of the following hematology parameters:

White blood cell (leukocyte) count	Mean corpuscular volume
Differential blood cell count	Mean corpuscular hemoglobin
Red blood cell (erythrocyte) count	Hemoglobin
Platelet count	Hematocrit
Blood smear	Mean corpuscular hemoglobin concentration
Reticulocyte Count	

5.2. Coagulation

Whole blood (approximately 0.70 mL) was collected in sodium citrate anticoagulant tubes from all surviving animals at scheduled euthanasia (Day 3 and Day 15) for determination of the following coagulation parameters:

Prothrombin time	Activated partial thromboplastin time

5.3. Clinical Chemistry

Whole blood (approximately 0.50 mL) was collected from all surviving animals at scheduled euthanasia (Day 3 and Day 15) and processed to serum for determination of the following clinical chemistry parameters:

Sodium	Total cholesterol
Potassium	Triglycerides
Chloride	Total protein
Alkaline phosphatase	Albumin
Alanine aminotransferase	Globulin (calculated)
Aspartate aminotransferase	Albumin/globulin ratio (calculated)
Glucose	Calcium
Blood urea nitrogen	Inorganic phosphorus
Creatinine	Total bilirubin
Gamma glutamyltransferase	

6. DATA ACQUISITION AND STATISTICAL ANALYSIS

6.1. Data Acquisition

Clinical pathology data were collected on the ADVIA120, ADVIA1800, and Diagnostica STAGO Coagulation Analyzer and key-punched into Paradox for reporting and statistical analysis.

6.2. Statistical Analysis

Statistical analysis of clinical pathology data was conducted according to the following analysis:

A one-way analysis of variance (ANOVA) was used to analyze the clinical pathology data; if the ANOVA was significant ($p \leq 0.05$), Dunnett's t-test (1955, 1964) was used for control versus treated group comparisons.

7. DATA AND SPECIMEN RETENTION

All original raw data, documentation, and the original final report originating from this test site will be retained at BASi or an approved archive facility for a period of two years following completion of the study (final report issue date). After the two-year retention period, study materials will be transferred to

SoBran or SoBran's designated archive facility, or may be retained at BASi based on a contractual agreement between SoBran and BASi.

All slides from this study may be stored at BASi for a period of four months following completion of the BASi report. After the four-month retention period, the slides may be transferred to SoBran or SoBran's designated archive facility, or may be retained at BASi based on a contractual agreement between SoBran and BASi.

All time-sensitive biological specimens from this study were discarded following analysis.

8. REFERENCES

Dunnett CW. New tables for multiple comparisons with a control. Biometrics 1964; 20:482-92.

Dunnett CW. A multiple comparison procedure for comparing several treatments with a control. J Amer Statis Assoc 1955; 50:1096-121.

BASi Project Number: 1327-14292 (SB-SU-003)

TABLES

BASi Project Number: 1327-14292 (SB-SU-003)

TABLE 1
Key to Summary of Hematology and Coagulation

Dosage Key

Group Number	Dose Level	Dose Conc.	Dose Volume	Dosing Route	Total # of Animals	Day 3 Cohort	Day 15 Cohort
1	0.00 mg/kg (vehicle)	0.00 mg/ml	5 ml/kg	IV	10M 10F	5M 5F	5M 5F
2	1.10 mg/kg	0.22 mg/ml	5 ml/kg	IV	10M 10F	5M 5F	5M 5F

Abbreviations

WBC	White blood cell count	x 10^3 cells/μL
ANE	Absolute neutrophil count	x 10^3 cells/μL
ALY	Absolute lymphocyte count	x 10^3 cells/μL
AMO	Absolute monocyte count	x 10^3 cells/μL
AEO	Absolute eosinophil count	x 10^3 cells/μL
ABA	Absolute basophil count	x 10^3 cells/μL
ALUC	Absolute large unstained cell count	x 10^3 cells/μL
RBC	Red blood cell count	x 10^6 cells/μL
HB	Hemoglobin	g/dL
HCT	Hematocrit	Percent
MCV	Mean corpuscular volume	fL
MCH	Mean corpuscular hemoglobin	pg
MCHC	Mean corpuscular hemoglobin concentration	g/dL
PLT	Platelet count	x 10^3 cells/μL
Retic	Reticulocyte count	Percent
PT	Prothrombin time	Seconds
APTT	Activated partial thromboplastin time	Seconds
FIB	Fibrinogen	mg/dL

Dunnett's Test Key

* = .05 Significance by Dunnett's Test
+ = .01 Significance by Dunnett's Test
< = Less than 5 degrees of freedom by Dunnett's Test
Ç = Control group used for Dunnett's Test comparison

BASi Project Number: 1327-14292 (SB-SU-003)

TABLE 1
Summary of Hematology and Coagulation
Males

	Group Number		WBC Day 3	WBC Day 15	ANE Day 3	ANE Day 15	ALY Day 3	ALY Day 15
Ç	1M	Mn:	10.15	12.50	1.73	1.93	7.88	9.94
Ç		SD:	2.19	1.14	.31	.38	1.91	1.14
Ç		N:	5	5	5	5	5	5
	2M	Mn:	13.10*	11.31	1.80	1.48	10.47*	9.33
		SD:	1.61	1.29	.35	.34	1.31	.90
		N:	5	5	5	5	5	5

	Group Number		AMO Day 3	AMO Day 15	AEO Day 3	AEO Day 15	ABA Day 3	ABA Day 15
Ç	1M	Mn:	.33	.42	.10	.07	.04	.07
Ç		SD:	.09	.10	.06	.01	.02	.03
Ç		N:	5	5	5	5	5	5
	2M	Mn:	.58+	.29	.08	.07	.06	.06
		SD:	.08	.10	.03	.02	.02	.02
		N:	5	5	5	5	5	5

	Group Number		ALUC Day 3	ALUC Day 15	RBC Day 3	RBC Day 15	HB Day 3	HB Day 15
Ç	1M	Mn:	.07	.09	7.24	8.17	14.3	15.4
Ç		SD:	.02	.01	.67	.11	1.5	.4
Ç		N:	5	5	5	5	5	5
	2M	Mn:	.11+	.08	7.53	8.05	14.6	15.6
		SD:	.02	.02	.16	.14	.4	.2
		N:	5	5	5	5	5	5

1

BASi Project Number: 1327-14292 (SB-SU-003)

TABLE 1
Summary of Hematology and Coagulation
Males

Group Number			HCT Day 3	HCT Day 15	MCV Day 3	MCV Day 15	MCH Day 3	MCH Day 15
Ç	1M	Mn:	44.6	46.8	61.6	57.3	19.7	18.8
Ç		SD:	4.6	.8	1.4	.8	.4	.3
Ç		N:	5	5	5	5	5	5
	2M	Mn:	45.8	47.0	60.9	58.4	19.4	19.3*
		SD:	1.6	.8	2.1	1.1	.6	.3
		N:	5	5	5	5	5	5

Group Number			MCHC Day 3	MCHC Day 15	PLT Day 3	PLT Day 15	Retic Day 3	Retic Day 15
Ç	1M	Mn:	31.9	32.9	1294	1142	5.66	4.25
Ç		SD:	.3	.4	68	134	.71	.37
Ç		N:	5	5	5	5	5	5
	2M	Mn:	31.9	33.0	1350	1145	6.24	4.41
		SD:	.4	.2	319	89	2.23	.43
		N:	5	5	5	5	5	5

Group Number			PT Day 3	PT Day 15	APTT Day 3	APTT Day 15	FIB Day 3	FIB Day 15
Ç	1M	Mn:	17.1	15.8	20.0	21.0	-	-
Ç		SD:	1.6	.8	2.6	10.9	-	-
Ç		N:	4	5	5	5	0	0
	2M	Mn:	17.8	15.5	21.8	24.5	-	-
		SD:	.8	.7	6.0	12.1	-	-
		N:	4	5	3	5	0	0

2

BASi Project Number: 1327-14292 (SB-SU-003)

TABLE 1
Summary of Hematology and Coagulation
Females

Group Number			WBC Day 3	WBC Day 15	ANE Day 3	ANE Day 15	ALY Day 3	ALY Day 15
Ç	1F	Mn:	6.44	9.11	1.14	1.76	5.02	6.96
Ç		SD:	.65	1.89	.45	.52	.69	1.45
Ç		N:	5	5	5	5	5	5
	2F	Mn:	6.56	8.43	.80	1.14	5.48	6.95
		SD:	1.72	1.50	.39	.33	1.50	1.14
		N:	5	5	5	5	5	5

Group Number			AMO Day 3	AMO Day 15	AEO Day 3	AEO Day 15	ABA Day 3	ABA Day 15
Ç	1F	Mn:	.14	.16	.08	.14	.02	.04
Ç		SD:	.04	.04	.02	.05	.02	.02
Ç		N:	5	5	5	5	5	5
	2F	Mn:	.11	.14	.07	.12	.05	.04
		SD:	.03	.06	.03	.04	.03	.01
		N:	5	5	5	5	5	5

Group Number			ALUC Day 3	ALUC Day 15	RBC Day 3	RBC Day 15	HB Day 3	HB Day 15
Ç	1F	Mn:	.04	.05	7.49	7.74	14.6	14.9
Ç		SD:	.02	.01	.13	.14	.3	.3
Ç		N:	5	5	5	5	5	5
	2F	Mn:	.04	.05	7.35	7.74	14.3	15.0
		SD:	.01	.02	.35	.09	.6	.3
		N:	5	5	5	5	5	5

3

BASi Project Number: 1327-14292 (SB-SU-003)

TABLE 1
Summary of Hematology and Coagulation
Females

	Group Number		HCT Day 3	HCT Day 15	MCV Day 3	MCV Day 15	MCH Day 3	MCH Day 15
Ç	1F	Mn:	44.0	43.8	58.8	56.5	19.5	19.3
Ç		SD:	1.2	.5	.8	.6	.2	.3
Ç		N:	5	5	5	5	5	5
	2F	Mn:	43.4	44.1	59.1	56.9	19.5	19.3
		SD:	1.6	.7	1.0	.8	.4	.3
		N:	5	5	5	5	5	5

	Group Number		MCHC Day 3	MCHC Day 15	PLT Day 3	PLT Day 15	Retic Day 3	Retic Day 15
Ç	1F	Mn:	33.1	34.1	1411	1169	3.34	2.89
Ç		SD:	.5	.4	154	124	.60	.93
Ç		N:	5	5	5	5	5	5
	2F	Mn:	32.9	33.9	1416	1175	4.01	3.13
		SD:	.8	.3	152	103	.90	.60
		N:	5	5	5	5	5	5

	Group Number		PT Day 3	PT Day 15	APTT Day 3	APTT Day 15	FIB Day 3	FIB Day 15
Ç	1F	Mn:	16.8	16.3	27.4	18.8	-	-
Ç		SD:	.9	1.3	.0	2.9	-	-
Ç		N:	5	4	1	4	0	0
	2F	Mn:	16.5	15.7	20.8<	14.1*	-	-
		SD:	.6	1.3	3.5	.9	-	-
		N:	3	5	3	4	0	0

4

BASi Project Number: 1327-14292 (SB-SU-003)

TABLE 2

Key to Summary of Clinical Chemistry

Dosage Key

Group Number	Dose Level	Dose Conc.	Dose Volume	Dosing Route	Total # of Animals	Day 3 Cohort	Day 15 Cohort
1	0.00 mg/kg (vehicle)	0.00 mg/ml	5 ml/kg	IV	10M 10F	5M 5F	5M 5F
2	1.10 mg/kg	0.22 mg/ml	5 ml/kg	IV	10M 10F	5M 5F	5M 5F

Abbreviations

NA	Sodium	mEq/L
K	Potassium	mEq/L
CL	Chloride	mEq/L
ALB	Albumin	g/dL
ALP	Alkaline phosphatase	U/L
ALT	Alanine aminotransferase	U/L
AST	Aspartate aminotransferase	U/L
BUN	Blood urea nitrogen	mg/dL
CA	Calcium	mg/dL
CHOL	Total cholesterol	mg/dL
CRE	Creatinine	mg/dL
GGT	Gamma Glutamyltransferase	U/L
GLU	Glucose	mg/dL
PHOS	Inorganic phosphorus	mg/dL
TBIL	Total bilirubin	mg/dL
TP	Total protein	g/dL
TRIG	Triglycerides	mg/dL
GLOB	Globulin (calculated)	g/dL
A/G	Albumin/globulin ratio (calculated)	--

Dunnett's Test Key

* = .05 Significance by Dunnett's Test
+ = .01 Significance by Dunnett's Test
< = Less than 5 degrees of freedom by Dunnett's Test
Ç = Control group used for Dunnett's Test comparison

BASi Project Number: 1327-14292 (SB-SU-003)

TABLE 2
Summary of Clincial Chemistry
Males

	Group Number		NA Day 3	NA Day 15	K Day 3	K Day 15	CL Day 3	CL Day 15
Ç	1M	Mn:	143	142	5.1	5.1	102	103
Ç		SD:	1	1	.3	.2	1	1
Ç		N:	5	5	5	5	5	5
	2M	Mn:	142	142	5.4	4.9	102	102
		SD:	2	0	.2	.3	1	1
		N:	5	5	5	5	5	5

	Group Number		ALB Day 3	ALB Day 15	ALP Day 3	ALP Day 15	ALT Day 3	ALT Day 15
Ç	1M	Mn:	3.5	3.8	235	190	64	68
Ç		SD:	.3	.1	19	21	6	4
Ç		N:	5	5	5	5	5	5
	2M	Mn:	3.6	3.8	252	247*	77+	80*
		SD:	.1	.1	9	36	6	9
		N:	5	5	5	5	5	5

	Group Number		AST Day 3	AST Day 15	BUN Day 3	BUN Day 15	CA Day 3	CA Day 15
Ç	1M	Mn:	125	107	16	17	9.9	9.9
Ç		SD:	16	7	5	2	.3	.1
Ç		N:	5	5	5	5	5	5
	2M	Mn:	115	111	15	20	10.0	9.9
		SD:	10	9	2	3	.3	.1
		N:	5	5	5	5	5	5

1

BASi Project Number: 1327-14292 (SB-SU-003)

TABLE 2
Summary of Clincial Chemistry
Males

Group Number			CHOL Day 3	CHOL Day 15	CRE Day 3	CRE Day 15	GGT Day 3	GGT Day 15
Ç	1M	Mn:	130	114	.26	.31	8	8
Ç		SD:	16	9	.06	.03	0	0
Ç		N:	5	5	5	5	5	5
	2M	Mn:	142	118	.23	.29	8	8
		SD:	11	8	.02	.01	0	0
		N:	5	5	5	5	5	5

Group Number			GLU Day 3	GLU Day 15	PHOS Day 3	PHOS Day 15	TBIL Day 3	TBIL Day 15
Ç	1M	Mn:	104	98	9.3	8.6	.07	.07
Ç		SD:	40	9	.5	.5	.01	.01
Ç		N:	5	5	5	5	5	5
	2M	Mn:	102	105	9.4	8.3	.10	.07
		SD:	23	17	.6	.3	.03	.03
		N:	5	5	5	5	5	5

Group Number			TP Day 3	TP Day 15	TRIG Day 3	TRIG Day 15	GLOB Day 3	GLOB Day 15
Ç	1M	Mn:	5.7	6.3	76	70	2.2	2.4
Ç		SD:	.5	.2	16	26	.2	.1
Ç		N:	5	5	5	5	5	5
	2M	Mn:	5.9	6.2	84	84	2.3	2.4
		SD:	.1	.2	34	25	.1	.1
		N:	5	5	5	5	5	5

2

BASi Project Number: 1327-14292 (SB-SU-003)

TABLE 2
Summary of Clincial Chemistry
Males

Group Number			A/G Day 3	A/G Day 15
Ç	1M	Mn:	1.6	1.6
Ç		SD:	.0	.1
Ç		N:	5	5
	2M	Mn:	1.6	1.6
		SD:	.1	.1
		N:	5	5

3

BASi Project Number: 1327-14292 (SB-SU-003)

TABLE 2
Summary of Clincial Chemistry
Females

	Group Number		NA Day 3	NA Day 15	K Day 3	K Day 15	CL Day 3	CL Day 15
Ç	1F	Mn:	141	141	4.7	4.5	104	104
Ç		SD:	1	1	.5	.3	1	0
Ç		N:	5	5	5	5	5	5
	2F	Mn:	142	141	4.7	4.5	105	104
		SD:	1	1	.2	.2	1	2
		N:	5	5	5	5	5	5

	Group Number		ALB Day 3	ALB Day 15	ALP Day 3	ALP Day 15	ALT Day 3	ALT Day 15
Ç	1F	Mn:	3.9	3.9	146	123	44	56
Ç		SD:	.2	.1	20	22	5	9
Ç		N:	5	5	5	5	5	5
	2F	Mn:	3.8	3.9	146	141	51	57
		SD:	.1	.2	26	14	6	8
		N:	5	5	5	5	5	5

	Group Number		AST Day 3	AST Day 15	BUN Day 3	BUN Day 15	CA Day 3	CA Day 15
Ç	1F	Mn:	103	112	20	20	9.9	9.7
Ç		SD:	11	12	6	2	.2	.1
Ç		N:	5	5	5	5	5	5
	2F	Mn:	110	110	20	21	9.7	9.9
		SD:	9	7	2	1	.1	.2
		N:	5	5	5	5	5	5

BASi Project Number: 1327-14292 (SB-SU-003)

TABLE 2
Summary of Clincial Chemistry
Females

	Group Number		CHOL Day 3	CHOL Day 15	CRE Day 3	CRE Day 15	GGT Day 3	GGT Day 15
Ç	1F	Mn:	130	106	.28	.32	8	8
Ç		SD:	18	13	.08	.03	0	0
Ç		N:	5	5	5	5	5	5
	2F	Mn:	132	116	.27	.34	8	8
		SD:	4	8	.02	.05	0	0
		N:	5	5	5	5	5	5

	Group Number		GLU Day 3	GLU Day 15	PHOS Day 3	PHOS Day 15	TBIL Day 3	TBIL Day 15
Ç	1F	Mn:	88	99	7.2	6.3	.05	.10
Ç		SD:	10	10	.6	.5	.01	.01
Ç		N:	5	5	5	5	5	5
	2F	Mn:	106	103	7.2	6.5	.05	.08
		SD:	18	13	.4	.7	.00	.02
		N:	5	5	5	5	5	5

	Group Number		TP Day 3	TP Day 15	TRIG Day 3	TRIG Day 15	GLOB Day 3	GLOB Day 15
Ç	1F	Mn:	6.2	6.2	55	44	2.3	2.3
Ç		SD:	.4	.2	16	12	.1	.1
Ç		N:	5	5	5	5	5	5
	2F	Mn:	6.1	6.3	41	64	2.3	2.4
		SD:	.2	.3	11	22	.1	.2
		N:	5	5	5	5	5	5

5

BASi Project Number: 1327-14292 (SB-SU-003)

TABLE 2
Summary of Clincial Chemistry
Females

Group Number			A/G Day 3	A/G Day 15
Ç	1F	Mn:	1.7	1.7
Ç		SD:	.0	.0
Ç		N:	5	5
	2F	Mn:	1.7	1.6
		SD:	.1	.1
		N:	5	5

6

Individual Hematology and Coagulation

BASi Study Number: 1327-14292 (SB-SU-003) Appendix No. 1

Key to Individual Hematology and Coagulation

Dosage Key

Group Number	Dose Level	Dose Conc.	Dose Volume	Dosing Route	Total # of Animals	Day 3 Cohort	Day 15 Cohort
1	0.00 mg/kg (vehicle)	0.00 mg/ml	5 ml/kg	IV	10M 10F	5M 5F	5M 5F
2	1.10 mg/kg	0.22 mg/ml	5 ml/kg	IV	10M 10F	5M 5F	5M 5F

Abbreviations

WBC	White blood cell count	x 10^3 cells/μL
ANE	Absolute neutrophil count	x 10^3 cells/μL
ALY	Absolute lymphocyte count	x 10^3 cells/μL
AMO	Absolute monocyte count	x 10^3 cells/μL
AEO	Absolute eosinophil count	x 10^3 cells/μL
ABA	Absolute basophil count	x 10^3 cells/μL
ALUC	Absolute large unstained cell count	x 10^3 cells/μL
RBC	Red blood cell count	x 10^6 cells/μL
HB	Hemoglobin	g/dL
HCT	Hematocrit	Percent
MCV	Mean cell volume	fL
MCH	Mean cell hemoglobin	pg
MCHC	Mean cell hemoglobin concentration	g/dL
PLT	Platelet count	x 10^3 cells/μL
Retic	Reticulocyte count	Percent
PT	Prothrombin time	Seconds
APTT	Activated partial thromboplastin time	Seconds
FIB	Fibrinogen	mg/dL

Report Code Key

A = Absent values and/or additional information
Q = Quantity not sufficient
- = Animal not sampled at time point
X = Animal dead
1+ = 10-25%
3+ = 51-75%

Key to Individual Hematology and Coagulation (cont.)

Absent Values and/or Additional Information

Day 3

1M801	3+ platelet clumping noted on slide
1F827	Few platelet clumps noted on slide
2F835	Few platelet clumps noted on slide

The following animals had elevated values, but insufficient quantity for confirmation. These values were not included in the report.

Animal No.	APTT	PT
2M811	61.2	
1F825	57.9	
1F826	42.1	
1F827	50.9	
2F835	35.0	
2F833		37.3

Day 15

1M809	1+ platelet clumping noted on slide
1M810	Few platelet clumps noted on slide
2M816	Few platelet clumps noted on slide
2M818	1+ platelet clumping noted on slide
2M819	Few platelet clumps noted on slide
2M820	Few platelet clumps noted on slide
1F831	1+ platelet clumping noted on slide
1F832	Few platelet clumps noted on slide

The following animals had elevated values, but insufficient quantity for confirmation. These values were not included in the report.

Animal No.	APTT	PT
1F830		20.6
2F838	33.8	

Dunnett's Test Key

* = .05 Significance by Dunnett's Test
+ = .01 Significance by Dunnett's Test
< = Less than 5 degrees of freedom by Dunnett's Test
Ç = Control group used for Dunnett's Test comparison

Individual Hematology and Coagulation
Males

Animal Number		WBC Day 3	WBC Day 15	ANE Day 3	ANE Day 15	ALY Day 3	ALY Day 15
1M	801	8.58	X	1.38	X	6.75	X
1M	802	8.30	X	1.90	X	5.88	X
1M	803	10.44	X	1.52	X	8.37	X
1M	804	13.75	X	2.17	X	10.88	X
1M	805	9.67	X	1.67	X	7.49	X
1M	806	-	13.27	-	1.38	-	11.35
1M	807	-	10.93	-	1.74	-	8.63
1M	808	-	12.40	-	1.97	-	9.77
1M	809	-	13.88	-	2.28	-	10.84
1M	810	-	12.04	-	2.26	-	9.10
Ç 1M	Mn:	10.15	12.50	1.73	1.93	7.88	9.94
2M	811	11.00	X	1.47	X	8.68	X
2M	812	13.75	X	1.76	X	11.23	X
2M	813	11.97	X	1.59	X	9.50	X
2M	814	13.74	X	1.79	X	11.16	X
2M	815	15.05	X	2.38	X	11.77	X
2M	816	-	12.42	-	1.89	-	9.97
2M	817	-	10.61	-	1.06	-	9.10
2M	818	-	12.90	-	1.77	-	10.45
2M	819	-	9.85	-	1.38	-	8.16
2M	820	-	10.78	-	1.28	-	8.99
2M	Mn:	13.10*	11.31	1.80	1.48	10.47*	9.33

BASi Study Number: 1327-14292 (SB-SU-003) Appendix No. 1

Individual Hematology and Coagulation
Males

Animal Number	AMO Day 3	AMO Day 15	AEO Day 3	AEO Day 15	ABA Day 3	ABA Day 15
1M 801	.19	X	.20	X	.02	X
1M 802	.37	X	.05	X	.03	X
1M 803	.34	X	.07	X	.05	X
1M 804	.43	X	.11	X	.07	X
1M 805	.33	X	.06	X	.04	X
1M 806	-	.31	-	.08	-	.05
1M 807	-	.37	-	.05	-	.05
1M 808	-	.37	-	.09	-	.12
1M 809	-	.57	-	.06	-	.06
1M 810	-	.46	-	.07	-	.06
Ç 1M Mn:	.33	.42	.10	.07	.04	.07
2M 811	.66	X	.06	X	.04	X
2M 812	.51	X	.10	X	.07	X
2M 813	.66	X	.05	X	.05	X
2M 814	.48	X	.12	X	.07	X
2M 815	.59	X	.09	X	.09	X
2M 816	-	.34	-	.07	-	.06
2M 817	-	.25	-	.06	-	.03
2M 818	-	.43	-	.09	-	.05
2M 819	-	.16	-	.04	-	.09
2M 820	-	.27	-	.10	-	.08
2M Mn:	.58+	.29	.08	.07	.06	.06

2

BASi Study Number: 1327-14292 (SB-SU-003) Appendix No. 1

Individual Hematology and Coagulation
Males

Animal Number		ALUC Day 3	ALUC Day 15	RBC Day 3	RBC Day 15	HB Day 3	HB Day 15
1M	801	.04	X	7.73	X	15.2	X
1M	802	.06	X	6.06	X	11.7	X
1M	803	.06	X	7.32	X	14.7	X
1M	804	.10	X	7.53	X	15.2	X
1M	805	.08	X	7.54	X	14.5	X
1M	806	-	.11	-	7.97	-	14.9
1M	807	-	.07	-	8.22	-	15.7
1M	808	-	.09	-	8.20	-	15.1
1M	809	-	.08	-	8.23	-	15.8
1M	810	-	.08	-	8.22	-	15.6
Ç 1M	Mn:	.07	.09	7.24	8.17	14.3	15.4
2M	811	.10	X	7.56	X	14.6	X
2M	812	.10	X	7.71	X	14.5	X
2M	813	.12	X	7.48	X	15.2	X
2M	814	.11	X	7.28	X	14.1	X
2M	815	.14	X	7.60	X	14.5	X
2M	816	-	.09	-	7.95	-	15.4
2M	817	-	.10	-	7.94	-	15.5
2M	818	-	.10	-	7.97	-	15.6
2M	819	-	.05	-	8.13	-	15.4
2M	820	-	.08	-	8.26	-	15.9
2M	Mn:	.11+	.08	7.53	8.05	14.6	15.6

3

Individual Hematology and Coagulation
Males

Animal Number		HCT Day 3	HCT Day 15	MCV Day 3	MCV Day 15	MCH Day 3	MCH Day 15
1M	801	48.5	X	62.7	X	19.7	X
1M	802	36.8	X	60.6	X	19.3	X
1M	803	45.5	X	62.2	X	20.0	X
1M	804	47.3	X	62.9	X	20.2	X
1M	805	45.1	X	59.8	X	19.3	X
1M	806	-	46.1	-	57.9	-	18.7
1M	807	-	47.3	-	57.6	-	19.0
1M	808	-	45.8	-	55.8	-	18.4
1M	809	-	47.4	-	57.6	-	19.2
1M	810	-	47.2	-	57.4	-	18.9
Ç 1M	Mn:	44.6	46.8	61.6	57.3	19.7	18.8
2M	811	45.2	X	59.9	X	19.3	X
2M	812	46.0	X	59.7	X	18.8	X
2M	813	48.4	X	64.7	X	20.4	X
2M	814	44.0	X	60.4	X	19.4	X
2M	815	45.4	X	59.8	X	19.1	X
2M	816	-	46.8	-	58.9	-	19.3
2M	817	-	46.5	-	58.6	-	19.5
2M	818	-	47.5	-	59.6	-	19.6
2M	819	-	46.1	-	56.7	-	18.9
2M	820	-	48.1	-	58.3	-	19.2
2M	Mn:	45.8	47.0	60.9	58.4	19.4	19.3*

Individual Hematology and Coagulation
Males

Animal Number		MCHC Day 3	MCHC Day 15	PLT Day 3	PLT Day 15	Retic Day 3	Retic Day 15
1M	801	31.4	X	1352A	X	5.51	X
1M	802	31.8	X	1380	X	5.42	X
1M	803	32.2	X	1231	X	5.01	X
1M	804	32.1	X	1266	X	6.88	X
1M	805	32.2	X	1241	X	5.46	X
1M	806	-	32.3	-	1205	-	4.49
1M	807	-	33.1	-	1168	-	4.76
1M	808	-	32.9	-	1018	-	3.88
1M	809	-	33.3	-	1318A	-	4.14
1M	810	-	32.9	-	999A	-	3.99
Ç 1M	Mn:	31.9	32.9	1294	1142	5.66	4.25
2M	811	32.3	X	984	X	5.22	X
2M	812	31.5	X	1628	X	5.38	X
2M	813	31.5	X	1108	X	10.17	X
2M	814	32.1	X	1720	X	5.75	X
2M	815	32.0	X	1311	X	4.68	X
2M	816	-	32.8	-	1121A	-	4.17
2M	817	-	33.2	-	1168	-	4.80
2M	818	-	32.8	-	1005A	-	4.92
2M	819	-	33.3	-	1199A	-	3.92
2M	820	-	33.0	-	1234A	-	4.24
2M	Mn:	31.9	33.0	1350	1145	6.24	4.41

BASi Study Number: 1327-14292 (SB-SU-003) Appendix No. 1

Individual Hematology and Coagulation
Males

Animal Number		PT Day 3	PT Day 15	APTT Day 3	APTT Day 15	FIB Day 3	FIB Day 15
1M	801	Q	X	22.1	X	Q	X
1M	802	19.4	X	21.1	X	Q	X
1M	803	16.5	X	22.0	X	Q	X
1M	804	16.5	X	19.0	X	Q	X
1M	805	16.0	X	15.9	X	Q	X
1M	806	-	14.8	-	10.5	-	Q
1M	807	-	15.6	-	38.1	-	Q
1M	808	-	15.9	-	19.6	-	Q
1M	809	-	17.1	-	13.2	-	Q
1M	810	-	15.6	-	23.4	-	Q
Ç 1M	Mn:	17.1	15.8	20.0	21.0	-	-
2M	811	18.5	X	Q	X	Q	X
2M	812	17.0	X	Q	X	Q	X
2M	813	18.4	X	18.1	X	Q	X
2M	814	17.3	X	28.7	X	Q	X
2M	815	Q	X	18.6	X	Q	X
2M	816	-	16.3	-	20.1	-	Q
2M	817	-	15.0	-	16.4	-	Q
2M	818	-	15.2	-	45.9	-	Q
2M	819	-	14.8	-	19.5	-	Q
2M	820	-	16.0	-	20.5	-	Q
2M	Mn:	17.8	15.5	21.8	24.5	-	-

6

BASi Study Number: 1327-14292 (SB-SU-003) Appendix No. 1

Individual Hematology and Coagulation
Females

Animal Number		WBC Day 3	WBC Day 15	ANE Day 3	ANE Day 15	ALY Day 3	ALY Day 15
1F	823	6.96	X	1.36	X	5.28	X
1F	824	6.09	X	1.78	X	4.02	X
1F	825	5.61	X	.74	X	4.65	X
1F	826	6.33	X	.72	X	5.32	X
1F	827	7.20	X	1.08	X	5.82	X
1F	828	-	9.57	-	1.74	-	7.48
1F	829	-	8.62	-	1.84	-	6.24
1F	830	-	12.05	-	2.53	-	9.03
1F	831	-	6.95	-	1.55	-	5.14
1F	832	-	8.36	-	1.11	-	6.89
Ç 1F	Mn:	6.44	9.11	1.14	1.76	5.02	6.96
2F	833	4.38	X	.55	X	3.63	X
2F	834	6.29	X	.52	X	5.50	X
2F	835	5.90	X	1.10	X	4.47	X
2F	836	9.05	X	1.34	X	7.39	X
2F	837	7.19	X	.50	X	6.44	X
2F	838	-	9.91	-	1.15	-	8.30
2F	839	-	8.33	-	1.15	-	6.93
2F	840	-	6.69	-	.84	-	5.57
2F	841	-	7.25	-	.90	-	6.10
2F	842	-	9.98	-	1.68	-	7.81
2F	Mn:	6.56	8.43	.80	1.14	5.48	6.95

7

BASi Study Number: 1327-14292 (SB-SU-003) Appendix No. 1

Individual Hematology and Coagulation
Females

Animal Number		AMO Day 3	AMO Day 15	AEO Day 3	AEO Day 15	ABA Day 3	ABA Day 15
1F	823	.19	X	.09	X	.02	X
1F	824	.16	X	.09	X	.02	X
1F	825	.12	X	.04	X	.02	X
1F	826	.15	X	.08	X	.01	X
1F	827	.09	X	.10	X	.05	X
1F	828	-	.15	-	.12	-	.03
1F	829	-	.22	-	.20	-	.06
1F	830	-	.18	-	.19	-	.06
1F	831	-	.11	-	.10	-	.02
1F	832	-	.14	-	.11	-	.04
Ç 1F	Mn:	.14	.16	.08	.14	.02	.04
2F	833	.09	X	.06	X	.03	X
2F	834	.09	X	.05	X	.08	X
2F	835	.17	X	.06	X	.06	X
2F	836	.10	X	.12	X	.05	X
2F	837	.12	X	.07	X	.01	X
2F	838	-	.20	-	.14	-	.05
2F	839	-	.10	-	.07	-	.03
2F	840	-	.09	-	.13	-	.03
2F	841	-	.09	-	.09	-	.03
2F	842	-	.20	-	.19	-	.04
2F	Mn:	.11	.14	.07	.12	.05	.04

8

Individual Hematology and Coagulation
Females

Animal Number		ALUC Day 3	ALUC Day 15	RBC Day 3	RBC Day 15	HB Day 3	HB Day 15
1F	823	.02	X	7.64	X	14.7	X
1F	824	.02	X	7.31	X	14.1	X
1F	825	.03	X	7.59	X	14.9	X
1F	826	.05	X	7.42	X	14.6	X
1F	827	.06	X	7.49	X	14.6	X
1F	828	-	.04	-	7.62	-	14.5
1F	829	-	.06	-	7.98	-	15.1
1F	830	-	.05	-	7.67	-	14.8
1F	831	-	.03	-	7.69	-	14.9
1F	832	-	.06	-	7.75	-	15.2
Ç 1F	Mn:	.04	.05	7.49	7.74	14.6	14.9
2F	833	.02	X	7.19	X	14.5	X
2F	834	.05	X	7.18	X	13.9	X
2F	835	.04	X	7.54	X	14.4	X
2F	836	.05	X	6.98	X	13.5	X
2F	837	.04	X	7.85	X	15.2	X
2F	838	-	.07	-	7.66	-	14.6
2F	839	-	.04	-	7.70	-	15.3
2F	840	-	.03	-	7.82	-	14.9
2F	841	-	.03	-	7.69	-	14.9
2F	842	-	.06	-	7.85	-	15.1
2F	Mn:	.04	.05	7.35	7.74	14.3	15.0

Individual Hematology and Coagulation
Females

Animal Number	HCT Day 3	HCT Day 15	MCV Day 3	MCV Day 15	MCH Day 3	MCH Day 15
1F 823	45.4	X	59.4	X	19.3	X
1F 824	42.6	X	58.3	X	19.2	X
1F 825	45.2	X	59.5	X	19.7	X
1F 826	43.7	X	59.0	X	19.7	X
1F 827	43.3	X	57.7	X	19.5	X
1F 828	-	42.9	-	56.2	-	19.0
1F 829	-	44.4	-	55.6	-	18.9
1F 830	-	43.9	-	57.2	-	19.4
1F 831	-	43.9	-	57.0	-	19.4
1F 832	-	43.7	-	56.4	-	19.6
Ç 1F Mn:	44.0	43.8	58.8	56.5	19.5	19.3
2F 833	42.7	X	59.3	X	20.2	X
2F 834	43.3	X	60.4	X	19.3	X
2F 835	43.5	X	57.7	X	19.2	X
2F 836	41.6	X	59.6	X	19.3	X
2F 837	46.0	X	58.5	X	19.3	X
2F 838	-	42.8	-	55.9	-	19.0
2F 839	-	44.5	-	57.7	-	19.8
2F 840	-	44.2	-	56.6	-	19.0
2F 841	-	44.5	-	57.8	-	19.4
2F 842	-	44.3	-	56.5	-	19.2
2F Mn:	43.4	44.1	59.1	56.9	19.5	19.3

Individual Hematology and Coagulation
Females

Animal Number		MCHC Day 3	MCHC Day 15	PLT Day 3	PLT Day 15	Retic Day 3	Retic Day 15
1F	823	32.4	X	1577	X	4.20	X
1F	824	33.0	X	1354	X	3.37	X
1F	825	33.0	X	1266	X	2.92	X
1F	826	33.3	X	1575	X	3.57	X
1F	827	33.8	X	1283A	X	2.66	X
1F	828	-	33.8	-	1136	-	2.56
1F	829	-	33.9	-	981	-	3.13
1F	830	-	33.8	-	1170	-	2.21
1F	831	-	34.1	-	1297A	-	4.39
1F	832	-	34.8	-	1262A	-	2.15
Ç 1F	Mn:	33.1	34.1	1411	1169	3.34	2.89
2F	833	34.1	X	1316	X	4.74	X
2F	834	32.0	X	1292	X	3.67	X
2F	835	33.2	X	1597A	X	3.63	X
2F	836	32.4	X	1567	X	5.10	X
2F	837	33.0	X	1309	X	2.89	X
2F	838	-	34.0	-	1179	-	2.39
2F	839	-	34.4	-	1105	-	2.66
2F	840	-	33.6	-	1352	-	3.25
2F	841	-	33.6	-	1124	-	3.59
2F	842	-	34.0	-	1113	-	3.78
2F	Mn:	32.9	33.9	1416	1175	4.01	3.13

Individual Hematology and Coagulation
Females

	Animal Number		PT Day 3	PT Day 15	APTT Day 3	APTT Day 15	FIB Day 3	FIB Day 15
	1F	823	17.1	X	27.4	X	Q	X
	1F	824	16.6	X	Q	X	Q	X
	1F	825	18.0	X	Q	X	Q	X
	1F	826	15.5	X	Q	X	Q	X
	1F	827	16.8	X	Q	X	Q	X
	1F	828	-	15.2	-	21.6	-	Q
	1F	829	-	16.6	-	18.0	-	Q
	1F	830	-	Q	-	Q	-	Q
	1F	831	-	17.9	-	20.5	-	Q
	1F	832	-	15.3	-	15.0	-	Q
Ç	1F	Mn:	16.8	16.3	27.4	18.8	-	-
	2F	833	Q	X	Q	X	Q	X
	2F	834	15.8	X	24.6	X	Q	X
	2F	835	17.0	X	Q	X	Q	X
	2F	836	16.6	X	17.6	X	Q	X
	2F	837	Q	X	20.2	X	Q	X
	2F	838	-	15.7	-	Q	-	Q
	2F	839	-	14.7	-	15.0	-	Q
	2F	840	-	17.9	-	14.4	-	Q
	2F	841	-	15.6	-	12.8	-	Q
	2F	842	-	14.7	-	14.0	-	Q
	2F	Mn:	16.5	15.7	20.8<	14.1*	-	-

Individual Clinical Chemistry

Key to Individual Clinical Chemistry

Dosage Key

Group Number	Dose Level	Dose Conc.	Dose Volume	Dosing Route	Total # of Animals	Day 3 Cohort	Day 15 Cohort
1	0.00 mg/kg (vehicle)	0.00 mg/ml	5 ml/kg	IV	10M 10F	5M 5F	5M 5F
2	1.10 mg/kg	0.22 mg/ml	5 ml/kg	IV	10M 10F	5M 5F	5M 5F

Abbreviations

NA	Sodium	mEq/L
K	Potassium	mEq/L
CL	Chloride	mEq/L
ALB	Albumin	g/dL
ALP	Alkaline phosphatase	U/L
ALT	Alanine aminotransferase	U/L
AST	Aspartate aminotransferase	U/L
BUN	Blood urea nitrogen	mg/dL
CA	Calcium	mg/dL
CHOL	Total cholesterol	mg/dL
CRE	Creatinine	mg/dL
GGT	Gamma Glutamyltransferase	U/L
GLU	Glucose	mg/dL
PHOS	Inorganic phosphorus	mg/dL
TBIL	Total bilirubin	mg/dL
TP	Total protein	g/dL
TRIG	Triglycerides	mg/dL
GLOB	Globulin (calculated)	g/dL
A/G	Albumin/globulin ratio (calculated)	--

Key to Individual Clinical Chemistry (cont.)

Report Code Key

- \- = Animal not sampled at time point
- X = Animal dead
- 8< = Value less than 8, although reported as 8 for statistical calculations

Dunnett's Test Key

- * = .05 Significance by Dunnett's Test
- + = .01 Significance by Dunnett's Test
- < = Less than 5 degrees of freedom by Dunnett's Test
- Ç = Control group used for Dunnett's Test comparison

Individual Clincial Chemistry
Males

Animal Number		NA Day 3	NA Day 15	K Day 3	K Day 15	CL Day 3	CL Day 15
1M	801	144	X	5.3	X	102	X
1M	802	141	X	4.6	X	103	X
1M	803	143	X	5.2	X	103	X
1M	804	144	X	5.1	X	102	X
1M	805	143	X	5.2	X	101	X
1M	806	-	142	-	5.3	-	103
1M	807	-	142	-	4.8	-	101
1M	808	-	142	-	5.0	-	103
1M	809	-	141	-	5.3	-	102
1M	810	-	143	-	5.1	-	105
Ç 1M	Mn:	143	142	5.1	5.1	102	103
2M	811	143	X	5.0	X	102	X
2M	812	142	X	5.6	X	102	X
2M	813	143	X	5.4	X	103	X
2M	814	140	X	5.4	X	102	X
2M	815	140	X	5.6	X	101	X
2M	816	-	143	-	4.4	-	101
2M	817	-	142	-	5.0	-	103
2M	818	-	142	-	4.8	-	102
2M	819	-	142	-	4.9	-	102
2M	820	-	142	-	5.2	-	103
2M	Mn:	142	142	5.4	4.9	102	102

BASi Study Number: 1327-14292 (SB-SU-003) Appendix No. 2

Individual Clincial Chemistry
Males

Animal Number		ALB Day 3	ALB Day 15	ALP Day 3	ALP Day 15	ALT Day 3	ALT Day 15
1M	801	3.7	X	245	X	63	X
1M	802	3.0	X	233	X	72	X
1M	803	3.6	X	211	X	55	X
1M	804	3.7	X	226	X	63	X
1M	805	3.6	X	262	X	66	X
1M	806	-	3.7	-	201	-	64
1M	807	-	3.9	-	216	-	66
1M	808	-	3.8	-	171	-	66
1M	809	-	3.9	-	167	-	73
1M	810	-	3.8	-	196	-	70
Ç 1M	Mn:	3.5	3.8	235	190	64	68
2M	811	3.5	X	240	X	74	X
2M	812	3.7	X	260	X	75	X
2M	813	3.6	X	245	X	76	X
2M	814	3.6	X	260	X	87	X
2M	815	3.6	X	257	X	73	X
2M	816	-	3.6	-	254	-	91
2M	817	-	3.7	-	288	-	83
2M	818	-	3.8	-	218	-	71
2M	819	-	3.8	-	202	-	70
2M	820	-	4.0	-	273	-	83
2M	Mn:	3.6	3.8	252	247*	77+	80*

2

Individual Clincial Chemistry
Males

Animal Number		AST Day 3	AST Day 15	BUN Day 3	BUN Day 15	CA Day 3	CA Day 15
1M	801	138	X	19	X	9.7	X
1M	802	142	X	22	X	9.5	X
1M	803	125	X	15	X	10.1	X
1M	804	105	X	11	X	10.2	X
1M	805	113	X	12	X	10.2	X
1M	806	-	97	-	15	-	9.8
1M	807	-	106	-	20	-	9.9
1M	808	-	115	-	16	-	9.9
1M	809	-	110	-	17	-	9.9
1M	810	-	108	-	18	-	10.0
Ç 1M	Mn:	125	107	16	17	9.9	9.9
2M	811	102	X	15	X	9.6	X
2M	812	121	X	12	X	10.0	X
2M	813	109	X	17	X	10.3	X
2M	814	129	X	14	X	10.1	X
2M	815	116	X	17	X	10.2	X
2M	816	-	115	-	22	-	10.0
2M	817	-	124	-	19	-	9.7
2M	818	-	111	-	24	-	10.0
2M	819	-	103	-	20	-	9.9
2M	820	-	104	-	15	-	10.0
2M	Mn:	115	111	15	20	10.0	9.9

Individual Clincial Chemistry
Males

Animal Number		CHOL Day 3	CHOL Day 15	CRE Day 3	CRE Day 15	GGT Day 3	GGT Day 15
1M	801	131	X	.32	X	8<	X
1M	802	105	X	.32	X	8<	X
1M	803	145	X	.24	X	8<	X
1M	804	142	X	.25	X	8<	X
1M	805	129	X	.19	X	8<	X
1M	806	-	125	-	.27	-	8<
1M	807	-	118	-	.35	-	8<
1M	808	-	111	-	.32	-	8<
1M	809	-	117	-	.32	-	8<
1M	810	-	101	-	.29	-	8<
Ç 1M	Mn:	130	114	.26	.31	8	8
2M	811	132	X	.23	X	8<	X
2M	812	144	X	.24	X	8<	X
2M	813	158	X	.24	X	8<	X
2M	814	144	X	.19	X	8<	X
2M	815	130	X	.23	X	8<	X
2M	816	-	119	-	.28	-	8<
2M	817	-	128	-	.30	-	8<
2M	818	-	117	-	.31	-	8<
2M	819	-	106	-	.28	-	8<
2M	820	-	118	-	.28	-	8<
2M	Mn:	142	118	.23	.29	8	8

Individual Clincial Chemistry
Males

Animal Number	GLU Day 3	GLU Day 15	PHOS Day 3	PHOS Day 15	TBIL Day 3	TBIL Day 15
1M 801	104	X	9.3	X	.06	X
1M 802	174	X	10.2	X	.07	X
1M 803	77	X	9.0	X	.08	X
1M 804	86	X	8.9	X	.08	X
1M 805	81	X	9.0	X	.07	X
1M 806	-	111	-	9.1	-	.08
1M 807	-	94	-	8.6	-	.06
1M 808	-	93	-	7.9	-	.08
1M 809	-	89	-	8.9	-	.07
1M 810	-	103	-	8.3	-	.07
Ç 1M Mn:	104	98	9.3	8.6	.07	.07
2M 811	107	X	8.5	X	.09	X
2M 812	86	X	9.0	X	.08	X
2M 813	73	X	10.0	X	.15	X
2M 814	110	X	9.6	X	.09	X
2M 815	132	X	9.8	X	.09	X
2M 816	-	88	-	8.8	-	.10
2M 817	-	108	-	8.1	-	.08
2M 818	-	98	-	8.1	-	.08
2M 819	-	99	-	8.3	-	.08
2M 820	-	133	-	8.3	-	.02
2M Mn:	102	105	9.4	8.3	.10	.07

Individual Clincial Chemistry
Males

Animal Number		TP Day 3	TP Day 15	TRIG Day 3	TRIG Day 15	GLOB Day 3	GLOB Day 15
1M	801	6.0	X	92	X	2.3	X
1M	802	4.9	X	60	X	1.9	X
1M	803	5.9	X	60	X	2.3	X
1M	804	6.0	X	75	X	2.3	X
1M	805	5.9	X	92	X	2.3	X
1M	806	-	6.0	-	82	-	2.3
1M	807	-	6.3	-	71	-	2.4
1M	808	-	6.4	-	106	-	2.6
1M	809	-	6.4	-	51	-	2.5
1M	810	-	6.2	-	40	-	2.4
Ç 1M	Mn:	5.7	6.3	76	70	2.2	2.4
2M	811	5.7	X	76	X	2.2	X
2M	812	6.0	X	44	X	2.3	X
2M	813	6.0	X	137	X	2.4	X
2M	814	5.8	X	77	X	2.2	X
2M	815	5.8	X	84	X	2.2	X
2M	816	-	6.0	-	113	-	2.4
2M	817	-	6.0	-	86	-	2.3
2M	818	-	6.3	-	102	-	2.5
2M	819	-	6.2	-	56	-	2.4
2M	820	-	6.4	-	61	-	2.4
2M	Mn:	5.9	6.2	84	84	2.3	2.4

BASi Study Number: 1327-14292 (SB-SU-003) Appendix No. 2

Individual Clincial Chemistry
Males

Animal Number		A/G Day 3	A/G Day 15
1M	801	1.6	X
1M	802	1.6	X
1M	803	1.6	X
1M	804	1.6	X
1M	805	1.6	X
1M	806	-	1.6
1M	807	-	1.6
1M	808	-	1.5
1M	809	-	1.6
1M	810	-	1.6
Ç 1M	Mn:	1.6	1.6
2M	811	1.6	X
2M	812	1.6	X
2M	813	1.5	X
2M	814	1.6	X
2M	815	1.6	X
2M	816	-	1.5
2M	817	-	1.6
2M	818	-	1.5
2M	819	-	1.6
2M	820	-	1.7
2M	Mn:	1.6	1.6

7

Individual Clincial Chemistry
Females

Animal Number		NA Day 3	NA Day 15	K Day 3	K Day 15	CL Day 3	CL Day 15
1F	823	139	X	5.5	X	103	X
1F	824	142	X	4.2	X	104	X
1F	825	142	X	4.4	X	103	X
1F	826	141	X	4.6	X	103	X
1F	827	142	X	4.7	X	106	X
1F	828	-	140	-	4.7	-	103
1F	829	-	141	-	4.6	-	104
1F	830	-	140	-	4.2	-	104
1F	831	-	143	-	4.1	-	104
1F	832	-	141	-	4.7	-	104
Ç 1F	Mn:	141	141	4.7	4.5	104	104
2F	833	143	X	4.4	X	105	X
2F	834	142	X	4.8	X	106	X
2F	835	142	X	4.8	X	104	X
2F	836	141	X	4.9	X	106	X
2F	837	142	X	4.5	X	104	X
2F	838	-	141	-	4.3	-	103
2F	839	-	141	-	4.4	-	104
2F	840	-	142	-	4.7	-	107
2F	841	-	142	-	4.3	-	106
2F	842	-	139	-	4.7	-	101
2F	Mn:	142	141	4.7	4.5	105	104

BASi Study Number: 1327-14292 (SB-SU-003) Appendix No. 2

Individual Clincial Chemistry
Females

Animal Number		ALB Day 3	ALB Day 15	ALP Day 3	ALP Day 15	ALT Day 3	ALT Day 15
1F	823	3.5	X	153	X	45	X
1F	824	4.0	X	144	X	42	X
1F	825	3.8	X	148	X	41	X
1F	826	3.9	X	171	X	52	X
1F	827	4.1	X	116	X	38	X
1F	828	-	3.9	-	122	-	56
1F	829	-	3.8	-	122	-	72
1F	830	-	4.0	-	156	-	53
1F	831	-	3.7	-	93	-	50
1F	832	-	3.9	-	123	-	50
Ç 1F	Mn:	3.9	3.9	146	123	44	56
2F	833	3.6	X	123	X	51	X
2F	834	3.9	X	162	X	60	X
2F	835	3.8	X	133	X	43	X
2F	836	3.9	X	184	X	47	X
2F	837	3.8	X	126	X	53	X
2F	838	-	3.8	-	128	-	63
2F	839	-	4.3	-	143	-	53
2F	840	-	3.8	-	126	-	53
2F	841	-	3.7	-	147	-	49
2F	842	-	3.9	-	161	-	69
2F	Mn:	3.8	3.9	146	141	51	57

BASi Study Number: 1327-14292 (SB-SU-003) Appendix No. 2

Individual Clincial Chemistry
Females

Animal Number		AST Day 3	AST Day 15	BUN Day 3	BUN Day 15	CA Day 3	CA Day 15
1F	823	123	X	19	X	9.6	X
1F	824	97	X	15	X	9.9	X
1F	825	102	X	16	X	9.7	X
1F	826	99	X	20	X	10.2	X
1F	827	95	X	29	X	9.9	X
1F	828	-	127	-	21	-	9.8
1F	829	-	118	-	21	-	9.7
1F	830	-	102	-	22	-	9.8
1F	831	-	97	-	18	-	9.6
1F	832	-	115	-	20	-	9.7
Ç 1F	Mn:	103	112	20	20	9.9	9.7
2F	833	125	X	20	X	9.5	X
2F	834	108	X	17	X	9.8	X
2F	835	109	X	19	X	9.7	X
2F	836	107	X	19	X	9.8	X
2F	837	102	X	23	X	9.7	X
2F	838	-	111	-	20	-	9.9
2F	839	-	110	-	21	-	10.1
2F	840	-	109	-	22	-	9.6
2F	841	-	100	-	21	-	9.7
2F	842	-	119	-	21	-	10.0
2F	Mn:	110	110	20	21	9.7	9.9

10

Individual Clincial Chemistry
Females

Animal Number	CHOL Day 3	CHOL Day 15	CRE Day 3	CRE Day 15	GGT Day 3	GGT Day 15
1F 823	117	X	.26	X	8<	X
1F 824	156	X	.25	X	8<	X
1F 825	113	X	.21	X	8<	X
1F 826	122	X	.24	X	8<	X
1F 827	141	X	.42	X	8<	X
1F 828	-	89	-	.36	-	8<
1F 829	-	106	-	.32	-	8<
1F 830	-	124	-	.34	-	8<
1F 831	-	103	-	.30	-	8<
1F 832	-	108	-	.30	-	8<
Ç 1F Mn:	130	106	.28	.32	8	8
2F 833	130	X	.30	X	8<	X
2F 834	136	X	.27	X	8<	X
2F 835	132	X	.24	X	8<	X
2F 836	134	X	.29	X	8<	X
2F 837	126	X	.27	X	8<	X
2F 838	-	113	-	.31	-	8<
2F 839	-	121	-	.35	-	8<
2F 840	-	109	-	.42	-	8<
2F 841	-	109	-	.31	-	8<
2F 842	-	128	-	.32	-	8<
2F Mn:	132	116	.27	.34	8	8

BASi Study Number: 1327-14292 (SB-SU-003) Appendix No. 2

Individual Clincial Chemistry
Females

Animal Number		GLU Day 3	GLU Day 15	PHOS Day 3	PHOS Day 15	TBIL Day 3	TBIL Day 15
1F	823	104	X	7.4	X	.05	X
1F	824	84	X	6.7	X	.05	X
1F	825	80	X	6.6	X	.06	X
1F	826	82	X	8.0	X	.05	X
1F	827	89	X	7.5	X	.06	X
1F	828	-	110	-	7.2	-	.11
1F	829	-	105	-	6.0	-	.09
1F	830	-	98	-	6.5	-	.11
1F	831	-	84	-	5.9	-	.09
1F	832	-	98	-	6.1	-	.09
Ç 1F	Mn:	88	99	7.2	6.3	.05	.10
2F	833	114	X	7.6	X	.05	X
2F	834	128	X	6.9	X	.05	X
2F	835	83	X	6.8	X	.06	X
2F	836	112	X	7.5	X	.05	X
2F	837	94	X	7.4	X	.05	X
2F	838	-	98	-	7.1	-	.09
2F	839	-	91	-	5.9	-	.10
2F	840	-	122	-	5.9	-	.09
2F	841	-	93	-	6.3	-	.06
2F	842	-	109	-	7.4	-	.08
2F	Mn:	106	103	7.2	6.5	.05	.08

12

BASi Study Number: 1327-14292 (SB-SU-003) Appendix No. 2

Individual Clincial Chemistry
Females

Animal Number		TP Day 3	TP Day 15	TRIG Day 3	TRIG Day 15	GLOB Day 3	GLOB Day 15
1F	823	5.6	X	80	X	2.1	X
1F	824	6.4	X	42	X	2.4	X
1F	825	6.1	X	59	X	2.3	X
1F	826	6.2	X	47	X	2.3	X
1F	827	6.5	X	45	X	2.4	X
1F	828	-	6.3	-	32	-	2.4
1F	829	-	6.1	-	59	-	2.3
1F	830	-	6.4	-	37	-	2.4
1F	831	-	5.9	-	56	-	2.2
1F	832	-	6.2	-	37	-	2.3
Ç 1F	Mn:	6.2	6.2	55	44	2.3	2.3
2F	833	5.8	X	53	X	2.2	X
2F	834	6.1	X	32	X	2.2	X
2F	835	6.1	X	47	X	2.3	X
2F	836	6.2	X	26	X	2.3	X
2F	837	6.1	X	45	X	2.3	X
2F	838	-	6.5	-	41	-	2.7
2F	839	-	6.7	-	81	-	2.4
2F	840	-	6.1	-	40	-	2.3
2F	841	-	6.0	-	76	-	2.3
2F	842	-	6.3	-	83	-	2.4
2F	Mn:	6.1	6.3	41	64	2.3	2.4

13

Individual Clincial Chemistry
Females

Animal Number		A/G Day 3	A/G Day 15
1F	823	1.7	X
1F	824	1.7	X
1F	825	1.7	X
1F	826	1.7	X
1F	827	1.7	X
1F	828	-	1.6
1F	829	-	1.7
1F	830	-	1.7
1F	831	-	1.7
1F	832	-	1.7
Ç 1F	Mn:	1.7	1.7
2F	833	1.6	X
2F	834	1.8	X
2F	835	1.7	X
2F	836	1.7	X
2F	837	1.7	X
2F	838	-	1.4
2F	839	-	1.8
2F	840	-	1.7
2F	841	-	1.6
2F	842	-	1.6
2F	Mn:	1.7	1.6

Report Amendment

REPORT AMENDMENT 1

1 IDENTIFYING INFORMATION FOR AMENDMENT

Amendment Number: 1
Amendment Date: 04 February 2015
Principal Investigator: Thea Riggs

2 IDENTIFYING INFORMATION FOR ORIGINAL DOCUMENT

Document Number: 1327-14292
Document Type: GLP Report
Document Date: 24 November 2014
Original Protocol Title: Clinical Pathology Evaluation for 14-Day Single Intravenous Dose Toxicity Study of [18F]FP-R01-MG-F2 in Sprague Dawley Rats
Amended Protocol Title: Clinical Pathology Evaluation for 14-Day Single Intravenous Dose Toxicity Study of [^{19}F]FP-$R_0$1-MG-F2 in Sprague Dawley Rats

These changes are for a correction of the test article name following finalization of the original report. The content of the amended report was reviewed by the BASi Quality Assurance Manager and Management and found to accurately reflect the original report.

3 CHANGES TO REPORT

Report Section: Title Page

Revision: Change "Final Report" to "Amended Final Report."

Revision: Add "Report Amendment #1 Completion: 04 February 2015" as last line of title page.

Revision: Change title of report to "Clinical Pathology Evaluation for 14-Day Single Intravenous Dose Toxicity Study of [^{19}F]FP-$R_0$1-MG-F2 in Sprague Dawley Rats."

Reason for Change: Report amended to change test article name.

Report Section: Table of Contents and Appendix 3

Revision: Add Appendix 3 Report Amendment to report and to Table of Contents.

Reason for Change: Inclusion of report amendment

Report Section: Quality Assurance Statement

Revision: Change title of study to "Clinical Pathology Evaluation for 14-Day Single Intravenous Dose Toxicity Study of [^{19}F]FP-$R_0$1-MG-F2 in Sprague Dawley Rats."

Reason for Change: Report amended to change test article name.

Report Section: Report Title, Page 1

Revision: Replace [18F]FP-R01-MG-F2 with [^{19}F]FP-$R_0$1-MG-F2 in the report title.

Reason for Change: Report amended to change test article name.

Report Section: 4.0 Study Design

Revision: Replace [18F]FP-R01-MG-F2 with [^{19}F]FP-$R_0$1-MG-F2 in the first and second sentences of the section.

Reason for Change: Report amended to change test article name.

4 INSTRUCTIONS

Please replace cover page, Table of Contents, and amended pages with the pages included in this amendment. Please add this amendment and its cover page to be Appendix 3. Original pages should be placed after this page in Appendix 3.

5 QUALITY ASSURANCE STATEMENT

Study Title	Clinical Pathology Evaluation for 14-Day Single Intravenous Dose Toxicity Study of [^{19}F]FP-$R_0$1-MG-F2 in Sprague Dawley Rats
BASi Study No.	1327-14292

In accordance with BASi policy and quality assurance procedures for Good Laboratory Practice (GLP), this final report amendment has been audited and the conduct of this study has been inspected as follows.

Date of Inspection	Inspection	Date Reported to Study Director & Management
04 February 2015	Report Amendment (1) Review	04 February 2015

Quality Assurance Unit
Sandra J. Fox, BS, RQAP-GLP
Quality Assurance Manager
BASi

Signature: Sandra J Fox   Date: 04Feb15

6 APPROVALS

Signature: Tina Riggs   Date: 04Feb15
T.A. Riggs AS, MLT(ASCP)
Clinical Pathology Coordinator
BASi

Signature: L D Hopper   Date: 4 Feb 2015
L.D. Hopper, DVM, PhD, DABT, RQAP-GLP
Director of Toxicology
BASi

FINAL REPORT

CLINICAL PATHOLOGY EVALUATION FOR 14-DAY SINGLE INTRAVENOUS DOSE TOXICITY STUDY OF [18F]FP-R01-MG-F2 IN SPRAGUE DAWLEY RATS

TEST SITE

BASi
10424 Middle Mt. Vernon Road
Mt. Vernon, IN 47620

BASI PROJECT NUMBER

1327-14292

SOBRAN STUDY NUMBER

SB-SU-003

SPONSOR

Stanford University School of Medicine
1201 Welch Road, Room PS049
Stanford, CA 94305-5484

PRINCIPAL INVESTIGATOR

T.A. Riggs AS, MLT (ASCP)

Report Completion: 24 November 2014

BASi Project Number: 1327-14292 (SB-SU-003)

QUALITY ASSURANCE STATEMENT

Study Title Clinical Pathology Evaluation for 14-Day Single Intravenous Dose Toxicity Study of [18F]FP-R01-MG-F2 in Sprague Dawley Rats

BASi Project No. 1327-14292

In accordance with BASi policy and quality assurance procedures for Good Laboratory Practice (GLP), this project has been audited and the conduct of this study has been inspected as follows:

Date of Inspection	Inspection	Date Reported to Study Director & Management
10 October 2014	Sample Receipt; Hematology Evaluation; Wedge Smear Preparation	10 October 2014
18 November 2014	Data; Draft under Circulation for Review and Comment	19 November 2014
24 November 2014	Final Report	24 November 2014

Quality Assurance Unit
Sandra J. Fox, BS, RQAP-GLP
Quality Assurance Manager
BASi

Sandra J Fox 24NOV14
Signature Date

iii

TABLE OF CONTENTS

Signatures.... i
Statement of Regulatory Compliance ii
Quality Assurance Statement iii
Table of Contents iv
1. Test Site.... 1
2. Principal Investigator.... 1
3. Personnel.... 1
4. Study Design.... 1
5. Blood Sample Collection and Storage 2
   5.1. Hematology.... 2
   5.2. Coagulation.... 3
   5.3. Clinical Chemistry 3
6. Data Acquisition and Statistical Analysis 3
   6.1. Data Acquisition 3
   6.2. Statistical Analysis 3
7. Data and Specimen Retention 3
8. References 4

Tables:

1 - Summary of Hematology and Coagulation
2 - Summary of Clinical Chemistry

Appendices:

1 - Individual Hematology and Coagulation
2 - Individual Clinical Chemistry

BASi Project Number: 1327-14292 (SB-SU-003)

Clinical Pathology Evaluation for 14-Day Single Intravenous Dose Toxicity Study of [18F]FP-R01-MG-F2 in Sprague Dawley Rats

1. TEST SITE

BASi
10424 Middle Mt. Vernon Road
Mt. Vernon, IN 47620 USA

2. PRINCIPAL INVESTIGATOR

T.A. Riggs AS, MLT (ASCP)
BASi
Phone: 812.985.5900 ext. 1122
Fax: 812.985.3403
Email: tariggs@basinc.com

3. PERSONNEL

Sr. Director, Preclinical Services
P. A. Downing, BA

Director of Toxicology
L.D. Hopper, DVM, PhD, DABT, RQAP-GLP

Clinical Pathology Coordinator
T.A. Riggs AS, MLT (ASCP)

Quality Assurance Manager
S. J. Fox, BS, RQAP-GLP

4. STUDY DESIGN

The goal of this study was to assess the toxicity of [18F]FP-R01-MG-F2 a small molecule (peptide) that is administered with a specific radioactive probe during Positron Emission Tomography (PET) scans to visualize tumors or targeted cells. For this study the toxicity of [18F]FP-R01-MG-F2 (note - small molecule only, no radioactive component) was assessed following a single intravenous (IV) dose of 1.10 mg/kg (250x the clinical dose) of the test article. The vehicle (10% ethanol in normal saline) was also tested to establish baseline toxicity.

Randomized animals were placed in the following groups:

Group Number	Dose Level	Dose Concentration	Dose Volume	Dosing Route	Total # of Animals	Day 3 Cohort	Day 15 Cohort
1	0.00 mg/kg (Vehicle)	0.00 mg/ml	5 ml/kg	IV	10M 10F	5M 5F	5M 5F
2	1.10 mg/kg	0.22 mg/ml	5 ml/kg	IV	10M 10F	5M 5F	5M 5F

APPENDIX C – PATHOLOGY REPORT

HSRL

Histo-Scientific Research Laboratories

FINAL REPORT AMENDMENT RECORD

**Study Number:** SB-SU-003	**Amendment Number:** 1	**Date:** 20Feb15
**Study Title: 14-Day Single Intravenous Dose Toxicity Study of [^{19}F]FP-R$_0$1-MG-F2 in Sprague Dawley Rats**		

This Amendment records a:

☒ **Modification of Final Report [Addition(s) and/or Deletion(s)]**
☐ **Replacement of Final Report**

Date of Final Report: 5Dec14

Amendment(s):

Subject: Final Pathology Report

Throughout the Final Pathology Report "[18F]FP-R01-MG-F2" will be replaced by "[^{19}F]FP-R$_0$1-MG-F2".

Reason for Amendment: Revision of test article name as per Protocol Amendment 1

Approving Signatures:

20FEB2015
Date

[signature]
Study Pathologist

23Feb15
Date

Victoria L. Cook
Quality Assurance

Form 908-1v2 | Page 1 of 1 | Origination: 22OCT10 Effective: 12NOV10

HSRL HistoScientific Research Laboratories

QUALITY ASSURANCE STATEMENT

Study Title: 14-Day Single Intravenous Dose Toxicity Study of [^{19}F]FP-R$_0$1-MG-F2 in Sprague Dawley Rats

Client Study Number: SB-SU-003

This histopathology project has been inspected and audited by the HSRL Quality Assurance Unit (QAU) as required by the Good Laboratory Practice (GLP) regulations disseminated by the U.S. Food and Drug Administration (FDA, 21 CFR 58).

Area Inspected		Dates: Inspection[1]	Reported[2]	Reported[3]
Critical Phase:	Embedding	05NOV14	11NOV14	11NOV14
Data Review		07,10-11NOV14	17NOV14	17NOV14
Pathology Report:	Draft	19-20NOV14	21NOV14	21NOV14
	Final	04-05DEC14	05DEC14	05DEC14
Amended Final Pathology Report	Amendment 1	19FEB15	20FEB15	20FEB15

All the results/conclusions of the pathology report accurately reflect the raw data.

[1] Date(s) of inspection

[2] Date(s) inspections reported to HSRL Laboratory Director/Test Site Management, Principal Investigator/HSRL Study Pathologist

[3] Date(s) inspections reported to Study Director, Test Facility Management, and Lead QA (if appropriate)

Victoria L. Cook
Victoria L. Cook, RQAP-GLP
Quality Assurance Auditor

20 February 2015
Date

Form 1501-1v3

Origination: 3/13/08
Effective: 3/14/08

HSRL INC. HISTO-SCIENTIFIC RESEARCH LABORATORIES | HISTOLOGY PATHOLOGY ARCHIVES

PATHOLOGY REPORT

14-DAY SINGLE INTRAVENOUS DOSE TOXICITY STUDY OF [18F]FP-R01-MG-F2 IN SPRAGUE DAWLEY RATS

SoBran Study Number SB-SU-003

Prepared by

HSRL
Histo-Scientific Research Laboratories
5930 Main Street
Mount Jackson, VA 22842

Testing Facility

SoBran Rangos Animal Facility
855 N. Wolfe Street, Suite 622
Baltimore, MD 21205

Sponsor

Stanford University School of Medicine
1201 Welch Road, Rm PS049
Stanford, CA 94305-5484

December 5, 2014

5930 Main Street
Mt. Jackson, VA 22842
540.477.4440 phone
540.477.4448 fax
www.hsrl.org

TABLE OF CONTENTS

1.0 Executive Summary 3
2.0 Introduction 4
2.1 Protocol 4
2.2 Objective 4
3.0 Methods 4
3.1 Compliance Statement 4
3.2 Study Design 4
Table 1. Group Assignments 4
3.3 Necropsy 4
3.4 Histological Processing 5
4.0 Results 5
4.1 Animal Mortality 5
4.2 Macroscopic Observations 5
4.3 Microscopic Evaluation 5
5.0 Conclusion 6
Appendix A. Histopathology Incidence Tables 7
Appendix B. Quality Assurance Statement 62

1.0 Executive Summary

The goal of this study was to assess the toxicity of [18F]FP-R01-MG-F2 a small molecule (peptide) that is administered with a specific radioactive probe during Positron Emission Tomography (PET) scans to visualize tumors or targeted cells. For this study the toxicity of [18F]FP-R01-MG-F2 (note- small molecule only, no radioactive component) was assessed following a single intravenous (IV) dose of 1.10 mg/kg (250x the clinical dose) of the test article. The vehicle (10% ethanol in normal saline) was also tested to establish baseline toxicity.

Methods: According to the protocol, 40 rats (20 male and 20 female) were assigned to two treatment groups for this study. Animals were dosed once via intravenous injection. On Day 3, five animals per sex per group were euthanized, while surviving animals remained on study untreated and were terminated on Day 15. All animals at both time points were subjected to a comprehensive necropsy. Protocol specified tissues were collected and forwarded to HSRL where tissues from all animals from both time points were processed, embedded in paraffin, sectioned and stained with hematoxylin and eosin (H&E). The resulting slides were evaluated via light microscopy by David S. Garlick, DVM, DACVP at HSRL.

Conclusion: The goal of this study was to assess the toxicity of [18F]FP-R01-MG-F2 a small molecule (peptide) that is administered with a specific radioactive probe during Positron Emission Tomography (PET) scans to visualize tumors or targeted cells. For this study the toxicity of [18F]FP-R01-MG-F2 (note- small molecule only, no radioactive component) was assessed following a single intravenous (IV) dose of 1.10 mg/kg (250x the clinical dose) of the test article. The vehicle (10% ethanol in normal saline) was also tested to establish baseline toxicity.

Under the conditions of this study, there were no microscopic findings in the tissues evaluated related to the single dose intravenous administration of [18F]FP-R01-MG-F2 at 1.10 mg/kg. Additionally, there were no microscopic findings related to administration of the vehicle, 10% ethanol in normal saline.

2.0 Introduction

2.1 Protocol

This report presents the histopathology results of a toxicity study of [18F]FP-R01-MG-F2 administered via a single intravenous dose to rats, SoBran Study Number SB-SU-003, Stanford University School of Medicine, Stanford, CA. All in-life procedures and tissue harvests were performed at SoBran Rangos Animal Facility under the direction of Adrienne Edgell, BS, CMAR, LATG, Study Director. Histology was performed at HSRL and microscopic evaluation was completed by David S. Garlick, DVM, DACVP at HSRL.

2.2 Objective

The goal of this study was to assess the toxicity of [18F]FP-R01-MG-F2 a small molecule (peptide) that is administered with a specific radioactive probe during Positron Emission Tomography (PET) scans to visualize tumors or targeted cells. For this study the toxicity of [18F]FP-R01-MG-F2 (note- small molecule only, no radioactive component) was assessed following a single intravenous (IV) dose of 1.10 mg/kg (250x the clinical dose) of the test article. The vehicle (10% ethanol in normal saline) was also tested to establish baseline toxicity.

3.0 Methods

3.1 Compliance Statement

The portions of this study conducted at HSRL were conducted in compliance with the Good Laboratory Practice Regulations 21 CFR Part 58. An electronic copy of this report in portable document format (PDF) will be provided to SoBran in addition to the hard copy. The PDF is a representation of the pathology report hard copy. However, only the signed hard copy of the pathology report is considered raw data.

3.2 Study Design

According to the protocol, 40 animals (20 male and 20 female) assigned to two treatment groups were enrolled in this study. The study design is further described in Table 1.

Table 1. Group Assignments

Group Number	Dose Level	Dose Concentration	Dose Volume	Dosing Route	Total Number of Animals	Day 3 Cohort	Day 15 Cohort
1	0.0 mg/kg (Vehicle)	0.0 mg/ml	5 ml/kg	Intravenous	10 male<br>10 female	5 male<br>5 female	5 male<br>5 female
2	1.10 mg/kg	0.22 mg/ml	5 ml/kg	Intravenous	10 male<br>10 female	5 male<br>5 female	5 male<br>5 female

3.3 Necropsy

On Day 3, five animals per sex per group were euthanized, while the surviving animals remained on study and were terminated on Day 15. All animals at both time points were subjected to a comprehensive necropsy. The following organs (when present) were weighed before fixation, with paired organs weighed together: brain, eyes with optic nerves, heart, kidneys, liver, lung, ovaries, spleen, testes, and thyroid with parathyroid. The following tissues from all animals were preserved in 10% neutral buffered formalin (except for the eyes, optic nerves, and testes which were preserved in modified Davidson's fixative):

Brain*
Cecum*
Colon*
Eyes with optic nerves (2)*
Heart*
Ileum*
Injection site*
Kidneys (2)*
Lesions (if present)*
Liver*
Lungs*
Lymph nodes (mesenteric)*
Ovaries (2) (females)*
Remaining carcass
Salivary glands [mandibular (2)]*
Spleen*
Testes (2) (males)*
Thyroid with parathyroid (2)*
Trachea*
Urinary bladder*

* For histopathological evaluation

3.4 Histological Processing

Collected tissues were forwarded to HSRL where protocol required tissues from all animals from both time points were processed, embedded in paraffin, sectioned and stained with hematoxylin and eosin (H&E). Animal information from SoBran-Rangos Individual Animal Necropsy Records was entered into PathData® Systems (PDS) at HSRL. All microscopic slides were evaluated by David S. Garlick, DVM, DACVP and entered directly into PDS at HSRL. Microscopic findings and microscopic grading definitions are presented in the Histopathology Incidence Tables portion of this report, where the following abbreviations apply due to PDS character limitations:

AOFT= Animal Organ Finding Table
INJECTION SITE= Injection site (tail)
I.V.= Intravenous
LYMPH NODE: MESENT.= Lymph node: mesenteric
RAT (Spr. Dawl.)= Rat (Sprague Dawley)
SALIVARY GLANDS= Salivary glands (mandibular)
Stanford University School= Stanford University School of Medicine

4.0 Results

4.1 Animal Mortality

There were no early deaths among the animals submitted to HSRL for histopathological evaluation.

4.2 Macroscopic Observations

There were no macroscopic observations reported by the Testing Facility.

4.3 Microscopic Evaluation

Injection sites: At Day 3, slight perivascular inflammation was noted at the injection sites in a single Group 1 vehicle male (Animal 801) and a single Group 2 test article female (Animal 836). This finding was characterized by the presence of minor macrophage and neutrophil infiltrates around tail veins used for treatment. The low incidence and intensity of this change was consistent with those that can be induced as a result of tissue trauma at the time of intravenous tail vein injections.

(Note: tail vein injection sites from Day 15 were not evaluated due to suboptimal tissue preservation.)

Systemic tissues: Additional tissues evaluated from males and females at Day 3 and Day 15 included brain, cecum, colon, eyes with optic nerves, heart, ileum, kidneys, liver, lungs,

mandibular salivary glands, mesenteric lymph nodes, ovaries (females), spleen, testes (males), thyroid gland with parathyroid gland, trachea, and urinary bladder.

At both Day 3 and Day 15, there were no microscopic findings in these tissues related to either the administration of the test article, [18F]FP-R01-MG-F2, or the vehicle, 10% ethanol in normal saline. At both time points, microscopic observations in systemic tissues were of sporadic intensity and no incidence patterns/trends to suggest a relationship to the administration of test article and they were incidental as can be observed in rats used in this study (McInnes, 2012).

5.0 Conclusion

The goal of this study was to assess the toxicity of [18F]FP-R01-MG-F2 a small molecule (peptide) that is administered with a specific radioactive probe during Positron Emission Tomography (PET) scans to visualize tumors or targeted cells. For this study the toxicity of [18F]FP-R01-MG-F2 (note- small molecule only, no radioactive component) was assessed following a single intravenous (IV) dose of 1.10 mg/kg (250x the clinical dose) of the test article. The vehicle (10% ethanol in normal saline) was also tested to establish baseline toxicity.

Under the conditions of this study, there were no microscopic findings in the tissues evaluated related to the single dose intravenous administration of [18F]FP-R01-MG-F2 at 1.10 mg/kg. Additionally, there were no microscopic findings related to administration of the vehicle, 10% ethanol in normal saline.

Signature:

David S. Garlick, DVM, DACVP
Study Pathologist

05DEC2014
Date

Reference

McInnes EF. *Background Lesions in Laboratory Animals, A Color Atlas*. Edinburgh: Saunders Elsevier, 2012.

Appendix A. Histopathology Incidence Tables

PATHOLOGY REPORT

PROJECT :SB-SU-003

TEST ITEM	: [18F]FP-R01-MG-F2	PATHOL. NO.: 14041 DSG
TEST SYSTEM	: RAT(SPR.DAWL.), 1 Day, I.V.	DATE : 05-DEC-14
SPONSOR	: Stanford University School	PATHDATA SYSTEM V6.2c2

AUTHENTICATION

I, the undersigned, hereby declare that the histopathology data in this report were compiled by me, and that they reflect accurately the primary data records.

05DEC2014

David Garlick
Pathologist

HSRL
5930 Main Street
Mount Jackson, VA 22842

8

PATHOLOGY REPORT

PROJECT :SB-SU-003

TEST ITEM : [18F]FP-R01-MG-F2
TEST SYSTEM : RAT(SPR.DAWL.), 1 Day, I.V.
SPONSOR : Stanford University School

PATHOL. NO.: 14041 DSG
DATE : 05-DEC-14
PATHDATA SYSTEM V6.2c2

EXPLANATION OF CODES AND SYMBOLS

CODES AND SYMBOLS USED AT ANIMAL LEVEL:

M = Male animal
F = Female animal
K0 = Terminal sacrifice group
R1...R9 = Recovery / post-treatment group 1...9

CODES AND SYMBOLS USED AT ORGAN LEVEL:

A = Severe autolysis, evaluation not possible
* = Comment in text of individual animal data
0 = Tissue not present for histologic examination
' = Histologic examination not required
+ = Organ examined, findings present
- = Organ examined, no pathologic findings noted (AOFT only)
(= Only one of paired organs examined/present

CODES AND SYMBOLS USED AT FINDING LEVEL:

GRADE 1 = Minimal / very few / very small
GRADE 2 = Slight / few / small
P = Finding present, severity not scored
(= Finding unilateral in paired organs

EXPLANATION OF TABLE TEXT(S) USED AT FINDING LEVEL:

HEART
- Mononuc. cell infil.
  = Mononuclear cell infitrates
INJECTION SITE (TAIL)
- Inflammation
  = Inflammation:perivascular
LIVER
- Mononuc. cell infil.
  = Mononuclear cell infitrates
SPLEEN
- Lymphoid hyperplasia
  = Lymphoid hyperplasia follicular

9

PATHOLOGY REPORT
SUMMARY TABLES — PROJECT :SB-SU-003

TEST ITEM : [18F]FP-R01-MG-F2 — PATHOL. NO.: 14041 DSG
TEST SYSTEM : RAT(SPR.DAWL.), 1 Day, I.V. — DATE : 05-DEC-14
SPONSOR : Stanford University School — PATHDATA SYSTEM V6.2c2

NUMBER OF ANIMALS WITH MICROSCOPIC FINDINGS BY ORGAN/GROUP/SEX
STATUS AT NECROPSY: K0

SEX :	MALE	
DOSE GROUP:	1	2
NO.ANIMALS:	5	5
INJECTION SITE :	5	5
- Inflammation :	1	-
Grade 2:	1	-

10

PATHOLOGY REPORT
SUMMARY TABLES
PROJECT :SB-SU-003

TEST ITEM : [18F]FP-R01-MG-F2
TEST SYSTEM : RAT(SPR.DAWL.), 1 Day, I.V.
SPONSOR : Stanford University School

PATHOL. NO.: 14041 DSG
DATE : 05-DEC-14
PATHDATA SYSTEM V6.2c2

NUMBER OF ANIMALS WITH MICROSCOPIC FINDINGS BY ORGAN/GROUP/SEX
STATUS AT NECROPSY: K0

SEX :	FEMALE	
DOSE GROUP:	1	2
NO.ANIMALS:	5	5
INJECTION SITE :	5	5
- Inflammation :	-	1
Grade 2:	-	1
LIVER :	5	5
- Mononuc. cell infil.:	1	-
Grade 1:	1	-
LUNG :	5	5
- Foreign body embolus:	1	-
Grade 1:	1	-
THYROID GLAND :	5	5
- Ectopic thymus :	1	-

11

PATHOLOGY REPORT
SUMMARY TABLES　　　PROJECT :SB-SU-003

TEST ITEM : [18F]FP-R01-MG-F2　　PATHOL. NO.: 14041 DSG
TEST SYSTEM : RAT(SPR.DAWL.), 1 Day, I.V.　　DATE : 05-DEC-14
SPONSOR : Stanford University School　　PATHDATA SYSTEM V6.2c2

NUMBER OF ANIMALS WITH MICROSCOPIC FINDINGS BY ORGAN/GROUP/SEX
STATUS AT NECROPSY: R1

SEX :	MALE	
DOSE GROUP:	1	2
NO.ANIMALS:	5	5
EYES :	5	5
- Degeneration:cornea :	-	1
Grade 2:	-	1
HEART :	5	5
- Mononuc. cell infil.:	1	-
Grade 1:	1	-
LIVER :	5	5
- Mononuc. cell infil.:	1	-
Grade 1:	1	-
THYROID GLAND :	5	5
- Ectopic thymus :	-	2

12

PATHOLOGY REPORT
SUMMARY TABLES

PROJECT :SB-SU-003

TEST ITEM : [18F]FP-R01-MG-F2
TEST SYSTEM : RAT(SPR.DAWL.), 1 Day, I.V.
SPONSOR : Stanford University School

PATHOL. NO.: 14041 DSG
DATE : 05-DEC-14
PATHDATA SYSTEM V6.2c2

NUMBER OF ANIMALS WITH MICROSCOPIC FINDINGS BY ORGAN/GROUP/SEX
STATUS AT NECROPSY: R1

SEX :	FEMALE	
DOSE GROUP:	1	2
NO.ANIMALS:	5	5
LIVER :	5	5
- Mononuc. cell infil.:	1	-
Grade 1:	1	-
SPLEEN :	5	5
- Lymphoid hyperplasia:	1	-
Grade 1:	1	-
THYROID GLAND :	5	5
- Ectopic thymus :	1	-

13

PATHOLOGY REPORT
INDIVIDUAL ANIMAL DATA PROJECT :SB-SU-003

TEST ITEM : [18F]FP-R01-MG-F2 PATHOL. NO.: 14041 DSG
TEST SYSTEM : RAT(SPR.DAWL.), 1 Day, I.V. DATE : 05-DEC-14
SPONSOR : Stanford University School PATHDATA SYSTEM V6.2c2

TABLE OF INDIVIDUAL MICROSCOPIC FINDINGS (AOFT)
DOSE GROUP : 1, Group 1

ANIMAL NUMBER :	801 MK0	802 MK0	803 MK0	804 MK0	805 MK0	806 MR1	807 MR1	808 MR1	809 MR1	810 MR1
BRAIN	-	-	-	-	-	-	-	-	-	-
CECUM	-	-	-	-	-	-	-	-	-	-
COLON	-	-	-	-	-	-	-	-	-	-
EYES	-	(-*	-	-	-	-	-	-	-	-
HEART	-	-	-	-	-	-	+	-	-	-
- Mononuc. cell infil.	.	.	.	.	.	.	1.	.	.	.
ILEUM	-	-	-	-	-	-	-	-	-	-
INJECTION SITE	+	-	-	-	-	A	A	A	A	A
- Inflammation	2.	.	.	.	.	.	.	.	.	.
KIDNEYS	-	-	-	-	-	-	-	-	-	-
LIVER	-	-	-	-	-	-	+	-	-	-
- Mononuc. cell infil.	.	.	.	.	.	.	1.	.	.	.
LUNG	-	-	-	-	-	-	-	-	-	-
LYMPH NODE: MESENT.	-	-	-	-	-	-	-	-	-	-
OPTIC NERVES	-	-	-	-	-	-	-	-	-	-
PARATHYROID GLANDS	-	(-	0	0	-	0	(-	0	-*	-
SALIVARY GLANDS	-	-	-	-	-	-	-	-	-	-
SPLEEN	-	-	-	-	-	-	-	-	-	-
TESTES	-	-	-	-	-	-	-	-	-	-
THYROID GLAND	-	-	0	-	-	-	-	-	-	-
TRACHEA	-	-	-	-	-	-	-	-	-	-

14

PATHOLOGY REPORT
INDIVIDUAL ANIMAL DATA PROJECT :SB-SU-003

TEST ITEM : [18F]FP-R01-MG-F2 PATHOL. NO.: 14041 DSG
TEST SYSTEM : RAT(SPR.DAWL.), 1 Day, I.V. DATE : 05-DEC-14
SPONSOR : Stanford University School PATHDATA SYSTEM V6.2c2

TABLE OF INDIVIDUAL MICROSCOPIC FINDINGS (AOFT)
DOSE GROUP : 1, Group 1

ANIMAL NUMBER :	801 MK0	802 MK0	803 MK0	804 MK0	805 MK0	806 MR1	807 MR1	808 MR1	809 MR1	810 MR1
URINARY BLADDER	-	-	-	-	-	-	-	-	-	-

....

15

PATHOLOGY REPORT
INDIVIDUAL ANIMAL DATA PROJECT :SB-SU-003

TEST ITEM : [18F]FP-R01-MG-F2 PATHOL. NO.: 14041 DSG
TEST SYSTEM : RAT(SPR.DAWL.), 1 Day, I.V. DATE : 05-DEC-14
SPONSOR : Stanford University School PATHDATA SYSTEM V6.2c2

TABLE OF INDIVIDUAL MICROSCOPIC FINDINGS (AOFT)
DOSE GROUP : 1, Group 1

ANIMAL NUMBER :	823 FK0	824 FK0	825 FK0	826 FK0	827 FK0	828 FR1	829 FR1	830 FR1	831 FR1	832 FR1
BRAIN	-	-	-	-	-	-	-	-	-	-
CECUM	-	-	-	-	-	-	-	-	-	-
COLON	-	-	-	-	-	-	-	-	-	-
EYES	-	-	-	-	-	-	-	-	-	-
HEART	-	-	-	-	-	-	-	-	-	-
ILEUM	-	-	-	-	-	-	-	-	-	-
INJECTION SITE	-	-	-	-	-	A	A	A	A	A
KIDNEYS	-	-	-	-	-	-	-	-	-	-
LIVER	+	-	-	-	-	-	-	-	+	-
- Mononuc. cell infil.	1.	.	.	.	.	.	.	.	1.	.
LUNG	-	+	-	-	-	-	-	-	-	-
- Foreign body embolus	.	1.	.	.	.	.	.	.	.	.
LYMPH NODE: MESENT.	-	-	-	-	-	-	-	-	-	-
OPTIC NERVES	-	-	-	-	-	-	-	-	-	-
OVARIES	-	-	-	-	-	-	-	-	-	-
PARATHYROID GLANDS	-	-	(-	(-	(-	0	0	(-	-	0
SALIVARY GLANDS	-	-	-	-	-	-	-	-	-	-
SPLEEN	-	-	-	-	-	-	-	-	-	+
- Lymphoid hyperplasia	.	.	.	.	.	.	.	.	.	1.
THYROID GLAND	-	-	(-	+	-	-	-	+	-	-
- Ectopic thymus	.	.	.	(P.	.	.	.	(P.	.	.
TRACHEA	-	-	-	-	-	-	-	-	-	-

16

PATHOLOGY REPORT
INDIVIDUAL ANIMAL DATA PROJECT :SB-SU-003

TEST ITEM : [18F]FP-R01-MG-F2 PATHOL. NO.: 14041 DSG
TEST SYSTEM : RAT(SPR.DAWL.), 1 Day, I.V. DATE : 05-DEC-14
SPONSOR : Stanford University School PATHDATA SYSTEM V6.2c2

TABLE OF INDIVIDUAL MICROSCOPIC FINDINGS (AOFT)
DOSE GROUP : 1, Group 1

ANIMAL NUMBER :	823 FK0	824 FK0	825 FK0	826 FK0	827 FK0	828 FR1	829 FR1	830 FR1	831 FR1	832 FR1
URINARY BLADDER	-	-	-	-	-	-	-	-	-	-

17

PATHOLOGY REPORT
INDIVIDUAL ANIMAL DATA — PROJECT :SB-SU-003

TEST ITEM : [18F]FP-R01-MG-F2 — PATHOL. NO.: 14041 DSG
TEST SYSTEM : RAT(SPR.DAWL.), 1 Day, I.V. — DATE : 05-DEC-14
SPONSOR : Stanford University School — PATHDATA SYSTEM V6.2c2

TABLE OF INDIVIDUAL MICROSCOPIC FINDINGS (AOFT)
DOSE GROUP : 2, Group 2

ANIMAL NUMBER :	811 MK0	812 MK0	813 MK0	814 MK0	815 MK0	816 MR1	817 MR1	818 MR1	819 MR1	820 MR1
BRAIN	-	-	-	-	-	-	-	-	-	-
CECUM	-	-	-	-	-	-	-	-	-	-
COLON	-	-	-	-	-	-	-	-	-	-
EYES	-	-	-	-	-	-	-	-	+	-
- Degeneration:cornea	.	.	.	.	.	.	.	.	(2.	.
HEART	-	-	-	-	-	-	-	-	-	-
ILEUM	-	-	-	-	-	-	-	-	-	-
INJECTION SITE	-	-	-	-	-	A	A	A	A	A
KIDNEYS	-	-	-	-	-	-	-	-	-	-
LIVER	-	-	-	-	-	-	-	-	-	-
LUNG	-	-	-	-	-	-	-	-	-	-
LYMPH NODE: MESENT.	-	-	-	-	-	-	-	-	-	-
OPTIC NERVES	-	-	-	-	-	-	-	-	-	-
PARATHYROID GLANDS	-	-	0	(-	-	0	-	0	(-*	(-
SALIVARY GLANDS	-	-	-	-	-	-	-	-	-	-
SPLEEN	-	-	-	-	-	-	-	-	-	-
TESTES	-	-	-	-	-	-	-	-	-	-
THYROID GLAND	-	-	-	-	-	+	-	(-	(-	+
- Ectopic thymus	.	.	.	.	.	(P.	.	.	.	(P.
TRACHEA	-	-	-	-	-	-	-	-	-	-
URINARY BLADDER	-	-	-	-	-	-	-	-	-	-

18

PATHOLOGY REPORT
INDIVIDUAL ANIMAL DATA PROJECT :SB-SU-003

TEST ITEM : [18F]FP-R01-MG-F2 PATHOL. NO.: 14041 DSG
TEST SYSTEM : RAT(SPR.DAWL.), 1 Day, I.V. DATE : 05-DEC-14
SPONSOR : Stanford University School PATHDATA SYSTEM V6.2c2

TABLE OF INDIVIDUAL MICROSCOPIC FINDINGS (AOFT)
DOSE GROUP : 2, Group 2

ANIMAL NUMBER :	833 FK0	834 FK0	835 FK0	836 FK0	837 FK0	838 FR1	839 FR1	840 FR1	841 FR1	842 FR1
BRAIN	-	-	-	-	-	-	-	-	-	-
CECUM	-	-	-	-	-	-	-	-	-	-
COLON	-	-	-	-	-	-	-	-	-	-
EYES	-	-	-	-	-	-	-	-	-	-
HEART	-	-	-	-	-	-	-	-	-	-
ILEUM	-	-	-	-	-	-	-	-	-	-
INJECTION SITE	-	-	-	+	-	A	A	A	A	A
- Inflammation	.	.	.	2.	.	.	.	.	.	.
KIDNEYS	-	-	-	-	-	-	-	-	-	-
LIVER	-	-	-	-	-	-	-	-	-	-
LUNG	-	-	-	-	-	-	-	-	-	-
LYMPH NODE: MESENT.	-	-	-	-	-	-	-	-	-	-
OPTIC NERVES	-	-	-	-	-	-	-	-	-	-
OVARIES	-	-	-	-	-	-	-	-	-	-
PARATHYROID GLANDS	(-	-	0	-	(-	(-	0	(-	0	0
SALIVARY GLANDS	-	-	-	-	-	-	-	-	-	-
SPLEEN	-	-	-	-	-	-	-	-	-	-
THYROID GLAND	-	-	-	-	-	-	-	-	-	(-
TRACHEA	-	-	-	-	-	-	-	-	-	-
URINARY BLADDER	-	-	-	-	-	-	-	-	-	-

PATHOLOGY REPORT
INDIVIDUAL ANIMAL DATA — PROJECT :SB-SU-003

TEST ITEM : [18F]FP-R01-MG-F2 — PATHOL. NO.: 14041 DSG
TEST SYSTEM : RAT(SPR.DAWL.), 1 Day, I.V. — DATE : 05-DEC-14
SPONSOR : Stanford University School — PATHDATA SYSTEM V6.2c2

ANIMAL HEADING DATA
DOSE GROUP : 1, Group 1

ANIMAL NUMBER	SEX M/F	DEFINED AND FINAL STATE OF NECROPSY		TEST DAYS	FIRST AND LAST DAY UNDER TEST		DATE OF NECROPSY
801	M	K0	K0	3	07-OCT-14	09-OCT-14	09-OCT-14
802	M	K0	K0	3	07-OCT-14	09-OCT-14	09-OCT-14
803	M	K0	K0	3	07-OCT-14	09-OCT-14	09-OCT-14
804	M	K0	K0	3	07-OCT-14	09-OCT-14	09-OCT-14
805	M	K0	K0	3	07-OCT-14	09-OCT-14	09-OCT-14
806	M	R1	R1	15	07-OCT-14	21-OCT-14	21-OCT-14
807	M	R1	R1	15	07-OCT-14	21-OCT-14	21-OCT-14
808	M	R1	R1	15	07-OCT-14	21-OCT-14	21-OCT-14
809	M	R1	R1	15	07-OCT-14	21-OCT-14	21-OCT-14
810	M	R1	R1	15	07-OCT-14	21-OCT-14	21-OCT-14
823	F	K0	K0	3	07-OCT-14	09-OCT-14	09-OCT-14
824	F	K0	K0	3	07-OCT-14	09-OCT-14	09-OCT-14
825	F	K0	K0	3	07-OCT-14	09-OCT-14	09-OCT-14
826	F	K0	K0	3	07-OCT-14	09-OCT-14	09-OCT-14
827	F	K0	K0	3	07-OCT-14	09-OCT-14	09-OCT-14
828	F	R1	R1	15	07-OCT-14	21-OCT-14	21-OCT-14
829	F	R1	R1	15	07-OCT-14	21-OCT-14	21-OCT-14
830	F	R1	R1	15	07-OCT-14	21-OCT-14	21-OCT-14
831	F	R1	R1	15	07-OCT-14	21-OCT-14	21-OCT-14
832	F	R1	R1	15	07-OCT-14	21-OCT-14	21-OCT-14

20

```
PATHOLOGY REPORT
INDIVIDUAL ANIMAL DATA                                    PROJECT     :SB-SU-003

TEST ITEM      : [18F]FP-R01-MG-F2                        PATHOL. NO.: 14041 DSG
TEST SYSTEM    : RAT(SPR.DAWL.), 1 Day, I.V.              DATE       : 05-DEC-14
SPONSOR        : Stanford University School               PATHDATA SYSTEM V6.2c2

TEXT OF GROSS AND MICROSCOPIC FINDINGS
DOSE GROUP     :  1, Group 1

* ANIMAL NUMBER               :      801            SEX  : MALE
  FIRST DAY ON TEST           : 07-Oct-14
  LAST  DAY ON TEST           : 09-Oct-14
  DAYS ON TEST                :        3
  DATE OF NECROPSY            : 09-Oct-14
  DEFINED SACR.GROUP          : TERMINAL SACRIFICE GROUP
  STATUS AT NECROPSY          : TERMINAL SACRIFICE GROUP
.........................................................................

* NECROPSY FINDINGS

  NO NECROPSY OBSERVATIONS NOTED.

* MICROSCOPIC FINDINGS

  INJECTION SITE (TAIL):
   -Inflammation:perivascular, grade 2
  ALL OTHER PROTOCOL TISSUES WITHOUT PATHOLOGIC FINDINGS.
```

21

```
PATHOLOGY REPORT
INDIVIDUAL ANIMAL DATA                                   PROJECT     :SB-SU-003

TEST ITEM      : [18F]FP-R01-MG-F2                       PATHOL. NO.: 14041 DSG
TEST SYSTEM    : RAT(SPR.DAWL.), 1 Day, I.V.             DATE       : 05-DEC-14
SPONSOR        : Stanford University School              PATHDATA SYSTEM V6.2c2

TEXT OF GROSS AND MICROSCOPIC FINDINGS
DOSE GROUP     :  1, Group 1

* ANIMAL NUMBER                :      802            SEX  : MALE
  FIRST DAY ON TEST            : 07-Oct-14
  LAST  DAY ON TEST            : 09-Oct-14
  DAYS ON TEST                 :        3
  DATE OF NECROPSY             : 09-Oct-14
  DEFINED SACR.GROUP           : TERMINAL SACRIFICE GROUP
  STATUS AT NECROPSY           : TERMINAL SACRIFICE GROUP
.........................................................................

* NECROPSY FINDINGS

  NO NECROPSY OBSERVATIONS NOTED.

* MICROSCOPIC FINDINGS

  EYES:
    Only one of paired organs examined/present
    One eye with artifact precludes evaluation.
  PARATHYROID GLANDS:
    Only one of paired organs examined/present
  NO MICROSCOPIC FINDINGS NOTED.
```

22

```
PATHOLOGY REPORT
INDIVIDUAL ANIMAL DATA                                  PROJECT    :SB-SU-003

TEST ITEM     : [18F]FP-R01-MG-F2                       PATHOL. NO.: 14041 DSG
TEST SYSTEM   : RAT(SPR.DAWL.), 1 Day, I.V.             DATE       : 05-DEC-14
SPONSOR       : Stanford University School              PATHDATA SYSTEM V6.2c2

TEXT OF GROSS AND MICROSCOPIC FINDINGS
DOSE GROUP    :  1, Group 1

* ANIMAL NUMBER               :      803             SEX  : MALE
  FIRST DAY ON TEST           : 07-Oct-14
  LAST  DAY ON TEST           : 09-Oct-14
  DAYS ON TEST                :        3
  DATE OF NECROPSY            : 09-Oct-14
  DEFINED SACR.GROUP          : TERMINAL SACRIFICE GROUP
  STATUS AT NECROPSY          : TERMINAL SACRIFICE GROUP
.........................................................................

* NECROPSY FINDINGS

  NO NECROPSY OBSERVATIONS NOTED.

* MICROSCOPIC FINDINGS

  PARATHYROID GLANDS:
    Tissue not present for histologic examination
  THYROID GLAND:
    Tissue not present for histologic examination
  ALL OTHER PROTOCOL TISSUES WITHOUT PATHOLOGIC FINDINGS.
```

23

```
PATHOLOGY REPORT
INDIVIDUAL ANIMAL DATA                                    PROJECT     :SB-SU-003

TEST ITEM      : [18F]FP-R01-MG-F2                        PATHOL. NO.: 14041 DSG
TEST SYSTEM    : RAT(SPR.DAWL.), 1 Day, I.V.              DATE       : 05-DEC-14
SPONSOR        : Stanford University School               PATHDATA SYSTEM V6.2c2

TEXT OF GROSS AND MICROSCOPIC FINDINGS
DOSE GROUP     :  1, Group 1

* ANIMAL NUMBER               :      804            SEX  : MALE
  FIRST DAY ON TEST           : 07-Oct-14
  LAST  DAY ON TEST           : 09-Oct-14
  DAYS ON TEST                :        3
  DATE OF NECROPSY            : 09-Oct-14
  DEFINED SACR.GROUP          : TERMINAL SACRIFICE GROUP
  STATUS AT NECROPSY          : TERMINAL SACRIFICE GROUP
.........................................................................

* NECROPSY FINDINGS

  NO NECROPSY OBSERVATIONS NOTED.

* MICROSCOPIC FINDINGS

  PARATHYROID GLANDS:
    Tissue not present for histologic examination
  ALL OTHER PROTOCOL TISSUES WITHOUT PATHOLOGIC FINDINGS.
```

24

```
PATHOLOGY REPORT
INDIVIDUAL ANIMAL DATA                                PROJECT    :SB-SU-003
---------------------------------------------------------------------------
TEST ITEM      : [18F]FP-R01-MG-F2                    PATHOL. NO.: 14041 DSG
TEST SYSTEM    : RAT(SPR.DAWL.), 1 Day, I.V.          DATE       : 05-DEC-14
SPONSOR        : Stanford University School           PATHDATA SYSTEM V6.2c2
---------------------------------------------------------------------------
TEXT OF GROSS AND MICROSCOPIC FINDINGS
DOSE GROUP     :  1, Group 1
---------------------------------------------------------------------------

* ANIMAL NUMBER              :      805            SEX  : MALE
  FIRST DAY ON TEST          : 07-Oct-14
  LAST  DAY ON TEST          : 09-Oct-14
  DAYS ON TEST               :         3
  DATE OF NECROPSY           : 09-Oct-14
  DEFINED SACR.GROUP         : TERMINAL SACRIFICE GROUP
  STATUS AT NECROPSY         : TERMINAL SACRIFICE GROUP
. . . . . . . . . . . . . . . . . . . . . . . . . . . . . . . . . . . . . .

* NECROPSY FINDINGS

  NO NECROPSY OBSERVATIONS NOTED.

* MICROSCOPIC FINDINGS

  NO MICROSCOPIC FINDINGS NOTED.
---------------------------------------------------------------------------
```

25

```
PATHOLOGY REPORT
INDIVIDUAL ANIMAL DATA                                    PROJECT     :SB-SU-003

TEST ITEM      : [18F]FP-R01-MG-F2                        PATHOL. NO.: 14041 DSG
TEST SYSTEM    : RAT(SPR.DAWL.), 1 Day, I.V.              DATE        : 05-DEC-14
SPONSOR        : Stanford University School               PATHDATA SYSTEM V6.2c2

TEXT OF GROSS AND MICROSCOPIC FINDINGS
DOSE GROUP     :  1, Group 1

* ANIMAL NUMBER               :      806            SEX  : MALE
  FIRST DAY ON TEST           : 07-Oct-14
  LAST  DAY ON TEST           : 21-Oct-14
  DAYS ON TEST                :        15
  DATE OF NECROPSY            : 21-Oct-14
  DEFINED SACR.GROUP          : RECOVERY / POST-TREATMENT GROUP
  STATUS AT NECROPSY          : RECOVERY / POST-TREATMENT GROUP
.........................................................................

* NECROPSY FINDINGS

  NO NECROPSY OBSERVATIONS NOTED.

* MICROSCOPIC FINDINGS

  INJECTION SITE (TAIL):
    Severe autolysis, evaluation not possible
  PARATHYROID GLANDS:
    Tissue not present for histologic examination
  ALL OTHER PROTOCOL TISSUES WITHOUT PATHOLOGIC FINDINGS.
```

```
PATHOLOGY REPORT
INDIVIDUAL ANIMAL DATA                                   PROJECT     :SB-SU-003

TEST ITEM      : [18F]FP-R01-MG-F2                       PATHOL. NO.: 14041 DSG
TEST SYSTEM    : RAT(SPR.DAWL.), 1 Day, I.V.             DATE       : 05-DEC-14
SPONSOR        : Stanford University School              PATHDATA SYSTEM V6.2c2

TEXT OF GROSS AND MICROSCOPIC FINDINGS
DOSE GROUP     :  1, Group 1

* ANIMAL NUMBER              :      807            SEX  : MALE
  FIRST DAY ON TEST          : 07-Oct-14
  LAST  DAY ON TEST          : 21-Oct-14
  DAYS ON TEST               :        15
  DATE OF NECROPSY           : 21-Oct-14
  DEFINED SACR.GROUP         : RECOVERY / POST-TREATMENT GROUP
  STATUS AT NECROPSY         : RECOVERY / POST-TREATMENT GROUP
.........................................................................

* NECROPSY FINDINGS

  NO NECROPSY OBSERVATIONS NOTED.

* MICROSCOPIC FINDINGS

  HEART:
   -Mononuclear cell infitrates, myocardium, focal, grade 1
  INJECTION SITE (TAIL):
    Severe autolysis, evaluation not possible
  LIVER:
   -Mononuclear cell infitrates, focal, grade 1
  PARATHYROID GLANDS:
    Only one of paired organs examined/present
  ALL OTHER PROTOCOL TISSUES WITHOUT PATHOLOGIC FINDINGS.
```

27

```
PATHOLOGY REPORT
INDIVIDUAL ANIMAL DATA                                   PROJECT     :SB-SU-003

TEST ITEM      : [18F]FP-R01-MG-F2                       PATHOL. NO.: 14041 DSG
TEST SYSTEM    : RAT(SPR.DAWL.), 1 Day, I.V.             DATE       : 05-DEC-14
SPONSOR        : Stanford University School              PATHDATA SYSTEM V6.2c2

TEXT OF GROSS AND MICROSCOPIC FINDINGS
DOSE GROUP     :  1, Group 1

* ANIMAL NUMBER              :      808            SEX  : MALE
  FIRST DAY ON TEST          : 07-Oct-14
  LAST  DAY ON TEST          : 21-Oct-14
  DAYS ON TEST               :       15
  DATE OF NECROPSY           : 21-Oct-14
  DEFINED SACR.GROUP         : RECOVERY / POST-TREATMENT GROUP
  STATUS AT NECROPSY         : RECOVERY / POST-TREATMENT GROUP
.........................................................................

* NECROPSY FINDINGS

  NO NECROPSY OBSERVATIONS NOTED.

* MICROSCOPIC FINDINGS

  INJECTION SITE (TAIL):
    Severe autolysis, evaluation not possible
  PARATHYROID GLANDS:
    Tissue not present for histologic examination
  ALL OTHER PROTOCOL TISSUES WITHOUT PATHOLOGIC FINDINGS.
```

28

```
PATHOLOGY REPORT
INDIVIDUAL ANIMAL DATA                                    PROJECT     :SB-SU-003

TEST ITEM      : [18F]FP-R01-MG-F2                        PATHOL. NO.: 14041 DSG
TEST SYSTEM    : RAT(SPR.DAWL.), 1 Day, I.V.              DATE       : 05-DEC-14
SPONSOR        : Stanford University School               PATHDATA SYSTEM V6.2c2

TEXT OF GROSS AND MICROSCOPIC FINDINGS
DOSE GROUP     :  1, Group 1

* ANIMAL NUMBER              :      809            SEX  : MALE
  FIRST DAY ON TEST          : 07-Oct-14
  LAST  DAY ON TEST          : 21-Oct-14
  DAYS ON TEST               :        15
  DATE OF NECROPSY           : 21-Oct-14
  DEFINED SACR.GROUP         : RECOVERY / POST-TREATMENT GROUP
  STATUS AT NECROPSY         : RECOVERY / POST-TREATMENT GROUP
.........................................................................

* NECROPSY FINDINGS

  NO NECROPSY OBSERVATIONS NOTED.

* MICROSCOPIC FINDINGS

  INJECTION SITE (TAIL):
    Severe autolysis, evaluation not possible
  PARATHYROID GLANDS:
    Bilateral parathyroid glands present for evaluation.
  ALL OTHER PROTOCOL TISSUES WITHOUT PATHOLOGIC FINDINGS.
```

29

```
PATHOLOGY REPORT
INDIVIDUAL ANIMAL DATA                                   PROJECT     :SB-SU-003

TEST ITEM      : [18F]FP-R01-MG-F2                       PATHOL. NO.: 14041 DSG
TEST SYSTEM    : RAT(SPR.DAWL.), 1 Day, I.V.             DATE       : 05-DEC-14
SPONSOR        : Stanford University School              PATHDATA SYSTEM V6.2c2

TEXT OF GROSS AND MICROSCOPIC FINDINGS
DOSE GROUP     :  1, Group 1

* ANIMAL NUMBER                :      810           SEX  : MALE
  FIRST DAY ON TEST            : 07-Oct-14
  LAST  DAY ON TEST            : 21-Oct-14
  DAYS ON TEST                 :        15
  DATE OF NECROPSY             : 21-Oct-14
  DEFINED SACR.GROUP           : RECOVERY / POST-TREATMENT GROUP
  STATUS AT NECROPSY           : RECOVERY / POST-TREATMENT GROUP
.........................................................................

* NECROPSY FINDINGS

  NO NECROPSY OBSERVATIONS NOTED.

* MICROSCOPIC FINDINGS

  INJECTION SITE (TAIL):
    Severe autolysis, evaluation not possible
  ALL OTHER PROTOCOL TISSUES WITHOUT PATHOLOGIC FINDINGS.
```

30

```
PATHOLOGY REPORT
INDIVIDUAL ANIMAL DATA                                    PROJECT     :SB-SU-003

TEST ITEM      : [18F]FP-R01-MG-F2                        PATHOL. NO.: 14041 DSG
TEST SYSTEM    : RAT(SPR.DAWL.), 1 Day, I.V.              DATE       : 05-DEC-14
SPONSOR        : Stanford University School               PATHDATA SYSTEM V6.2c2

TEXT OF GROSS AND MICROSCOPIC FINDINGS
DOSE GROUP     :  1, Group 1

* ANIMAL NUMBER              :      823            SEX  : FEMALE
  FIRST DAY ON TEST          : 07-Oct-14
  LAST  DAY ON TEST          : 09-Oct-14
  DAYS ON TEST               :        3
  DATE OF NECROPSY           : 09-Oct-14
  DEFINED SACR.GROUP         : TERMINAL SACRIFICE GROUP
  STATUS AT NECROPSY         : TERMINAL SACRIFICE GROUP
.........................................................................

* NECROPSY FINDINGS

  NO NECROPSY OBSERVATIONS NOTED.

* MICROSCOPIC FINDINGS

  LIVER:
   -Mononuclear cell infitrates, focal, grade 1
  ALL OTHER PROTOCOL TISSUES WITHOUT PATHOLOGIC FINDINGS.
```

```
PATHOLOGY REPORT
INDIVIDUAL ANIMAL DATA                                  PROJECT     :SB-SU-003

TEST ITEM      : [18F]FP-R01-MG-F2                      PATHOL. NO.: 14041 DSG
TEST SYSTEM    : RAT(SPR.DAWL.), 1 Day, I.V.            DATE        : 05-DEC-14
SPONSOR        : Stanford University School             PATHDATA SYSTEM V6.2c2

TEXT OF GROSS AND MICROSCOPIC FINDINGS
DOSE GROUP     :  1, Group 1

* ANIMAL NUMBER              :      824            SEX  : FEMALE
  FIRST DAY ON TEST          : 07-Oct-14
  LAST  DAY ON TEST          : 09-Oct-14
  DAYS ON TEST               :        3
  DATE OF NECROPSY           : 09-Oct-14
  DEFINED SACR.GROUP         : TERMINAL SACRIFICE GROUP
  STATUS AT NECROPSY         : TERMINAL SACRIFICE GROUP
.........................................................................

* NECROPSY FINDINGS

  NO NECROPSY OBSERVATIONS NOTED.

* MICROSCOPIC FINDINGS

  LUNG:
   -Foreign body embolus, single, grade 1
  ALL OTHER PROTOCOL TISSUES WITHOUT PATHOLOGIC FINDINGS.
```

32

```
PATHOLOGY REPORT
INDIVIDUAL ANIMAL DATA                                PROJECT     :SB-SU-003

TEST ITEM      : [18F]FP-R01-MG-F2                    PATHOL. NO.: 14041 DSG
TEST SYSTEM    : RAT(SPR.DAWL.), 1 Day, I.V.          DATE       : 05-DEC-14
SPONSOR        : Stanford University School           PATHDATA SYSTEM V6.2c2

TEXT OF GROSS AND MICROSCOPIC FINDINGS
DOSE GROUP     :  1, Group 1

* ANIMAL NUMBER                :      825            SEX  : FEMALE
  FIRST DAY ON TEST            : 07-Oct-14
  LAST  DAY ON TEST            : 09-Oct-14
  DAYS ON TEST                 :        3
  DATE OF NECROPSY             : 09-Oct-14
  DEFINED SACR.GROUP           : TERMINAL SACRIFICE GROUP
  STATUS AT NECROPSY           : TERMINAL SACRIFICE GROUP
.........................................................................

* NECROPSY FINDINGS

  NO NECROPSY OBSERVATIONS NOTED.

* MICROSCOPIC FINDINGS

  PARATHYROID GLANDS:
    Only one of paired organs examined/present
  THYROID GLAND:
    Only one of paired organs examined/present
  NO MICROSCOPIC FINDINGS NOTED.
```

33

PATHOLOGY REPORT
INDIVIDUAL ANIMAL DATA　　　　PROJECT :SB-SU-003

TEST ITEM	: [18F]FP-R01-MG-F2	PATHOL. NO.:	14041 DSG
TEST SYSTEM	: RAT(SPR.DAWL.), 1 Day, I.V.	DATE	: 05-DEC-14
SPONSOR	: Stanford University School	PATHDATA SYSTEM V6.2c2	

TEXT OF GROSS AND MICROSCOPIC FINDINGS
DOSE GROUP : 1, Group 1

* ANIMAL NUMBER : 826　　SEX : FEMALE
  FIRST DAY ON TEST : 07-Oct-14
  LAST DAY ON TEST : 09-Oct-14
  DAYS ON TEST : 3
  DATE OF NECROPSY : 09-Oct-14
  DEFINED SACR.GROUP : TERMINAL SACRIFICE GROUP
  STATUS AT NECROPSY : TERMINAL SACRIFICE GROUP

....

* NECROPSY FINDINGS

  NO NECROPSY OBSERVATIONS NOTED.

* MICROSCOPIC FINDINGS

  PARATHYROID GLANDS:
    Only one of paired organs examined/present
  THYROID GLAND:
   -Ectopic thymus, unilateral
  ALL OTHER PROTOCOL TISSUES WITHOUT PATHOLOGIC FINDINGS.

34

```
PATHOLOGY REPORT
INDIVIDUAL ANIMAL DATA                                   PROJECT     :SB-SU-003

TEST ITEM      : [18F]FP-R01-MG-F2                       PATHOL. NO.: 14041 DSG
TEST SYSTEM    : RAT(SPR.DAWL.), 1 Day, I.V.             DATE       : 05-DEC-14
SPONSOR        : Stanford University School              PATHDATA SYSTEM V6.2c2

TEXT OF GROSS AND MICROSCOPIC FINDINGS
DOSE GROUP     :  1, Group 1

* ANIMAL NUMBER              :      827             SEX  : FEMALE
  FIRST DAY ON TEST          : 07-Oct-14
  LAST  DAY ON TEST          : 09-Oct-14
  DAYS ON TEST               :        3
  DATE OF NECROPSY           : 09-Oct-14
  DEFINED SACR.GROUP         : TERMINAL SACRIFICE GROUP
  STATUS AT NECROPSY         : TERMINAL SACRIFICE GROUP
.........................................................................

* NECROPSY FINDINGS

  NO NECROPSY OBSERVATIONS NOTED.

* MICROSCOPIC FINDINGS

  PARATHYROID GLANDS:
    Only one of paired organs examined/present
  NO MICROSCOPIC FINDINGS NOTED.
```

35

```
PATHOLOGY REPORT
INDIVIDUAL ANIMAL DATA                                  PROJECT     :SB-SU-003
-------------------------------------------------------------------------------
TEST ITEM      : [18F]FP-R01-MG-F2                      PATHOL. NO.: 14041 DSG
TEST SYSTEM    : RAT(SPR.DAWL.), 1 Day, I.V.            DATE       : 05-DEC-14
SPONSOR        : Stanford University School             PATHDATA SYSTEM V6.2c2
-------------------------------------------------------------------------------
TEXT OF GROSS AND MICROSCOPIC FINDINGS
DOSE GROUP     :  1, Group 1
-------------------------------------------------------------------------------

* ANIMAL NUMBER                :      828           SEX  : FEMALE
  FIRST DAY ON TEST            : 07-Oct-14
  LAST  DAY ON TEST            : 21-Oct-14
  DAYS ON TEST                 :       15
  DATE OF NECROPSY             : 21-Oct-14
  DEFINED SACR.GROUP           : RECOVERY / POST-TREATMENT GROUP
  STATUS AT NECROPSY           : RECOVERY / POST-TREATMENT GROUP
.........................................................................

* NECROPSY FINDINGS

  NO NECROPSY OBSERVATIONS NOTED.


* MICROSCOPIC FINDINGS

  INJECTION SITE (TAIL):
    Severe autolysis, evaluation not possible
  PARATHYROID GLANDS:
    Tissue not present for histologic examination
  ALL OTHER PROTOCOL TISSUES WITHOUT PATHOLOGIC FINDINGS.

-------------------------------------------------------------------------------
```

36

```
PATHOLOGY REPORT
INDIVIDUAL ANIMAL DATA                                   PROJECT     :SB-SU-003

TEST ITEM      : [18F]FP-R01-MG-F2                       PATHOL. NO.: 14041 DSG
TEST SYSTEM    : RAT(SPR.DAWL.), 1 Day, I.V.             DATE       : 05-DEC-14
SPONSOR        : Stanford University School              PATHDATA SYSTEM V6.2c2

TEXT OF GROSS AND MICROSCOPIC FINDINGS
DOSE GROUP     :  1, Group 1

* ANIMAL NUMBER               :      829             SEX  : FEMALE
  FIRST DAY ON TEST           : 07-Oct-14
  LAST  DAY ON TEST           : 21-Oct-14
  DAYS ON TEST                :        15
  DATE OF NECROPSY            : 21-Oct-14
  DEFINED SACR.GROUP          : RECOVERY / POST-TREATMENT GROUP
  STATUS AT NECROPSY          : RECOVERY / POST-TREATMENT GROUP
.........................................................................

* NECROPSY FINDINGS

  NO NECROPSY OBSERVATIONS NOTED.

* MICROSCOPIC FINDINGS

  INJECTION SITE (TAIL):
    Severe autolysis, evaluation not possible
  PARATHYROID GLANDS:
    Tissue not present for histologic examination
  ALL OTHER PROTOCOL TISSUES WITHOUT PATHOLOGIC FINDINGS.
```

```
PATHOLOGY REPORT
INDIVIDUAL ANIMAL DATA                                   PROJECT     :SB-SU-003
--------------------------------------------------------------------------------
TEST ITEM      : [18F]FP-R01-MG-F2                       PATHOL. NO.: 14041 DSG
TEST SYSTEM    : RAT(SPR.DAWL.), 1 Day, I.V.             DATE       : 05-DEC-14
SPONSOR        : Stanford University School              PATHDATA SYSTEM V6.2c2
--------------------------------------------------------------------------------
TEXT OF GROSS AND MICROSCOPIC FINDINGS
DOSE GROUP     :  1, Group 1
--------------------------------------------------------------------------------

* ANIMAL NUMBER                :     830            SEX  : FEMALE
  FIRST DAY ON TEST            : 07-Oct-14
  LAST  DAY ON TEST            : 21-Oct-14
  DAYS ON TEST                 :       15
  DATE OF NECROPSY             : 21-Oct-14
  DEFINED SACR.GROUP           : RECOVERY / POST-TREATMENT GROUP
  STATUS AT NECROPSY           : RECOVERY / POST-TREATMENT GROUP
.........................................................................

* NECROPSY FINDINGS

  NO NECROPSY OBSERVATIONS NOTED.


* MICROSCOPIC FINDINGS

  INJECTION SITE (TAIL):
    Severe autolysis, evaluation not possible
  PARATHYROID GLANDS:
    Only one of paired organs examined/present
  THYROID GLAND:
   -Ectopic thymus, unilateral
  ALL OTHER PROTOCOL TISSUES WITHOUT PATHOLOGIC FINDINGS.
--------------------------------------------------------------------------------
```

38

```
PATHOLOGY REPORT
INDIVIDUAL ANIMAL DATA                                    PROJECT    :SB-SU-003
-------------------------------------------------------------------------------
TEST ITEM     : [18F]FP-R01-MG-F2                         PATHOL. NO.: 14041 DSG
TEST SYSTEM   : RAT(SPR.DAWL.), 1 Day, I.V.               DATE       : 05-DEC-14
SPONSOR       : Stanford University School                PATHDATA SYSTEM V6.2c2
-------------------------------------------------------------------------------
TEXT OF GROSS AND MICROSCOPIC FINDINGS
DOSE GROUP    :  1, Group 1
-------------------------------------------------------------------------------

* ANIMAL NUMBER               :      831            SEX  : FEMALE
  FIRST DAY ON TEST           : 07-Oct-14
  LAST  DAY ON TEST           : 21-Oct-14
  DAYS ON TEST                :        15
  DATE OF NECROPSY            : 21-Oct-14
  DEFINED SACR.GROUP          : RECOVERY / POST-TREATMENT GROUP
  STATUS AT NECROPSY          : RECOVERY / POST-TREATMENT GROUP
.........................................................................

* NECROPSY FINDINGS

  NO NECROPSY OBSERVATIONS NOTED.

* MICROSCOPIC FINDINGS

  INJECTION SITE (TAIL):
    Severe autolysis, evaluation not possible
  LIVER:
   -Mononuclear cell infitrates, multifocal, grade 1
  ALL OTHER PROTOCOL TISSUES WITHOUT PATHOLOGIC FINDINGS.
-------------------------------------------------------------------------------
```

39

```
PATHOLOGY REPORT
INDIVIDUAL ANIMAL DATA                                    PROJECT     :SB-SU-003
--------------------------------------------------------------------------------
TEST ITEM       : [18F]FP-R01-MG-F2                       PATHOL. NO.: 14041 DSG
TEST SYSTEM     : RAT(SPR.DAWL.), 1 Day, I.V.             DATE        : 05-DEC-14
SPONSOR         : Stanford University School              PATHDATA SYSTEM V6.2c2
--------------------------------------------------------------------------------
TEXT OF GROSS AND MICROSCOPIC FINDINGS
DOSE GROUP     :  1, Group 1
--------------------------------------------------------------------------------

* ANIMAL NUMBER                :      832            SEX  : FEMALE
  FIRST DAY ON TEST            : 07-Oct-14
  LAST  DAY ON TEST            : 21-Oct-14
  DAYS ON TEST                 :        15
  DATE OF NECROPSY             : 21-Oct-14
  DEFINED SACR.GROUP           : RECOVERY / POST-TREATMENT GROUP
  STATUS AT NECROPSY           : RECOVERY / POST-TREATMENT GROUP
.........................................................................

* NECROPSY FINDINGS

  NO NECROPSY OBSERVATIONS NOTED.

* MICROSCOPIC FINDINGS

  INJECTION SITE (TAIL):
    Severe autolysis, evaluation not possible
  PARATHYROID GLANDS:
    Tissue not present for histologic examination
  SPLEEN:
   -Lymphoid hyperplasia follicular, grade 1
  ALL OTHER PROTOCOL TISSUES WITHOUT PATHOLOGIC FINDINGS.
--------------------------------------------------------------------------------
```

40

PATHOLOGY REPORT
INDIVIDUAL ANIMAL DATA   PROJECT :SB-SU-003

TEST ITEM : [18F]FP-R01-MG-F2   PATHOL. NO.: 14041 DSG
TEST SYSTEM : RAT(SPR.DAWL.), 1 Day, I.V.   DATE : 05-DEC-14
SPONSOR : Stanford University School   PATHDATA SYSTEM V6.2c2

ANIMAL HEADING DATA
DOSE GROUP : 2, Group 2

ANIMAL NUMBER	SEX M/F	DEFINED AND FINAL STATE OF NECROPSY		TEST DAYS	FIRST AND LAST DAY UNDER TEST		DATE OF NECROPSY
811	M	K0	K0	3	07-OCT-14	09-OCT-14	09-OCT-14
812	M	K0	K0	3	07-OCT-14	09-OCT-14	09-OCT-14
813	M	K0	K0	3	07-OCT-14	09-OCT-14	09-OCT-14
814	M	K0	K0	3	07-OCT-14	09-OCT-14	09-OCT-14
815	M	K0	K0	3	07-OCT-14	09-OCT-14	09-OCT-14
816	M	R1	R1	15	07-OCT-14	21-OCT-14	21-OCT-14
817	M	R1	R1	15	07-OCT-14	21-OCT-14	21-OCT-14
818	M	R1	R1	15	07-OCT-14	21-OCT-14	21-OCT-14
819	M	R1	R1	15	07-OCT-14	21-OCT-14	21-OCT-14
820	M	R1	R1	15	07-OCT-14	21-OCT-14	21-OCT-14
833	F	K0	K0	3	07-OCT-14	09-OCT-14	09-OCT-14
834	F	K0	K0	3	07-OCT-14	09-OCT-14	09-OCT-14
835	F	K0	K0	3	07-OCT-14	09-OCT-14	09-OCT-14
836	F	K0	K0	3	07-OCT-14	09-OCT-14	09-OCT-14
837	F	K0	K0	3	07-OCT-14	09-OCT-14	09-OCT-14
838	F	R1	R1	15	07-OCT-14	21-OCT-14	21-OCT-14
839	F	R1	R1	15	07-OCT-14	21-OCT-14	21-OCT-14
840	F	R1	R1	15	07-OCT-14	21-OCT-14	21-OCT-14
841	F	R1	R1	15	07-OCT-14	21-OCT-14	21-OCT-14
842	F	R1	R1	15	07-OCT-14	21-OCT-14	21-OCT-14

```
PATHOLOGY REPORT
INDIVIDUAL ANIMAL DATA                                  PROJECT     :SB-SU-003

TEST ITEM      : [18F]FP-R01-MG-F2                      PATHOL. NO.: 14041 DSG
TEST SYSTEM    : RAT(SPR.DAWL.), 1 Day, I.V.            DATE       : 05-DEC-14
SPONSOR        : Stanford University School             PATHDATA SYSTEM V6.2c2

TEXT OF GROSS AND MICROSCOPIC FINDINGS
DOSE GROUP     :  2, Group 2

* ANIMAL NUMBER               :      811             SEX  : MALE
  FIRST DAY ON TEST           : 07-Oct-14
  LAST  DAY ON TEST           : 09-Oct-14
  DAYS ON TEST                :         3
  DATE OF NECROPSY            : 09-Oct-14
  DEFINED SACR.GROUP          : TERMINAL SACRIFICE GROUP
  STATUS AT NECROPSY          : TERMINAL SACRIFICE GROUP
.........................................................................

* NECROPSY FINDINGS

  NO NECROPSY OBSERVATIONS NOTED.

* MICROSCOPIC FINDINGS

  NO MICROSCOPIC FINDINGS NOTED.
```

42

```
PATHOLOGY REPORT
INDIVIDUAL ANIMAL DATA                                    PROJECT     :SB-SU-003

TEST ITEM      : [18F]FP-R01-MG-F2                        PATHOL. NO.: 14041 DSG
TEST SYSTEM    : RAT(SPR.DAWL.), 1 Day, I.V.              DATE       : 05-DEC-14
SPONSOR        : Stanford University School               PATHDATA SYSTEM V6.2c2

TEXT OF GROSS AND MICROSCOPIC FINDINGS
DOSE GROUP     :  2, Group 2

* ANIMAL NUMBER               :      812             SEX  : MALE
  FIRST DAY ON TEST           : 07-Oct-14
  LAST  DAY ON TEST           : 09-Oct-14
  DAYS ON TEST                :         3
  DATE OF NECROPSY            : 09-Oct-14
  DEFINED SACR.GROUP          : TERMINAL SACRIFICE GROUP
  STATUS AT NECROPSY          : TERMINAL SACRIFICE GROUP
.........................................................................

* NECROPSY FINDINGS

  NO NECROPSY OBSERVATIONS NOTED.

* MICROSCOPIC FINDINGS

  NO MICROSCOPIC FINDINGS NOTED.
```

43

```
PATHOLOGY REPORT
INDIVIDUAL ANIMAL DATA                                     PROJECT     :SB-SU-003

TEST ITEM      : [18F]FP-R01-MG-F2                         PATHOL. NO.: 14041 DSG
TEST SYSTEM    : RAT(SPR.DAWL.), 1 Day, I.V.               DATE       : 05-DEC-14
SPONSOR        : Stanford University School                PATHDATA SYSTEM V6.2c2

TEXT OF GROSS AND MICROSCOPIC FINDINGS
DOSE GROUP     :  2, Group 2

* ANIMAL NUMBER                :      813              SEX  : MALE
  FIRST DAY ON TEST            : 07-Oct-14
  LAST  DAY ON TEST            : 09-Oct-14
  DAYS ON TEST                 :        3
  DATE OF NECROPSY             : 09-Oct-14
  DEFINED SACR.GROUP           : TERMINAL SACRIFICE GROUP
  STATUS AT NECROPSY           : TERMINAL SACRIFICE GROUP
.........................................................................

* NECROPSY FINDINGS

  NO NECROPSY OBSERVATIONS NOTED.

* MICROSCOPIC FINDINGS

  PARATHYROID GLANDS:
    Tissue not present for histologic examination
  ALL OTHER PROTOCOL TISSUES WITHOUT PATHOLOGIC FINDINGS.
```

44

```
PATHOLOGY REPORT
INDIVIDUAL ANIMAL DATA                                     PROJECT    :SB-SU-003

TEST ITEM      : [18F]FP-R01-MG-F2                         PATHOL. NO.: 14041 DSG
TEST SYSTEM    : RAT(SPR.DAWL.), 1 Day, I.V.               DATE       : 05-DEC-14
SPONSOR        : Stanford University School                PATHDATA SYSTEM V6.2c2

TEXT OF GROSS AND MICROSCOPIC FINDINGS
DOSE GROUP     :  2, Group 2

* ANIMAL NUMBER              :      814             SEX  : MALE
  FIRST DAY ON TEST          : 07-Oct-14
  LAST  DAY ON TEST          : 09-Oct-14
  DAYS ON TEST               :        3
  DATE OF NECROPSY           : 09-Oct-14
  DEFINED SACR.GROUP         : TERMINAL SACRIFICE GROUP
  STATUS AT NECROPSY         : TERMINAL SACRIFICE GROUP
.........................................................................

* NECROPSY FINDINGS

  NO NECROPSY OBSERVATIONS NOTED.

* MICROSCOPIC FINDINGS

  PARATHYROID GLANDS:
    Only one of paired organs examined/present
  NO MICROSCOPIC FINDINGS NOTED.
```

45

```
PATHOLOGY REPORT
INDIVIDUAL ANIMAL DATA                                     PROJECT     :SB-SU-003

TEST ITEM      : [18F]FP-R01-MG-F2                         PATHOL. NO.: 14041 DSG
TEST SYSTEM    : RAT(SPR.DAWL.), 1 Day, I.V.               DATE       : 05-DEC-14
SPONSOR        : Stanford University School                PATHDATA SYSTEM V6.2c2

TEXT OF GROSS AND MICROSCOPIC FINDINGS
DOSE GROUP     :  2, Group 2

* ANIMAL NUMBER                :      815             SEX  : MALE
  FIRST DAY ON TEST            : 07-Oct-14
  LAST  DAY ON TEST            : 09-Oct-14
  DAYS ON TEST                 :        3
  DATE OF NECROPSY             : 09-Oct-14
  DEFINED SACR.GROUP           : TERMINAL SACRIFICE GROUP
  STATUS AT NECROPSY           : TERMINAL SACRIFICE GROUP
.........................................................................

* NECROPSY FINDINGS

  NO NECROPSY OBSERVATIONS NOTED.

* MICROSCOPIC FINDINGS

  NO MICROSCOPIC FINDINGS NOTED.
```

46

```
PATHOLOGY REPORT
INDIVIDUAL ANIMAL DATA                                   PROJECT     :SB-SU-003
--------------------------------------------------------------------------------
TEST ITEM      : [18F]FP-R01-MG-F2                       PATHOL. NO.: 14041 DSG
TEST SYSTEM    : RAT(SPR.DAWL.), 1 Day, I.V.             DATE       : 05-DEC-14
SPONSOR        : Stanford University School              PATHDATA SYSTEM V6.2c2
--------------------------------------------------------------------------------
TEXT OF GROSS AND MICROSCOPIC FINDINGS
DOSE GROUP     :  2, Group 2
--------------------------------------------------------------------------------

* ANIMAL NUMBER                 :      816              SEX  : MALE
  FIRST DAY ON TEST             : 07-Oct-14
  LAST  DAY ON TEST             : 21-Oct-14
  DAYS ON TEST                  :        15
  DATE OF NECROPSY              : 21-Oct-14
  DEFINED SACR.GROUP            : RECOVERY / POST-TREATMENT GROUP
  STATUS AT NECROPSY            : RECOVERY / POST-TREATMENT GROUP
.........................................................................

* NECROPSY FINDINGS

  NO NECROPSY OBSERVATIONS NOTED.


* MICROSCOPIC FINDINGS

  INJECTION SITE (TAIL):
    Severe autolysis, evaluation not possible
  PARATHYROID GLANDS:
    Tissue not present for histologic examination
  THYROID GLAND:
   -Ectopic thymus, unilateral
  ALL OTHER PROTOCOL TISSUES WITHOUT PATHOLOGIC FINDINGS.
--------------------------------------------------------------------------------
```

47

```
PATHOLOGY REPORT
INDIVIDUAL ANIMAL DATA                                  PROJECT     :SB-SU-003

TEST ITEM      : [18F]FP-R01-MG-F2                      PATHOL. NO.: 14041 DSG
TEST SYSTEM    : RAT(SPR.DAWL.), 1 Day, I.V.            DATE       : 05-DEC-14
SPONSOR        : Stanford University School             PATHDATA SYSTEM V6.2c2

TEXT OF GROSS AND MICROSCOPIC FINDINGS
DOSE GROUP     :  2, Group 2

* ANIMAL NUMBER               :      817            SEX  : MALE
  FIRST DAY ON TEST           : 07-Oct-14
  LAST  DAY ON TEST           : 21-Oct-14
  DAYS ON TEST                :        15
  DATE OF NECROPSY            : 21-Oct-14
  DEFINED SACR.GROUP          : RECOVERY / POST-TREATMENT GROUP
  STATUS AT NECROPSY          : RECOVERY / POST-TREATMENT GROUP
.........................................................................

* NECROPSY FINDINGS

  NO NECROPSY OBSERVATIONS NOTED.

* MICROSCOPIC FINDINGS

  INJECTION SITE (TAIL):
    Severe autolysis, evaluation not possible
  ALL OTHER PROTOCOL TISSUES WITHOUT PATHOLOGIC FINDINGS.
```

```
PATHOLOGY REPORT
INDIVIDUAL ANIMAL DATA                                   PROJECT     :SB-SU-003

TEST ITEM      : [18F]FP-R01-MG-F2                       PATHOL. NO.: 14041 DSG
TEST SYSTEM    : RAT(SPR.DAWL.), 1 Day, I.V.             DATE        : 05-DEC-14
SPONSOR        : Stanford University School              PATHDATA SYSTEM V6.2c2

TEXT OF GROSS AND MICROSCOPIC FINDINGS
DOSE GROUP     :  2, Group 2

* ANIMAL NUMBER               :      818           SEX  : MALE
  FIRST DAY ON TEST           : 07-Oct-14
  LAST  DAY ON TEST           : 21-Oct-14
  DAYS ON TEST                :        15
  DATE OF NECROPSY            : 21-Oct-14
  DEFINED SACR.GROUP          : RECOVERY / POST-TREATMENT GROUP
  STATUS AT NECROPSY          : RECOVERY / POST-TREATMENT GROUP
.........................................................................

* NECROPSY FINDINGS

  NO NECROPSY OBSERVATIONS NOTED.

* MICROSCOPIC FINDINGS

  INJECTION SITE (TAIL):
    Severe autolysis, evaluation not possible
  PARATHYROID GLANDS:
    Tissue not present for histologic examination
  THYROID GLAND:
    Only one of paired organs examined/present
  ALL OTHER PROTOCOL TISSUES WITHOUT PATHOLOGIC FINDINGS.
```

49

```
PATHOLOGY REPORT
INDIVIDUAL ANIMAL DATA                                    PROJECT     :SB-SU-003

TEST ITEM      : [18F]FP-R01-MG-F2                        PATHOL. NO.: 14041 DSG
TEST SYSTEM    : RAT(SPR.DAWL.), 1 Day, I.V.              DATE       : 05-DEC-14
SPONSOR        : Stanford University School               PATHDATA SYSTEM V6.2c2

TEXT OF GROSS AND MICROSCOPIC FINDINGS
DOSE GROUP     :  2, Group 2

* ANIMAL NUMBER                :      819            SEX  : MALE
  FIRST DAY ON TEST            : 07-Oct-14
  LAST  DAY ON TEST            : 21-Oct-14
  DAYS ON TEST                 :        15
  DATE OF NECROPSY             : 21-Oct-14
  DEFINED SACR.GROUP           : RECOVERY / POST-TREATMENT GROUP
  STATUS AT NECROPSY           : RECOVERY / POST-TREATMENT GROUP
.........................................................................

* NECROPSY FINDINGS

  NO NECROPSY OBSERVATIONS NOTED.

* MICROSCOPIC FINDINGS

  EYES:
   -Degeneration:cornea, central, unilateral, grade 2
  INJECTION SITE (TAIL):
    Severe autolysis, evaluation not possible
  PARATHYROID GLANDS:
    Only one of paired organs examined/present
    One parathyroid gland present for evaluation.
  THYROID GLAND:
    Only one of paired organs examined/present
  ALL OTHER PROTOCOL TISSUES WITHOUT PATHOLOGIC FINDINGS.
```

50

```
PATHOLOGY REPORT
INDIVIDUAL ANIMAL DATA                                PROJECT     :SB-SU-003
-----------------------------------------------------------------------------
TEST ITEM      : [18F]FP-R01-MG-F2                    PATHOL. NO.: 14041 DSG
TEST SYSTEM    : RAT(SPR.DAWL.), 1 Day, I.V.          DATE       : 05-DEC-14
SPONSOR        : Stanford University School           PATHDATA SYSTEM V6.2c2
-----------------------------------------------------------------------------
TEXT OF GROSS AND MICROSCOPIC FINDINGS
DOSE GROUP     :  2, Group 2
-----------------------------------------------------------------------------

* ANIMAL NUMBER               :     820          SEX  : MALE
  FIRST DAY ON TEST           : 07-Oct-14
  LAST  DAY ON TEST           : 21-Oct-14
  DAYS ON TEST                :       15
  DATE OF NECROPSY            : 21-Oct-14
  DEFINED SACR.GROUP          : RECOVERY / POST-TREATMENT GROUP
  STATUS AT NECROPSY          : RECOVERY / POST-TREATMENT GROUP
.............................................................................

* NECROPSY FINDINGS

  NO NECROPSY OBSERVATIONS NOTED.


* MICROSCOPIC FINDINGS

  INJECTION SITE (TAIL):
    Severe autolysis, evaluation not possible
  PARATHYROID GLANDS:
    Only one of paired organs examined/present
  THYROID GLAND:
   -Ectopic thymus, unilateral
  ALL OTHER PROTOCOL TISSUES WITHOUT PATHOLOGIC FINDINGS.
-----------------------------------------------------------------------------
```

51

```
PATHOLOGY REPORT
INDIVIDUAL ANIMAL DATA                                PROJECT    :SB-SU-003

TEST ITEM      : [18F]FP-R01-MG-F2                    PATHOL. NO.: 14041 DSG
TEST SYSTEM    : RAT(SPR.DAWL.), 1 Day, I.V.          DATE       : 05-DEC-14
SPONSOR        : Stanford University School           PATHDATA SYSTEM V6.2c2

TEXT OF GROSS AND MICROSCOPIC FINDINGS
DOSE GROUP     :  2, Group 2

* ANIMAL NUMBER               :     833             SEX  : FEMALE
  FIRST DAY ON TEST           : 07-Oct-14
  LAST  DAY ON TEST           : 09-Oct-14
  DAYS ON TEST                :         3
  DATE OF NECROPSY            : 09-Oct-14
  DEFINED SACR.GROUP          : TERMINAL SACRIFICE GROUP
  STATUS AT NECROPSY          : TERMINAL SACRIFICE GROUP
.........................................................................

* NECROPSY FINDINGS

  NO NECROPSY OBSERVATIONS NOTED.

* MICROSCOPIC FINDINGS

  PARATHYROID GLANDS:
    Only one of paired organs examined/present
  NO MICROSCOPIC FINDINGS NOTED.
```

```
PATHOLOGY REPORT
INDIVIDUAL ANIMAL DATA                                  PROJECT     :SB-SU-003
--------------------------------------------------------------------------------
TEST ITEM       : [18F]FP-R01-MG-F2                     PATHOL. NO.: 14041 DSG
TEST SYSTEM     : RAT(SPR.DAWL.), 1 Day, I.V.           DATE       : 05-DEC-14
SPONSOR         : Stanford University School            PATHDATA SYSTEM V6.2c2
--------------------------------------------------------------------------------
TEXT OF GROSS AND MICROSCOPIC FINDINGS
DOSE GROUP     :  2, Group 2
--------------------------------------------------------------------------------

* ANIMAL NUMBER                 :      834             SEX  : FEMALE
  FIRST DAY ON TEST             : 07-Oct-14
  LAST  DAY ON TEST             : 09-Oct-14
  DAYS ON TEST                  :        3
  DATE OF NECROPSY              : 09-Oct-14
  DEFINED SACR.GROUP            : TERMINAL SACRIFICE GROUP
  STATUS AT NECROPSY            : TERMINAL SACRIFICE GROUP
.........................................................................

* NECROPSY FINDINGS

  NO NECROPSY OBSERVATIONS NOTED.

* MICROSCOPIC FINDINGS

  NO MICROSCOPIC FINDINGS NOTED.
--------------------------------------------------------------------------------
```

53

```
PATHOLOGY REPORT
INDIVIDUAL ANIMAL DATA                                   PROJECT     :SB-SU-003

TEST ITEM      : [18F]FP-R01-MG-F2                       PATHOL. NO.: 14041 DSG
TEST SYSTEM    : RAT(SPR.DAWL.), 1 Day, I.V.             DATE       : 05-DEC-14
SPONSOR        : Stanford University School              PATHDATA SYSTEM V6.2c2

TEXT OF GROSS AND MICROSCOPIC FINDINGS
DOSE GROUP     :  2, Group 2

* ANIMAL NUMBER               :      835            SEX  : FEMALE
  FIRST DAY ON TEST           : 07-Oct-14
  LAST  DAY ON TEST           : 09-Oct-14
  DAYS ON TEST                :         3
  DATE OF NECROPSY            : 09-Oct-14
  DEFINED SACR.GROUP          : TERMINAL SACRIFICE GROUP
  STATUS AT NECROPSY          : TERMINAL SACRIFICE GROUP
.........................................................................

* NECROPSY FINDINGS

  NO NECROPSY OBSERVATIONS NOTED.

* MICROSCOPIC FINDINGS

  PARATHYROID GLANDS:
    Tissue not present for histologic examination
  ALL OTHER PROTOCOL TISSUES WITHOUT PATHOLOGIC FINDINGS.
```

54

```
PATHOLOGY REPORT
INDIVIDUAL ANIMAL DATA                                  PROJECT     :SB-SU-003

TEST ITEM      : [18F]FP-R01-MG-F2                      PATHOL. NO.: 14041 DSG
TEST SYSTEM    : RAT(SPR.DAWL.), 1 Day, I.V.            DATE       : 05-DEC-14
SPONSOR        : Stanford University School             PATHDATA SYSTEM V6.2c2

TEXT OF GROSS AND MICROSCOPIC FINDINGS
DOSE GROUP     :  2, Group 2

* ANIMAL NUMBER               :      836              SEX  : FEMALE
  FIRST DAY ON TEST           : 07-Oct-14
  LAST  DAY ON TEST           : 09-Oct-14
  DAYS ON TEST                :        3
  DATE OF NECROPSY            : 09-Oct-14
  DEFINED SACR.GROUP          : TERMINAL SACRIFICE GROUP
  STATUS AT NECROPSY          : TERMINAL SACRIFICE GROUP
.........................................................................

* NECROPSY FINDINGS

  NO NECROPSY OBSERVATIONS NOTED.

* MICROSCOPIC FINDINGS

  INJECTION SITE (TAIL):
   -Inflammation:perivascular, grade 2
  ALL OTHER PROTOCOL TISSUES WITHOUT PATHOLOGIC FINDINGS.
```

55

```
PATHOLOGY REPORT
INDIVIDUAL ANIMAL DATA                                     PROJECT     :SB-SU-003

TEST ITEM       : [18F]FP-R01-MG-F2                        PATHOL. NO.: 14041 DSG
TEST SYSTEM     : RAT(SPR.DAWL.), 1 Day, I.V.              DATE       : 05-DEC-14
SPONSOR         : Stanford University School               PATHDATA SYSTEM V6.2c2

TEXT OF GROSS AND MICROSCOPIC FINDINGS
DOSE GROUP     :  2, Group 2

* ANIMAL NUMBER                :      837              SEX  : FEMALE
  FIRST DAY ON TEST            : 07-Oct-14
  LAST  DAY ON TEST            : 09-Oct-14
  DAYS ON TEST                 :        3
  DATE OF NECROPSY             : 09-Oct-14
  DEFINED SACR.GROUP           : TERMINAL SACRIFICE GROUP
  STATUS AT NECROPSY           : TERMINAL SACRIFICE GROUP
.........................................................................

* NECROPSY FINDINGS

  NO NECROPSY OBSERVATIONS NOTED.

* MICROSCOPIC FINDINGS

  PARATHYROID GLANDS:
    Only one of paired organs examined/present
  NO MICROSCOPIC FINDINGS NOTED.
```

56

```
PATHOLOGY REPORT
INDIVIDUAL ANIMAL DATA                                    PROJECT     :SB-SU-003

TEST ITEM      : [18F]FP-R01-MG-F2                        PATHOL. NO.: 14041 DSG
TEST SYSTEM    : RAT(SPR.DAWL.), 1 Day, I.V.              DATE       : 05-DEC-14
SPONSOR        : Stanford University School               PATHDATA SYSTEM V6.2c2

TEXT OF GROSS AND MICROSCOPIC FINDINGS
DOSE GROUP     :  2, Group 2

* ANIMAL NUMBER              :      838            SEX  : FEMALE
  FIRST DAY ON TEST          : 07-Oct-14
  LAST  DAY ON TEST          : 21-Oct-14
  DAYS ON TEST               :        15
  DATE OF NECROPSY           : 21-Oct-14
  DEFINED SACR.GROUP         : RECOVERY / POST-TREATMENT GROUP
  STATUS AT NECROPSY         : RECOVERY / POST-TREATMENT GROUP
.........................................................................

* NECROPSY FINDINGS

  NO NECROPSY OBSERVATIONS NOTED.

* MICROSCOPIC FINDINGS

  INJECTION SITE (TAIL):
    Severe autolysis, evaluation not possible
  PARATHYROID GLANDS:
    Only one of paired organs examined/present
  ALL OTHER PROTOCOL TISSUES WITHOUT PATHOLOGIC FINDINGS.
```

57

```
PATHOLOGY REPORT
INDIVIDUAL ANIMAL DATA                                    PROJECT     :SB-SU-003

TEST ITEM      : [18F]FP-R01-MG-F2                        PATHOL. NO.: 14041 DSG
TEST SYSTEM    : RAT(SPR.DAWL.), 1 Day, I.V.              DATE       : 05-DEC-14
SPONSOR        : Stanford University School               PATHDATA SYSTEM V6.2c2

TEXT OF GROSS AND MICROSCOPIC FINDINGS
DOSE GROUP     :  2, Group 2

* ANIMAL NUMBER                :      839            SEX  : FEMALE
  FIRST DAY ON TEST            : 07-Oct-14
  LAST  DAY ON TEST            : 21-Oct-14
  DAYS ON TEST                 :        15
  DATE OF NECROPSY             : 21-Oct-14
  DEFINED SACR.GROUP           : RECOVERY / POST-TREATMENT GROUP
  STATUS AT NECROPSY           : RECOVERY / POST-TREATMENT GROUP
.........................................................................

* NECROPSY FINDINGS

  NO NECROPSY OBSERVATIONS NOTED.

* MICROSCOPIC FINDINGS

  INJECTION SITE (TAIL):
    Severe autolysis, evaluation not possible
  PARATHYROID GLANDS:
    Tissue not present for histologic examination
  ALL OTHER PROTOCOL TISSUES WITHOUT PATHOLOGIC FINDINGS.
```

```
PATHOLOGY REPORT
INDIVIDUAL ANIMAL DATA                                 PROJECT     :SB-SU-003

TEST ITEM      : [18F]FP-R01-MG-F2                     PATHOL. NO.: 14041 DSG
TEST SYSTEM    : RAT(SPR.DAWL.), 1 Day, I.V.           DATE        : 05-DEC-14
SPONSOR        : Stanford University School            PATHDATA SYSTEM V6.2c2

TEXT OF GROSS AND MICROSCOPIC FINDINGS
DOSE GROUP     :  2, Group 2

* ANIMAL NUMBER              :      840             SEX  : FEMALE
  FIRST DAY ON TEST          : 07-Oct-14
  LAST  DAY ON TEST          : 21-Oct-14
  DAYS ON TEST               :        15
  DATE OF NECROPSY           : 21-Oct-14
  DEFINED SACR.GROUP         : RECOVERY / POST-TREATMENT GROUP
  STATUS AT NECROPSY         : RECOVERY / POST-TREATMENT GROUP
.........................................................................

* NECROPSY FINDINGS

  NO NECROPSY OBSERVATIONS NOTED.

* MICROSCOPIC FINDINGS

  INJECTION SITE (TAIL):
    Severe autolysis, evaluation not possible
  PARATHYROID GLANDS:
    Only one of paired organs examined/present
  ALL OTHER PROTOCOL TISSUES WITHOUT PATHOLOGIC FINDINGS.
```

59

```
PATHOLOGY REPORT
INDIVIDUAL ANIMAL DATA                                    PROJECT     :SB-SU-003

TEST ITEM      : [18F]FP-R01-MG-F2                        PATHOL. NO.: 14041 DSG
TEST SYSTEM    : RAT(SPR.DAWL.), 1 Day, I.V.              DATE        : 05-DEC-14
SPONSOR        : Stanford University School               PATHDATA SYSTEM V6.2c2

TEXT OF GROSS AND MICROSCOPIC FINDINGS
DOSE GROUP     :  2, Group 2

* ANIMAL NUMBER               :      841            SEX  : FEMALE
  FIRST DAY ON TEST           : 07-Oct-14
  LAST  DAY ON TEST           : 21-Oct-14
  DAYS ON TEST                :        15
  DATE OF NECROPSY            : 21-Oct-14
  DEFINED SACR.GROUP          : RECOVERY / POST-TREATMENT GROUP
  STATUS AT NECROPSY          : RECOVERY / POST-TREATMENT GROUP
.........................................................................

* NECROPSY FINDINGS

  NO NECROPSY OBSERVATIONS NOTED.

* MICROSCOPIC FINDINGS

  INJECTION SITE (TAIL):
    Severe autolysis, evaluation not possible
  PARATHYROID GLANDS:
    Tissue not present for histologic examination
  ALL OTHER PROTOCOL TISSUES WITHOUT PATHOLOGIC FINDINGS.
```

60

```
PATHOLOGY REPORT
INDIVIDUAL ANIMAL DATA                                      PROJECT     :SB-SU-003
----------------------------------------------------------------------------------
TEST ITEM      : [18F]FP-R01-MG-F2                          PATHOL. NO.: 14041 DSG
TEST SYSTEM    : RAT(SPR.DAWL.), 1 Day, I.V.                DATE       : 05-DEC-14
SPONSOR        : Stanford University School                 PATHDATA SYSTEM V6.2c2
----------------------------------------------------------------------------------
TEXT OF GROSS AND MICROSCOPIC FINDINGS
DOSE GROUP     :  2, Group 2
----------------------------------------------------------------------------------

* ANIMAL NUMBER                :     842            SEX  : FEMALE
  FIRST DAY ON TEST            : 07-Oct-14
  LAST  DAY ON TEST            : 21-Oct-14
  DAYS ON TEST                 :       15
  DATE OF NECROPSY             : 21-Oct-14
  DEFINED SACR.GROUP           : RECOVERY / POST-TREATMENT GROUP
  STATUS AT NECROPSY           : RECOVERY / POST-TREATMENT GROUP
. . . . . . . . . . . . . . . . . . . . . . . . . . . . . . . . . . . . . . . . .

* NECROPSY FINDINGS

  NO NECROPSY OBSERVATIONS NOTED.

* MICROSCOPIC FINDINGS

  INJECTION SITE (TAIL):
    Severe autolysis, evaluation not possible
  PARATHYROID GLANDS:
    Tissue not present for histologic examination
  THYROID GLAND:
    Only one of paired organs examined/present
  ALL OTHER PROTOCOL TISSUES WITHOUT PATHOLOGIC FINDINGS.
----------------------------------------------------------------------------------
```

61

Appendix B. Quality Assurance Statement

HSRL HistoScientific Research Laboratories

QUALITY ASSURANCE STATEMENT

Study Title: 14-Day Single Intravenous Dose Toxicity Study of [18F]FP-R01-MG-F2 in Sprague Dawley Rats

Client Study Number: SB-SU-003

This histopathology project has been inspected and audited by the HSRL Quality Assurance Unit (QAU) as required by the Good Laboratory Practice (GLP) regulations disseminated by the U.S. Food and Drug Administration (FDA, 21 CFR 58).

		Dates		
**Area Inspected**		Inspection[1]	Reported[2]	Reported[3]
Critical Phase:	Embedding	05NOV14	11NOV14	11NOV14
Data Review		07,10-11NOV14	17NOV14	17NOV14
Pathology Report:	Draft	19-20NOV14	21NOV14	21NOV14
	Final	04-05DEC14	05DEC14	05DEC14

All the results/conclusions of the pathology report accurately reflect the raw data.

[1] Date(s) of inspection

[2] Date(s) inspections reported to HSRL Laboratory Director/Test Site Management, Principal Investigator/HSRL Study Pathologist

[3] Date(s) inspections reported to Study Director, Test Facility Management, and Lead QA (if appropriate)

Victoria L. Cook
Victoria L. Cook, RQAP-GLP
Quality Assurance Auditor

05 December 2014
Date

Form 1501-1v3

Origination: 3/13/08
Effective: 3/14/08

APPENDIX D – CERTIFICATE OF ANALYSIS FOR VEHICLE COMPONENTS AND TEST ARTICLE

SB-SU-003

TRUE AND EXACT COPY
[illegible] 10/17/14
INITIAL / DATE

Baxter
Healthcare

MEDICATION DELIVERY
NORTH COVE FACILITY
Hwy 221 N PO Box 1390
Marion N.C. 28752
Telephone: (828) 756-4151
Fax: (828) 756-4821

Certificate of Analysis

Product: 0.9% Sodium Chloride Injection, USP
Lot #: C886374
Code: 2B1321
Manufacture/ Sterilization Date: 11/12/2012
Expiry: 11/2014

Chemical Testing per Specification: 03-15-19-061 Issue Date: 10/3/2008

TEST	LIMIT	RESULT
NaCl (g/L)	8.55 - 9.45 g/L	8.88 g/L
Sodium ID	Positive	Positive
Sodium ID- Flame	Positive	Positive
pH at 25 deg. C	4.5 - 7.0	5.6
Particle Analysis	NMT 25 ≥ 10 um/ml	NMT 25
Particle Analysis	NMT 3 ≥ 25 um/ml	NMT 3
Sterility	Pass Parametric Release	Pass

Limulus Testing per 13-01-V

RESULT	LIMIT	DISPOSITION	DATE PASSED
< 0.25 EU/ml	< 0.25 EU/ml	Pass	12/19/2012

This is to certify that this product was manufactured according to current GMP and fulfills the requirements of the Master Production Document.

Date Batch Released	Parametric Release Date	Quantity Released
12/19/2012	12/19/2012	NA

	Print Name	Signature/Date
Prepared by:	Sherrie Hopson	Sherrie Hopson 1/2/13
Approved by: Quality Manager or Designee	Keith Hawk	KHawk 1/2/13 for S. Hudson

Page 1 of 1

SB-SU-003

SIGMA-ALDRICH®

TRUE AND EXACT COPY
RT 10/17/14
INITIAL / DATE

sigma-aldrich.com
3050 Spruce Street, Saint Louis, MO 63103 USA
Website: www.sigmaaldrich.com
Email USA: techserv@sial.com
Outside USA: eurtechserv@sial.com

Certificate of Analysis

Product Name:
Ethanol - 200 proof, meets USP testing specifications

CH_3CH_2OH

**Product Number:**	**493546**
**Lot Number:**	**SHBB0212V**
Brand:	SIAL
CAS Number:	64-17-5
MDL Number:	MFCD00003568
Formula:	C2H6O
Formula Weight:	46.07 g/mol
Quality Release Date:	22 MAR 2011

Test	Specification	Result
Appearance (Color)	Colorless	Colorless
Appearance (Form)	Liquid	Liquid
Infrared spectrum	Conforms to Structure	Conforms
UV Absorbance 340nm	≤ 0.10	0.00
UV Absorbance 270nm	≤ 0.10	0.01
UV Absorbance 260nm	≤ 0.30	0.04
UV Absorbance 250nm	≤ 0.30	0.11
UV Absorbance 240nm	≤ 0.40	0.27
UV Absorbance (340 - 235nm) Absorption Curve is Smooth	Pass	Pass
Appearance (Clarity) Clear (as compared to Opalescence Reference Suspension A)	Pass	Pass
Purity (GC)	≥ 99.50%	99.87%
Impurity Benzene	≤ 2ppm	< 1ppm
Impurity as Acetaldehyde	≤ 10ppm	< 1ppm
Impurity Methanol	≤ 100ppm	< 1ppm
Impurity as Other Contaminants	≤ 300ppm	< 1ppm

Sigma-Aldrich warrants, that at the time of the quality release or subsequent retest date this product conformed to the information contained in this publication. The current Specification sheet may be available at Sigma-Aldrich.com. For further inquiries, please contact Technical Service. Purchaser must determine the suitability of the product for its particular use. See reverse side of invoice or packing slip for additional terms and conditions of sale.

Version Number: 1

Page 1 of 2

SB-SU-003

Page: 1
Date: 01/08/13 at 12:45 PM

BDH

TRUE AND EXACT COPY
[illegible] 10/17/14
INITIAL / DATE

Certificate of Analysis / QC Results

Packaged Product: Alternate code: BDH3419
Product Code: 4360
HYDROCHLORIC ACID 5.0 NORMAL

Test	Target/UOM	Range	Result
PREPARED TO FORMULATION ON FILE	YES		YES
APPEARANCE AND COLOR	CLEAR LIQUID APPEARANCE		PASS
INSTRUMENTS USED DURING PREPARATION	INSTRUMENT		T-D1,T-143
NORMALITY	5.0000 ACT NORMALITY	4.9800 - 5.0200	4.9890
EXPIRATION DATE	MM/DD/YY EXP DATE		01/30/15
COLOR (APHA)	< 10		0
N.I.S.T. Traceable to SRM 723	YES		YES

Lot # 2123149
Made 01/08/13

RM: 9765 WATER DEIONIZED REAGENT GRADE
[illegible]t: DAILY

RM: H2505 HYDROCHLORIC ACID 37% REAGENT ACS
Lot: 227610

This is to certify that the product listed above has been prepared according to the agreed-upon formulation. The solutions producer has a Quality Management System which governs each step of the manufacturing process to insure the production of a consistent product. Traceability from the producer's lot numbers to the original manufacturer's lot numbers is maintained. The lot number and description of each raw material used to prepare this product are listed above. Certificates of Analysis for these individual raw materials are available upon request.

The weights and/or volumes used to prepare this product are N.I.S.T. Traceable. All balances used in the preparation of product are calibrated daily against N.I.S.T. Traceable weights. The balances are maintained and serviced on a regular basis by an outside certified company. All volumetric glassware used is N.I.S.T. Traceable and certified as meeting Class A specifications.

Unless otherwise agreed upon, all chemicals used in the preparation of this product are Reagent ACS grade.

Sherman Nelson
Quality Manager
Aqua Solutions

SB-SU-003

Sodium Hydroxide, 5N Volumetric Solution
BAKER ANALYZED® Reagent

TRUE AND EXACT COPY
10/17/14
INITIAL / DATE

Material No.: 5671-02
Batch No.: 0000071409
Manufactured Date: 2014/02/12
Expiration Date: 2016/02/12

Certificate of Analysis

Nist Number & Date : 84L/PT

Test	Specification	Result
Appearance (Clear, colorless liquid)	Passes Test	PT
Normality	4.95 - 5.05	5.01
Chloride (Cl)	<= 5 ppm	< 3
ACS - Heavy Metals (as Pb)	<= 1 ppm	< 1
Trace Impurities - Iron (Fe)	<= 0.5 ppm	< 0.3

For Laboratory, Research or Manufacturing Use
Standardization at 25°C traceable to NIST Standard Reference Material.

Storage Conditions: Protect from air to avoid absorption of carbon dioxide.
Country of Origin: US
Packaging Site: Paris Mfg Ctr & DC

ISO
Phillipsburg, NJ 9001:2008, 14001:2004
Paris, KY 9001:2008
Mexico City, Mexico 9001:2008
Deventer, The Netherlands 9001:2008, 14001:2004, 15485:2003
Gliwice, Poland 9001:2008, 17025:2005
Selangor, Malaysia 9001:2008
Dehradun, India, 9001:2008, 14001:2004, 13485:2003
Mumbai, India, 9001:2008, 17025:2005
Panoli, India 9001:2008

Richard M Siberski
Vice President Global Quality

For questions on this Certificate of Analysis please contact Technical Services at 855.282.6867 or +1.610.573.2600

Avantor ™ Performance Materials Inc.
3477 Corporate Parkway, Suite #200, Center Valley, PA 18034, U.S.A. Phone: 610.573.2600 . Fax: 610.573.2610

C S Bio Co.
People Who Know Peptides

Quality Control Record

Product: FP-Gly-36-Gly-NH_2
Sequence: FP-Gly-Cys-Ile-Leu-Asn-Gly-Arg-Thr-Asp-Leu-Gly-Thr-Leu-Leu-Phe-Arg-Cys-Arg-Arg-Asp-Ser-Asp-Cys-Pro-Gly-Ala-Cys-Ile-Cys-Arg-Gly-Asn-Gly-Tyr-Cys-Gly-NH_2

Note: C:C = disulfide bond

Catalog No.: CS12213-P2 Expected M.W.: 3904.46 Found M.W.: 3905.55 Lot : N149

**APPEARANCE:**	**White Powder**
**MOLECULAR WEIGHT VERIFICATION:**	**Confirmed**
**PURITY:** By HPLC in TFA System:<br>Gradient: 25-46% Buffer B in 20 minutes<br>Buffer A: 0.1% TFA in H_2O<br>Buffer B: 0.1% TFA in ACN	**95.48%**
**SUGGESTIONS FOR PEPTIDE DISSOLUTION:**	**Water / Acetonitrile**
**COUNTERIONS PRESENT:**	**TFA**
**STORAGE:**	**All peptides should be stored dry at -20°C**

This material is not listed as hazardous by •NIOSH/RTECS. Therefore, no MATERIAL SAFETY DATA SHEET is required. However, the chemical, physical and toxicological properties of this product have not been thoroughly investigated. Therefore, please exercise due care when handling this material. This action is in compliance with State and Federal OSHA standards and regulations.

Quality Control: Kylin Li

Date: July 16th, 2014

C S Bio Co.
20 Kelly Court, Menlo Park, CA 94025 USA
Tel: 650-322-1111 • Fax: 650-322-2278 • Web:www.csbio.com • Email:peptides@csbio.com

C S Bio Co.
People Who Know Peptides

Compound: CS12213-P2 FP-Gly-36-Gly-NH_2 Lot Number: N149 Expected M.W.: 3904.46 Found M.W.: 3905.55

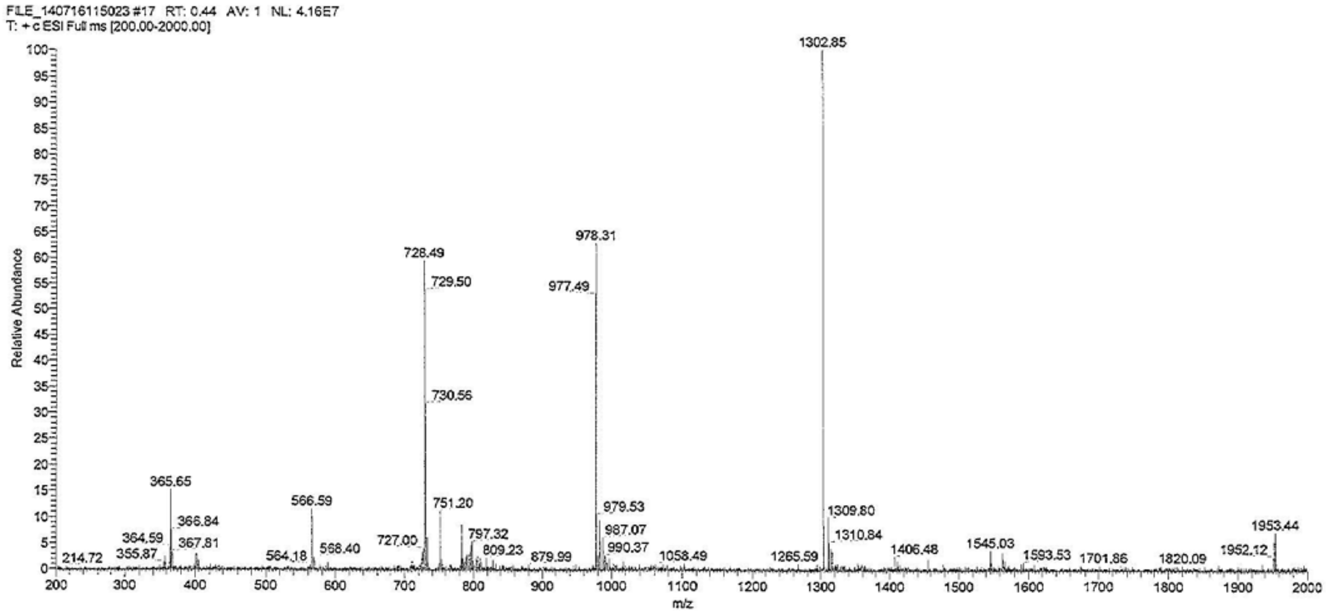

```
Data file   : D:\MYDATA\JUL2014\WG_2014-07-11_722\1BC-0101.D
Sample Name: 12213-P2-FINAL                                                   1
=========================================================================
Injection Date  : Fri, 11. Jul. 2014            Seq Line   :          1
Sample Name     : 12213-P2-FINAL                Location   :    P1-B-03
Acq Operator    : WG                            Inj. No.   :          1
Acq. Method     :                               Inj. Vol.  :      20 µl
Analysis Method : D:\MYDATA\JUL2014\WG_2014-07-11_722\25-46-20-320-200->
Last Changed    : D:\MYDATA\JUL2014\WG_2014-07-11_722\1BC-0101.D\DA.M ->
                  Wed, 16. Jul. 2014, 04:32:46 pm
                  (modified after loading)
Column: Poroshell 120 SB-C18, 4.6 x 50mm, 2.7-Micron, P.N.: 689975-902
Buffer A: 0.1%TFA in Water
Buffer B: 0.1%TFA in ACN
Flow Rate: 1ml/min
Gradient: 25% to 46% Buffer B in 20min                                  ->
```

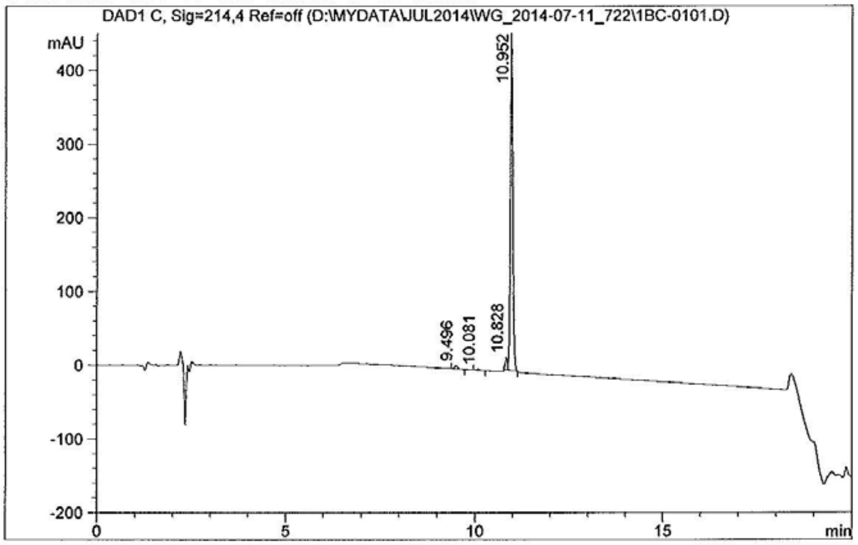

Signal 1: DAD1 C, Sig=214,4 Ref=off

Peak #	RT [min]	Type	Width [min]	Area	Area %	Name
1	9.496	BB	0.082	26.778	1.203	
2	10.081	BB	0.076	11.151	0.501	
3	10.828	MF	0.061	62.584	2.812	
4	10.952	FM	0.076	2124.753	95.483	

*** End of Report ***

Instrument 1   Wed, 16. Jul. 2014   04:33:10 pm   Page   1 of 1

AAA
Service Laboratory Inc.

Date 7/30/2014
User Stanford University
Sample ID FP-Rot-MG-F2-72814
MW 3681

Concentration 688.00 µM

Est. Concentration (uM) 150

Sequence:
GCYGNGRCICAGPCDSDRRCFLLTGLDTGRNLICG

Core Sequence
GCYGNGRCICAGPCDSDRRCFLLTGLDTGRNLICG

Volume Hydrolyzed (µL) 20
Ratio Injected 0.667

	Exp. Values	pmol analyzed	pmol protein	Over 15%	Under 15%	Observed	Theoretical
CYSO2		0	FALSE	FALSE	FALSE	0.00	0
HYP (Z)		0	FALSE	FALSE	FALSE	0.00	0
ASP (D)	42.956	46145	9229	9229	9229	5.03	5
THR (T)	16.829	18079	9039	9039	9039	2.07	2
SER (S)	8.21	8820	8820	8820	8820	1.01	1
GLU (E)	0	0	FALSE	FALSE	FALSE	0.00	0
PRO (P)	8.621	9261	9261	9261	9261	1.01	1
GLY (G)	60.35	64831	9262	9262	9262	7.06	7
ALA (A)	9.061	9734	9734	9734	9734	1.06	1
VAL (V)	0	0	FALSE	FALSE	FALSE	0.00	0
MET (M)	0	0	FALSE	FALSE	FALSE	0.00	0
ILE (I)	15.967	17153	8576	8576	8576	1.87	2
LEU (L)	33.294	35766	8941	8941	8941	3.89	4
NLE	6.209	0	FALSE	FALSE	FALSE	0.00	0
TYR (Y)	8.487	9117	9117	9117	9117	0.99	1
PHE (F)	9.051	9723	9723	9723	9723	1.06	1
HIS (H)	0	0	FALSE	FALSE	FALSE	0.00	0
HLYS	0	0	FALSE	FALSE	FALSE	0.00	0
LYS (K)	0	0	FALSE	FALSE	FALSE	0.00	0
ARG (R)	34.659	37232	9308	9308	9308	4.05	4
NLE CF	1.07						
Total	253.694	265860				29	29

Total aa's

Concentration (pmol/µL)	688 µM
Total µgrams	50.68
Concentration (mg/mL)	2.53
Peptide Content	458.7 %

Total pmol amino acid	265860
Est. pmol protein	9183
Total pmol hydrolyzed	13767
Anal. pmol protein	9183
15%	1377
-15%	7806
+15%	10560

APPENDIX E – STATISTICAL REPORT

Statistical Report for Study SB-SU-003

Patrick E. McKnight, Ph.D.

November 25, 2014

The following brief report summarizes a statistical analysis comparing two groups of treated animals according to the SB-SU-003 study protocol. Two groups (Groups 1 and 2) had total body weight (Days 1, 2 (Day 3 cohort only; pre-fasting), 3 (Day 3 cohort only; post-fasting), 8, 14 (pre-fasting) and 15 (post-fasting)) and organ weights (Days 3 and 15 by cohort) supplied in three separate comma separated text files. Each of the following analyses used original data without any transformations or alterations to the data.

Statistical Analyses Summary

All analyses reported below were conducted using the statistical package R version 3.1.2 (for ANOVA using the aov function) and the multcomp package for Dunnett's t-tests. Documentation for these statistical packages are available at the following URL's:

R: http://www.r-project.org

multcomp: http://cran.r-project.org/web/packages/multcomp

Statistics reflect differences within sex where the contrast comparison was Group 1 (negative control) and all critical p-values were set a priori at 0.05 (two-sided) but adjusted according to the Dunnett post-hoc comparison method.

Basic Descriptive Statistics

Body Weight Means and Standard Deviations

Group	Day	Mean	SD
1	1	180.60	30.11
1	2	182.16	32.42
1	3	175.72	32.14
1	8	211.85	40.55
1	14	233.00	49.15
1	15	224.19	49.14
2	1	179.93	29.08

2	2	183.30	31.08
2	3	179.47	32.85
2	8	210.83	37.64
2	14	232.03	47.12
2	15	223.16	46.06

Organ Weight Means and Standard Deviations

Group	Organ	Mean	SD
1	Brain	1.61	0.14
1	Day	9.00	6.16
1	Eyes	0.29	0.03
1	Heart	0.88	0.27
1	Kidneys	1.61	0.30
1	Liver	7.89	2.04
1	Lungs	1.64	0.33
1	OvariesF	0.16	0.02
1	Spleen	0.57	0.10
1	TestesM	3.18	0.40
1	Thyroid	0.03	0.02
2	Brain	1.62	0.11
2	Day	9.00	6.16
2	Eyes	0.30	0.04
2	Heart	0.84	0.18
2	Kidneys	1.63	0.35
2	Liver	8.25	2.06
2	Lungs	1.67	0.28
2	OvariesF	0.16	0.02
2	Spleen	0.61	0.10
2	TestesM	3.29	0.32
2	Thyroid	0.03	0.01

Body Weight ANOVA models

Standard ANOVA results

Male Results by Day

Day 1:

	Df	Sum Sq	Mean Sq	F value	Pr(>F)
Group	1	14.31	14.31	0.66	0.4275
Residuals	18	390.98	21.72		

```
        Simultaneous Tests for General Linear Hypotheses

Multiple Comparisons of Means: Dunnett Contrasts


Fit: aov(formula = Bodywt ~ Group, data = subset(Body, Sex == "M" &
    Day == 1))

Linear Hypotheses:
           Estimate Std. Error t value Pr(>|t|)
2 - 1 == 0   -1.692      2.084  -0.812    0.428
(Adjusted p values reported -- single-step method)
```

Day 2:

	Df	Sum Sq	Mean Sq	F value	Pr(>F)
Group	1	0.05	0.05	0.00	0.9742
Residuals	8	361.44	45.18		

```
        Simultaneous Tests for General Linear Hypotheses

Multiple Comparisons of Means: Dunnett Contrasts


Fit: aov(formula = Bodywt ~ Group, data = subset(Body, Sex == "M" &
    Day == 2))

Linear Hypotheses:
           Estimate Std. Error t value Pr(>|t|)
2 - 1 == 0   -0.142      4.251  -0.033    0.974
(Adjusted p values reported -- single-step method)
```

Day 3:

	Df	Sum Sq	Mean Sq	F value	Pr(>F)
Group	1	39.60	39.60	0.58	0.4675
Residuals	8	544.62	68.08		

```
        Simultaneous Tests for General Linear Hypotheses

Multiple Comparisons of Means: Dunnett Contrasts


Fit: aov(formula = Bodywt ~ Group, data = subset(Body, Sex == "M" &
    Day == 3))
```

```
Linear Hypotheses:
           Estimate Std. Error t value Pr(>|t|)
2 - 1 == 0    3.980      5.218   0.763    0.468
(Adjusted p values reported -- single-step method)
```

Day 8:

	Df	Sum Sq	Mean Sq	F value	Pr(>F)
Group	1	36.48	36.48	3.17	0.1128
Residuals	8	92.05	11.51		

```
         Simultaneous Tests for General Linear Hypotheses

Multiple Comparisons of Means: Dunnett Contrasts


Fit: aov(formula = Bodywt ~ Group, data = subset(Body, Sex == "M" &
    Day == 8))

Linear Hypotheses:
           Estimate Std. Error t value Pr(>|t|)
2 - 1 == 0   -3.820      2.145  -1.781    0.113
(Adjusted p values reported -- single-step method)
```

Day 14:

	Df	Sum Sq	Mean Sq	F value	Pr(>F)
Group	1	21.43	21.43	0.69	0.4314
Residuals	8	249.81	31.23		

```
         Simultaneous Tests for General Linear Hypotheses

Multiple Comparisons of Means: Dunnett Contrasts


Fit: aov(formula = Bodywt ~ Group, data = subset(Body, Sex == "M" &
    Day == 14))

Linear Hypotheses:
           Estimate Std. Error t value Pr(>|t|)
2 - 1 == 0   -2.928      3.534  -0.828    0.431
(Adjusted p values reported -- single-step method)
```

Day 15:

	Df	Sum Sq	Mean Sq	F value	Pr(>F)
Group	1	38.93	38.93	1.34	0.2798
Residuals	8	231.71	28.96		

```
         Simultaneous Tests for General Linear Hypotheses

Multiple Comparisons of Means: Dunnett Contrasts


Fit: aov(formula = Bodywt ~ Group, data = subset(Body, Sex == "M" &
    Day == 15))

Linear Hypotheses:
           Estimate Std. Error t value Pr(>|t|)
2 - 1 == 0   -3.946      3.404  -1.159     0.28
(Adjusted p values reported -- single-step method)
```

Female Results

Day 1:

	Df	Sum Sq	Mean Sq	F value	Pr(>F)
Group	1	0.60	0.60	0.04	0.8368
Residuals	18	248.05	13.78		

```
         Simultaneous Tests for General Linear Hypotheses

Multiple Comparisons of Means: Dunnett Contrasts


Fit: aov(formula = Bodywt ~ Group, data = subset(Body, Sex == "F" &
    Day == 1))

Linear Hypotheses:
           Estimate Std. Error t value Pr(>|t|)
2 - 1 == 0    0.347      1.660   0.209    0.837
(Adjusted p values reported -- single-step method)
```

Day 2:

	Df	Sum Sq	Mean Sq	F value	Pr(>F)
Group	1	14.79	14.79	1.04	0.3370
Residuals	8	113.39	14.17		

```
         Simultaneous Tests for General Linear Hypotheses
```

```
Multiple Comparisons of Means: Dunnett Contrasts


Fit: aov(formula = Bodywt ~ Group, data = subset(Body, Sex == "F" &
    Day == 2))

Linear Hypotheses:
           Estimate Std. Error t value Pr(>|t|)
2 - 1 == 0    2.432      2.381   1.021    0.337
(Adjusted p values reported -- single-step method)
```

Day 3:

	Df	Sum Sq	Mean Sq	F value	Pr(>F)
Group	1	30.70	30.70	6.24	0.0370
Residuals	8	39.33	4.92		

```
         Simultaneous Tests for General Linear Hypotheses

Multiple Comparisons of Means: Dunnett Contrasts


Fit: aov(formula = Bodywt ~ Group, data = subset(Body, Sex == "F" &
    Day == 3))

Linear Hypotheses:
           Estimate Std. Error t value Pr(>|t|)
2 - 1 == 0    3.504      1.402   2.499    0.037 *
---
Signif. codes:  0 âĂŸ***âĂŹ 0.001 âĂŸ**âĂŹ 0.01 âĂŸ*âĂŹ 0.05 âĂŸ.âĂŹ 0.1 âĂŸ âĂŹ 1
(Adjusted p values reported -- single-step method)
```

Day 8:

	Df	Sum Sq	Mean Sq	F value	Pr(>F)
Group	1	7.81	7.81	0.16	0.6961
Residuals	8	381.23	47.65		

```
         Simultaneous Tests for General Linear Hypotheses

Multiple Comparisons of Means: Dunnett Contrasts


Fit: aov(formula = Bodywt ~ Group, data = subset(Body, Sex == "F" &
    Day == 8))
```

```
Linear Hypotheses:
           Estimate Std. Error t value Pr(>|t|)
2 - 1 == 0    1.768      4.366   0.405    0.696
(Adjusted p values reported -- single-step method)
```

Day 14:

	Df	Sum Sq	Mean Sq	F value	Pr(>F)
Group	1	2.46	2.46	0.03	0.8680
Residuals	8	668.50	83.56		

```
         Simultaneous Tests for General Linear Hypotheses

Multiple Comparisons of Means: Dunnett Contrasts


Fit: aov(formula = Bodywt ~ Group, data = subset(Body, Sex == "F" &
    Day == 14))

Linear Hypotheses:
           Estimate Std. Error t value Pr(>|t|)
2 - 1 == 0    0.992      5.781   0.172    0.868
(Adjusted p values reported -- single-step method)
```

Day 15:

	Df	Sum Sq	Mean Sq	F value	Pr(>F)
Group	1	8.97	8.97	0.16	0.6994
Residuals	8	447.57	55.95		

```
         Simultaneous Tests for General Linear Hypotheses

Multiple Comparisons of Means: Dunnett Contrasts


Fit: aov(formula = Bodywt ~ Group, data = subset(Body, Sex == "F" &
    Day == 15))

Linear Hypotheses:
           Estimate Std. Error t value Pr(>|t|)
2 - 1 == 0    1.894      4.731     0.4    0.699
(Adjusted p values reported -- single-step method)
```

Summary

There was significant Sex by Day differences for Females on Day 3 observed in the above results. A significant difference exists between the experimental group (Group 2) and the control group (Group 1) where the Female experimental group weighed significantly more than the Female control group on Day 3. The sole difference finding might better be represented box and whisker plots - presented below.

Males

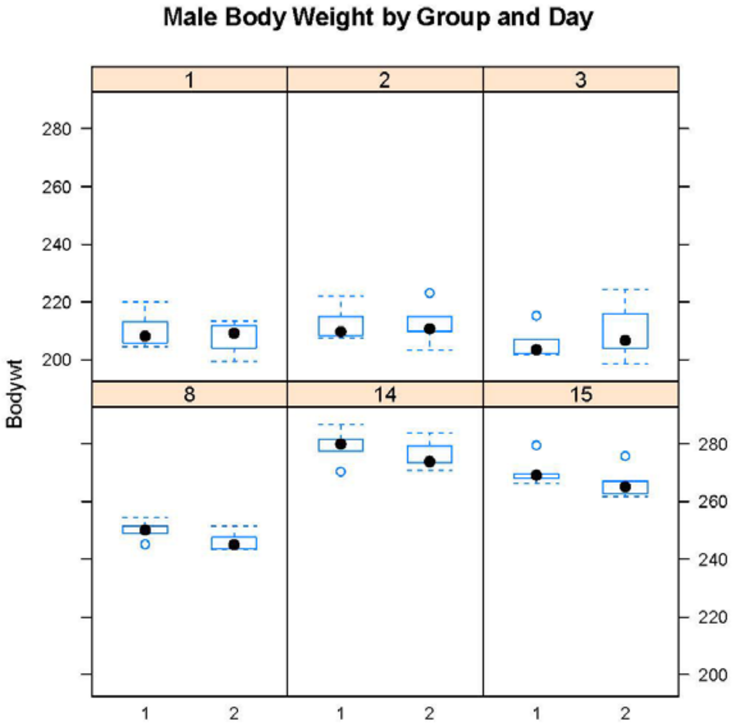

Females

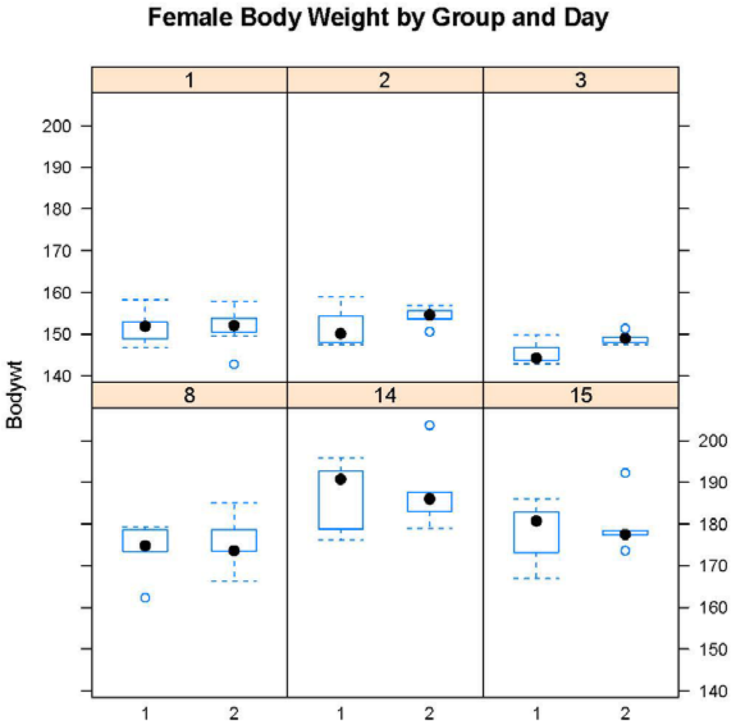

Organ Weight ANOVA models

Brain

Male

	Df	Sum Sq	Mean Sq	F value	Pr(>F)
Group	1	0.01	0.01	0.26	0.6159
Residuals	18	0.41	0.02		

```
         Simultaneous Tests for General Linear Hypotheses

Multiple Comparisons of Means: Dunnett Contrasts
```

9

```
Fit: aov(formula = Brain ~ Group, data = subset(Organ, Sex == "M"))

Linear Hypotheses:
           Estimate Std. Error t value Pr(>|t|)
2 - 1 == 0  0.03452    0.06763    0.51    0.616
(Adjusted p values reported -- single-step method)
```

Female

	Df	Sum Sq	Mean Sq	F value	Pr(>F)
Group	1	0.00	0.00	0.18	0.6798
Residuals	18	0.16	0.01		

```
         Simultaneous Tests for General Linear Hypotheses

Multiple Comparisons of Means: Dunnett Contrasts


Fit: aov(formula = Brain ~ Group, data = subset(Organ, Sex == "F"))

Linear Hypotheses:
           Estimate Std. Error t value Pr(>|t|)
2 - 1 == 0 -0.01763    0.04202   -0.42     0.68
(Adjusted p values reported -- single-step method)
```

Eyes

Males

	Df	Sum Sq	Mean Sq	F value	Pr(>F)
Group	1	0.00	0.00	2.03	0.1710
Residuals	18	0.03	0.00		

```
         Simultaneous Tests for General Linear Hypotheses

Multiple Comparisons of Means: Dunnett Contrasts


Fit: aov(formula = Eyes ~ Group, data = subset(Organ, Sex == "M"))

Linear Hypotheses:
           Estimate Std. Error t value Pr(>|t|)
2 - 1 == 0  0.02421    0.01698   1.426    0.171
(Adjusted p values reported -- single-step method)
```

Females

	Df	Sum Sq	Mean Sq	F value	Pr(>F)
Group	1	0.00	0.00	0.02	0.8829
Residuals	18	0.02	0.00		

```
         Simultaneous Tests for General Linear Hypotheses

Multiple Comparisons of Means: Dunnett Contrasts


Fit: aov(formula = Eyes ~ Group, data = subset(Organ, Sex == "F"))

Linear Hypotheses:
           Estimate Std. Error t value Pr(>|t|)
2 - 1 == 0 -0.00196    0.01312  -0.149    0.883
(Adjusted p values reported -- single-step method)
```

Heart

Male

	Df	Sum Sq	Mean Sq	F value	Pr(>F)
Group	1	0.05	0.05	1.83	0.1930
Residuals	18	0.50	0.03		

```
         Simultaneous Tests for General Linear Hypotheses

Multiple Comparisons of Means: Dunnett Contrasts


Fit: aov(formula = Heart ~ Group, data = subset(Organ, Sex == "M"))

Linear Hypotheses:
           Estimate Std. Error t value Pr(>|t|)
2 - 1 == 0 -0.10045    0.07427  -1.352    0.193
(Adjusted p values reported -- single-step method)
```

Female

	Df	Sum Sq	Mean Sq	F value	Pr(>F)
Group	1	0.00	0.00	0.64	0.4344
Residuals	18	0.05	0.00		

```
         Simultaneous Tests for General Linear Hypotheses
```

```
Multiple Comparisons of Means: Dunnett Contrasts


Fit: aov(formula = Heart ~ Group, data = subset(Organ, Sex == "F"))

Linear Hypotheses:
           Estimate Std. Error t value Pr(>|t|)
2 - 1 == 0  0.01879    0.02350     0.8    0.434
(Adjusted p values reported -- single-step method)
```

Kidneys

Male

	Df	Sum Sq	Mean Sq	F value	Pr(>F)
Group	1	0.01	0.01	0.27	0.6095
Residuals	18	0.94	0.05		

```
         Simultaneous Tests for General Linear Hypotheses

Multiple Comparisons of Means: Dunnett Contrasts


Fit: aov(formula = Kidneys ~ Group, data = subset(Organ, Sex == "M"))

Linear Hypotheses:
           Estimate Std. Error t value Pr(>|t|)
2 - 1 == 0  0.05303    0.10202    0.52     0.61
(Adjusted p values reported -- single-step method)
```

Female

	Df	Sum Sq	Mean Sq	F value	Pr(>F)
Group	1	0.00	0.00	0.26	0.6155
Residuals	18	0.17	0.01		

```
         Simultaneous Tests for General Linear Hypotheses

Multiple Comparisons of Means: Dunnett Contrasts


Fit: aov(formula = Kidneys ~ Group, data = subset(Organ, Sex == "F"))

Linear Hypotheses:
           Estimate Std. Error t value Pr(>|t|)
```

```
2 - 1 == 0 -0.02236    0.04375  -0.511    0.616
(Adjusted p values reported -- single-step method)
```

Liver

Male

	Df	Sum Sq	Mean Sq	F value	Pr(>F)
Group	1	0.63	0.63	0.24	0.6318
Residuals	18	47.71	2.65		

```
         Simultaneous Tests for General Linear Hypotheses

Multiple Comparisons of Means: Dunnett Contrasts


Fit: aov(formula = Liver ~ Group, data = subset(Organ, Sex == "M"))

Linear Hypotheses:
           Estimate Std. Error t value Pr(>|t|)
2 - 1 == 0   0.3549     0.7281   0.487    0.632
(Adjusted p values reported -- single-step method)
```

Female

	Df	Sum Sq	Mean Sq	F value	Pr(>F)
Group	1	0.73	0.73	1.66	0.2136
Residuals	18	7.88	0.44		

```
         Simultaneous Tests for General Linear Hypotheses

Multiple Comparisons of Means: Dunnett Contrasts


Fit: aov(formula = Liver ~ Group, data = subset(Organ, Sex == "F"))

Linear Hypotheses:
           Estimate Std. Error t value Pr(>|t|)
2 - 1 == 0   0.3816     0.2959   1.289    0.214
(Adjusted p values reported -- single-step method)
```

Lungs

Male

	Df	Sum Sq	Mean Sq	F value	Pr(>F)
Group	1	0.00	0.00	0.04	0.8450
Residuals	18	1.56	0.09		

```
         Simultaneous Tests for General Linear Hypotheses

Multiple Comparisons of Means: Dunnett Contrasts


Fit: aov(formula = Lungs ~ Group, data = subset(Organ, Sex == "M"))

Linear Hypotheses:
           Estimate Std. Error t value Pr(>|t|)
2 - 1 == 0  -0.0261     0.1316  -0.198    0.845
(Adjusted p values reported -- single-step method)
```

Female

	Df	Sum Sq	Mean Sq	F value	Pr(>F)
Group	1	0.05	0.05	1.29	0.2702
Residuals	18	0.63	0.04		

```
         Simultaneous Tests for General Linear Hypotheses

Multiple Comparisons of Means: Dunnett Contrasts


Fit: aov(formula = Lungs ~ Group, data = subset(Organ, Sex == "F"))

Linear Hypotheses:
           Estimate Std. Error t value Pr(>|t|)
2 - 1 == 0  0.09524    0.08372   1.138     0.27
(Adjusted p values reported -- single-step method)
```

Ovaries (Females Only)

Female

	Df	Sum Sq	Mean Sq	F value	Pr(>F)
Group	1	0.00	0.00	0.17	0.6850
Residuals	16	0.01	0.00		

```
        Simultaneous Tests for General Linear Hypotheses

Multiple Comparisons of Means: Dunnett Contrasts


Fit: aov(formula = OvariesF ~ Group, data = subset(Organ, Sex == "F"))

Linear Hypotheses:
            Estimate Std. Error t value Pr(>|t|)
2 - 1 == 0 -0.004289   0.010383  -0.413    0.685
(Adjusted p values reported -- single-step method)
```

Spleen

Male

	Df	Sum Sq	Mean Sq	F value	Pr(>F)
Group	1	0.02	0.02	2.47	0.1334
Residuals	18	0.15	0.01		

```
        Simultaneous Tests for General Linear Hypotheses

Multiple Comparisons of Means: Dunnett Contrasts


Fit: aov(formula = Spleen ~ Group, data = subset(Organ, Sex == "M"))

Linear Hypotheses:
           Estimate Std. Error t value Pr(>|t|)
2 - 1 == 0  0.06416    0.04081   1.572    0.133
(Adjusted p values reported -- single-step method)
```

Female

	Df	Sum Sq	Mean Sq	F value	Pr(>F)
Group	1	0.00	0.00	0.04	0.8428
Residuals	18	0.09	0.00		

```
        Simultaneous Tests for General Linear Hypotheses

Multiple Comparisons of Means: Dunnett Contrasts


Fit: aov(formula = Spleen ~ Group, data = subset(Organ, Sex == "F"))
```

15

```
Linear Hypotheses:
           Estimate Std. Error t value Pr(>|t|)
2 - 1 == 0  0.00624    0.03102   0.201    0.843
(Adjusted p values reported -- single-step method)
```

Testes (Males Only)

Male

	Df	Sum Sq	Mean Sq	F value	Pr(>F)
Group	1	0.05	0.05	0.40	0.5351
Residuals	18	2.39	0.13		

```
         Simultaneous Tests for General Linear Hypotheses

Multiple Comparisons of Means: Dunnett Contrasts


Fit: aov(formula = TestesM ~ Group, data = subset(Organ, Sex == "M"))

Linear Hypotheses:
           Estimate Std. Error t value Pr(>|t|)
2 - 1 == 0   0.1031     0.1631   0.632    0.535
(Adjusted p values reported -- single-step method)
```

Thyroid (with parathyroid)

Male

	Df	Sum Sq	Mean Sq	F value	Pr(>F)
Group	1	0.00	0.00	0.96	0.3394
Residuals	18	0.00	0.00		

```
         Simultaneous Tests for General Linear Hypotheses

Multiple Comparisons of Means: Dunnett Contrasts


Fit: aov(formula = Thyroid ~ Group, data = subset(Organ, Sex == "M"))

Linear Hypotheses:
            Estimate Std. Error t value Pr(>|t|)
2 - 1 == 0 -0.006940   0.007072  -0.981    0.339
(Adjusted p values reported -- single-step method)
```

Female

	Df	Sum Sq	Mean Sq	F value	Pr(>F)
Group	1	0.00	0.00	1.57	0.2275
Residuals	17	0.00	0.00		

```
         Simultaneous Tests for General Linear Hypotheses

Multiple Comparisons of Means: Dunnett Contrasts


Fit: aov(formula = Thyroid ~ Group, data = subset(Organ, Sex == "F"))

Linear Hypotheses:
            Estimate Std. Error t value Pr(>|t|)
2 - 1 == 0 0.007668   0.006124   1.252    0.227
(Adjusted p values reported -- single-step method)
```

There were no significant effects observed in the Sex by Organ weight ANOVA models presented above.

General Summary

The comparisons between the experimental and control group produced one significant result for body weight (Females in the experimental group weighed significantly more than the Females in the control group on Day 3) and none for organ weights. A single significant finding from more than 20 protected tests still falls within the expected error and should not signify a meaningful effect.

I submitted this report with source code for the analysis and data files analyzed on November 25th, 2014.

Patrick E. McKnight, Ph.D.
McKnight Consulting, LLC

17
