# Supplementary material for: Evaluation of integrin αvβ6 cystine knot PET tracers to detect cancer and idiopathic pulmonary fibrosis
Source: Nat Commun. 2019 Oct 14;10:4673. doi: 10.1038/s41467-019-11863-w (PMC6791878; doi:10.1038/s41467-019-11863-w)
Supplement: Supplementary file 3 — Description of Additional Supplementary Files [file 41467_2019_11863_MOESM3_ESM.pdf]

Description of Additional Supplementary Files

File Name: Supplementary Movie 1

Description: [^{18}F]FP-R01-MG-F2 Maximum Intensity Projection PET of a representative Healthy Volunteer. Tracer biodistribution is shown at 5 min after tracer administration.

File Name: Supplementary Movie 2

Description: [^{18}F]FP-R01-MG-F2 Maximum Intensity Projection PET of a representative Healthy Volunteer. Tracer biodistribution is shown at 60 min after tracer administration.

File Name: Supplementary Movie 3

Description: [^{18}F]FP-R01-MG-F2 Maximum Intensity Projection PET of a representative Healthy Volunteer. Tracer biodistribution is shown at 120 min after tracer administration.

File Name: Supplementary Movie 4

Description: [^{18}F]FP-R01-MG-F2 Volume Rendered PET/CT Imaging Pancreatic Cancer Patient, 1 hour after injection. Close Up. Transparent arrow denotes position of the tumor in frame 1.

File Name: Supplementary Movie 5

Description: [^{18}F]FDG Volume Rendered PET/CT Imaging Pancreatic Cancer Patient, 1 hour after injection. Transparent arrow denotes position of the tumor in frame 1.

File Name: Supplementary Movie 6

Description: [^{68}Ga]NODAGA-R01-MG Volume Rendered PET/CT Imaging Pancreatic Cancer Patient, 1 hour after injection. Close Up. Static Images are described in Figure 4C. Transparent arrow denotes position of the tumor in frame 1.

File Name: Supplementary Movie 7

Description: [^{68}Ga]NODAGA-R01-MG Volume Rendered PET/CT Imaging Pancreatic Cancer Patient, 1 hour after injection. Close Up. Static Images are described in Figure 1. Transparent arrow denotes position of the tumor in frame 1.

File Name: Supplementary Movie 8

Description: [^{68}Ga]NODAGA-R01-MG Volume Rendered PET/CT Imaging Cervical Cancer Patient, 1 hour after injection. Close Up. Static Images are described in Figure 6. Transparent arrow denotes position of the tumor in frame 1.

File Name: Supplementary Movie 9

Description: [^{68}Ga]NODAGA-R01-MG Volume Rendered PET/CT Imaging Cervical Cancer Patient, 1 hour after injection. Close Up. Static Images are described in Supplementary Figure 13. Transparent arrow denotes position of the tumor in frame 1.

File Name: Supplementary Movie 10

Description: [^{18}F]FP-R01-MG-F2 15 minute dynamic PET scan (axial view) of patient IPF-1, a 71-year-old male former pipe smoker (40 years) with a history of inhalational exposure to occupational risk agents and birds. The light blue arrows indicate regions of fibrosis and the white arrows indicate relatively healthy regions.

File Name: Supplementary Movie 11

Description: [^{18}F]FP-R01-MG-F2 15 minute dynamic PET scan of IPF-1 shown in sagittal view.

File Name: Supplementary Movie 12

Description: [^{18}F]FP-R01-MG-F2 15 minute dynamic PET scan of IPF-1 shown in coronal view.

File Name: Supplementary Movie 13

Description: [^{18}F]FP-R01-MG-F2 volume rendered PET scan of IPF-1 approximately 1 hr after tracer administration.
