# Supplementary material for: Evaluation of integrin αvβ6 cystine knot PET tracers to detect cancer and idiopathic pulmonary fibrosis
Source: Nat Commun. 2019 Oct 14;10:4673. doi: 10.1038/s41467-019-11863-w (PMC6791878; doi:10.1038/s41467-019-11863-w)
Supplement: Supplementary file 17 — Reporting Summary [file 41467_2019_11863_MOESM17_ESM.pdf]

nature research

Corresponding author(s): Sanjiv Sam Gambhir

Last updated by author(s): Jul 5, 2019

Reporting Summary

Nature Research wishes to improve the reproducibility of the work that we publish. This form provides structure for consistency and transparency in reporting. For further information on Nature Research policies, see Authors & Referees and the Editorial Policy Checklist.

Statistics

For all statistical analyses, confirm that the following items are present in the figure legend, table legend, main text, or Methods section.

n/a	Confirmed	
☐	☒	The exact sample size (*n*) for each experimental group/condition, given as a discrete number and unit of measurement
☐	☒	A statement on whether measurements were taken from distinct samples or whether the same sample was measured repeatedly
☐	☒	The statistical test(s) used AND whether they are one- or two-sided<br>*Only common tests should be described solely by name; describe more complex techniques in the Methods section.*
☐	☒	A description of all covariates tested
☐	☒	A description of any assumptions or corrections, such as tests of normality and adjustment for multiple comparisons
☐	☒	A full description of the statistical parameters including central tendency (e.g. means) or other basic estimates (e.g. regression coefficient) AND variation (e.g. standard deviation) or associated estimates of uncertainty (e.g. confidence intervals)
☒	☐	For null hypothesis testing, the test statistic (e.g. *F*, *t*, *r*) with confidence intervals, effect sizes, degrees of freedom and *P* value noted<br>*Give P values as exact values whenever suitable.*
☒	☐	For Bayesian analysis, information on the choice of priors and Markov chain Monte Carlo settings
☒	☐	For hierarchical and complex designs, identification of the appropriate level for tests and full reporting of outcomes
☒	☐	Estimates of effect sizes (e.g. Cohen's *d*, Pearson's *r*), indicating how they were calculated

Our web collection on statistics for biologists contains articles on many of the points above.

Software and code

Policy information about availability of computer code

Data collection	n/a
Data analysis	Microsoft Excel, Kaleidagraph (Synergy Software), Stata Release 15.1 (StataCorp) Inveon Research Workstation (Siemens)

For manuscripts utilizing custom algorithms or software that are central to the research but not yet described in published literature, software must be made available to editors/reviewers. We strongly encourage code deposition in a community repository (e.g. GitHub). See the Nature Research guidelines for submitting code & software for further information.

Data

Policy information about availability of data

All manuscripts must include a data availability statement. This statement should provide the following information, where applicable:

- Accession codes, unique identifiers, or web links for publicly available datasets
- A list of figures that have associated raw data
- A description of any restrictions on data availability

Source data are provided as a Source Data file.

Field-specific reporting

Please select the one below that is the best fit for your research. If you are not sure, read the appropriate sections before making your selection.

☒ Life sciences ☐ Behavioural & social sciences ☐ Ecological, evolutionary & environmental sciences

For a reference copy of the document with all sections, see nature.com/documents/nr-reporting-summary-flat.pdf

Life sciences study design

All studies must disclose on these points even when the disclosure is negative.

Sample size	The number of replicates were based on whether statistical significance, where informative or necessary, could be determined.
Data exclusions	Data was not excluded.
Replication	Positive and negative controls were used in the studies.
Randomization	Randomization was not used.
Blinding	Blinding was not used.

Reporting for specific materials, systems and methods

We require information from authors about some types of materials, experimental systems and methods used in many studies. Here, indicate whether each material, system or method listed is relevant to your study. If you are not sure if a list item applies to your research, read the appropriate section before selecting a response.

Materials & experimental systems

n/a	Involved in the study	
☐	☒	Antibodies
☐	☒	Eukaryotic cell lines
☒	☐	Palaeontology
☐	☒	Animals and other organisms
☐	☒	Human research participants
☐	☒	Clinical data

Methods

n/a	Involved in the study	
☒	☐	ChIP-seq
☒	☐	Flow cytometry
☒	☐	MRI-based neuroimaging

Antibodies

Antibodies used	1. AB_2535826, Cat # A-21281 (ThermoFisher), 2. AB_2535858, Cat # A-21427 (ThermoFisher), 3. Clone NK1-M9, Cat # 327908, (Biolegend), 4. Product # 6.2A1 (Biogen Idec)
Validation	Antibodies were validated by the manufacturer.

Eukaryotic cell lines

Policy information about cell lines

Cell line source(s)	1. BxPC3 (ATCC), 2. A431 (ATCC), 3. HEK 293T (Lab Stock)
Authentication	Expression of integrin avb6 was verified by flow cytometry.
Mycoplasma contamination	Cell lines were negative for mycoplasma.
Commonly misidentified lines (See ICLAC register)	Commonly misidentified cell lines were not used.

Animals and other organisms

Policy information about studies involving animals; ARRIVE guidelines recommended for reporting animal research

Laboratory animals	Mice: Nu/Nu Female 6-8 weeks old.
Wild animals	n/a
Field-collected samples	n/a
Ethics oversight	Stanford University Administrative Panels on Laboratory Animal Care

Note that full information on the approval of the study protocol must also be provided in the manuscript.

Human research participants

Policy information about studies involving human research participants

Population characteristics	Healthy volunteers, and patients with cancer or IPF.
Recruitment	Extensive inclusion and exclusion criteria are described in the methods section.
Ethics oversight	Stanford's Institutional Review Board and Scientific Review Committee

Note that full information on the approval of the study protocol must also be provided in the manuscript.

Clinical data

Policy information about clinical studies

All manuscripts should comply with the ICMJE guidelines for publication of clinical research and a completed CONSORT checklist must be included with all submissions.

Clinical trial registration	FDA eIND 126379, ClinicalTrials.gov Identifier NCT02683824
Study protocol	The study protocol is fully stated in the methods section of the manuscript.
Data collection	Data was collected at Stanford University and Peking University Medical College.
Outcomes	Uptake of PET tracer in diseased organs/tissues.
